# Advancing New Approach Methodologies (NAMs) for Tobacco Harm Reduction: Synopsis from the 2021 CORESTA SSPT—NAMs Symposium

**DOI:** 10.3390/toxics10120760

**Published:** 2022-12-06

**Authors:** Kyeonghee Monica Lee, Richard Corley, Annie M. Jarabek, Nicole Kleinstreuer, Alicia Paini, Andreas O. Stucki, Shannon Bell

**Affiliations:** 1 Altria Client Services, 601 E Jackson St, Richmond, VA 23219, USA; 2 Greek Creek Toxicokinetics Consulting, LLC, Boise, ID 83714, USA; 3 Office of Research and Development, U.S. Environmental Protection Agency (EPA), Research Triangle Park, NC 27711, USA; 4 National Toxicology Program Interagency Center for Evaluation of Alternative Toxicological Methods (NICEATM), Research Triangle Park, NC 27711, USA; 5 European Commission Joint Research Center (EC JRC), 2749 Ispra, Italy; 6 PETA Science Consortium International e.V., 70499 Stuttgart, Germany; 7 Inotiv-RTP, Research Triangle Park, NC 27709, USA

**Keywords:** NAMS, tobacco harm reduction, integrated approaches

## Abstract

New approach methodologies (NAMs) are emerging chemical safety assessment tools consisting of in vitro and in silico (computational) methodologies intended to reduce, refine, or replace (3R) various in vivo animal testing methods traditionally used for risk assessment. Significant progress has been made toward the adoption of NAMs for human health and environmental toxicity assessment. However, additional efforts are needed to expand their development and their use in regulatory decision making. A virtual symposium was held during the 2021 Cooperation Centre for Scientific Research Relative to Tobacco (CORESTA) Smoke Science and Product Technology (SSPT) conference (titled “Advancing New Alternative Methods for Tobacco Harm Reduction”), with the goals of introducing the concepts and potential application of NAMs in the evaluation of potentially reduced-risk (PRR) tobacco products. At the symposium, experts from regulatory agencies, research organizations, and NGOs shared insights on the status of available tools, strengths, limitations, and opportunities in the application of NAMs using case examples from safety assessments of chemicals and tobacco products. Following seven presentations providing background and application of NAMs, a discussion was held where the presenters and audience discussed the outlook for extending the NAMs toxicological applications for tobacco products. The symposium, endorsed by the CORESTA In Vitro Tox Subgroup, Biomarker Subgroup, and NextG Tox Task Force, illustrated common ground and interest in science-based engagement across the scientific community and stakeholders in support of tobacco regulatory science. Highlights of the symposium are summarized in this paper.

## 1. NAM-00: Introduction

New approach methodologies (NAMs) are any technologies, methods, approaches, or combinations that inform chemical hazard and risk assessment and that replace or reduce the use of animals, including in silico, in chemico, in vitro, and ex vivo approaches [1,2]. These animal testing alternatives do not necessarily seek a 1-to-1 replacement with animal tests. Instead, NAMs use multiple lines of evidence from non-animal methods, enabling an integrated and mechanism-based toxicological risk assessment. Since the seminal 2007 National Research Council’s report on 21st century toxicology testing [3] was released, there have been significant advancements to using NAMs in toxicological assessments of chemicals (“Alternative Methods Accepted by US Agencies”, accessed May 2022 [4]). NAMs pursue more human-relevant, reliable, and efficient means to assess chemicals and are already being applied to replace some traditional in vivo animal tests [5,6,7].

The potential of NAMs for assessment of a variety of rapidly evolving, smoke-free alternative tobacco and nicotine products is especially of interest. Many of these alternative products have significantly fewer harmful chemicals found in tobacco cigarette smoke, adding potential for reduced biological risk compared to cigarettes [8,9,10,11,12,13]. At the same time, these products are not homogeneous, containing nicotine and other non-tobacco ingredients. Therefore, such products are not risk-free, and their potential toxicity remains unknown, particularly with long-term use. Using NAMs in pre-marketing applications for these potentially reduced-risk (PRR) tobacco products would enable more efficient and human-relevant assessments while limiting or avoiding in vivo testing.

A virtual symposium, “Advancing NAMs for Tobacco Harm Reduction” (Table 1; presentations in Supplemental File), was held on 19 October 2021, during the annual CORESTA SSPT conference (https://www.coresta.org/events/smoke-techno-conference-sspt2021-34597.html, accessed on 1 March 2022), to introduce the CORESTA community to NAMs concepts and potential applications for evaluating PRR products. Reflecting on recent advances in NAM applications, experts from regulatory agencies, research organizations, and NGOs presented the latest status, tools, and case examples. The presentations were followed by a live discussion where the presenters and audience discussed gaps and opportunities in applying NAMs for supporting the reduced risk potential of smoke-free alternative tobacco and nicotine products. For instance, some non-tobacco ingredients (e.g., flavor ingredients and humectants) in electronic nicotine delivery systems (ENDS) are generally recognized as safe (GRAS) but only for systemic toxicity via food or oral consumption. That is, PRR products under the conditions of long-term inhalation exposures are unknown and present challenges related to insufficient characterization of toxicity profiles [14]. At the same time, diverse smoke-free PRR alternatives that include both inhaled (e.g., ENDS, heated tobacco products) and oral use (e.g., tobacco-free nicotine pouches) products are rapidly evolving in the global market, albeit with limited information on human health risk. There has been much interest in advancing NAMs for assessing these products, as it is not scientifically or ethically desirable to test all PRR products in animals.

Evaluating PRR products requires tiered and weight-of-evidence toxicological assessment (NAM-00; Lee and Bell, Supplemental). For inhalable products, when no inhalation data are available, the default approach would be to conduct regulatory in vivo testing in rodents, for example, to identify a benchmark dose (benchmark dose (BMD) is the dose level corresponding to specific response levels at the low end of the concentration response curve; https://www.epa.gov/sites/default/files/2015-01/documents/benchmark_dose_guidance.pdf, accessed on 1 March 2022; https://www.epa.gov/sites/default/files/2015-01/documents/benchmark_dose_guidance.pdf, accessed on 1 March 2022) or the no observed adverse effect level (NOAEL) needed in quantitative risk assessment. While these in vivo tests are often considered the gold standard, they are resource- and time-intensive and may be of limited human relevance, particularly if physiological differences for inhalation dosimetry between humans and rodents are not characterized. Recognizing these limitations of the traditional animal test methods, NAMs are designed to be more human-relevant and target the mode of action. NAMs can have greater predictive utility, as they rely on the use of computational tools and targeted cellular in vitro testing to overcome the gaps posed by traditional toxicology [15,16]. In addition to developing novel tools, NAMs reframe the question from the current “what in vivo exposure leads to a NOAEL” to “what cellular (in vitro) exposure produces no adverse effect?”. Like traditional animal testing, NAMS also asks “how does this correspond to an in vivo exposure threshold or acceptable level?”. This reframing requires comprehensive integration of several scientific domains (e.g., exposure, delivery, adverse outcomes) to address exposures for humans. However, instead of identifying the dose–response relationship from in vivo studies with descriptive outcomes (e.g., histopathological disease lesions), NAMs establish a dose–response bioactivity from human-cell-based in vitro studies. An in vitro-based understanding of the dose–response relationship may lead to further confirmatory testing. These iterative processes ultimately allow for quantitative risk assessments, such as the margin-of-safety calculation under various use scenarios [17].

**Figure 1:**
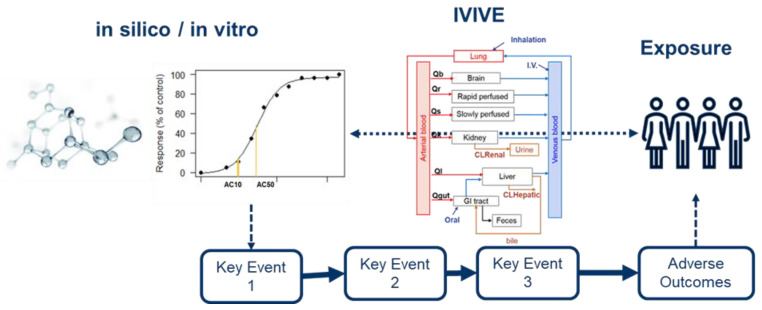
Schematic representation of NAM-based testing approach in risk assessment. Diverse evidence provided by NAMs (e.g., AOPs, IVIVE, and in vitro and in silico technologies) enables the integration of human exposure to kinetic and dynamics information needed for risk assessment without using in vivo animal data (Part of NAM-00 presentation, Supplementary Materials).

A NAMs-based evaluation approach is shown in a simplified diagram in Figure 1. Cellular events leading to clinical disease outcomes can be mapped using an adverse outcome pathway (AOP) framework. This connects an initial exposure event to a series of mechanistic and testable key events (KE) and ultimately to a clinically relevant disease outcome [18,19]. Links from the in vivo (human) exposure to a specific dose metric in a target tissue or a plasma level prediction can be approximated by biology-based kinetic dosimetry models. These models form the basis for in vitro to in vivo extrapolation (IVIVE). Figure 1 also illustrates different lines of in vitro and in silico-based evidence provided by NAMs, the overarching concept for the rest of the individual presentations at this symposium (Table 1).

## 2. NAM-01: U.S. Federal Efforts to Develop and Implement Alternatives to Animal Testing

An overview on NAMs opened the session and set the stage for specific considerations. The overview highlighted the work of National Toxicology Program Interagency Center for Evaluation of Alternative Toxicological Methods (NICEATM) and the U.S. Interagency Coordinating Committee on the Validation of Alternative Methods (ICCVAM), a group of 17 federal research and regulatory agencies. This congressionally mandated committee was created to establish guidelines and recommendations for regulators and stakeholders and promote regulatory acceptance of NAMs [20]. NICEATM and ICCVAM are engaged in a variety of projects to promote the application of NAMs at U.S. federal agencies in collaboration with industry, NGOs, academic stakeholders, and international counterparts.

### 2.1. Case Example: Alternatives to the Acute Toxicity “6-Pack” In Vivo Testing

The acute toxicity “6-pack” testing consists of in vivo assays for oral, dermal, and inhalation acute lethality; eye and skin irritation; and skin sensitization. They are required for pesticide registration [21,22,23] and are often used for safety testing of other chemicals. In 2018, the U.S. EPA issued a strategic plan to promote alternative test methods within the Toxic Substances Control Act (TSCA) program to use more human-relevant non-animal tests [2]. Instances of successful ICCVAM use of NAMs instead of additional in vivo testing were presented as case examples and are summarized below.

#### 2.1.1. Waiver for 6-Pack: Acute Lethality Tests

Acute toxicity tests identify the LC50 or LD50 (the concentration or dose at which 50% of the test animals die following a single exposure) that is used for safety protections in chemical handling. Currently, the U.S. EPA accepts a waiver for dermal lethality testing with an acute oral test, but there is no accepted non-animal alternative to the oral lethality test. To develop an NAM replacement for the acute oral lethality test, groups across the globe collaborated to develop a consensus computational model for predicting acute oral toxicity based on chemical structural features or chemical properties known as quantitative structure activity relationship (QSAR) models [6]. The resulting collaborative acute toxicity modeling suite (CATMoS) QSAR model is implemented in the free open-source OPEn structure–activity Relationship App (OPERA) platform [6,24,25]. NICEATM is collaborating with U.S. federal agency partners to determine how substituting CATMoS values for traditional animal study values would impact regulatory risk assessments.

Mixtures such as product formulations require additional toxicity information beyond individual components. The mixture additivity equation of the globally harmonized system (GHS), used for safety data sheets and labeling [26], estimates the toxicity of mixtures based on the toxicity of its individual components. A recent retrospective study compared the median lethal dose (LD50) derived from in vivo testing of individual ingredients with the mixture values obtained using the additivity equation. For less-toxic substances, the study found that within-class agreement was consistently over 85% regardless of the classification system used (EPA or GHS) [27]. This highlights an opportunity for using the existing component information to estimate requisite toxicity for mixture formulations without defaulting to more animal tests.

#### 2.1.2. Opportunities: Addressing the Variability of In Vivo Data When Developing NAMS

Existing animal data are often used as the standard for validating new approaches, but retrospective studies have highlighted the high level of variability and lack of reproducibility in some animal testing conducted for regulatory registration purposes. Recently published assessments indicated a lack of reproducibility for many in vivo study types that involve both acute and repeated exposures. Such variability is particularly prevalent for mild-to-moderate adverse effects, where the often-subjective scoring procedures are less than 50% likely to be reproduced [28,29]. When evaluations were pared down to a simple binary approach (i.e., toxic vs. non-toxic), the concordance was below 85% [28,29,30,31,32]. Thus, it is unrealistic to expect NAMs to achieve a higher concordance level than the intrinsic concordance exhibited by the in vivo test. Ongoing work for skin and eye irritation, skin sensitization, and acute inhalation now incorporates human biological relevance into NAM assessment rather than a direct comparison with in vivo toxicology study result [5,33,34].

#### 2.1.3. Case Example: Defined Approach (DA) for Skin Sensitization

**Figure 2:**
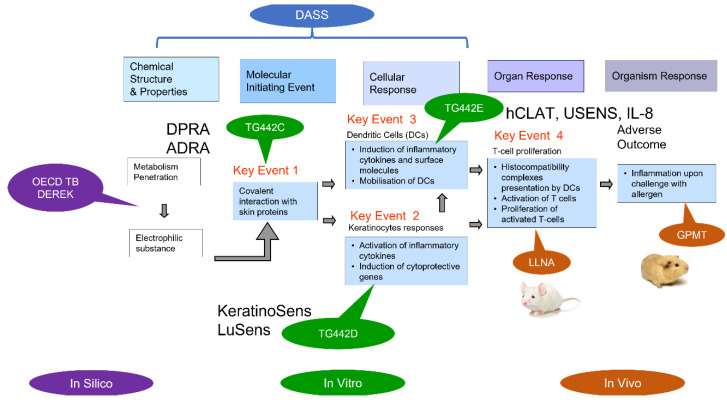
Overview of defined approach (DA) for skin sensitization. The DA for skin sensitization starts with in silico and in vitro approaches mapped to the adverse outcome pathway (AOP). (Part of NAM-01 presentation, Supplemental File).

Ideally, human data would be used to validate methods of assessing human health impacts, but robust clinical data are not commonly available for environmental chemicals, with skin sensitization being an exception. The recent guideline on defined approaches (DAs) for skin sensitization [35] issued by the Organisation for Economic Co-operation and Development (OECD) shows that an NAM testing strategy combining in vitro, in silico, and in chemico methods based on human mechanistic understanding outperforms animal tests when compared to the available human data. These NAM-based methods were also equal to or better for outcome reproducibility [36]. The success of DAs for skin sensitization is due to a detailed mechanistic understanding of the adverse outcome pathway (AOP) from molecular initiating events to key cellular events and then to tissue- or organism-level toxicity (Figure 2). As such, mechanistic understanding is the basis for advancing future inhalation NAM development [37].

DAs combine multiple test methods that map to different parts of the AOP that, when combined, provide enough biological coverage to predict the endpoint or adverse outcome of interest. This example highlights the potential for human biology-based AOPs to serve as a scaffold for mechanism-based (non-animal) risk assessment [38].

### 2.2. Tools for NAM-Based Computational Toxicological Assessment

NICEATM has a collection of public computational tools and datasets that can be used to support NAMs development and applications. The EPA National Center for Computational Toxicology (NCCT) created the Open (Quantitative) Structure–Activity/property Relationship Application (OPERA) as a free and open-source and open-data suite of QSAR models. OPERA provides access to QSAR models for physicochemical and metabolic parameters, environmental fate parameters, and toxicity endpoints including androgen and estrogen receptor binding models and the acute toxicity model mentioned above [6,39,40]. All OPERA models were built with curated data and QSAR-ready chemical structures and standardized using an open-source workflow [39]. The Integrated Chemical Environment (ICE: https://ice.ntp.niehs.nih.gov/, [41,42]) provides access to NICEATM and partner data along with tools supporting the use and evaluation of NAMs. ICE contains OPERA predictions for >800,000 chemicals and curated EPA ToxCast and NIEHS Tox21 high-throughput screening (cHTS) data. The cHTS data include integrated chemical and bioactivity quality-control information along with biological annotations to provide mechanistic target information to help users find relevant in vitro assays. The interactive ICE Curve Surfer tool [41] allows users to visualize the cHTS concentration response curve data and interact with the curves to compare bioactivity, potency, and efficacy ranges.

ICE also has biokinetic modeling tools allowing comparison and translation between in vitro concentration and in vivo doses. The physiologically based pharmacokinetic (PBPK) tool models a tissue-level concentration resulting from an external exposure, while the IVIVE tool uses cHTS data or user-provided in vitro data to calculate the external dose that would give a comparable plasma concentration based on the user-selected dosing regimens. The PBPK and IVIVE tools are based on the httk R package from the EPA [43]. In vivo dosimetry data, where available, can be added for comparison against the in-vitro-based predictions of in vivo exposure values. Additionally, ICE allows structure-based searching and characterization, including comparison of chemical lists based on molecular or physicochemical properties or based on consumer use categories, using the EPA’s Consumer Product use database. Another example of applying these data and the IVIVE tool to characterize health impacts of e-cigarettes has been published [44]. NAMs can be used to create context and provide insights on issues of reproducibility, throughput, and human relevance encountered with in vivo studies.

## 3. NAM-02: Applications of Biokinetic Modeling for In Vivo to In Vitro Extrapolation in Chemical Risk Assessment

Mathematical kinetic models that translate experimental data to in vivo systems are necessary to move towards non-animal NAM chemical risk assessments. PBPK models describing the adsorption, distribution, metabolism, and excretion (ADME) of a chemical within the body are already routinely used to relate an external exposure with an internal tissue concentration (called “forward” dosimetry modeling). Additional confounders, such as a chemical’s distribution and availability within the in vitro test system, must be characterized to relate the nominal concentration (amount added to the test system) to the bioactivity concentration. The kinetic models can then be used in “reverse” dosimetry modeling to relate the in vitro measurements to a comparable in vivo exposure scenario, referred to as IVIVE, an important part in using NAM data for chemical risk assessment.

**Figure 3:**
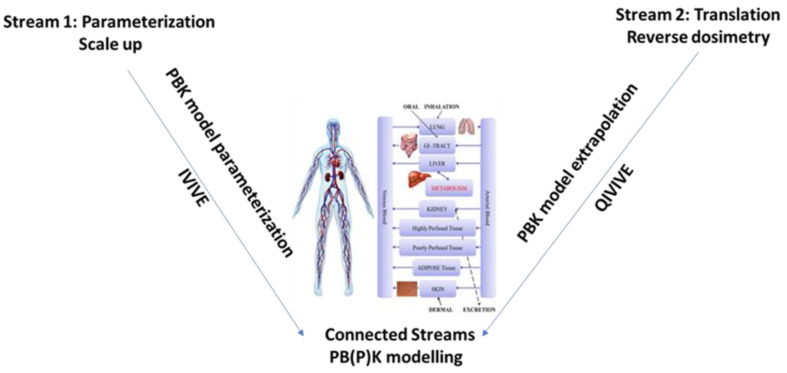
Components of IVIVE. Stream 1 (parameterization) scales up in vitro measures to parameterize the PBPK model. Stream 2 (translation) accounts for the chemical’s availability in vivo and in vitro. (Part of NAM-02 presentation, Supplemental File).

IVIVE can be considered part of two computational streams: application parameterization and translation (Figure 3). Parameterization involves scaling up in vitro measures by applying physiological correction factors to the data, such as using hepatocyte clearance rates to approximate in vivo liver clearance. This extrapolation of small-scale in vitro metabolism data is important for the models to account for in vivo metabolic and renal chemical clearance [45]. Translation focuses on reverse dosimetry, where the PBPK model is used to extrapolate the in vitro target response concentration in a specific tissue to an external exposure. In the translation stream, factors affecting the chemical’s availability in both in vitro (e.g., plastics binding, short exposures, air–liquid interface system, and single cells) and in vivo (e.g., gut absorption, clearance) systems should be accounted for [46,47]. By using the reverse dosimetry step for IVIVE, toxicologists can gain in vivo context for a variety of in vitro bioactivity assays. Examples of IVIVE linking toxicological endpoints of developmental toxicity, genotoxicity, neurotoxicity, and more recently endocrine disruptors are currently available [48,49,50,51,52,53,54,55,56,57,58,59,60,61].

Confidence in IVIVE is critical for wider applications and regulatory acceptance, which can be gained using standardized methods and broadly accepted guidelines. The recent OECD guidance for PBPK (the OECD document is titled “Guidance document on the characterisation, validation and reporting of Physiologically Based Kinetic (PBK) models for regulatory purposes”, part of the Series on Testing and Assessment with No. 331; regardless of the terminology used, to define the mathematical model PBK, PBPK, PBBK, and PBTK can all be considered synonyms) models document [62] was developed to:

- Provide recommendations and procedures for characterizing, reporting, and evaluating PBPK models intended for regulatory decision making;
- Address challenges to developing and evaluating PBPK models for chemicals without in vivo kinetic data;
- Promote use of PBPK models in regulatory risk assessment and facilitate dialogue between model developers and users.

This guidance focuses not only on defining the context and implementation of the model but also model validation, a critical step in gaining confidence in IVIVE. Characterizing in vivo and in vitro biokinetics using IVIVE is critical for adding biological context to in vitro results. Trust in these mathematical models can be achieved by providing goodness-of-fit and predictivity and to emphasize underlying uncertainties and assumptions for assessing their application.

## 4. NAM-03: Inhalation Exposure Modeling for Assessment of Aerosols and Vapors

Kinetic modeling for inhalation exposures to toxic chemicals, the primary route of exposure for inhalable tobacco products, has evolved over decades and is very mature. Modeling airway exposure is more complex than a standard oral or injection exposure. Models need to account for aerosols and vapors being delivered and deposited across the respiratory tract, with site-specific delivery and uptake into the tissues. Computational fluid dynamic (CFD) or computational fluid particle dynamic (CFPD)-based models can incorporate the site-specific 3D anatomy, physiology, and clearance processes with realistic breathing and exposure scenarios [63,64,65,66]. This allows for site-specific dosimetry calculations that can be correlated with tissue-specific bioactivity or toxicity.

CFD is a numerical method for describing how fluid flows over space and time within a three-dimensional system considering size, shape, and fluid properties. These methods are already used in industries such as aerospace and energy to aid design research and development. There have been significant advancements using CFD-based approaches to describe biological systems over the last 20 years, prompted by advancements in medical imaging systems (e.g., 3D/4D magnetic resonance imaging (MRI) and X-ray computed tomography (CT) imaging) and computational capabilities. Models across species and for different disease states are now possible, as are personalized models based on an individual’s specific anatomy and physiology [64,65,67].

Two cases of CFD application for tobacco-related toxicity assessment were presented. The first used CFD and PBPK to model localized cell- and tissue-specific internal doses of reactive aldehydes, allowing hazard ranking of tobacco smoke constituents [64]. The second case study focused on reducing and replacing sub chronic (90-day) animal inhalation studies for pesticide aerosols with realistic human exposure characterizations, in vitro toxicity studies with human cells, and kinetic modeling of aerosol dosimetry as a follow-on for short-term toxicity studies [5,68]. Here, CFD with muco-ciliary clearance modeling was used to calculate region-specific retained doses and risk assessment, enabling a waiver for a 90-day inhalation study of chlorothalonil required for the pesticide registration renewal. Below are additional details on these two cases.

### 4.1. Case Example: Multiscale CFD/PBPK Modeling for Aldehydes

Reactive aldehydes, such as formaldehyde, acetaldehyde, and acrolein, are industrial chemical intermediates and by-products of combustion, including tobacco smoke, as well as that naturally produced within the human body. Inhalation studies with these chemicals showed resultant cytotoxicity and carcinogenicity in localized sites within the rodent upper respiratory tract and served as the basis for many of the human health risk assessments by regulatory agencies. The site specificity of resulting lesions along with species differences in anatomy, respiratory physiology, and clearance were evaluated using CFD and PBPK modeling to better capture these species-specific differences in risk assessment [64]. The study used 3D-imaging of rat and human upper respiratory tracts to model airway anatomy and physiology across the different cell types and regions. Each surface facet of the CFD model had its own two-way coupled PBPK tissue model, which allowed for capturing the tissue- and species-specific ADME processes. Linking these site-specific PBPK models to the airway lumen CFD model via the mucus layer allowed for more realistic capture of inhalation exposure [64,65] (NAM-03, Slide-6, Supplemental File). Aldehydes are modeled to cross airway epithelial and subepithelial layers, where they can react with macromolecules, be metabolized by aldehyde dehydrogenases, or cleared into circulating blood. The local tissue dose is then simulated using realistic breathing profiles in rats. The resulting respiratory targeted site doses and lesions from animal studies were then used to predict the human tissue doses under realistic “yield in use” and measured smoking conditions.

**Figure 4:**
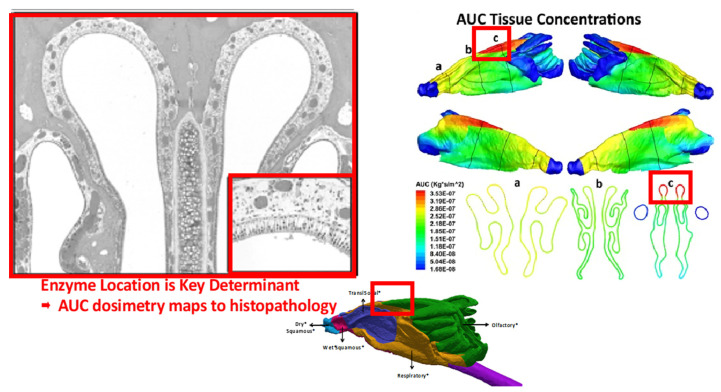
Histopathology section through anterior dorsal olfactory epithelium of the rat nasal airway corresponding to locations of maximum epithelial tissue concentrations of acetaldehyde (AUC) following 6 h/d, 5 d/wk inhalation exposures (from [64,69]; part of NAM-03 presentation, Supplemental File).

Comparisons between the two species were done using site-specific (“hot spot”) area under the curve (AUC) tissue concentrations for each airway region and aldehyde exposure condition. For the rat model, simulated exposure conditions used the NOAEL concentrations from published sub chronic in vivo studies, with the “hot spots” representing the target areas within each airway region that attained higher AUC concentrations of the aldehydes during inhalation exposure. The locations of respiratory tissue hotspots correlated with sites for local tissue damage observed in the prior sub chronic in vivo inhalation studies (Figure 4).

**Figure 5:**
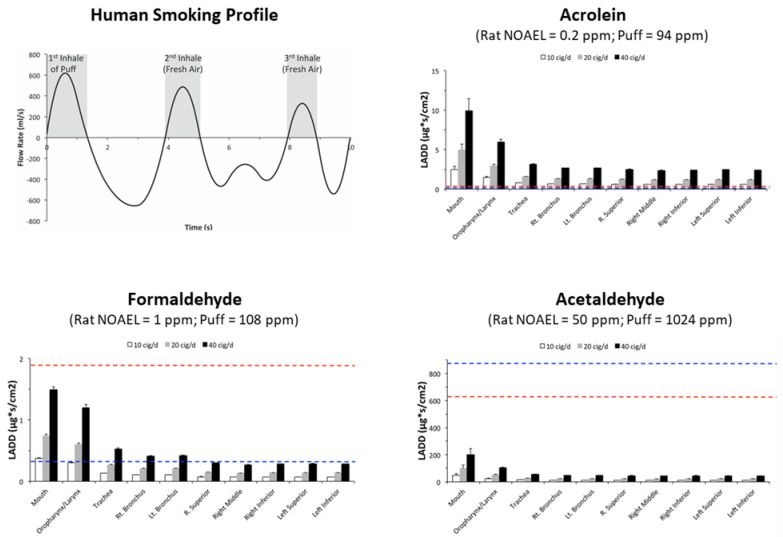
Comparisons of lifetime average daily doses (LADD) in rat respiratory (blue dashed lines) and olfactory (red dashed lines) nasal tissues determined by CFD/PBPK modeling at NOAEL’s from acrolein, formaldehyde, and acetaldehyde sub-chronic inhalation studies for each airway region of a human smoker, assuming 10, 20, or 40 cigarettes smoked per day (from [64]; part of NAM-03 presentation, Supplemental File).

This case study highlighted the benefit of CFD/PBPK modeling, linking the species-specific anatomy and spatial representations to toxicological outcomes. The human model simulated a measured breathing profile with an inhalation puff exposure followed by two breaths of fresh air for the aldehydes alone or in a mixture. Comparisons between the two rat and human models used “hot spots”, defined as the top 2.5% of surface element AUCs in each region, with adjustments in exposure to account for differences in the sub-chronic rat NOAEL AUC/breath vs. the human AUC/puff, puffs/cigarette, and cigarettes/day, to calculate the lifetime average daily doses (LADD) for each species. (NAM-03, Slide-9, Supplemental File) Since the upper respiratory lesions in the rat nose constituted the primary toxicity endpoints for risk assessments, the LADDs for rat olfactory and respiratory or transitional tissues were compared to the LADDs of human smoker tissues throughout the conducting airways for the aldehydes under different smoking levels (10, 20, and 40 cigarettes/day) (NAM-03, Slide-9, Supplemental File or Figure 5). The results of these simulations enabled a rank ordering of the aldehydes of concern (e.g., acrolein > formaldehyde > acetaldehyde) based on the presumed risk considering realistic exposure conditions and species differences in respiratory biology [64].

### 4.2. Case Example: Waiver of Sub Chronic 90-Day In Vivo Inhalation Study for Pesticide Reregistration

**Figure 6:**
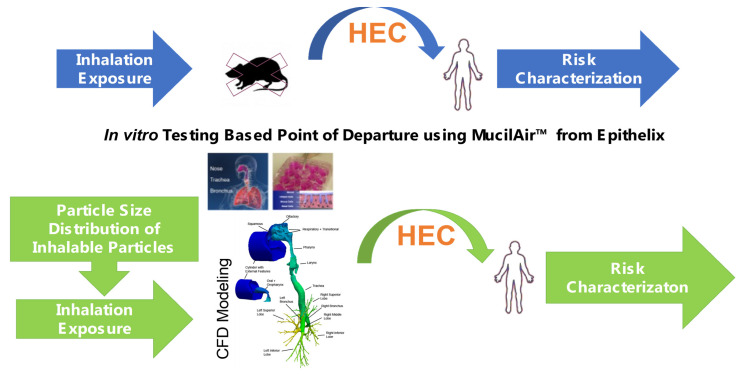
Replacement of default EPA pesticide registration requirement for a 90-day rat inhalation toxicity study for the pesticide chlorothalonil, with an NAM based upon a combination of in vitro toxicity testing with human respiratory cells grown at air–liquid interface (ALI), CFPD modeling for determining human equivalent exposure concentrations (HECs), and characterization of aerosol sizes and distributions under realistic human occupational exposure conditions (from [5,68]; part of NAM-03 presentation, Supplemental File).

Chlorothalonil has been available since 1966 and is a widely used fungicide labeled for use in more than 65 crops. It is known to be a contact irritant for all exposure routes and has extremely low volatility and water solubility. As part of the re-registration process for the Office of Pesticide Programs (OPP) under the U.S. Environmental Protection Agency (EPA), acute and short-term (two-week) range-finding inhalation studies were first done. These studies demonstrated that the expected site-specific cytotoxicity in the upper conducting airways of rats were either largely resolved or reduced in a two-week recovery period. Traditionally, these toxicity findings would have triggered the requirement of a follow-up 90-day inhalation study. With the U.S. EPA’s focus on a reduction in animal testing, data from in vitro studies with human respiratory cells coupled with enhanced characterization of the exposure and target doses were submitted in support of risk assessments as shown in Figure 6 [5,70]. For the in vitro cytotoxicity testing, the MucilAir™ system (Epithelix SA, Geneva, Switzerland) was used to derive an in vitro BMD for cytotoxicity as a point of departure (POD) for use in CFD-PBPK modeling. As in the previous multiscale CFD/PBPK modeling case study, the distribution of aerosols was predicted based on particle size and distribution. Species-specific breathing patterns to predict localized deposited dose across the respiratory tract and enabling identification of local target tissue deposition were confirmed using rat histopathological data. Respiratory deposition in humans, modeled under a variety of breathing conditions to account for different ventilation patterns for applicators and bystanders, were evaluated. The discrete deposited aerosol mass per local airway surface area were determined for both species. The comparisons showed clear species differences in target site deposited masses, depending upon aerosol size distribution and breathing mode (nasal vs. oral). In addition, CFD model outputs for humans were used as inputs to a mucociliary clearance model to obtain the retained doses in each airway region that could be compared with results from in vitro studies with human respiratory epithelial cells. From the combined CFD/clearance model analyses, the retained dose AUC measures were compared with benchmark doses from the in vitro studies and for calculating HECs for various human exposure scenarios. Using the human in vitro data and model allowed for the reduction in uncertainty factors, and the resulting risk assessments were accepted as sufficient to waive the 90-day rat inhalation study requirements. This was one of the clearest case studies wherein kinetic models integrated with in vitro data to support the reduction in animal testing and improved characterization of risk.

## 5. NAM-04: Assessing Respiratory Toxicity of Chemicals in Two Human Bronchial In Vitro Systems

The availability and development of in vitro methodologies for inhalation toxicity testing were described. The human respiratory tract is composed of more than 40 cell types, and the pulmonary region contains 100–150 m^2^ of surface area with a thin air–blood barrier, making it an excellent surface for gas exchange and an important portal of entry. Inhalation toxicity testing is typically conducted using rodents despite the anatomical and physiological differences compared to the human respiratory system that require adjustment, such as the dosimetry models described previously. Species differences in breathing patterns (nose vs. mouth breathing), the airway architecture including branching patterns, and cell distribution and mucous composition can impact how aerosols are delivered and deposited along the respiratory tract. In addition to dosimetry differences, biological responses in cellular targets could vary, and the use of human cell-based testing system is more representative of the human-specific effects in response to inhaled substances.

The IN vitro System to Predict REspiratory toxicity (INSPiRE) Initiative was born out of a recommendation from a 2016 workshop, “Alternative Approaches for Acute Inhalation Toxicity Testing to Address Global Regulatory and Non-Regulatory Data Requirements” [71]. The workshop developed a decision-tree testing strategy that considered physicochemical properties and dosimetry to inform future conduct and deployment of proof-of-concept testing. The INSPiRE Initiative is aimed at the latter. The INSPiRE Initiative’s goals are to present a case study on how in vitro approaches may be used for inhalation toxicity testing and thereby strengthen scientific confidence in in vitro models for risk assessment. The project assesses the use of a cell line grown as monoculture and human reconstructed lung tissues (MucilAir™) for assessing the toxicity of example chemicals and to potentially rank them based on toxicity. Although this project focuses on a few substances only, the outcomes may still inform future testing of mixtures and formulations.

**Figure 7:**
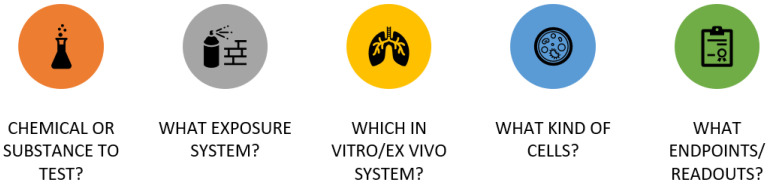
Questions influencing the INSPiRE Initiative study design (Part of NAM-04 presentation, Supplemental File).

A series of key questions (Figure 7) relevant for designing any inhalation study were considered for the initiative and discussed in detail below.

- **What chemical or substance?** The chemical or substance to test will dictate an appropriate study design. The physicochemical properties determine how the substance is inhaled and which region of the respiratory tract will be most affected—in silico aerosol models (e.g., CFD models as discussed in NAM 03) can be helpful for identification. Whether the substance is locally metabolized will further influence the choice of cells to use. If these properties are unknown, a structurally similar compound (i.e., a read-across approach) may give some insight.
- **What in vitro exposure system?** When selecting the in vitro exposure systems, considerations to balance include the ease of conducting the experiment with physiological relevance in inhalation exposures. Pipetting and air–liquid interface (ALI) exposures are the two most common exposure routes to a test chemical for an in vitro system. While aerosol or gas exposure using an ALI exposure system would require specialized equipment and training, it may be more human-relevant than pipetting. No matter what exposure system is used, the robustness of the in vitro test method should be assessed to identify and account for any potential variability [72].
- **Which in vitro/ex vivo test system?** Various test systems exist ranging from relatively simple monocultures to more complex organotypic micro-physiological systems and precision-cut lung slices. The choice of test system depends on the goals of the study. Table 2 highlights some advantages and disadvantages of representative test systems.
- **What kinds of cells?** The human respiratory tract is composed of more than 40 cell types. The cells used should be from the region(s) of the respiratory tract that is most affected by the test chemical (in silico model predictions should help with the identification of the area). However, while there are a wide range of cells from the proximal respiratory tract available (cell lines and primary cells), the choice for distal respiratory tract cells is currently more limited.
- **What endpoints/readouts?** Assay endpoint and readouts selections will depend on the properties of the chemical as well as the goal of the study. Using AOPs can be helpful in linking assay readouts to the steps (key events) along the pathway and identifying adverse outcomes of interest.

### Case Example: Silanes in 2D Monoculture and 3D Human Tissue Models

For the INSPiRE Initiative, workshop experts chose silanes as the first chemical category to test. Silanes are used as reducing and coupling agents. Since silanes are highly reactive and hydrolyze quickly, and occupational exposure is most likely to be silane vapor, using conventional cell culture systems (with medium on top of the cells) is not possible. Therefore, an ALI exposure module (VITROCELL^®^ 6/4, Waldkirch, Germany), connected to a system to generate vapor mixed with dry air or nitrogen, was selected for the INSPiRE Initiative. It was decided to test the simplest cell system that allows for ALI exposure, a bronchial cell line (BEAS-2B) grown as a monoculture on cell inserts, as well as a reconstructed human lung tissue model (MucilAir™, Epithelix SA, Geneva, Switzerland) from bronchial cells of single donors (four different donors used). While a formal AOP is not available for silanes toxicity, data from existing animal studies showed pulmonary edema and hemorrhage, indicating loss of the epithelial barrier from the uptake and hydrolysis of silane (Triethoxysilane Dossier, ECHA, 2022). The concentration of silane in the cells and the basal media were measured to estimate the delivered dose, cell health (cytotoxicity, viability), and sub-toxicity effects (inflammatory cytokines) for the 3D model, MucilAir™ tissues. Trans-epithelial electrical resistance (TEER) (to estimate the barrier integrity), cilia beat frequency, and histology were performed as well.

The initial trials provided several insights. For example, because the silanes hydrolyze quickly (in a matter of seconds), dried air had to be used during exposure. The BEAS-2B monoculture was highly sensitive to prolonged exposure to dry air, impacting baseline viability. Therefore, adjustments (i.e., reduced exposure time) to the exposure conditions were needed to avoid artifacts. These preliminary findings are encouraging in the use of non-animal testing for health risk assessment. Despite many recent developments allowing for more human-relevant in vitro testing, there is no “one size fits all” approach and the choice of NAMs will depend on the scientific question. Early engagement with stakeholders and consultations with regulators to discuss proposed NAM strategy are useful in shaping experimental strategies. In vitro methods may need adaptation depending on the test substance, and it is likely that a battery of assays is needed for adequate coverage for risk assessment of all potential inhalation toxicity endpoints. In combination with in silico models, in vitro testing can provide human-relevant mechanistic insights, beyond simply replacing animal testing, which is also highlighted in the pesticide reregistration case study (see NAM-03).

## 6. NAM-05: In Silico Toxicology as a New Approach Method in Tobacco Regulatory Science

Computational models and in silico science are integral to everyday activities, such as weather predictions. Regulatory agencies such as the FDA also use in silico modeling approaches to identify the possible human health impacts of a chemical in hazard identification and characterization. There are many computational toxicology tools offering competitive rapidity and cost effectiveness advantages over experimental in vivo and in vitro testing. These approaches can be used to screen and guide additional testing as part of an overall toxicological assessment; however, it is critical to ensure they are performed and communicated appropriately for the intended use.

**Figure 8:**
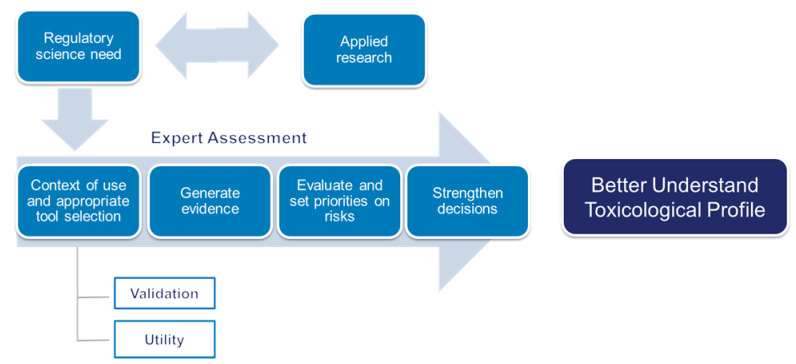
The role of computational toxicology in tobacco regulatory science (Part of NAM-05 presentation, Supplemental File).

When applied to tobacco regulatory science, the FDA has used in silico toxicology to better understand the toxicological profile of tobacco ingredients and products [73,74]. A conceptual overview is given in Figure 8. While these in silico approaches are highly automated and rapid, their implementation requires subject matter expert (SME) oversight. The SME will review the suitability of the methods and interpretation of the findings in the context of overall toxicological assessment. Therefore, outputs require review and guidance from those trained in their use, and the methods and procedures employed need to be validated in the context of use.

In silico approaches can be used for versatile and rapid screening, offering advantages over traditional toxicology tools. Use of software to generate visualizations of similar structures and scaffolds allows toxicologists to identify structural alerts and support read across techniques. Using molecular descriptors to calculate the similarity between compounds provides a science-based, quantitative method to evaluating read across compounds. Mechanistic information can also be evaluated; for example, identification of Michael acceptors, capable of forming covalent bonds to nucleophilic sites of protein and DNA, can screen for potential adverse outcomes such as mutagenicity.

As illustrations, there are several applied research projects funded by the FDA’s Center for Tobacco Products (CTP) that assess various in silico tools to aid in understanding how these approaches work for evaluating various types of tobacco-related chemicals, ingredients, and products including e-fluids. In one study, 900 tobacco constituents not found in the training data were used to assess the performance of QSAR models in predicting mutagenicity [75]. These models generally performed comparable to the human expert structure–activity relationship (SAR) model, suggesting that a single QSAR system could screen hazard with confidence in mutagenicity endpoint. Assessment of the molecular coverage was an important aspect of this research, ensuring that a diversity of structures of interest were covered by the QSAR model. The utility of using multiple technologies to identify the genotoxicity mode of action was evaluated using both in silico QSAR models and an in vitro multiflow assay for DNA damage [73]. Hazard predictions and prioritizations were performed for 150 flavors used in e-vapor products and demonstrated a high concordance between in silico and in vitro systems for the clastogenicity predictions. These in silico studies highlight the advantages of using these techniques as part of tobacco research, showing pragmatic utility in supporting the hazard characterization of tobacco products.

## 7. NAM-06: Applications of Mechanistic Data in Risk Assessment: Exposure Alignment and Evidence Integration

**Figure 9:**
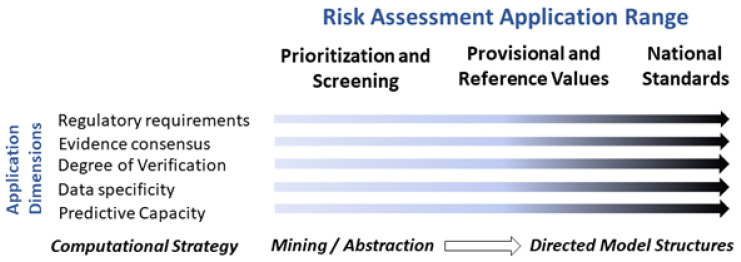
Risk assessment landscape. Adapted from the U.S. EPA Human Health Risk Assessment Strategic Research Action Plan for 2016–2019 [76] for illustration purpose only (Part of NAM-06 presentation, Supplemental File).

Mechanism-based NAM approaches can be used to modernize risk assessment approaches and address various regulatory needs. Figure 9 depicts the large landscape of environmental risk assessment applications required by the U.S. EPA. These range from screening and prioritization to setting National Ambient Air Quality Standards (NAAQS) for ubiquitous exposure to inhalation pollutants such as particulate matter and ozone. The regulatory requirements, scientific evidence, predictive capacity, and degree of verification all increase significantly from left to right in the schematic, and the type of computational methods also significantly shifts as well. For screening and prioritization, data mining and abstraction may suffice, while a deeper understanding using directed model structures, such as that described in NAM-02 and NAM-03, and more enhanced validation are needed to support national standards. In considering the level of confidence needed for such assessment applications, a mechanistic approach can aid in constructing a more coherent context for decisions across this landscape.

The critical challenge inherent in any risk assessment is evidence integration, and several important transitions are currently modernizing assessment approaches. First is the transition to an increasing use of systematic review approaches and criteria for data quality and relevance in support of evidence integration. Systematic review is used to obtain and evaluate data and literature for hazard identification according to population, exposure, comparator, and outcome (PECO) statements that reflect a problem formulation related to a given assessment goal. Typically, evaluation of human health (e.g., epidemiological, occupational or clinical studies), laboratory animal, and mechanistic data streams is performed in a parallel and hierarchical fashion (NAM-06, Slide 4, Supplemental File). However, mechanistic data, such as from NAMs, may best serve as a scaffold to provide context and aid coherence across data streams for evidence integration [38]. The second important transition in risk assessment is the pivot to comprehensive source-to-outcome characterization to tailor and refine risk assessments [38,77]. Source-to-outcome characterization was advanced by and composed of critical and integrated components. The first component is the characterization of exposure events, which can be described by an aggregate exposure pathway (AEP) [78]. The AEP is used for exposure characterization to organize data and describes key transitions from emission sources to media-specific exposure routes, resulting in an interface with the receptor (e.g., humans or ecological species) in an analogous conceptual framework such as that of the AOP. The second component is the definition and description of the target site exposure (TSE), which serves as the critical link between the AEP and the AOP frameworks (NAM-06, Slide 5, Supplemental File). The TSE is best characterized using multi-scale dosimetry models, such as described in NAM-02 and NAM-03 above. Such dosimetry models facilitate integration of the determinants of the physicochemical properties of the inhaled agent and ADME. Importantly, depending on the level of mechanistic understanding, the TSE can be described at either the molecular initiating event (MIE) or various key events (KE) involved in the pathogenesis to an adverse outcome. The last component in the source to outcome continuum is the AOP, as previously described, that connects the MIE and various KE to characterize pathogenesis as a process resulting in adverse outcomes. The multi-scale capability of dosimetry models, coupled with the biological understanding underlying the relevant mechanisms represented by the AOP, allow the TSE to be tailored to the level of observation (e.g., molecular, cellular, tissue, population) of the adverse effect being measured and used for dose–response analysis in a risk assessment.

The third transition in risk assessment methods is the use of NAMs. The U.S. EPA drafted a strategic plan in 2018 to use NAMs to refine, reduce, or replace animal testing under the TSCA [1,2]. The strategy to develop and implement NAMs in decision making under TSCA [2,79] has the following goals: (1) identify, develop, and integrate NAMs into existing assessment approaches; (2) build confidence in their utility and reliability; and (3) implement NAMs in decision making through collaboration, training, and education. The EPA subsequently also released a work plan for the use of NAMs, first in 2020 and then updated in 2021, that describes agency efforts to reduce vertebrate animal testing using NAMs [79,80]. The objectives of the work plan are as follows: evaluate the regulatory flexibility for accommodating NAMs; develop baselines and metrics for assessing progress; establish scientific confidence in NAMs and demonstrate application to regulatory decisions; develop NAMs to address scientific challenges and fill important data gaps; and engage and communicate with stakeholders. Participation in the CORESTA conference is an example of the last objective and is needed to advance understanding and foster collaboration.

Translation across different experimental platforms is also a critical aspect of advancing risk assessment methods. Key events of existing AOP help to create the biological contexts needed to target NAM development and advance the understanding of how these assays relate to adverse outcomes, including bridging acute assays to chronic outcome measures [37]. Dosimetry models have been used for extrapolation across species and are now considered critical to aid IVIVE. For inhalation exposures, these models integrate critical physicochemical characteristics of gases or of aerosols (e.g., particle diameter, size distribution, and density) with other anatomical determinants, such as species-specific airway architecture and physiological determinants of dosimetry such as breathing mode, ventilation rate, cellular composition, and tissue capacities for ADME. Different dosimetry models are deployed depending on the physicochemical properties of the inhaled agent, e.g., the Multiple-path Particle Dosimetry (MPPD) model for particles or CFD models for reactive gases [64,81,82], and can range from default algorithms for dosimetric adjustments to more sophisticated structures [83]. The NASEM in its report entitled “Using 21st Century Science to Improve Risk-related Evaluations” [84] noted the need to deploy varied dosimetry models to account for mechanisms dictating dosimetry differences across various experimental platforms (e.g., cell-free systems, cell culture systems, ALI, organ-on-a-chip) and in vivo or human exposure to achieve exposure alignment. Exposure alignment ensures that inferences across the platforms can be compared on the same basis: an “apples to apples” comparison. Some ADME characteristics and physicochemical properties are similar across platforms, but others are very specific to the exposure system, such as binding to media or culture plates and protein binding and solubility. Thus, incorporating computational dosimetry models to the PBPK models described earlier can capture the uptake and distribution of a chemical within a system and is critical to exposure alignment.

Mechanistic modeling further improves this translation and evolves empirical modeling from asking “what” to pursuing a quantitative characterization of “how” and “why” the adverse outcomes occur. Such mechanistic models incorporate quantitative test measures of the KE, translating the TSE across different test systems to improve data integration and refine inferences. Dosimetry models help characterize the TSE by accounting for key physicochemical properties and integrating them with anatomical and physiological determinants. Translation of the TSE and the human equivalent concentration (HEC) requires proper scaling up. Scale up of the TSE accounts for both the physicochemical properties and the ADME determinants of the test system, be it animal or in vitro test system. For example, the dose for in vitro test systems should account for the dimension of the system, flow rate or media reactions, and surface area of the well, as these can dictate the flux to the cells or determine the internal tissue concentration. These measurements can then be extrapolated using models to an HEC. Typically, the dose metric is commensurate with the level of observation. For example, for particles, it is the deposited or retained mass normalized to the surface area of the respiratory region effected [81]. Proper reporting of exposure system parameters and tissue features is requisite for translation to be accurate and support repurposing for evidence integration in risk assessment.

Finally, mechanistic models advance understanding and characterization from a qualitative AOP merely describing the sequence of KE to a quantitative AOP as needed for quantitative risk assessment. The KE relationships that drive the transitions from one key event to the next are needed [85,86]. For instance, it is not as important to ascertain that a chemical can cause cytotoxicity as it is to determine the degree of cytotoxicity within the tissue that could lead to the next KE of regenerative cell proliferation. This quantitative description is necessary for determining various dose–response relationships and aiding evidence integration for risk characterization. As NAM approaches gain further acceptance and confidence, they can provide mechanistic insights and aid this translation and integration. Eventually, this will support a transition of NAMs themselves from their use only in early prioritization and hazard identification to their use in quantitative AOPs with kinetic modeling, ultimately supporting toxicological risk assessment [37,84,87].

### 7.1. Case Example: Assessing New Chemical Substances for TSCA Using an AOP-Inspired IATA

Section 5 of TSCA does not require upfront testing for new chemicals but relies on existing data to make a preliminary safety assessment. While various methods are used to assess chemical risks using limited data, such as the read across approaches using analogs described earlier (NAM-05), NAMs are now also being considered to help construct chemical categories that help inform such preliminary assessments. Case examples using an integrated approach to testing and assessment (IATA) based on dosimetry modeling and AOP-inspired NAMs for two categories, namely inhaled general surfactants and poorly soluble low toxicity (PSLT) polymers, have been developed via a collaboration between the EPA Office of Research and Development with the Office of Chemical Safety and Pollution Prevention overseeing TSCA implementation. These IATA to define the two chemical categories are in the process of being published. 

**Figure 10:**
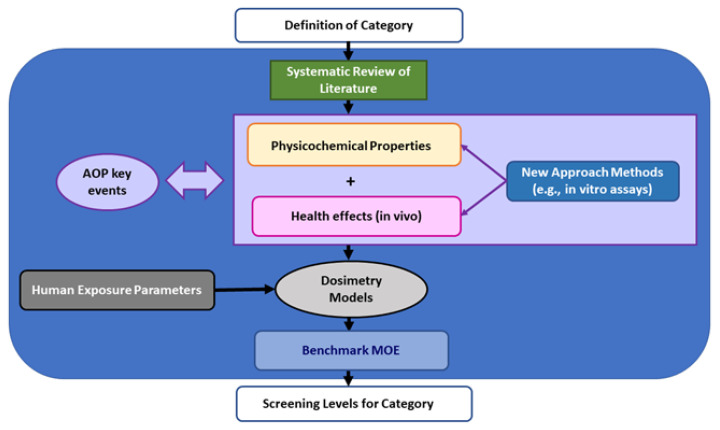
General schematic of an integrated approach to testing and assessment (IATA) for constructing chemical categories used to screen new chemical substances under TSCA. AOP, adverse outcome pathway; MOE, margin of exposure (Part of NAM-06 presentation, Supplemental File).

A generalized schematic of these IATA is given in Figure 10, highlighting the key steps. To start, a systematic literature review of available data defining the category is conducted for characteristic physicochemical properties as well as any health-effect data. NAMs, including the QSAR models mentioned earlier, may be used to characterize the physicochemical properties that define the category or may provide mechanistic data describing various KE in the AOPs selected as relevant. Dosimetry models are used for exposure alignment and translation of data across experimental platforms. NAMs may also help inform those models. For example, a NAM characterizing in vitro solubility for a given particle of interest may help inform clearance rates in dosimetry models such as the MPPD model. Dosimetry models can be customized to specific human exposure scenarios with targeted parameters, e.g., actual aerosol size distribution and density for a specific operation and corresponding ventilation rate. Dosimetry models are used to calculate the HEC values for derivation of the benchmark dose for margin of exposure (MOE) risk assessment. The benchmark MOE values establish the screening levels for the category.

In applying these examples, a generalized AOP-inspired IATA in six steps can be articulated (NAM-06, Slide-21, Supplemental File). The first step is the identification of problem formulation, target exposure, and the inhaled agent. The second step is the evaluation of available information and obtaining physiochemical properties needed to conduct the modeling. The third step consists of the prediction of internal dose and location of exposures based on the ADME properties and the AOP. The fourth step includes evaluation of what is known about the AOP. This will help choose a test system that enables capture of information relevant for the expected impacts. The fifth step involves a battery of (in vitro/in silico) assays, not a single study, for testing the KE in the AOP. The sixth and last step involves exposure alignment of the results to human relevant exposures using IVIVE and integration with the overall health risk assessment.

### 7.2. Additional Considerations in the Use of NAMs

Reporting standards can serve as a road map in establishing best practices for the use of NAMs in assessment and support adherence to findable, accessible, interoperable, and reusable (FAIR) data principles [88] now required of all National Institutes of Health grantees and aimed at increasing data transparency. Some specific reporting standards needed for both exposure systems and choice of cell types were provided (NAM-06, Slides 22–24, Supplemental File). When using testing tools, including NAMs, in risk assessment, it is important to not only identify outcomes but also discern how and why they are relevant to the specific contexts of health risk or decisions. For instance, translating NAMs results to the TSE require sufficient quantitative details to identify key parameters needed to carry out dosimetry modeling. Information on the test systems used and assays conditions are critical given the highly customizable nature of these exposure systems. Additionally, it is crucial to articulate the rationale for the choice of tissue used given its bearing on the relationship to the adverse outcome of interest or KEs being measured in the assay. The quality and performance metrics of an assay for a given test material should be documented and reported.

Another consideration for advancing NAMs is characterization of the translation factors used to relate the findings back to the target in vivo (human) context. Traditional uncertainty factors such as interspecies variations, duration, and severity may still be relevant for the non-animal approaches. Additionally, novel translations may be required to account for target tissue specificity and metabolic competency of the test system. Providing this additional detail fosters dialogue around the values and assumptions going into AOP-based IATA. The information supports inferences made and allows for data and model integration. Transparent and detailed reporting using standardized procedures and formats can make it easier to contextualize interpretation and repurpose models for different chemicals or exposures.

## 8. Panel Discussion and Closing Remarks

When the presentations concluded, symposium participants joined the organizers and speakers via live (virtual) discussion sessions. Participants expressed a great deal of common interest in future opportunities for promoting the development and use of NAMs in tobacco science and facilitating on-going dialog among stakeholders, researchers, and regulatory agencies. While there was overwhelming support towards the intent to use NAMs in principle, all participants acknowledged that the path for using NAMs is still at infancy, in part reflecting the limited confidence and uncertainty in utilizing NAMs beyond hazard identification and screening. A widely shared sentiment was the need for identifying and standardizing the criteria for establishing confidence [16] in rapidly introduced NAM tools as well as challenges with a wider application in risk assessment, particularly for use in regulatory decision making. As noted in NAM-06, reporting standards can serve as a road map to establishing best practices for the wider use of NAMs in risk assessment. Adoption of data-sharing standards, such as Minimum Information About a Microarray Experiment (MIAME) [89] and SEND for nonclinical data reporting in a consistent format (https://www.cdisc.org/standards/foundational/send, accessed on 1 March 2022), help consolidate findings and publication norms in the relevant fields. In addition, recent reporting guidelines for PBPK models [62,90] provide further transparency when using new methods. Leveraging these approaches and concepts can allow for greater re-use of data and contextualization as well as boosting confidence of stakeholders and regulators. Clear communication around the use of NAMs continues to be a focus area, and dedicated engagement and communication opportunities among regulatory agencies, NAM developers, and industry stakeholders are critical to sustain the momentum [15].

The symposium ended with the concluding remarks that NAMs, including in silico (computational) modeling along with in vitro testing, have the potential to not only replace but also possibly outperform traditional animal testing for evaluating human health impacts. Given the successful case examples, NAM approaches are pragmatic in terms of cost, time, and resources and offer enhanced sensitivity in predicting human-relevant health impacts, for example, AOP-based IATAs. At the same time, opportunities exist to gain confidence in the realms of context of use and standardization. Clarity on the degree of qualification is required before NAMs-based risk assessments achieve full legitimacy for regulatory decision making.

In summary,

- Expanded use of NAMs in toxicological assessment applications requires a shift in paradigm from screening and hazard identification to hazard characterization and, ultimately, quantitative risk assessments for regulatory applications and with that a shift from the apical in vivo endpoints to mechanistic NAM-based endpoints. This means changing the question, for example, from testing that seeks an “in vivo no effect level” to one generating a “POD for a mechanistic cellular event that leads to adverse outcomes”.
- In vitro testing needs to be designed considering a variety of factors including properties of the test substance, test system, and the desired endpoints under the intended use. There is likely more than one set of NAM assay data to answer toxicological questions typically addressed by in vivo testing. Computational kinetic models allow for extrapolation of dosimetry data across test systems to provide human relevance and simulate different exposure scenarios for qualitative and quantitative exposure and risk assessment.
- IATAs provide a structure to combine mechanistic information and data from different NAMs in a weight of evidence-based toxicological assessment.
- To fully recognize the potential of NAMs for risk assessment, criteria for establishing confidence in fit for purpose, reliable, and relevant NAMs are necessary. For example, in vitro inhalation testing is not yet standardized for longer-term exposures. Addressing these deficiencies will help new methods’ scientific confidence and traction.
- For tobacco products, including novel smoke-free products, there are increasing human data available from volunteers from clinical trials or consumers in addition to historical epidemiological data from cigarettes. This availability of human data is unique and can help substantially in gaining scientific confidence in an application of NAMs for PRR products and for enhancing tobacco regulatory sciences.

## Figures and Tables

**Table 1:** Symposium agenda *.

Speaker	Title
NAM-00: K Monica Lee, Altria; S Bell, ILS	Advancing New Alternative Methods for Tobacco Harm Reduction: Introduction
NAM-01: Nicole Kleinstreuer, U.S. NIEHS	U.S. Federal Efforts to Develop and Implement Alternatives to Animal Testing
NAM-02: Alicia Paini and Andrew Worth, EC JRC ^1^	Application of Biokinetic Modeling for IVIVE in Chemical Risk Assessment
NAM-03: Richard Corley, GCTC LLC	Inhalation Exposure Modeling for Assessing Health Risks of Toxic Aerosols and Vapors
NAM-04: Andreas O. Stucki, PETA Science Consortium International	Assessing Respiratory Toxicity of Chemicals in Two Human Bronchial In Vitro Systems
NAM-05: Luis Valerio Jr., U.S. FDA/CTP	In Silico Toxicology as a New Approach Methodology in Tobacco Regulatory Science
NAM-06: Annie Jarabek, U.S. EPA	Application of Mechanistic Data in Risk Assessment: Exposure Alignment and Evidence Integration

^1^ Affiliation: Alicia Paini changed to esqLABS GmbH, Saterland, Germany. * A copy of the presentations (including NAM Symposium program and the bibliographies) is provided in the Supplementary Files.

**Table 2:** Advantages and disadvantages of certain inhalation test systems. (Part of NAM-04 presentation, Supplemental file).

Cell System	Advantages	Disadvantages
Monoculture	Simple and least expensive High throughput	Inherent limitations of cells used (e.g., cell lines) Often only short-term ALI cultures possible
Reconstructed tissues	In-situ-like epithelium ALI cultures possible for months Obtainable from healthy or diseased donors	Low-medium throughput
Micro-physiological systems	Relevant microenvironment Mechanical stimuli possible (stretch or flow) Allow combinations of different tissues (e.g., lung–liver)	Materials used may interfere with chemical Standardization and comparability difficult Low-medium throughput
Precision cut lung slices	Obtainable from healthy or diseased donors All relevant cells and structures present Cryopreservation possible (circumvents the lack of donor tissues)	Cross-section (non-physiological exposure of interstitium possible) Multiple cell types may make readout challenging Donor tissues not readily available

## Supplementary Materials

The following supporting information can be downloaded at: https://www.mdpi.com/article/10.3390/toxics10120760/s1, File S1. PDF of all presentations submitted as a single file.

## Abbreviations

ADME	Absorptions, distribution, metabolism, and elimination
AEP	Aggregate exposure pathway
AOP	Adverse outcome pathway
BMD	Benchmark dose
CFD	Computational fluid dynamics
CFPD	Computational fluid particle dynamics
CORESTA	Cooperation Centre for Scientific Research Relative to Tobacco
DA	Defined approach
ENDS	Electronic nicotine delivery system
GRAS	Generally recognized as safe
HEC	Human equivalent concentration
IATA	Integrated approaches to testing and assessment
IVIVE	In vitro to in vivo extrapolation
KE	Key event
LADD	Lifetime average daily dose
LD/LC50	Lethal dose/concentration at 50%
NAMs	New approach methodologies
NGO	Non-governmental organization
NOAEL	No observable adverse effect level
PBPK	Physiologically based pharmacokinetic
PSLT	Poorly soluble low toxicity
QRA	Quantitative risk assessment
SME	Subject matter expert
SSPT	Smoke-Science and Product Technology
TSE	Target site exposure
TSCA	Toxic Substances Control Act
