# Supplementary material for: Advancing New Approach Methodologies (NAMs) for Tobacco Harm Reduction: Synopsis from the 2021 CORESTA SSPT—NAMs Symposium
Source: Toxics. 2022 Dec 6;10(12):760. doi: 10.3390/toxics10120760 (PMC9781465; doi:10.3390/toxics10120760)
Supplement: Supplementary file 1 [file toxics-10-00760-s001.zip › toxics-1979748-supplementary-File S1.pdf]

Advancing New Alternative Methods (NAMs) for Tobacco Harm Reduction

INTRODUCTION

K Monica Lee, PhD DABT
Altria Client Services, LLC, kyeonghee.m.lee@altria.com

Shannon Bell, PhD
Integrated Laboratory Systems, sbell@ils-inc.com

October 19, 2021

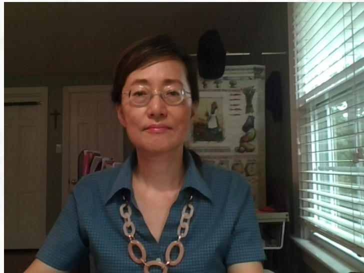

2021 NAM Symposium: Agenda

Topics and Rosters

1. Nicole Kleinstreuer, US NIEHS	US Federal Efforts to Develop and Implement Alternatives to Animal Testing
2. Alicia Paini, EU JRC	Application of Biokinetic Modeling for IVIVE in Chemical Risk Assessment
3. Rick Corley, GCTC LLC	Inhalation Exposure Modeling for Assessing Health Risks of Toxic Aerosols and Vapors
**10-Min Break**	
4. Andy O Stucki, PETA	Assessing Respiratory Toxicity of Chemicals in Two Human Bronchial in vitro Systems
5. Luis Valerio Jr., US FDA/CTP	In Silico Toxicology as a New Approach Methodology in Tobacco Regulatory Science
6. Annie Jarabek, US EPA	Application of Mechanistic Data in Risk Assessment: Exposure Alignment and Evidence Integration
**20-Min Discussion**	

DISCLAIMER: The presentations reflect personal opinions of speakers and do not represent the views of their affiliated organizations.

Introduction TOC

- Background
  - Setting the stage
  - New Alternative Methods…for Toxicology
  - Key Concepts
- Why
  - Tobacco Harm Reduction
  - Non-Combustible Alternatives

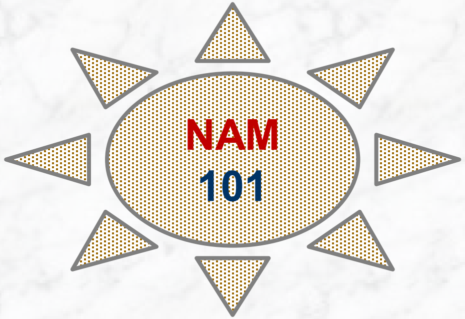

! Key Points !

SYMPOSIUM

Advancing New Alternative Methods (NAMs) for Tobacco Harm Reduction

What is a NAM?

- NAMs ≈ New Alternative (or Approach) Methods

  "A New Alternative Method (NAM) is ***any*** *technology, methodology, approach, or combination thereof* that can be used to provide information on *chemical* ***hazard and risk*** *assessment* that ***avoids the use of intact animals***" (EPA, 2018)

  - https://www.epa.gov/assessing-and-managing-chemicals-under-tsca/alternative-test-methods-and-strategies-reduce

- Other related terms ≈ "Alternative to animal testing"
  - (*https://en.wikipedia.org/wiki/Alternatives_to_animal_testing*)

- NAMs are more than avoiding in vivo animal studies
  - NAMs are not seeking a 1-to-1 replacement
  - NAMs pursue a better way we do toxicology

NAM is the 21st Century Toxicology

- NAMs are to modernize toxicology

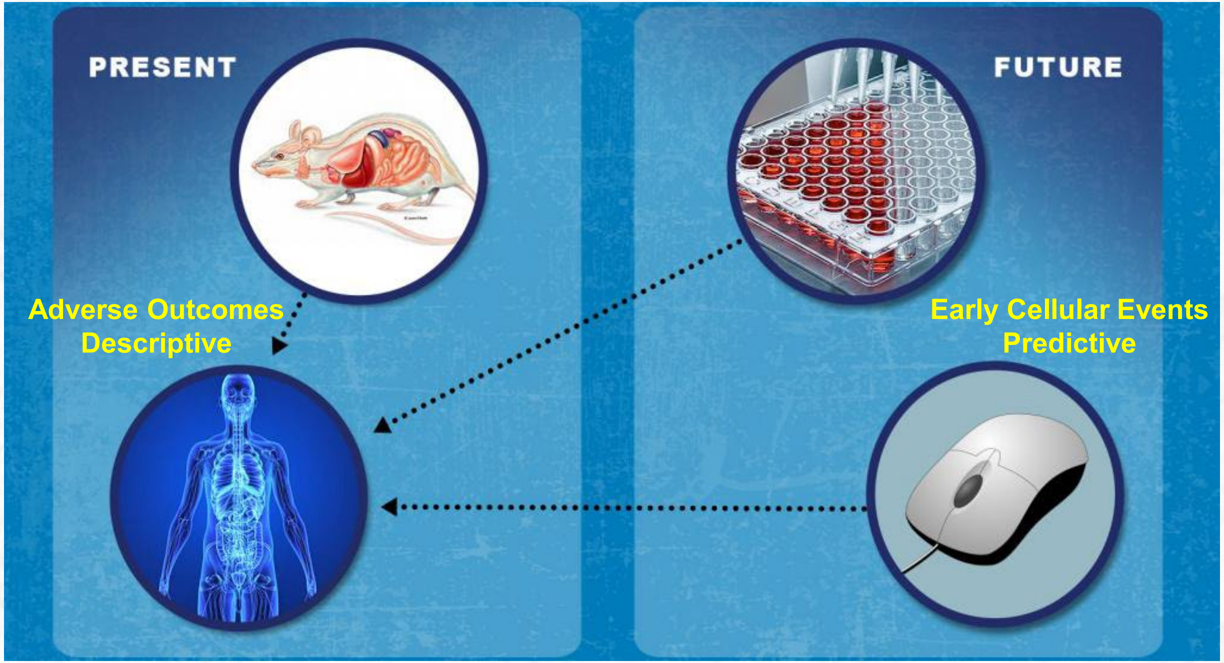

https://www.fda.gov/science-research/about-science-research-fda/advancing-alternative-methods-FDA

NAMs can…

- ✓ Be clinically relevant - human cell-based in vitro assays
- ✓ Be Predictive - connecting based on Mode-of-Action & early events
- ✓ Leverage in silico - structure-based chemical evaluation; computational tools
- → **Drive the 3Rs (Reduce/Refine/ Replace) animal-based testing**

Opportunities…

- ! Awareness vs. Application
- ! Supporting vs. Replacing what in vivo
- ! Uncertainty & Context of use

There are successful case examples

Our World - Tobacco Harm Reduction (THR)

Interest & Need for Non-Combustible Alternatives

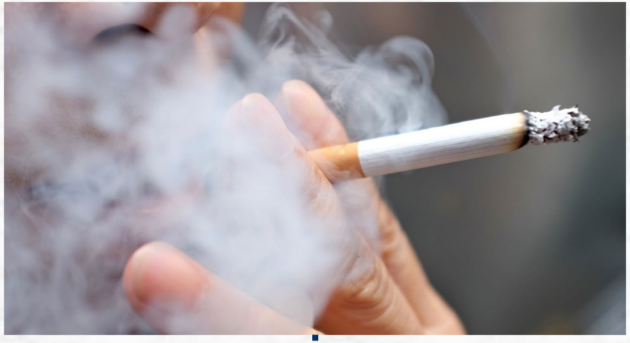

Inhalable

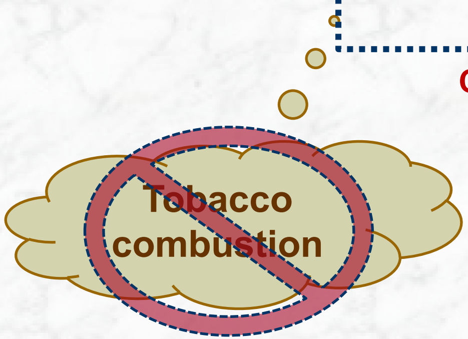

Complete Switching

Oral

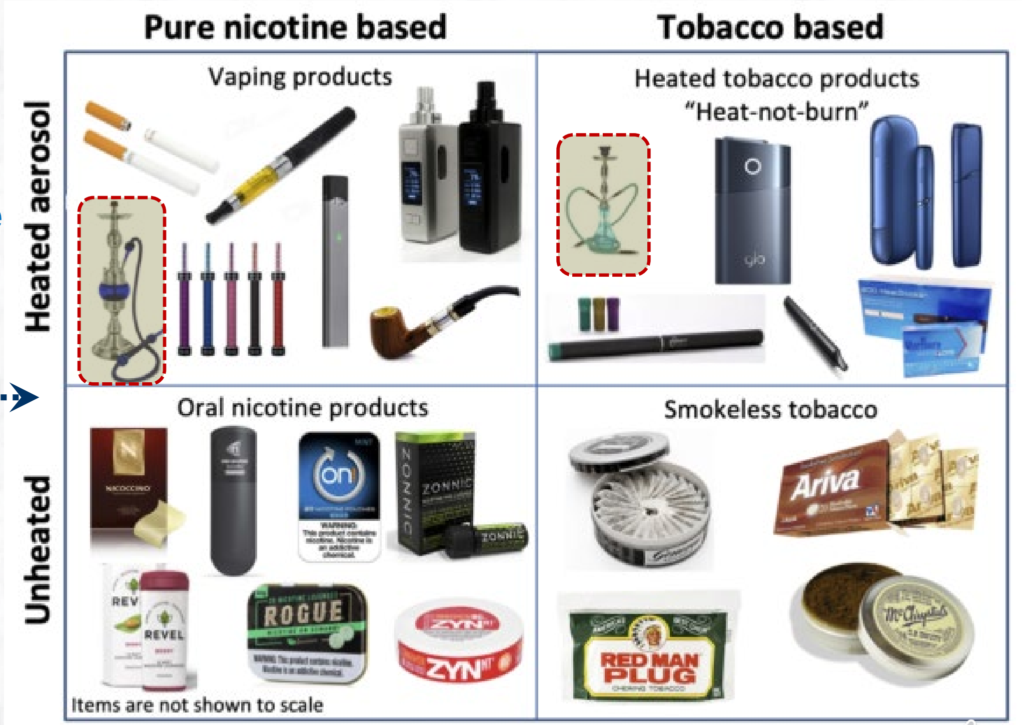

https://www.clivebates.com/vaping-tobacco-harm-reduction-nicotine-science-and-policy-q-a/

Potential Reduced-Risk Products (RRPs) – Examples (shaded, not considered RRPs)

Evaluating Health Risk of RRPs

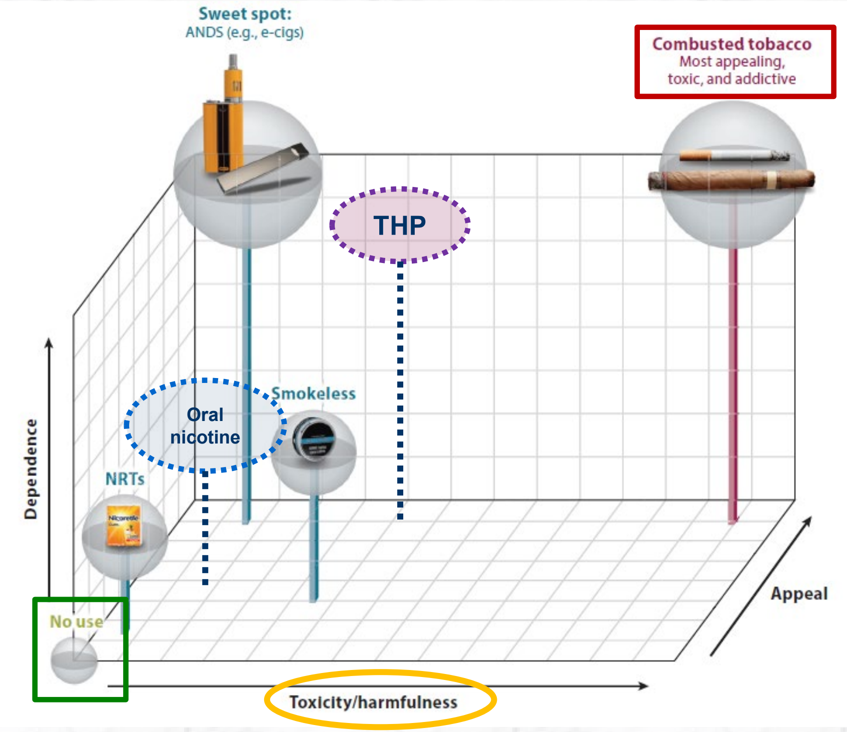

Abrams et al. 2018 https://pubmed.ncbi.nlm.nih.gov/29323611/ (marked for illustration)

WoE Toxicological Assessment

Ingredient - QRA
- GRAS, literature & In silico

Product – Analytical / QRA
- HPHCs, byproducts

Biological – in vitro
- Standard & Mechanistic

?

Biological – in vivo*
- (*if needed) NOEL & in vitro hazard

Should we change the way we pose questions?

WoE= Weight-of-Evidence; GRAS=Generally regarded as safe; HPHCs=Harmful and potentially harmful chemicals; QRA=Quantitative risk assessment; NOEL=No-observable-effect-level

New Alternative Methods (NAMs)

offer a different way of "connecting the dots" for tox assessment

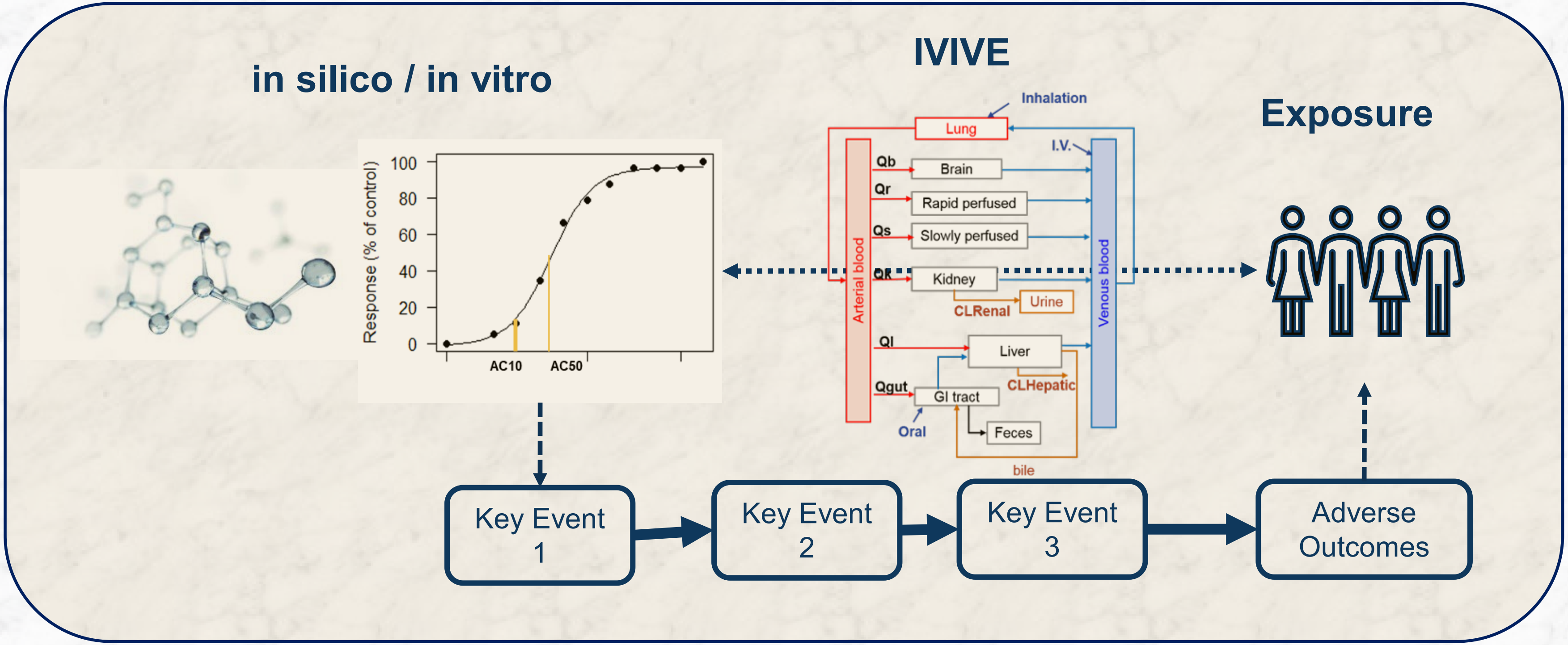

IVIVE ((in vitro to in vivo extrapolation) – Quantitative relationship, using kinetic modeling, between in vitro bioactivity and the in vivo exposure expected to result in adverse outcomes

Last Comments

- **This symposium will provide:**
  - A high-level overview – new terminologies, some are technical
  - Introducing publicly available NAM tools and resources
  - Case examples with success stories, on-going efforts
- **Participants are encouraged to:**
  - Contrast to what we currently do & context of use
  - Share thoughts on potential barriers and limitations
  - Consider common areas of NAMs for collective opportunity
- ***Comment & ask questions!***
  - *Don't forget the Panel Discussion at the end*

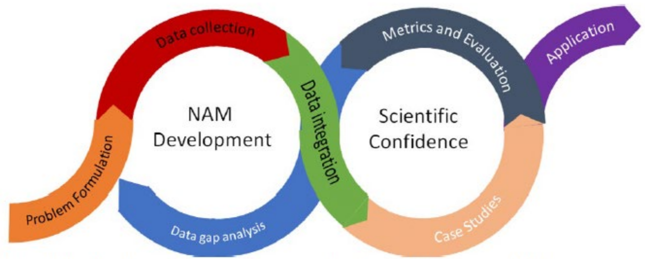

Figure 2. Problem-focused research planning and implementation process at EPA.

THANK YOU

- CORESTA Scientific Commission
  - Next Generation Tox (NGT) Task Force
  - In Vitro Tox (IVT) Subgroup
  - Biomarker (BMK) Subgroup
- Invited Speakers
- Altria Client Services, LLC
- Integrated Laboratory Systems
- Participants

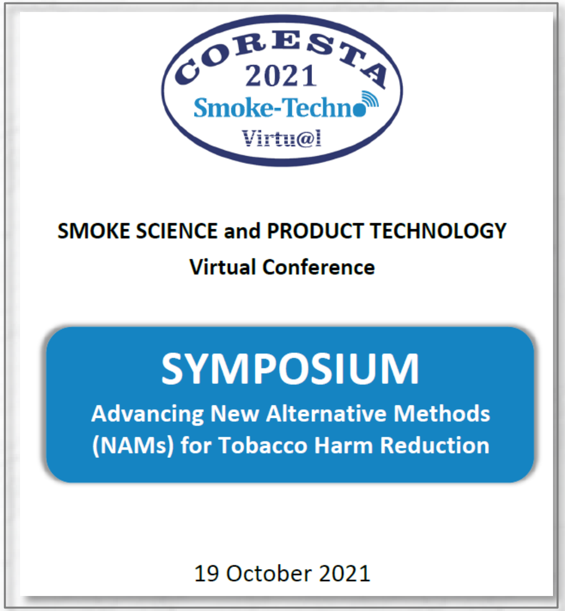
CORESTA 2021 Smoke-Techno Virtu@l

SMOKE SCIENCE and PRODUCT TECHNOLOGY
Virtual Conference

SYMPOSIUM
Advancing New Alternative Methods (NAMs) for Tobacco Harm Reduction

19 October 2021

Selected References

- ICCVAM https://ntp.niehs.nih.gov/whatwestudy/niceatm/iccvam/index.html
- Tox21 https://tox21.gov/overview/
- Abrams et al. 2018 https://pubmed.ncbi.nlm.nih.gov/29323611/
- Avila et al. 2020 https://pubmed.ncbi.nlm.nih.gov/32325112/
- Parish et al. 2020 https://pubmed.ncbi.nlm.nih.gov/32017962/
- The Counterfactual 2020 https://www.clivebates.com/vaping-tobacco-harm-reduction-nicotine-science-and-policy-q-a/
- OECD TG331 2021 https://www.oecd.org/officialdocuments/publicdisplaydocumentpdf/?cote=ENV-CBC-MONO(2021)1%20&doclanguage=en
- New Approach Methods Work Plan, EPA 2020 https://www.epa.gov/sites/default/files/2020-06/documents/epa_nam_work_plan.pdf

NTP
National Toxicology Program

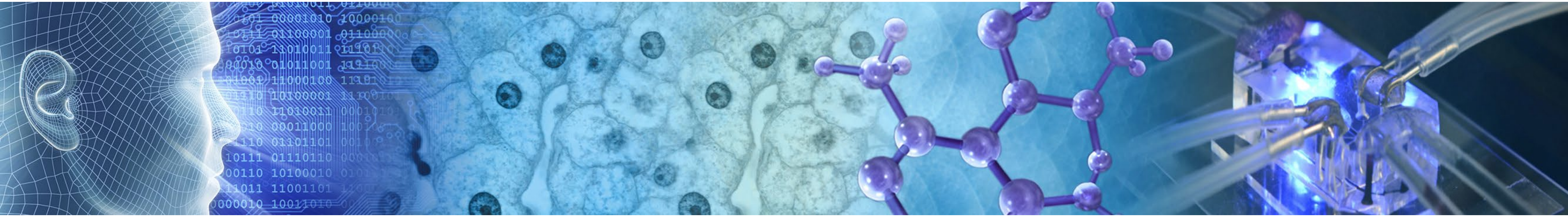

US federal efforts to develop and implement alternatives to animal testing

CORESTA NAMs Symposium, 2021
19 October, 2021

Nicole Kleinstreuer
Acting NICEATM Director

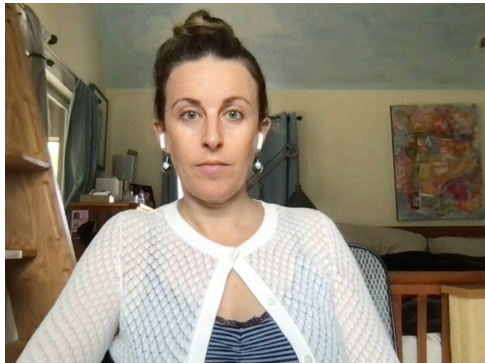

NICEATM and ICCVAM

- National Toxicology Program Interagency Center for the Evaluation of Alternative Toxicological Methods (**NICEATM**), supporting the Interagency Coordinating Committee for the Validation of Alternative Methods (**ICCVAM**)
- ICCVAM Authorization Act of 2000: To establish, wherever feasible, guidelines, recommendations, and regulations that promote the regulatory acceptance of new and revised toxicological tests that protect human and animal health and the environment while reducing, refining, or replacing (**3Rs**) animal tests and ensuring human safety and product effectiveness.

10 Research Agencies

Agency for Toxic Substances and Disease Registry
National Institute for Occupational Safety and Health
National Cancer Institute
National Institute of Environmental Health Sciences
National Library of Medicine
National Institutes of Health
Department of Defense
Department of Energy
National Institute of Standards and Technology
Veterans Affairs Office of Research and Development

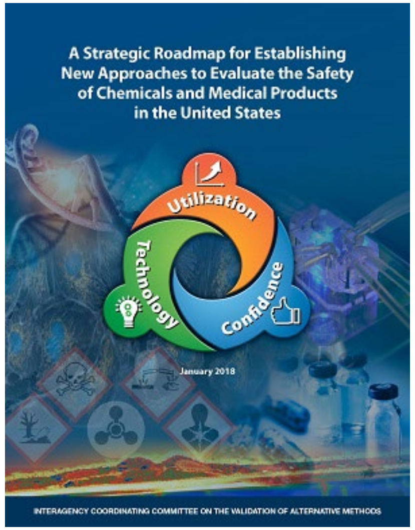

7 Regulatory Agencies

Consumer Product Safety Commission
Department of Agriculture
Department of the Interior
Department of Transportation
Environmental Protection Agency
Food and Drug Administration
Occupational Safety and Health Administration

*Other participants include: NCATS, Tox21 Repres[illegible]atives

More information: https://ntp.niehs.nih.gov/go/iccvam

Ongoing NICEATM and ICCVAM Projects

- Integrated Chemical Environment
- OPERA (QSAR/QSPR)
- Quantitative IVIVE
- Reference data curation
- Variability of in vivo data
- Acute Systemic Toxicity
- Dermal absorption
- Eye and skin irritation
- Skin sensitization
- ZF models (SEAZIT)
- Acute Fish Retrospective
- Carcinogenesis
- Cardiovascular toxicity
- Developmental Toxicity
- Animal-free affinity reagents
- Microphysiological Systems
- Evolving Process of Validation

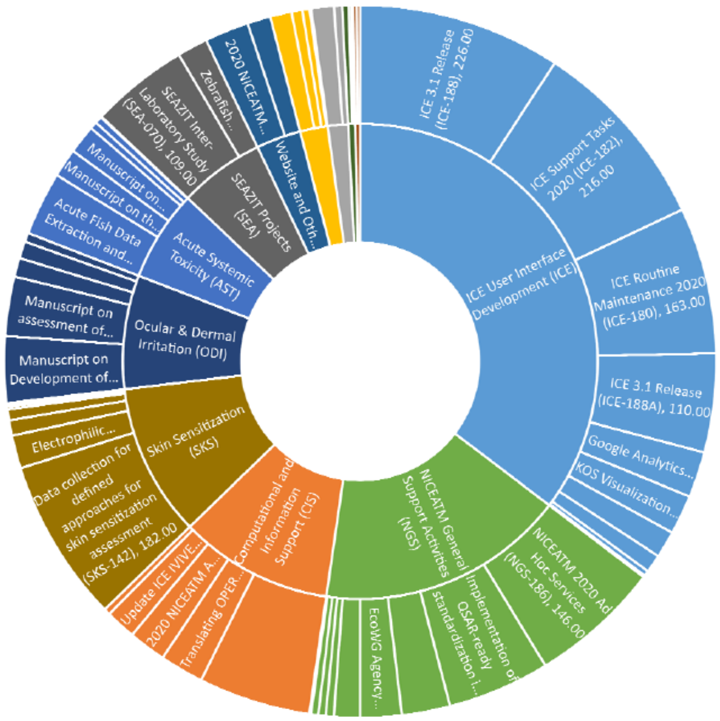

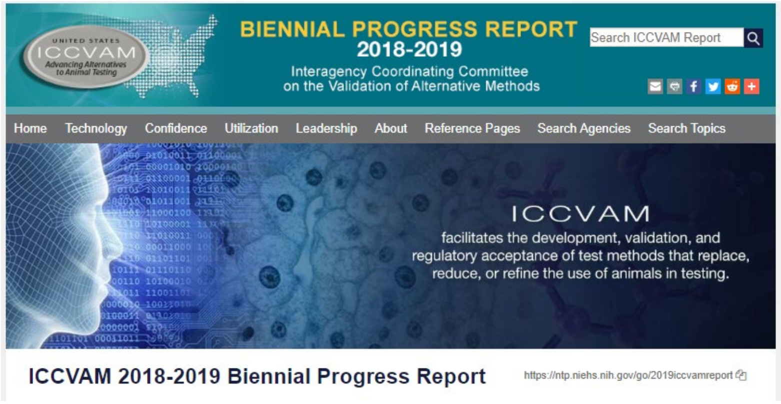

- Summarizes US agency activities to promote alternatives or reduce animal use
  - Contributions from every ICCVAM member agency
- 2018-2019 report published in July 2020, available online at: https://ntp.niehs.nih.gov/go/2019iccvamreport

- **Subscribe to NICEATMNews email list**

  https://ntp.niehs.nih.gov/go/niceatm

Acute 6-Pack Alternatives

Dermal lethality	• US EPA Waiver guidance available
Oral lethality	• In silico (CATMoS) for single chemicals; additivity for formulations under consideration
Inhalation lethality	• 3D models being evaluated; LC50 database for in silico model development being built
Eye irritation	• NAMs for Cat I and/or Cat IV (TG 437, 438, 460, 491, 492, 494); Prospective testing ongoing
Skin irritation	• NAMs for Cat I or Cat IV (TG 430, 431, 435, 439); Prospective testing ongoing
Skin sensitization	• EPA science policy, draft risk assessment, and OECD international DASS guideline

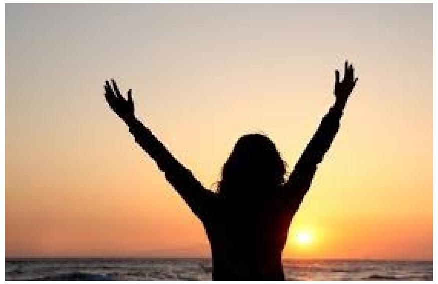

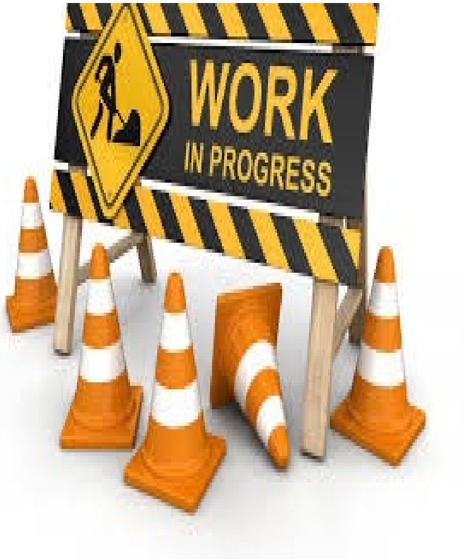

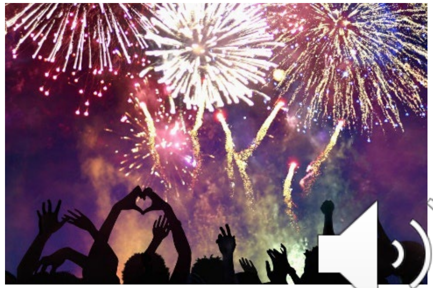

Mansouri et al. 2021 EHP; Clippinger et al. 2021 Cut Ocu Tox; Rooney et al. 2021 Reg Tox Pharm; Allen et al. 2021 ALTEX; Hamm et al. 2021 Reg Tox Pharm

Collaborative Modeling Project for Predicting Acute Oral Toxicity (CATMoS)

Mansouri et al. 2021 EHP

CATMoS implementation in OPERA

OPERA suite of models:

- Free, open-source, and open-data
- Command line and GUI
- Single chemical and batch mode
- Windows OS and Linux
- Embeddable wrapper libraries in Java, C, C++, and Python

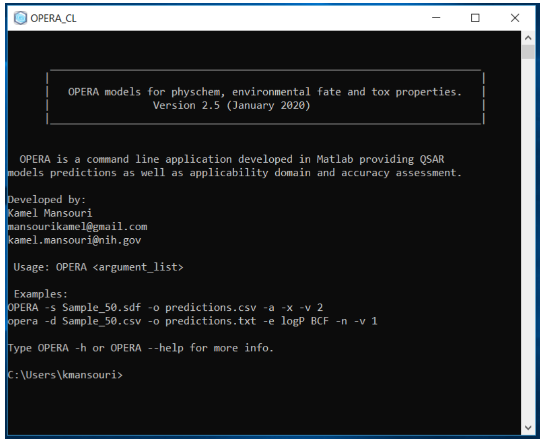

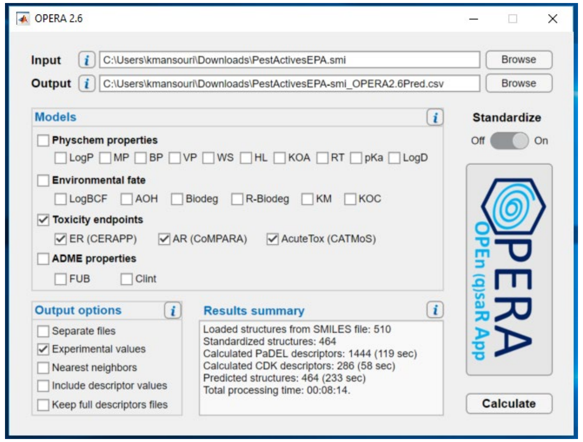

Collaboration with ATWG partners and ICCVAM agencies

Agency	No. Substances	Agency	No. Substances
Air Force	421	EPA OPP	36
Army Public Health Command	18	EPA OPPT	8
Army Edgewood Chemical Biological Center	42	EPA NCCT	4815
CPSC	110	EPA EFED	160
DOT	3671	FDA CFSAN	22

Progress made with EPA EFED

- Compare CATMoS predictions to acute oral toxicity data on 160 pesticides registered in the last 25 years.
- Determine impact on risk assessments, leading to additional curation and characterizing confidence in predictions.

Mansouri et al. 2021 EHP

Acute Toxicity Mixtures Equation Analyses

- GHS Mixtures Equation - mathematical approach to calculating toxicity of mixtures based on components
- Compare LD50s predicted for formulations based on the GHS Mixtures Equation to those from in vivo results with the complete formulation.
- Data set consisted of 671 formulations produced by eight companies:
  - 51 antimicrobial cleaning products (AMCPs), 620 agrochemical formulations
- Analysis based on PPE requirements demonstrated 82% concordance overall.

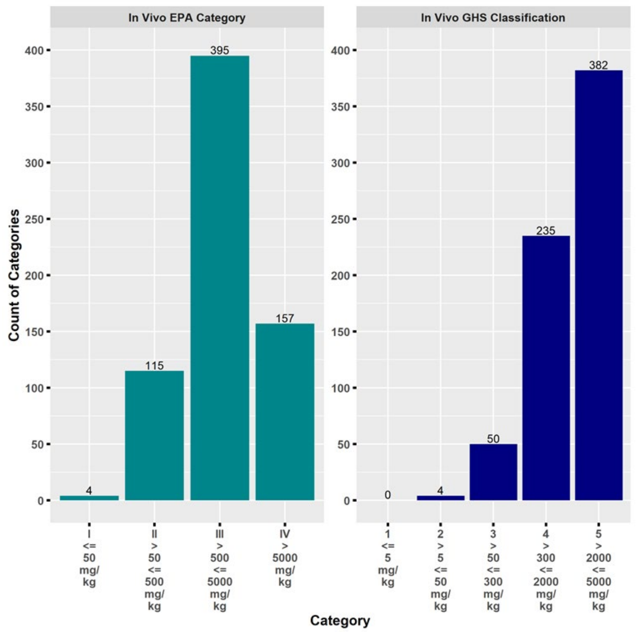

Within-class concordance for less toxic substances was consistently over 85% regardless of classification system (EPA, GHS).

In vivo LD_{50}	Additivity LD_{50} Prediction (mg/kg)			Within-class Concordance
	≤50	>50 to ≤500	>500	
**≤50**	3	1	0	75%
**>50 to ≤500**	4	30	81	26%
**>500**	1	35	514	93%
**Total**	8	66	595	82%

Hamm et al. 2021 Reg Tox Pharm

Human-relevant approaches for eye corrosion/irritation potential

Clippinger et al. 2021 Cut Ocu Tox

Prior GHS category	1	2A	2B	NC
1 (serious eye damage)	73%	16%	0%	10%
2A (irritant)	4%	33%	4%	59%
2B (mild irritant)	0%	4%	16%	80%
NC (non-irritant)	1%	4%	2%	94%

Adapted from Luechtefeld et al., ALTEX 33(2), 2016.

- The rabbit test should not be used as a reference method to demonstrate the validity of *in vitro/ex vivo* assays
- *In vitro/ex vivo* methods are as or more reliable and relevant than the rabbit test

Consider strengths and limitations of all available methods with respect to:

- their relevance to human ocular anatomy
- the mechanisms of eye irritation/corrosion in humans

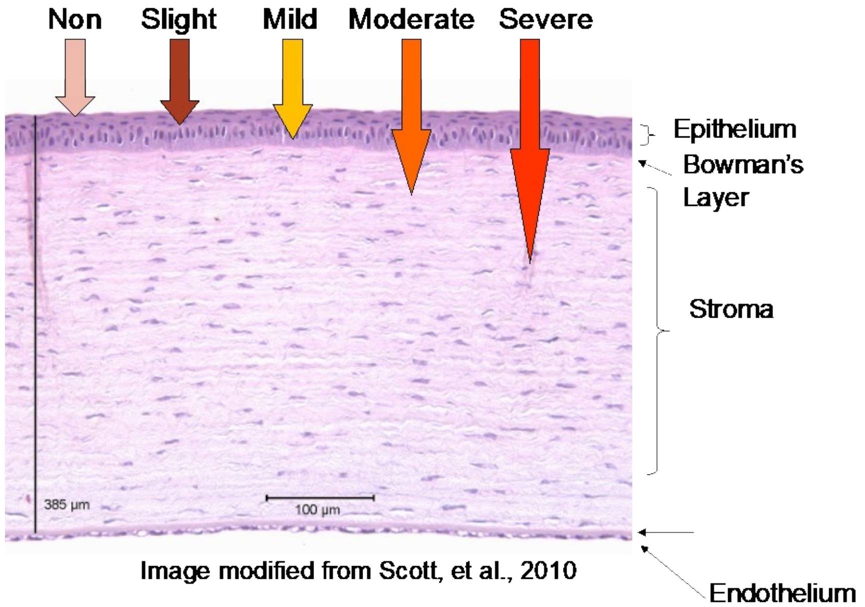

Image modified from Scott, et al., 2010

Skin Irritation Variability

EPA	Category I	Category II	Category III	Category IV
PDII	Corrosive	>5.0	2.1-5.0	0-2.0
Signal Word	DANGER	WARNING	CAUTION	CAUTION
PPE Required	Coveralls worn over long-sleeved shirt and long pants	Coveralls worn over short-sleeved shirt and short pants	Long-sleeved shirt and long pants	Long-sleeved shirt and long pants
	socks	socks	socks	socks
	Chemical-resistant footwear	Chemical-resistant footwear	Shoes	Shoes
	Waterproof or chemical resistant gloves	Waterproof or chemical resistant gloves	Waterproof or chemical resistant gloves	No minimum
	Irritant		Non-irritant	

Curated Dataset with Binary Approach

Prior Result	Irritant (Cat I or II)	Non-irritant (Cat III or IV)
Irritant (Cat I or II)	75.6%	24.4%
Non-irritant (Cat III or IV)	3.9%	96.1%

Curated Dataset

Prior result	COR	II	III	IV
COR	86.3%	4.2%	7.1%	2.5%
II	14.1%	44.9%	20.5%	20.5%
III	6.9%	5.2%	53.6%	34.3%
IV	0.9%	2.0%	9.1%	88.0%

Rooney et al. 2021 Reg Tox Pharm

Inhalation Tox Variability: LC50 Ranges Over EPA Categories

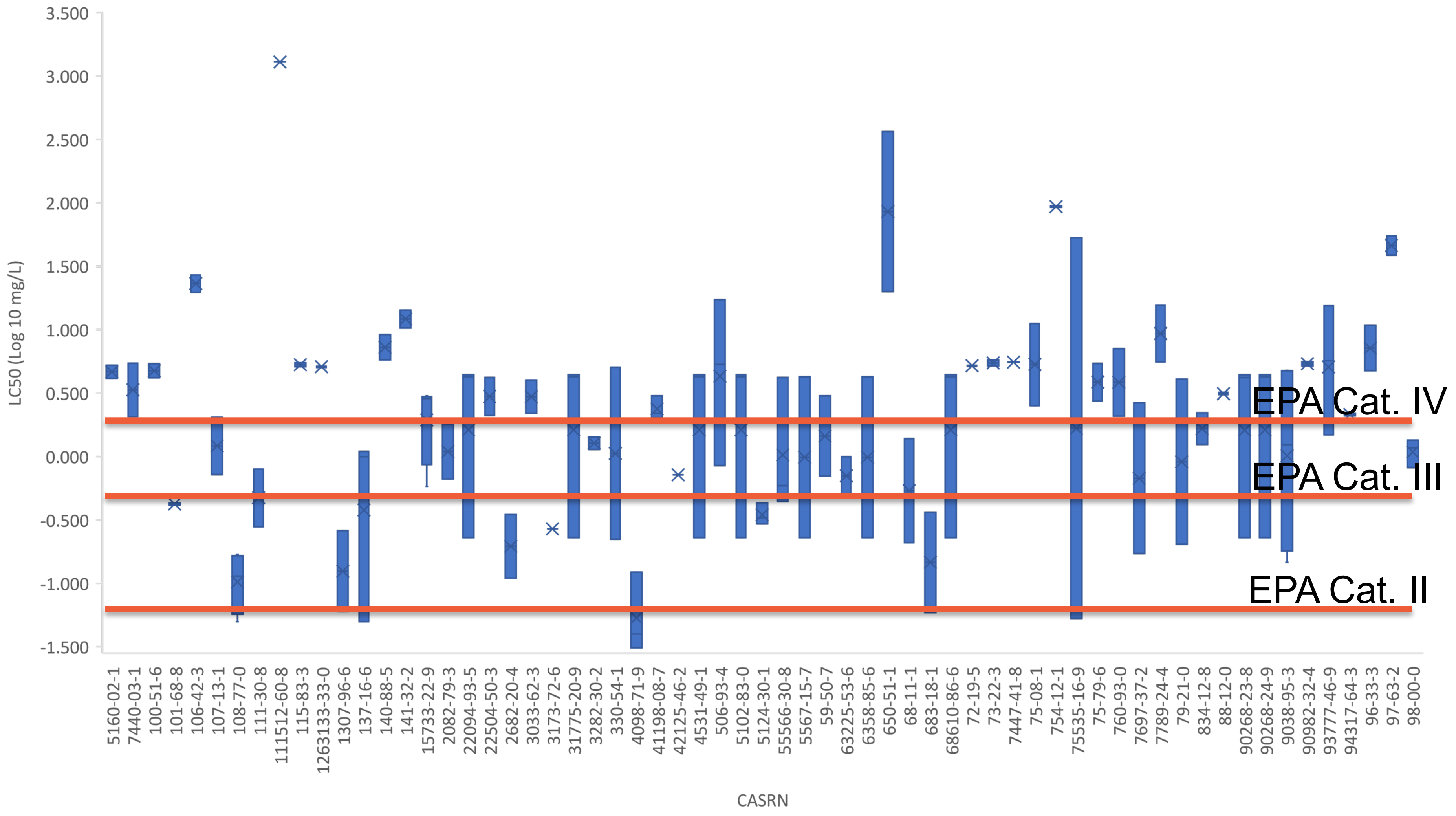

*unpublished data

Dermal Absorption Analyses

Allen et al. 2021 ALTEX

- Absorption through in vitro human skin was found to be similar to, or less than, that observed in rat skin (in vitro and in vivo) for all formulations.
- The human in vitro assay provided a similar or higher estimate of dermal absorption than the triple pack
- For human health risk assessment, in vitro assays using human skin would be preferable. Such tests would be directly relevant to the species of interest (humans) and avoid any overestimation of dermal absorption using rat models.

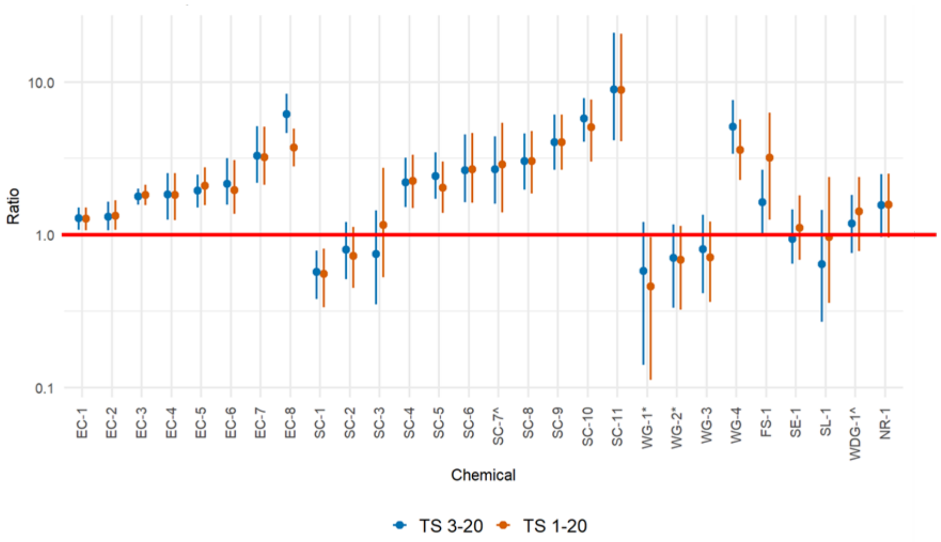

- However, rat in vitro studies would still have utility if human in vitro data were not available.
- In vitro rat data provide estimates of dermal absorption that are at least as protective as in vivo rat data, and thus could also be considered adequate for use in establishing dermal absorption factors.

$$triple\ pack\ DAF = rat\ in\ vivo \times (human\ in\ vitro \div rat\ in\ vitro)$$

Defined Approaches for Skin Sensitization

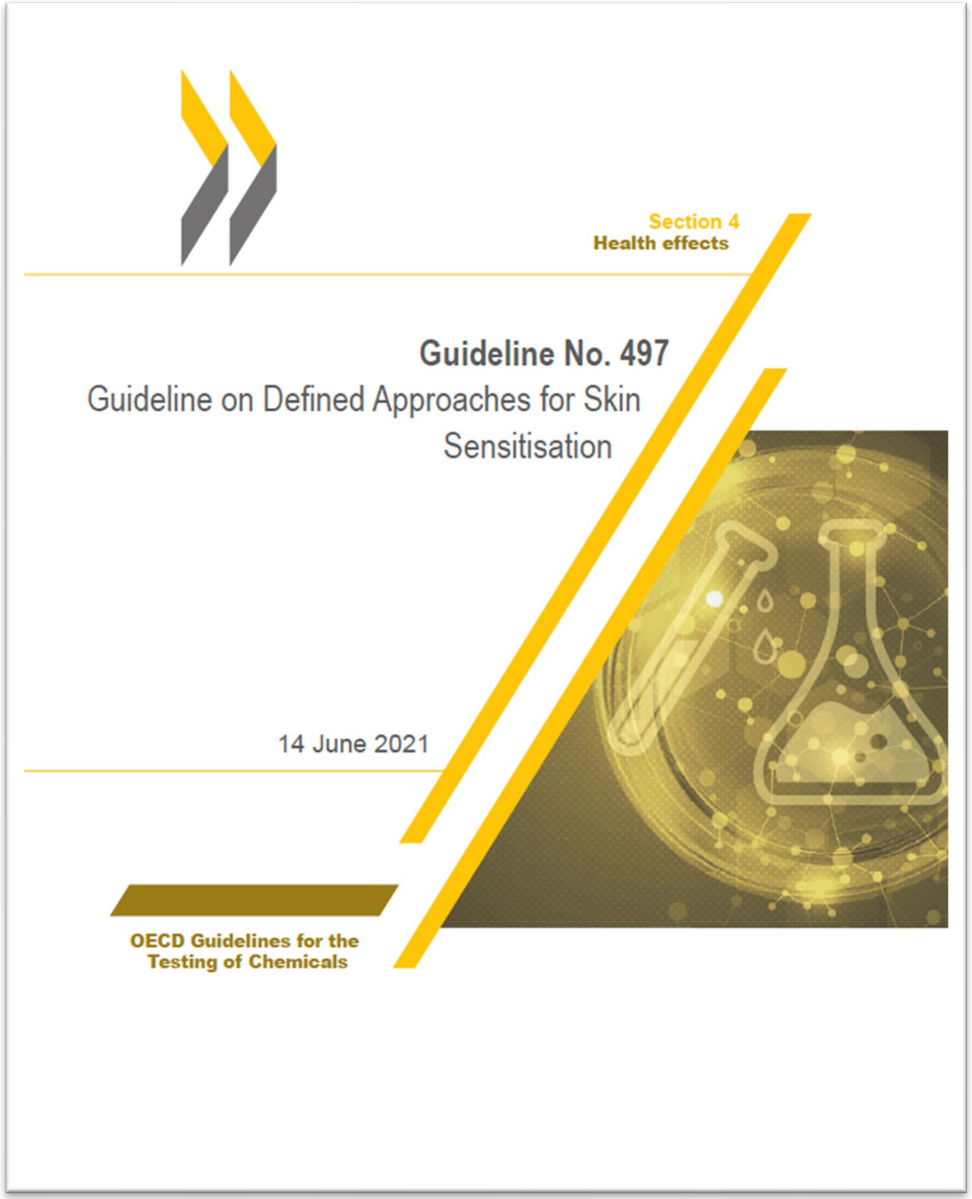

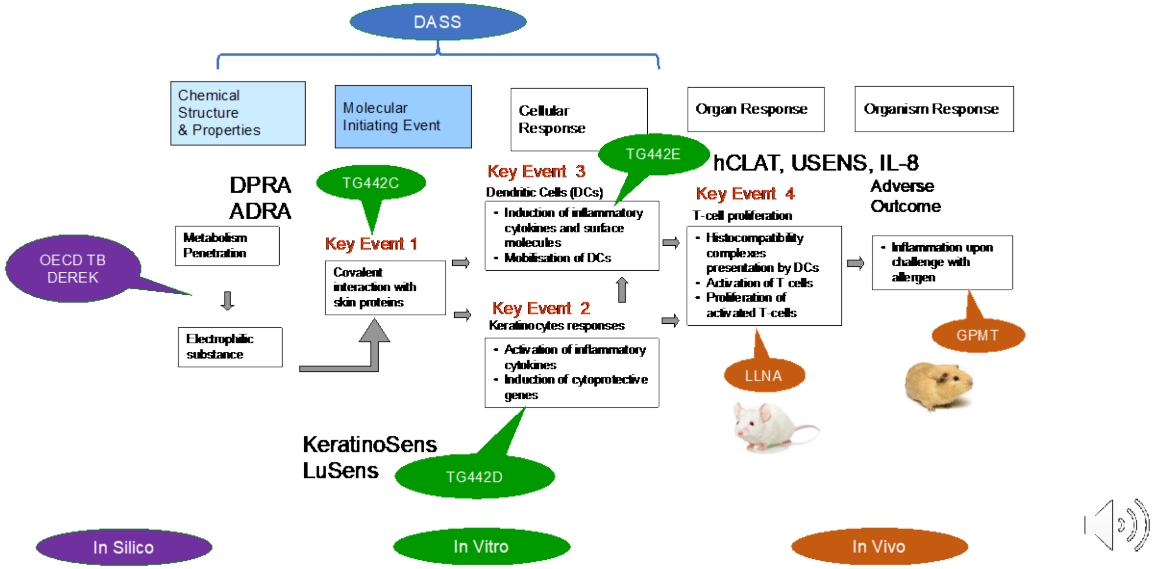

DA/Method	Information Sources	Capability (Hazard and/or Potency)	Hazard Performance vs. LLNA N~168	Hazard Performance vs. Human N~63	GHS Potency Performance vs. LLNA (Accuracy)	GHS Potency Performance vs. Human (Accuracy)
2o3 DA	DPRA, KeratinoSens™, h-CLAT	Hazard	84% BA, 82% Sens, 85% Spec	88% BA, 89% Sens, 88% Spec	-	-
ITSv1 DA	DPRA, h-CLAT, DEREK Nexus v6.1.0	Hazard, Potency (GHS)	81% BA, 92% Sens, 70% Spec	69% BA, 93% Sens, 44% Spec	70% NC, 71% 1B, 74% 1A	44% NC, 77% 1B, 65% 1A
ITSv2 DA	DPRA, h-CLAT, OECD QSAR Toolbox v4.5	Hazard, Potency (GHS)	80% BA, 93% Sens, 67% Spec	69% BA, 94% Sens, 44% Spec	67% NC, 72% 1B, 72% 1A	44% NC, 80% 1B, 67% 1A
LLNA (provided for comparison)	*in vivo*	Hazard, Potency	-	58% BA, 94% Sens, 22% Spec	-	25% NC, 74% 1B, 56% 1A

Regulatory Risk Assessment

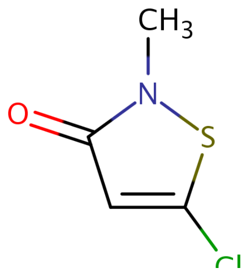

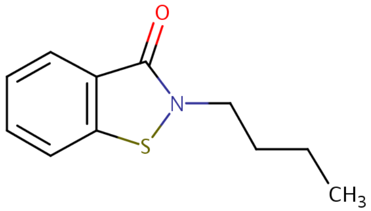

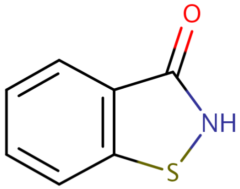

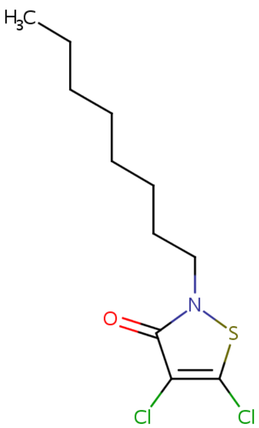

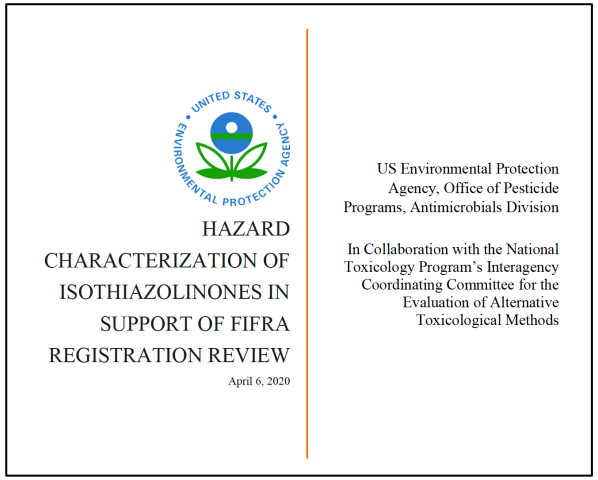
UNITED STATES ENVIRONMENTAL PROTECTION AGENCY

HAZARD CHARACTERIZATION OF ISOTHIAZOLINONES IN SUPPORT OF FIFRA REGISTRATION REVIEW

April 6, 2020

US Environmental Protection Agency, Office of Pesticide Programs, Antimicrobials Division

In Collaboration with the National Toxicology Program's Interagency Coordinating Committee for the Evaluation of Alternative Toxicological Methods

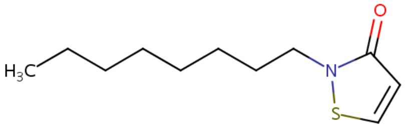

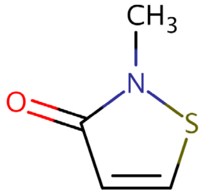

DASS for Isothiazolinone biocides: material preservatives to prevent the growth of microbial organisms and are used in industrial processes and consumer products

https://www.federalregister.gov/documents/2020/05/14/2020-10376/pesticide-registration-review-draft-human-health-and-ecological-risk-assessments-for-several

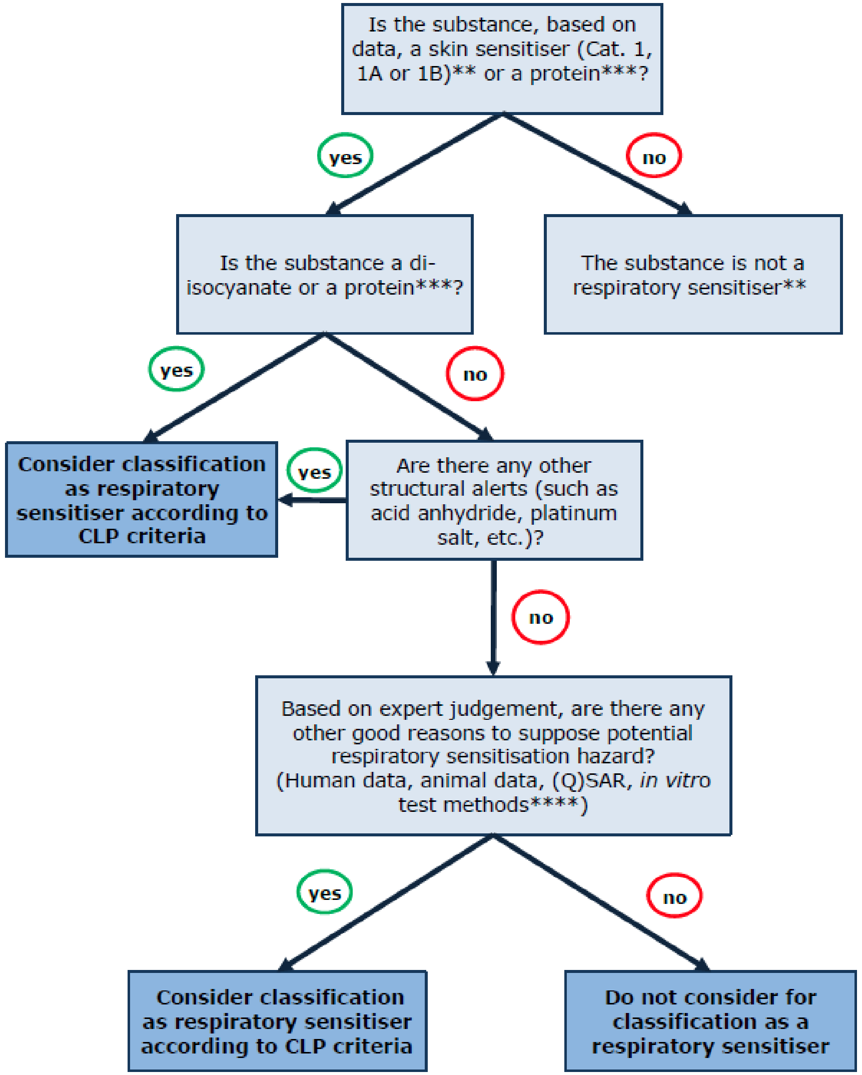

Decision tree for respiratory sensitiza[illegible]n

Fabrice Broeckaert & Laura Rossi, ECHA

Integrated Chemical Environment ICEv3.4

Users	Resources	Outcomes
Method Developers<br>Chemical Producers<br>Risk Assessors	High-Quality Data<br>Reference Chemicals<br>Computational Tools	• Identify opportunities to develop new methods<br>• Compare method performance<br>• Identify data gaps<br>• Obtain and examine toxicity and chemical data<br>• Develop testing strategies

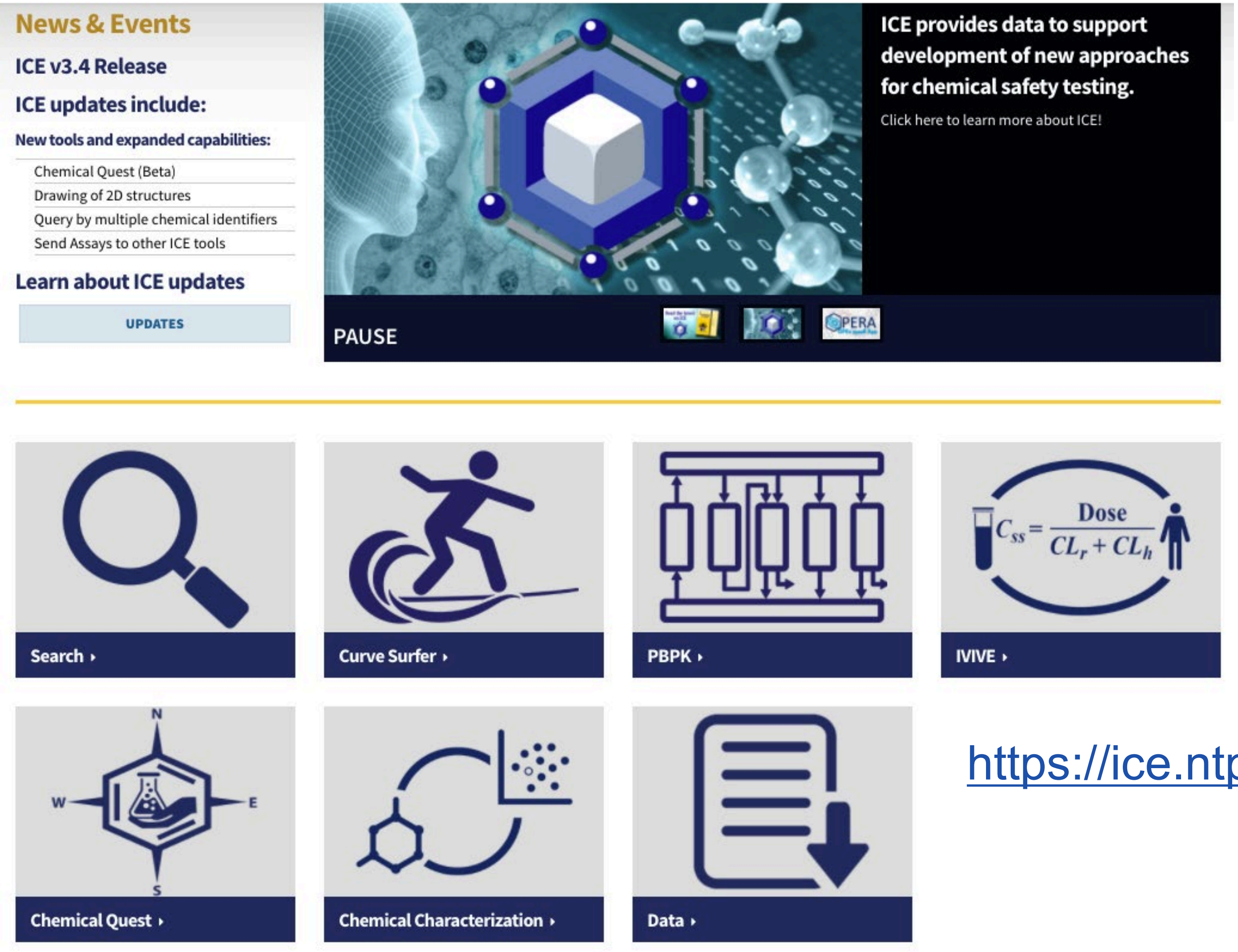

https://ice.ntp.niehs.nih.gov/

Bell et a[illegible]17 EHP
Bell et al. 2020 Tox In Vitro
Abedini et al. 2021 CompTox

Open Structure-Activity/Property Relationship App

- OPERA is a free and open-source quantitative structure-activity relationship (QSAR) tool with a suite of AI/ML models.
- OPERA predictions include:
  - Physchem properties
    - General structural properties
    - Environmental fate
  - ADME properties
  - Tissue partition coefficient inputs
  - Models for Toxicity Endpoints
    - CERAPP: Collaborative Estrogen Receptor Activity Prediction Project
    - CoMPARA: Collaborative Modeling Project for Androgen Receptor Activity
    - CATMoS: Collaborative Acute Toxicity Modeling Suite

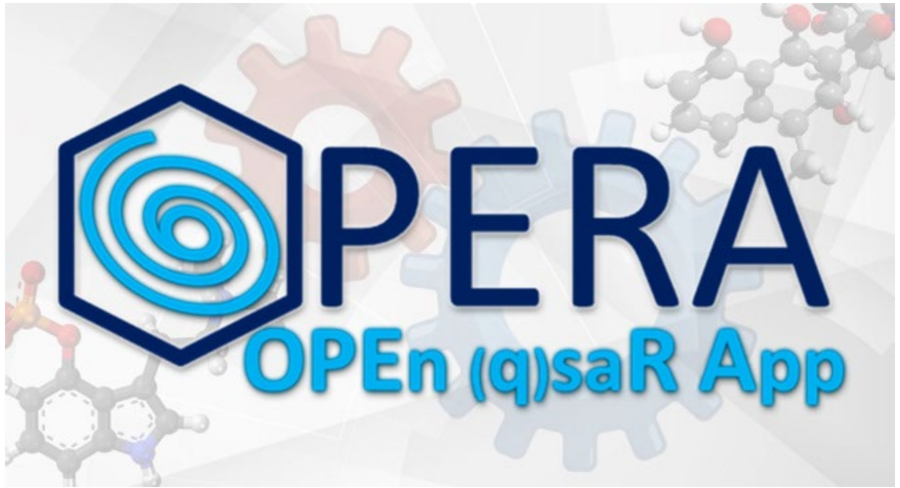

https://github.com/NIEHS/OPERA

Mansouri et al. J Cheminform (2018)

Curation to assist meaningful assay selection and model building

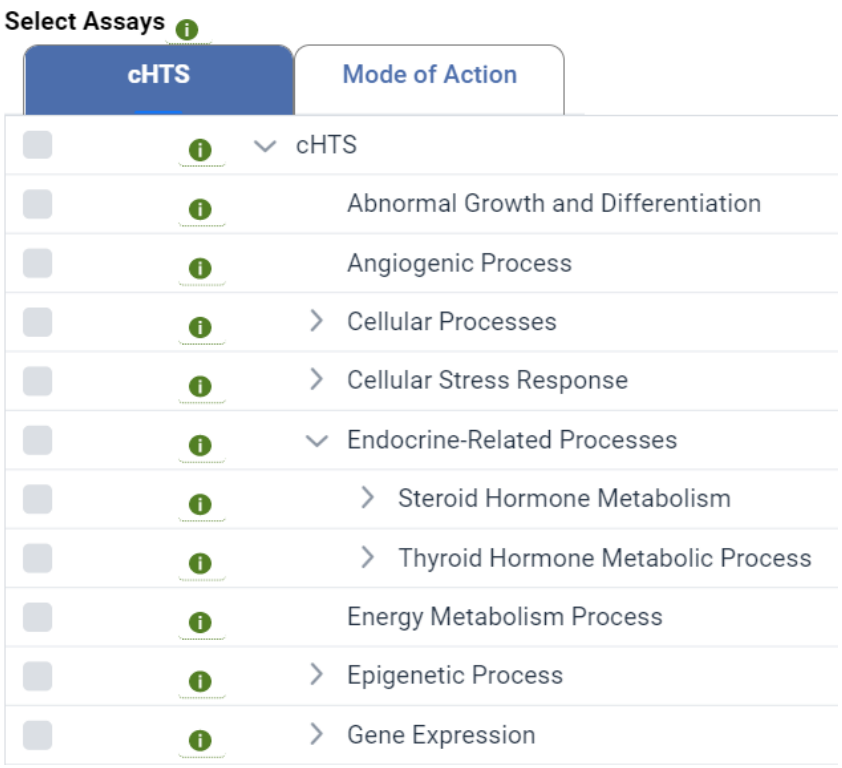

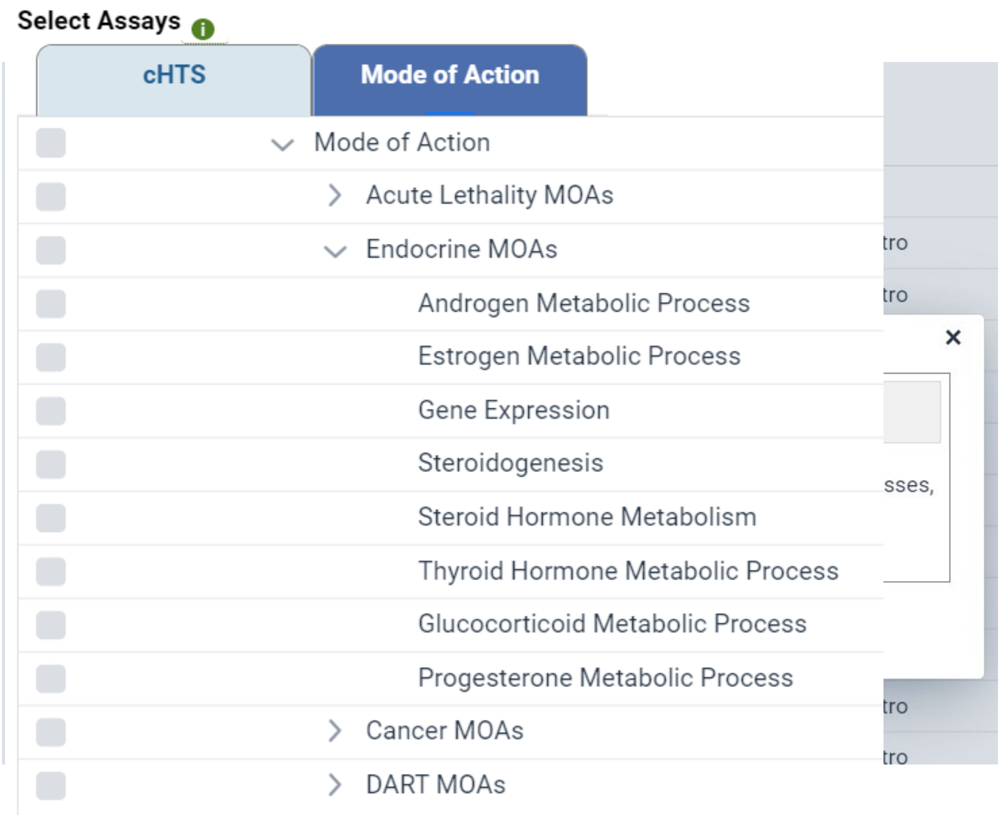

- Curated high-throughput screening data (cHTS) starts with EPA invitrodb and incorporates chemical QC information and technology-specific flags
- Assays are grouped by biological process, mechanistic target, and MoA, and linked to ontologies

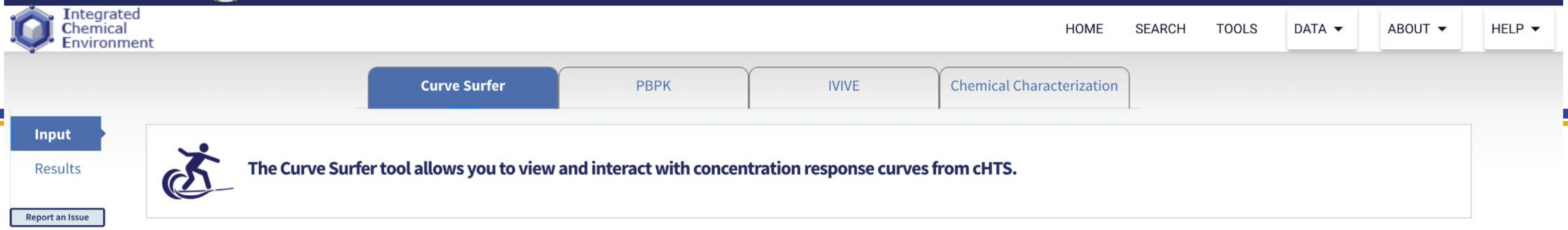

Curve Surfer is an interactive concentration response visualization tool for cHTS data

- Select/filter assays based on Mechanistic Target
- View specific assays/chemicals
- Filter on activity call, AC50

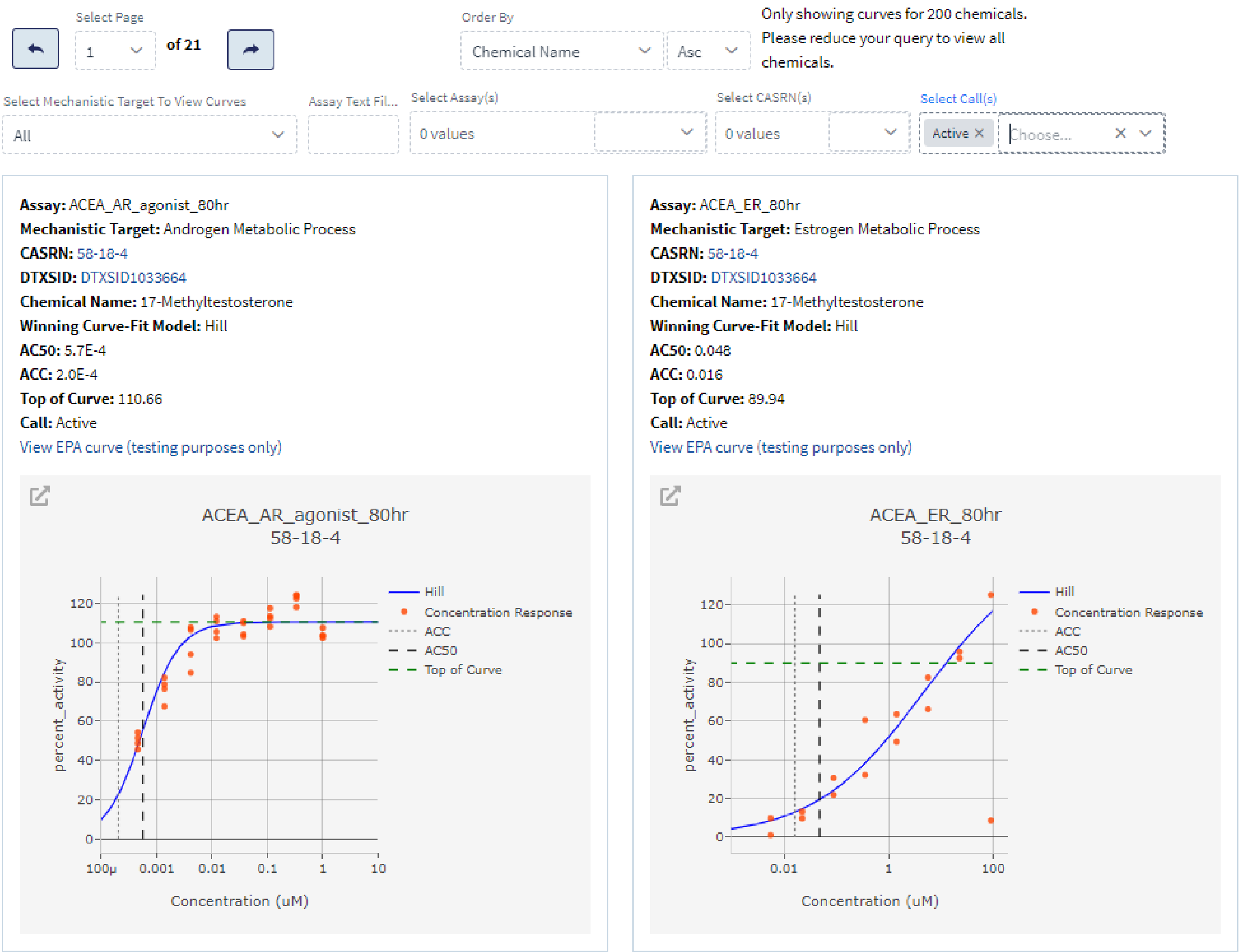

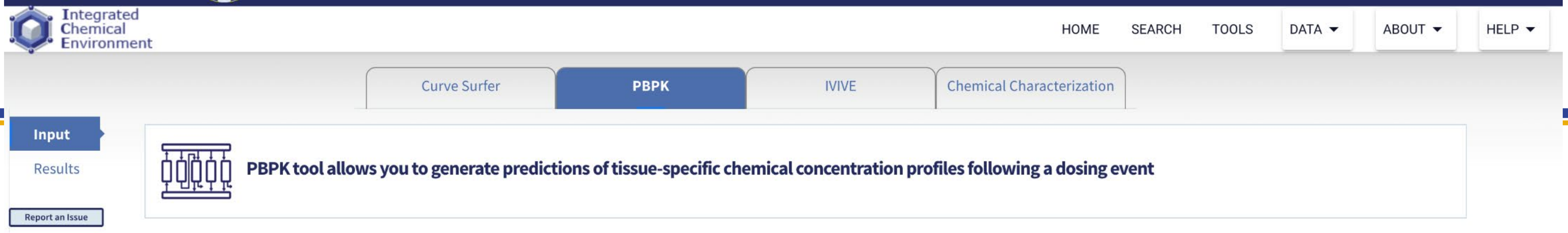

PBPK tool allows users to calculate internal chemical concentrations using PBPK models from the EPA httk R package and in-house code

- Tissue level concentrations
- View individual chemical curves
- View overall distribution in different tissue compartments for all query chemicals

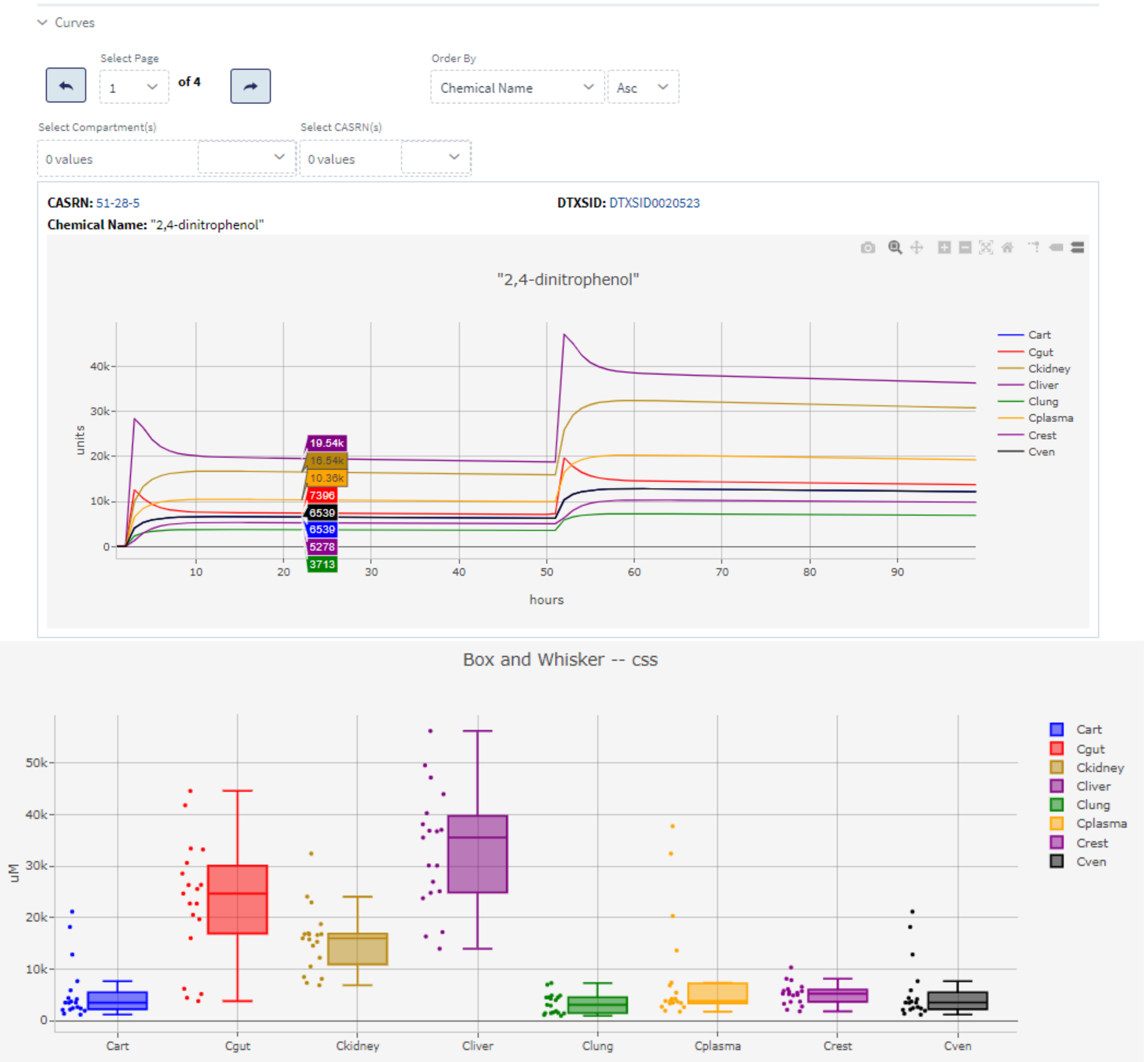

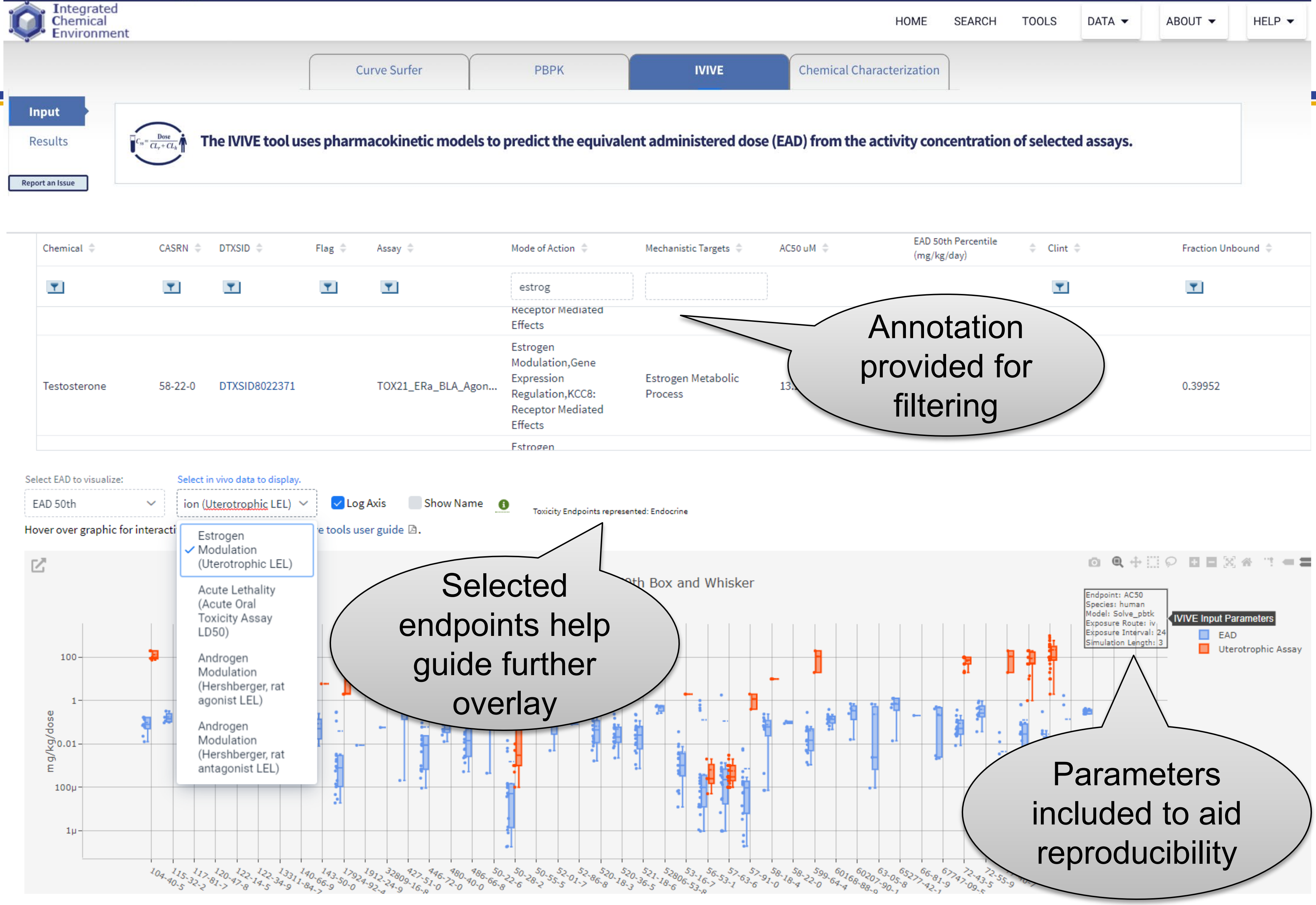

Transparency and annotation to help guide use and interpretation

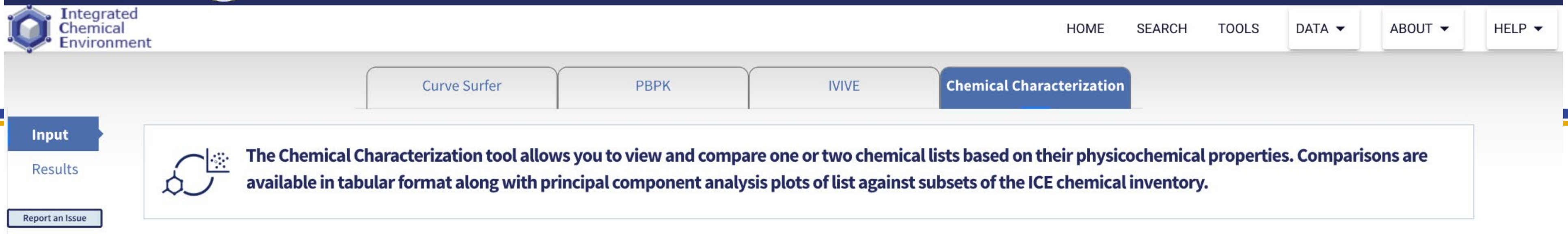

Chemical Characterization tool allows users to explore one or two chemical lists.

- Physicochemical property distributions
- Interactive PCA plots of chemical space coverage
- Presence in consumer products (EPA CPDat)

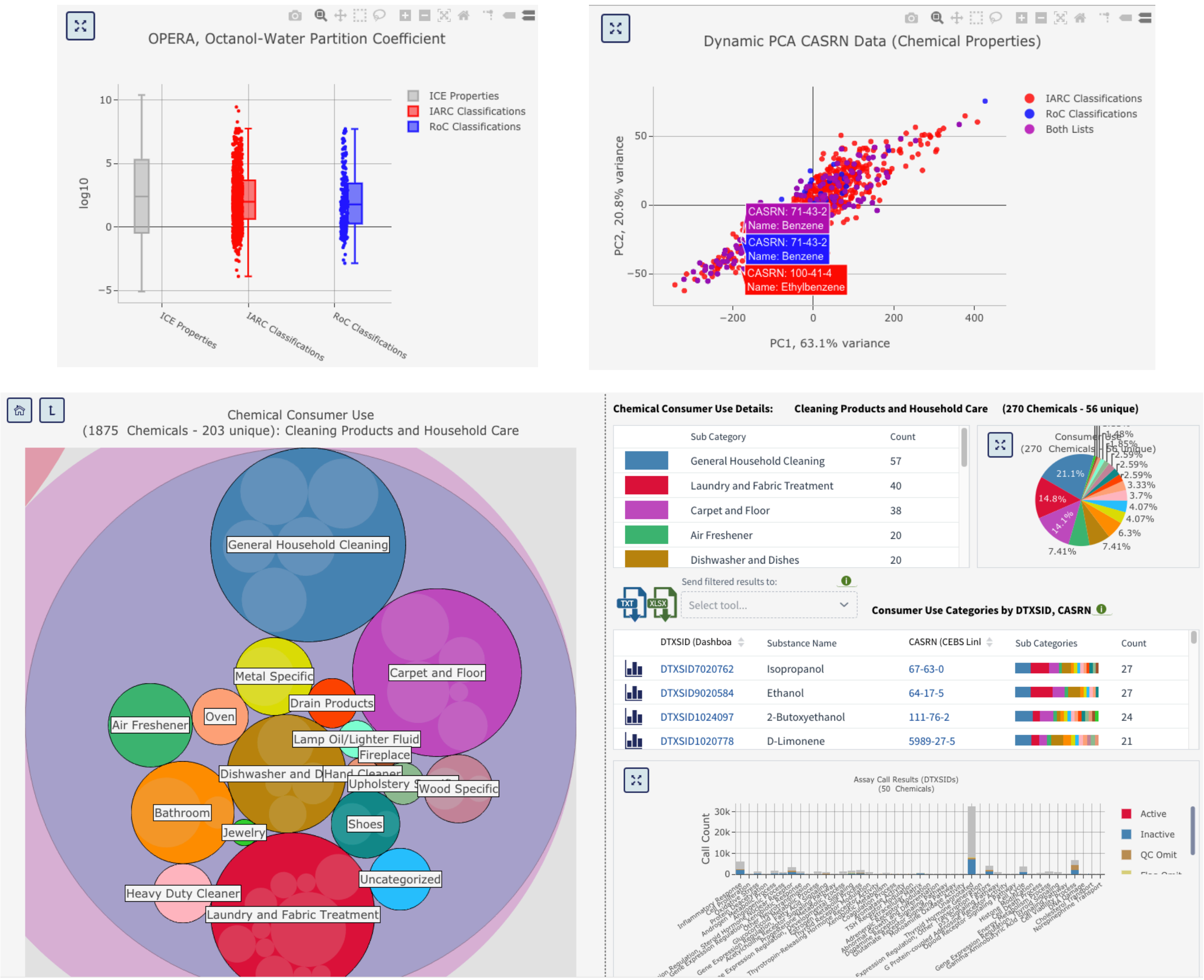

IVIVE for exposure and health impacts of e-cigarette flavor mixtures

Chang et al. 2021 Tox In Vitro

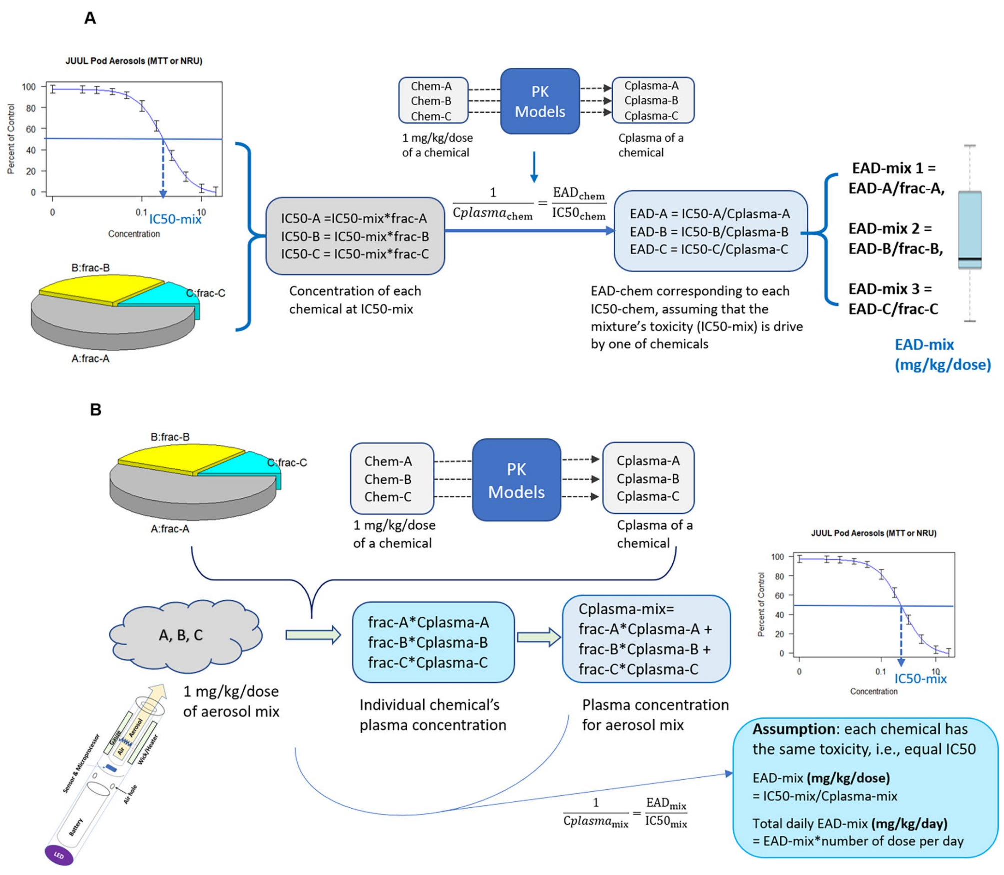

- Explored impact of PK modeling approach, handling of chemical mixtures for IVIVE modeling, and selection of *in vitro* assays.
- Considered results of cytotoxicity assays and *in vitro* assays that have more diverse and specific mechanistic (sub toxic) targets – stronger relevance to human health risk.

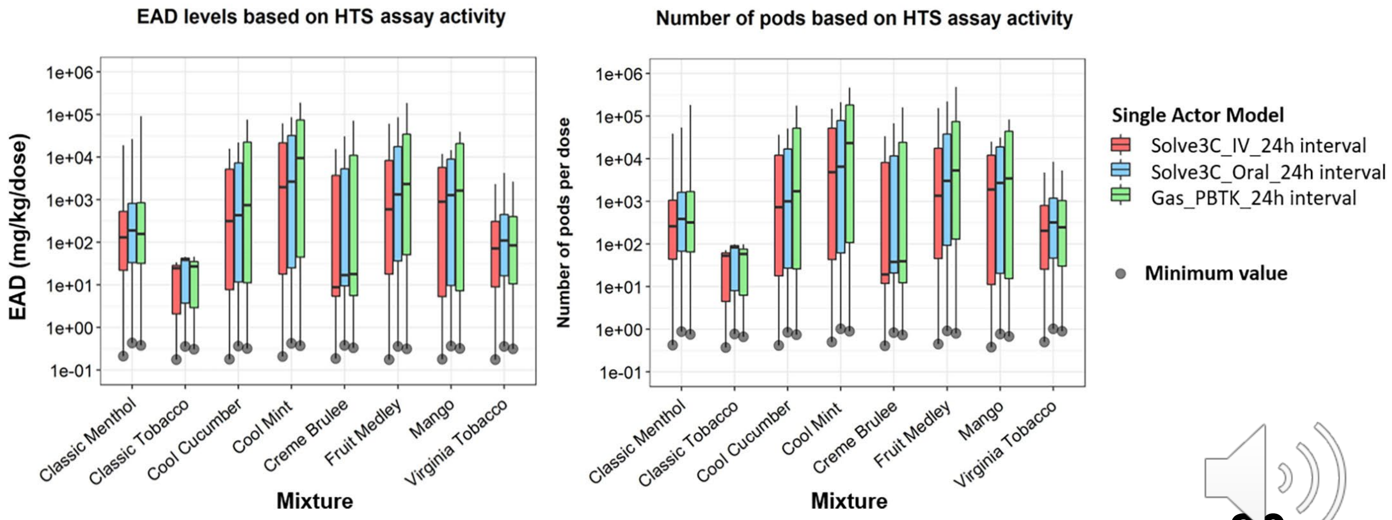

Take Home Messages

- Many computational toxicology tools are available for generation and interpretation of NAM data.
- Flavor ingredients and other components can be effectively tested in in vitro assays.
- In vivo inhalation data, like many in vivo toxicology study designs, suffer from lack of reproducibility.
- NAMs are accepted alternatives for many acute toxicity endpoints.
- Combining kinetic modeling and in vitro concentration response data can serve as an effective toxicity screening approach.
- Engaging regulatory authorities early in the process is critical for effective NAM implementation.

Acknowledgments: The NICEATM Group

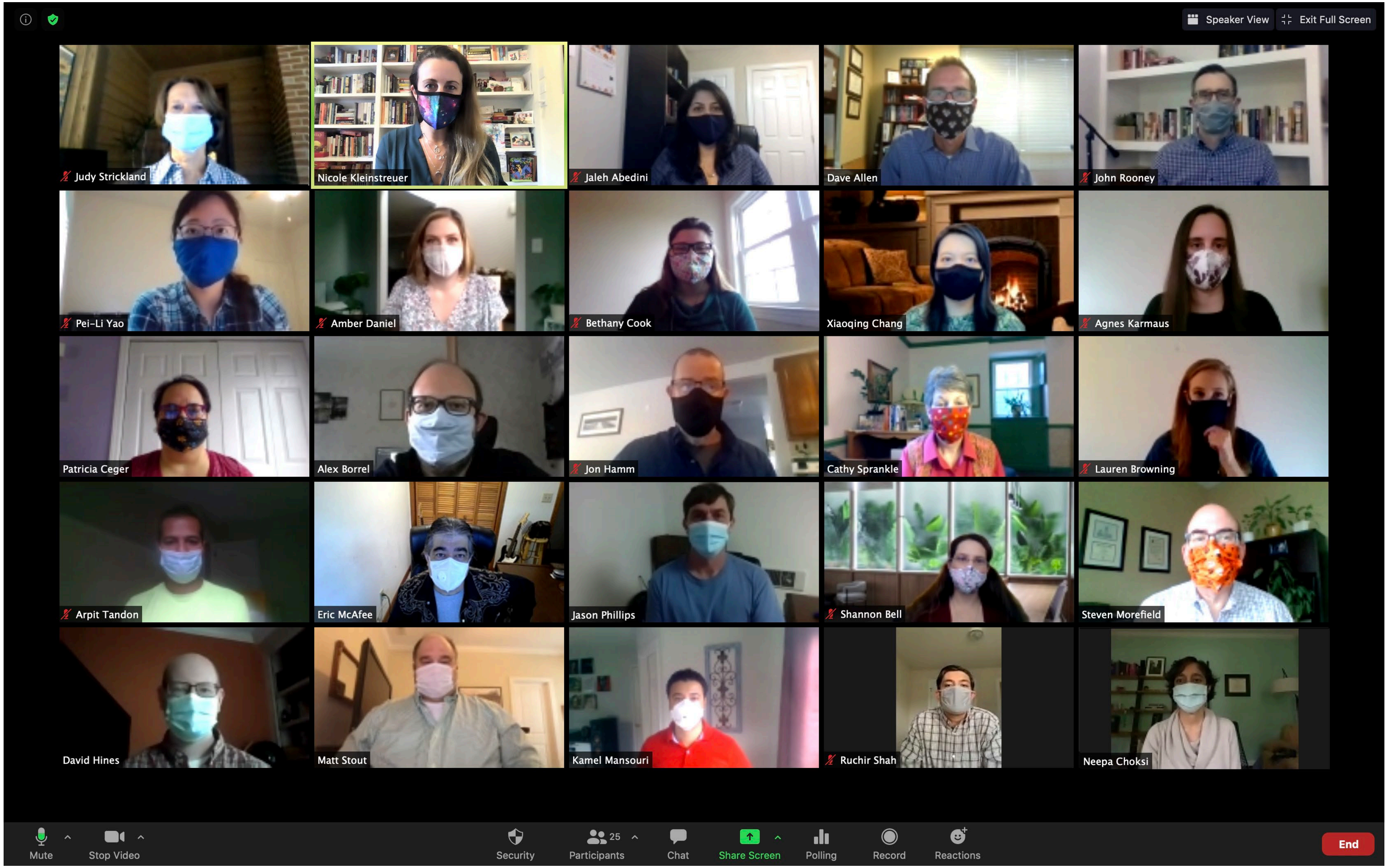

Acknowledgments: The NICEATM Group

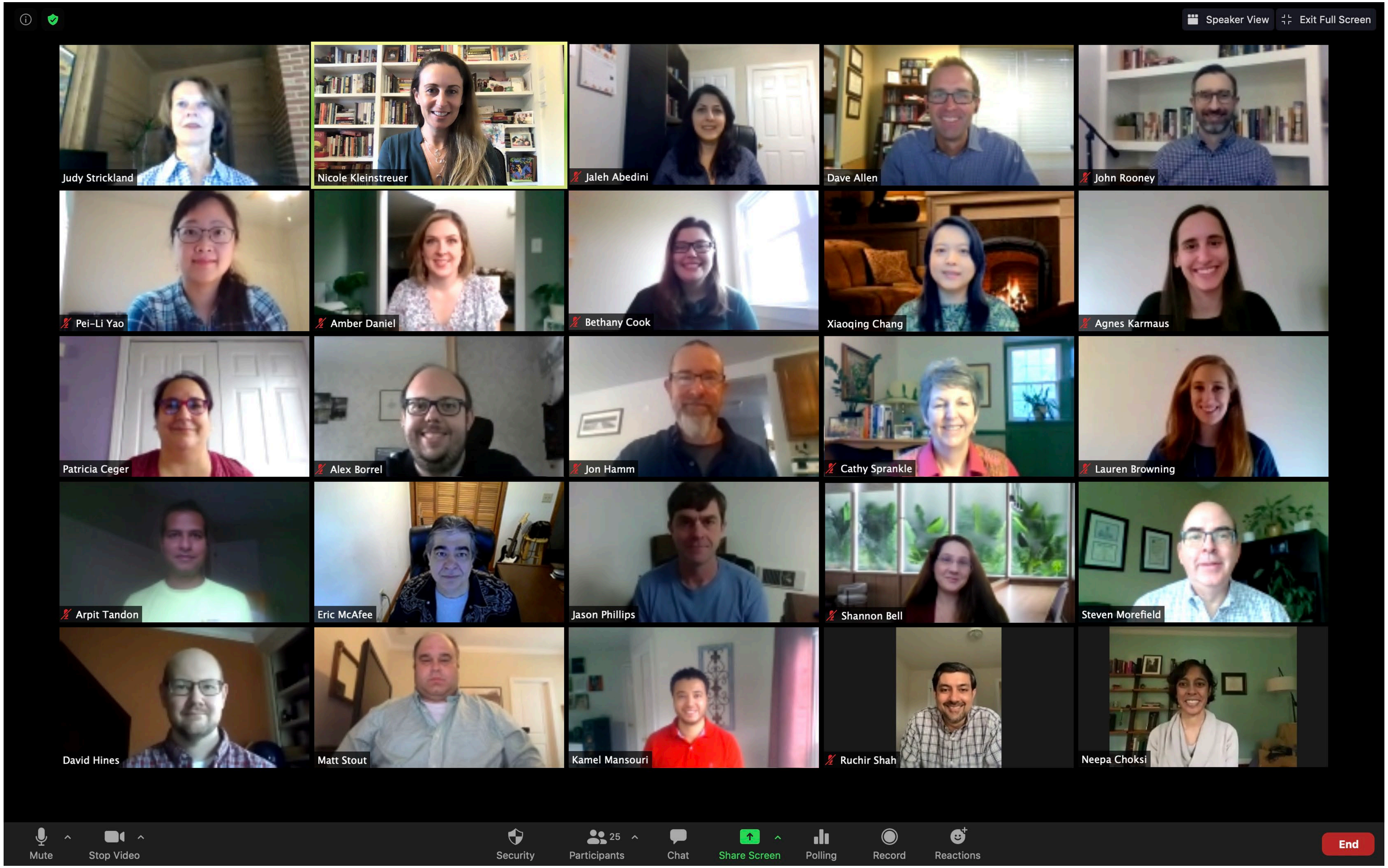

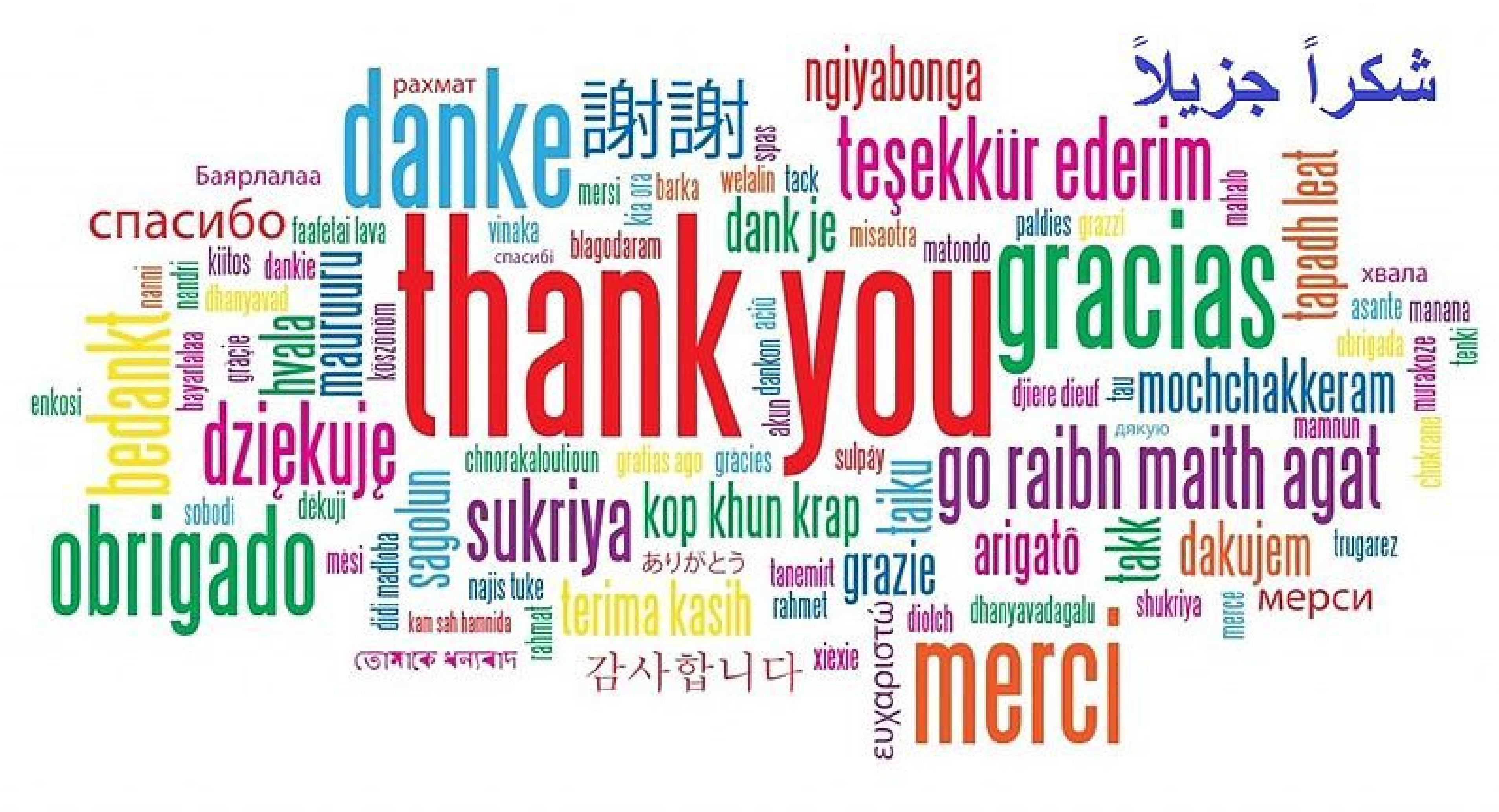
thank you
danke
謝謝
ngiyabonga
شكراً جزيلاً
рахмат
teşekkür ederim
Баярлалаа
спасибо
faafetai lava
mersi
barka
welalin
tack
spas
kia ora
vinaka
спасибі
blagodaram
dank je
misaotra
matondo
paldies
grazzi
mahalo
tapadh leat
gracias
kiitos
dankie
dhanyavad
nanni
nandri
bedankt
mauruuru
hvala
köszönöm
gràcie
bayarlalaa
enkosi
aciū
dankon
akun
хвала
asante
manana
obrigada
murakoze
tenki
djiere dieuf
tau
mochchakkeram
дякую
mamnun
dziękuję
chnorakaloutioun
grafias ago
gràcies
sulpáy
taiku
go raibh maith agat
chokrane
sobodi
dėkuji
sagolun
sukriya
kop khun krap
obrigado
mèsi
didi madloba
najis tuke
ありがとう
tanemirt
grazie
arigatô
takk
dakujem
trugarez
kam sah hamnida
rahmat
terima kasih
rahmet
ευχαριστώ
diolch
dhanyavadagalu
shukriya
merce
мерси
তোমাকে ধন্যবাদ
감사합니다
xièxie
merci

Questions?

TK to Connect Metabolism and Variability in Humans

Toxicology Letters 312 (2019) 173–180
Contents lists available at ScienceDirect
Toxicology Letters
journal homepage: www.elsevier.com/locate/toxlet

Metabolism of triflumuron in the human liver: Contribution of cytochrome P450 isoforms and esterases

Rim Timoumi[a,b], Franca M. Buratti[c,*], Salwa Abid-Essefi[a], Jean-Lou C.M. Dorne[d], Emanuela Testai[c]

Toxicology Letters
journal homepage: www.elsevier.com/locate/toxlet

Inter-phenotypic differences in CYP2C9 and CYP2C19 metabolism: Bayesian meta-regression of human population variability in kinetics and application in chemical risk assessment

Nadia Quignot[a,1,*], Witold Więcek[b,1,*], Leonie Lautz[c], Jean-Lou Dorne[d], Billy Amzal[a]
[a] CERTARA, Paris, France

Computational Toxicology
journal homepage: www.elsevier.com/locate/comtox

Inter-ethnic differences in CYP3A4 metabolism: A Bayesian meta-analysis for the refinement of uncertainty factors in chemical risk assessment

Keyvin Darney[a,*], Emanuela Testai[b], Franca M. Buratti[b], Emma Di Consiglio[b], Emma E.J. Kasteel[c], Nynke Kramer[c], Laura Turco[b], Susanna Vichi[b], Alain-Claude Roudot[d], Jean-Lou Dorne[e], Camille Béchaux[a]
[a] Risk Assessment Department, French Agency for Food, Environmental and Occupational Health & Safety (ANSES), 14 rue Pierre et Marie Curie, Maisons-Alfort F-94701, France
[b] Department of Environment and Health, Istituto Superiore di Sanità, Viale Regina Elena 299, 00161 Rome RM, Italy
[c] Institute for Risk Assessment Sciences, Utrecht University, P.O. Box 80177, 3508TD Utrecht, The Netherlands
[d] Laboratoire des Interactions Epithéliums Neurones, Université Bretagne Loire (UBL), UFR Sciences et Techniques, 6 Av. Victor Le Gorgeu, CS93837, Cedex 3, Brest 29238, France
[e] European Food Safety Authority, 1a, Via Carlo Magno, 1A, 43126 Parma, PR, Italy

Environment International
journal homepage: www.elsevier.com/locate/envint

Bayesian meta-analysis of inter-phenotypic differences in human serum paraoxonase-1 activity for chemical risk assessment

K. Darney[a], E.E.J. Kasteel[b], F.M. Buratti[c], L. Turco[c], S. Vichi[c], C. Béchaux[a], A.C. Roudot[d], N.I. Kramer[b], E. Testai[c], J.L.C.M. Dorne[e], E. Di Consiglio[c], L.S. Lautz[a,*]

Archives of Toxicology
https://doi.org/10.1007/s00204-020-02765-8
TOXICOKINETICS AND METABOLISM

Human variability in isoform-specific UDP-glucuronosyltransferases: markers of acute and chronic exposure, polymorphisms and uncertainty factors

E. E. J. Kasteel[1] · K. Darney[2] · N. I. Kramer[1] · J. L. C. M. Dorne[3] · L. S. Lautz[2]

Computational Toxicology
journal homepage: www.elsevier.com/locate/comtox

A generic Bayesian hierarchical model for the meta-analysis of human population variability in kinetics and its applications in chemical risk assessment

Witold Wiecek[a,*], Jean-Lou Dorne[b], Nadia Quignot[a], Camille Bechaux[c], Billy Amzal[a]

Covering Phase I CYP450 and Phase II UGTs enzymes

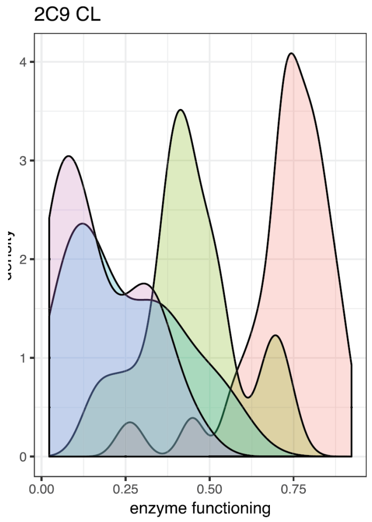

PBPK models + virtual population

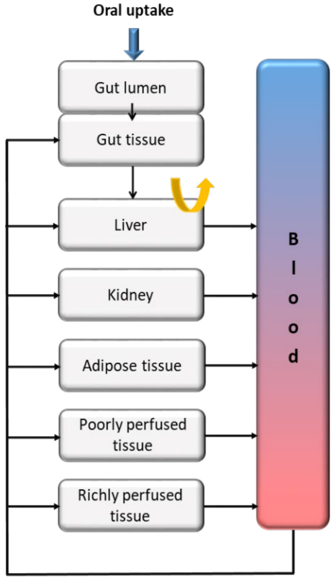

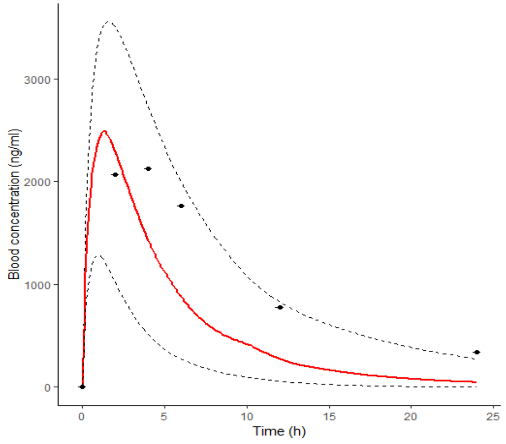

https://ice.ntp.niehs.nih.gov/

Pharmacokinetic data on compounds metabolised by UGT isoforms is collected and summarised in a database

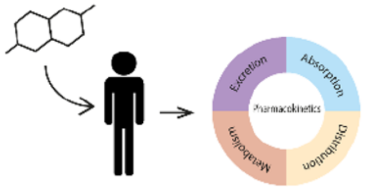

Data on polymorphism frequencies in different populations is collected and summarised

Interindividual differences in kinetics and polymorphisms

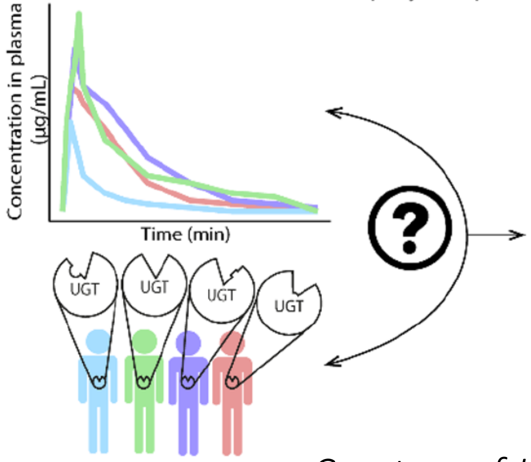

UGT-related uncertainty factors

Courtesy of Jean-Lou Dorne

European Food Safety Authority

Application of biokinetic modelling for in vitro-in vivo extrapolation (IVIVE) in chemical risk assessment

Alicia Paini & Andrew Worth

European Commission Joint Research Centre (JRC), Ispra, Italy

Premise

- Chemical Risk Assessment can and should be based on non-animal data
- This implies the need to use alternatives such as in vitro and in silico methods
- Especially to interpret and use *in vitro* toxicity data in combination with biokinetic data
- Biokinetic (ADME) data can be generated by *in silico* and *in vitro* models
- Mathematical modelling is the way to accurately integrate and use in vitro data for the design of experiments and extrapolate in vitro to in vivo for safety assessment
- Robust and reliable mathematical models are available

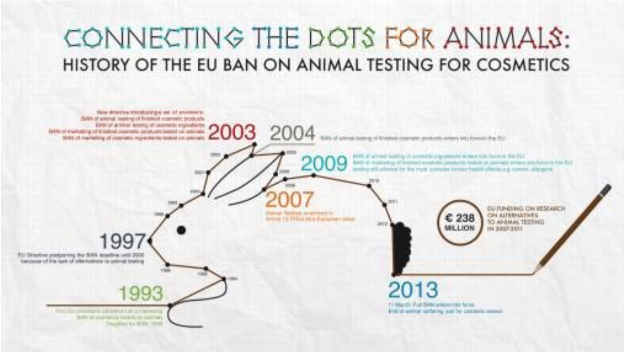

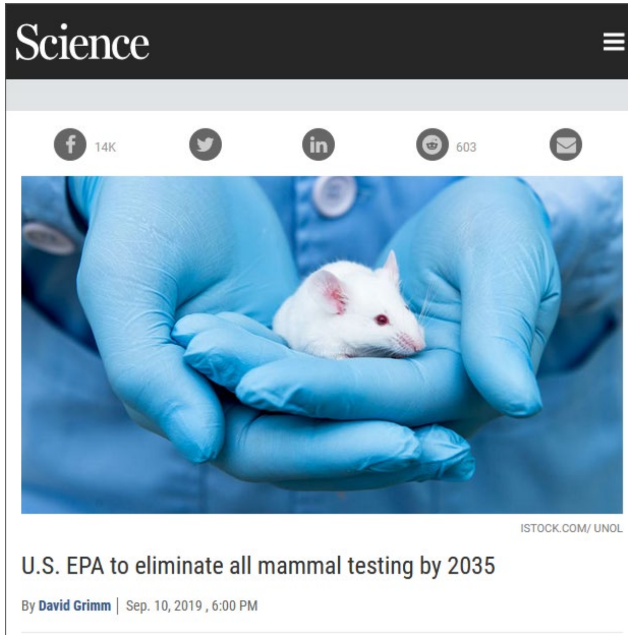

Pictures source: https://ec.europa.eu/growth/sectors/cosmetics/animal-testing_en
https://www.sciencemag.org/news/2019/09/us-epa-eliminate-all-mammal-testing-2035

What kinds of models are in scope?

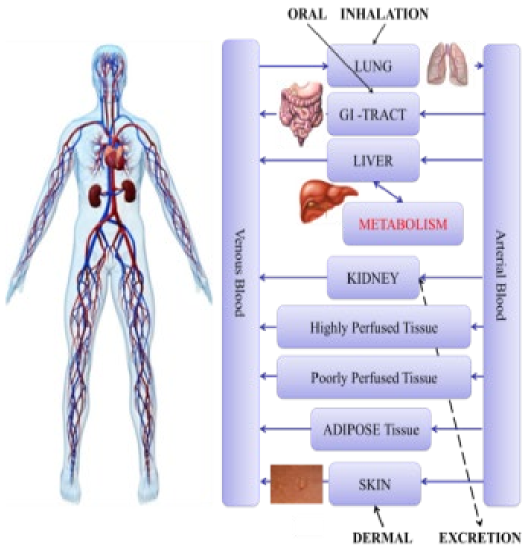

Physiologically based kinetic (PBK) model

Mathematical description of the body, simulating the xenobiotic distribution into the different organs.

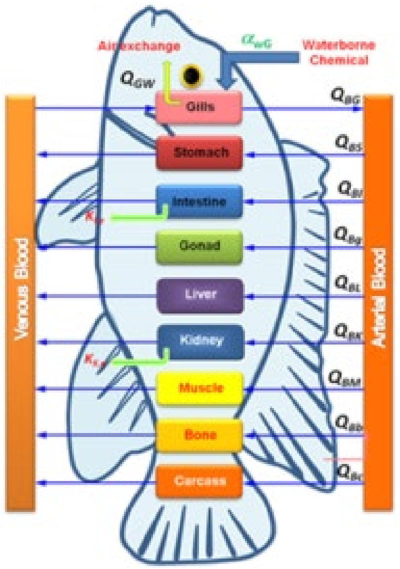

Throughout this presentation the more general term PBK will be used.
Noting that PBK, PBPK, PBBK and PBTK are synonyms.

Physiologically based pharmacokinetic (PBPK) is the most widely used term for kinetic models describing the absorption, distribution, metabolism and excretion of a drug within the body. Although widely used in the pharmaceutical sector, the "PBPK" term is not strictly correct in the area of chemical risk assessment. An alternative is "PBTK" with the TK representing toxicokinetic, but this is not appropriate either (Clewell & Clewell, 2008). ***More general terms, such as physiologically based biokinetic (PBBK) or physiologically based kinetic (PBK), are thus more appropriate.***

Fate and Distribution model

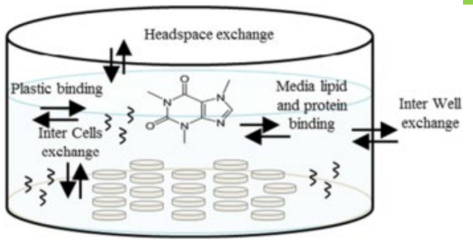

Mathematical description of the well, simulating the xenobiotic distribution into the different in vitro set up compartments.

Pictures source: https://ec.europa.eu/jrc/en/publication/eurl-ecvam-workshop-new-generation-physiologically-based-kinetic-models-risk-assessment (human)
https://www.frontiersin.org/articles/10.3389/fmars.2015.00114/full (fish)

In vitro to in vivo extrapolation

Stream 1
Scale up of parameters

PBK model parametrisation

Scale-up of in vitro data to in vivo is performed by analyzing the correlation between in vitro and in vivo data or applying physiological correction factors.
in vitro data provides the parameter values for developing a model.[

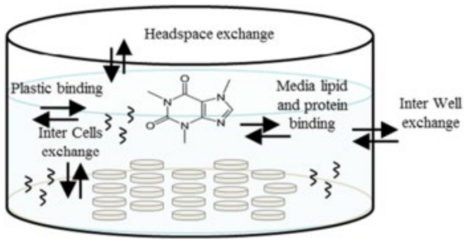

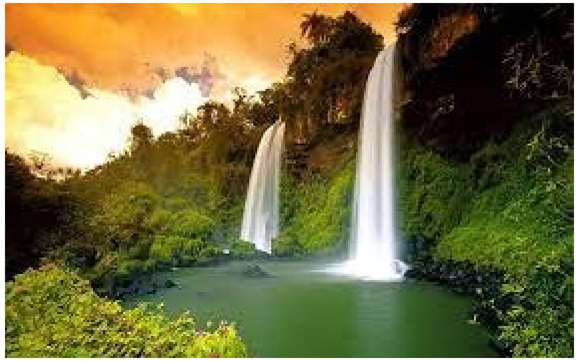

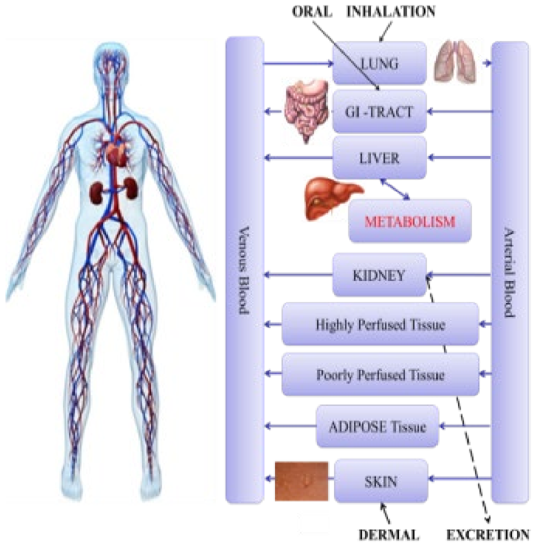

Stream 2
Reverse dosimetry

PBK model extrapolation

Translation of in vitro concentration effect curves into in vivo dose response curves.

Obtain an oral equivalent dose or a PoD.

Extrapolating adverse effects observed in vitro to an in vivo exposure.

Stream 1: Scale up of parameters

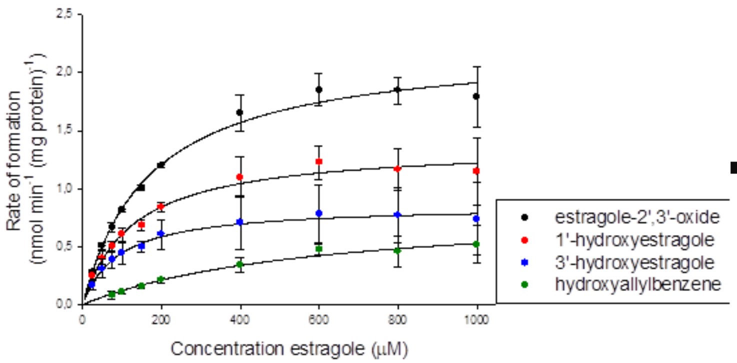

In vitro incubation rate of metabolism or clerance

nmol min-1 (mg protein)-1
nmol min-1 (mg S9 protein)-1
(measuring rate of formation)

→ V = Vmax (S) /Km + (S) →

Vmax → Needs to be scaled from in vivo to in vitro
Km → assumed to be tha same as the in vivo Km (uM)

Using scaling factors (form literature); hepatocullularity values or microsomal recovery factors, non specific binding and liver weights.

- Cyps abundance
- S9 abundance
- protein abundance (HLM)

Vmax, in vivo umol hr^{-1}
Clint,H, in vivo uL/min/g Liver →

In vitro to in vivo extrapolation of parameters

Incorporate of the scaled in vivo parameters in the PBK model
Liver model: Well stirred, parallel tube, dispersion

Yoon et al., 2012 June 2012
Critical Reviews in Toxicology 42(8):633-52
10.3109/10408444.2012.692115
Punt A. (2009) WUR PBK model course

Stream 2: Reverse dosimetry

Difference in exposure

Effect measured in vitro

- 5 or 10% serum
- Single cells
- High concentration
- Non bioaccumulation
- Plastics/Evaporation
- Short exposure
- Batch and experimental set up variability

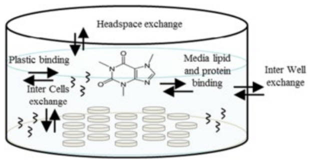

Effect measured in vivo

- 100% serum
- Connected complex cell system
- Low concentration
- Bioaccumulation
- No plastic/No evaporation
- Long exposure
- Inter-individual variability

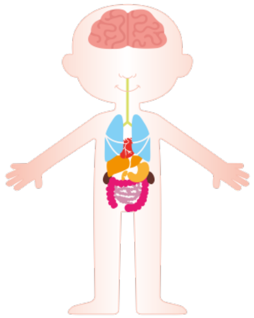

Difference in dose metrics

Maybe best dose metric: internal concentration

Adapted from presentation by Rendal et al., 2017,
NC3R event London 15-16 February 2017

Stream 2: Reverse dosimetry - Steps

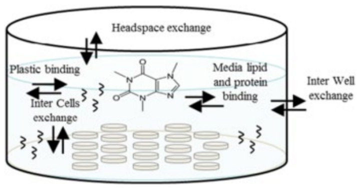

Nominal Concentrations

Experimental dilutions → **Distribution math models** → **Free conc**

- Armitage model (2014)
- Kramer model (2010)
- Zaldivar model (2016)
- Proenca et al (2021)

Free Concentrations

*Extrapolation Translation

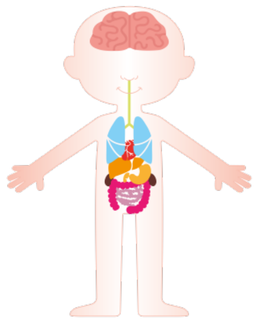

Exposure dose → **PBK models** → **Organ concentration**

mg/kg BW

- Httk
- PKSim
- SymCip
- Simulation plus
- IndusChemFate
- MeGEN
- Berkley Madonna/Matlab/R
- Among others

Cmax & AUC

*Assumption that the free concentration in the assay and the organ concertation can be considered =
Paini et al., 2017, Tox in vitro – OECD IATA 2020 – under review Systemic Toxicity of Phenoxyethanol – Pistollato et al., 2021 Rep.Tox

Stream 2: Reverse dosimetry – endpoint

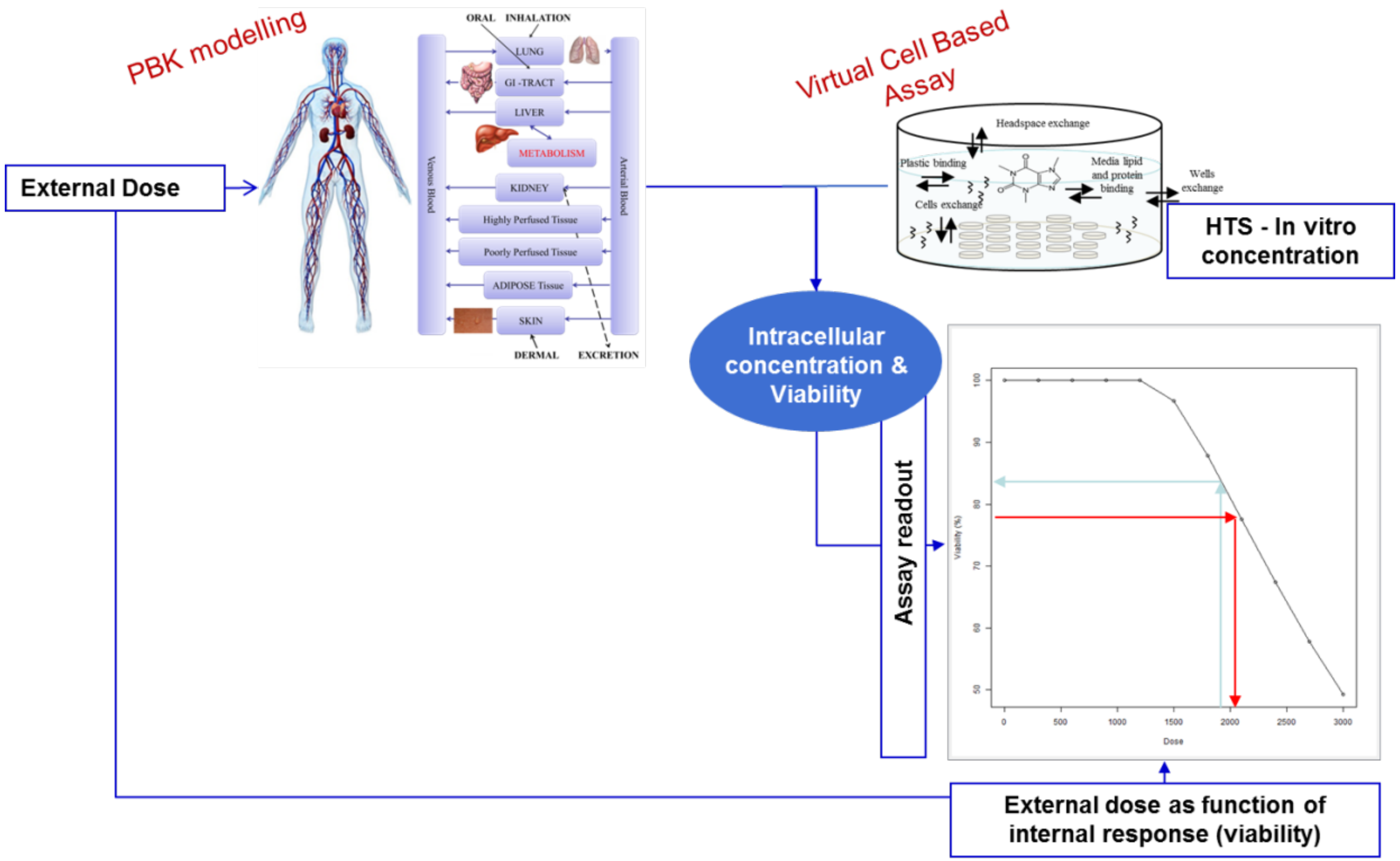

This strategy has been applied to a number of **toxicological endpoints** including **developmental toxicity, genotoxicity, acute toxicity and hepatotoxicity, nephrotoxicity, neurotoxicity, and, more recently, endocrine disruption**.

Source for the different endpoint: Gubbelsvan Hal et al. 2005, Verwej et al. 2006, Forsby and Blaauboer 2007, Paini et al. 2010, **Louisse et al. 2010**, 2015; **Wetmore et al., 2012**, Strikwold et al. 2013, 2017; Li et al. 2017; Abdullah et al. 2016, Zhang et al. 2018, Fabien et al., 2019.

How to accurately integrate in vitro data

Stream 1
Scale up

Several PBK models
available in the literature

PBK model parametrisation

IVIVE

How to gain confidence???

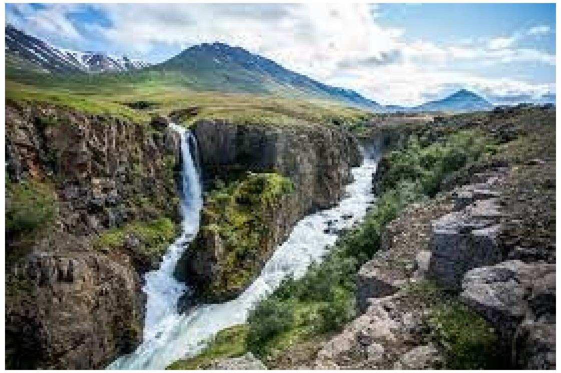

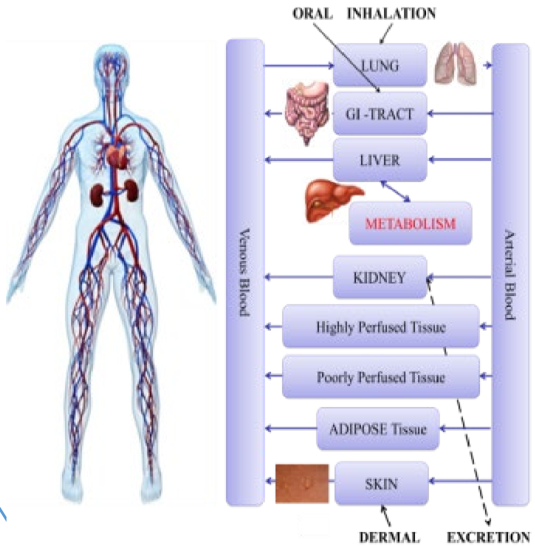

Stream 2
Reverse dosimetry

PBK model extrapolation

QIVIVE

Connected Streams
PB(P)K modelling

Connected Streams

In vitro input parameters → OECD TG & GD (OHTs) or GIVIMP

In silico input parameters → OECD QSAR GD - QMRF

Evaluation/qualification/validation PBK model → OECD PBK model GD

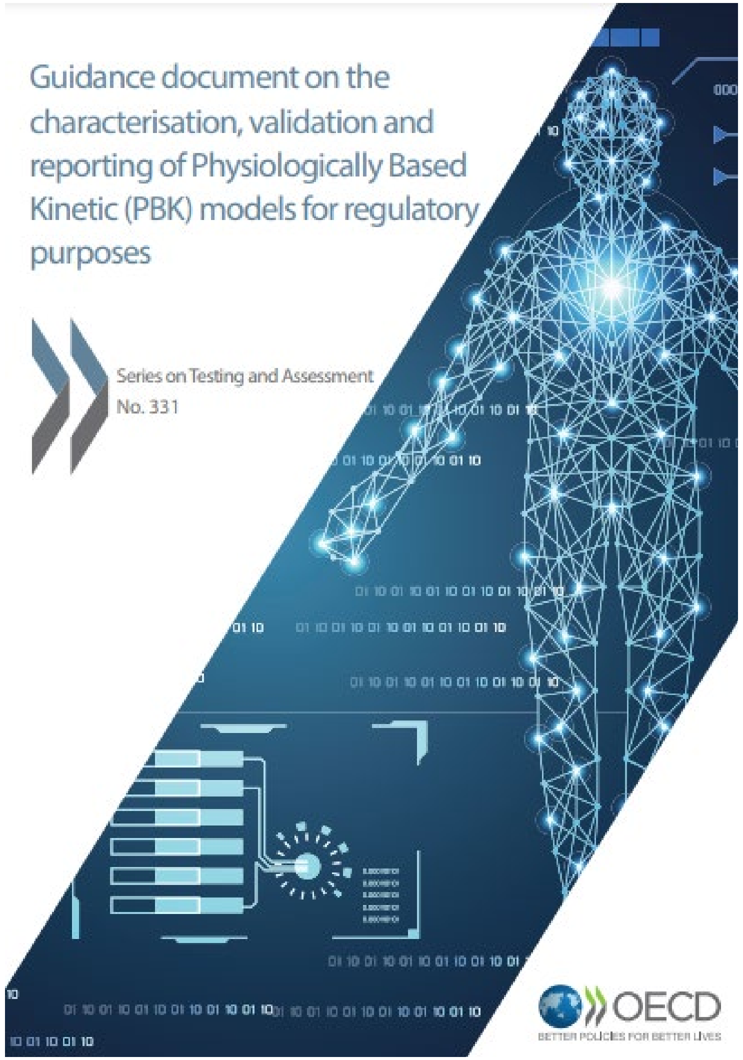

OECD PBK model GD
Purpose and scope

- Provide guidance on characterising, reporting, and evaluating PBK models used in regulatory assessment of chemicals
- Address challenges associated with developing and evaluating PBK models for chemicals without *in vivo* kinetic data
- Promote the use of PBK models in regulatory risk assessment and facilitate dialogue between model developers and users

Contents of OECD Guidance Document

1. PBK Model workflow

- Step 1
  - Problem formulation and model conceptualisation
- Step 2
  - Model parameterisation
- Step 3
  - Solving the equations
- Step 4
  - Model validation
    - Sensitivity, variability and uncertainty analyses
    - Predictive capacity
- Step 5
  - Model reporting and dissemination

Scientific workflow for characterising and validating PBK models, with emphasis on the use of in vitro and in silico data for absorption, distribution, metabolism and excretion (ADME) parameters, and in scenarios where in vivo kinetic data are limited or unavailable to parameterise model parameters

Contents of OECD Guidance Document

2. Regulatory assessment framework of PBK models

Context & Implementation

- Regulatory purpose
- Model applications
- Software implementation
- Peer input / review
- Documentation

Model validity

- Biological basis (model structure and parameters)
- Theoretical basis of model equations
- Reliability of input parameters
- Sensitivity of output to parameters
- Goodness-of-fit and predictivity

Reporting template

Evaluation checklist

Develop an assessment framework for evaluating PBK models, with emphasis on the major uncertainties underlying the model predictions.

Contents of OECD Guidance Document

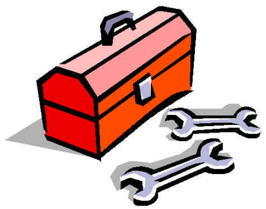

3. PBK model Evaluation tool box

1. Model Reporting Template

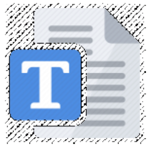

		Uncertainty in variability of the input parameter estimates		
		High	Medium	Low
SENSITIVITY	High		Parameter 1	Parameter 3<br>Parameter 4<br>Parameter 7
	Medium		Parameter 2	Parameter 10
	Low			Parameter 12<br>Parameter 13

2. Evaluation Checklist

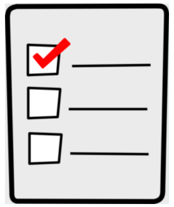

3. Overall Evaluation Matrix (adapted from WHO 2010)

LEVEL OF CONFIDENCE: NONE ⟷ HIGH

	NONE		HIGH
**Biological basis**	The model parameters, structure or assumptions are consistent with neither the biology nor the current state of knowledge regarding the kinetics of the chemical.	The biological basis of some model parameters, structural elements or assumptions is questionable.	The model parameters and structure have reasonable biological basis and are consistent with available kinetic data in several experiments using a single set of input parameters .
**Model simulations of data**	Model is unable to reproduce the shape (i.e. bumps, valleys) of the kinetic time course curves, neither for the chemical of interest nor for a suitable analogue.	Model reproduces the shape of part but not all of the kinetic time course curves, either for the chemical of interest or suitable analogue.	Model reproduces consistently all kinetic data, including the shape of time course profiles for chemical of interest.
**Uncertainty in input parameters and model output; Sensitivity of model output to input**	No uncertainty and sensitivity analyses were performed	Local Sensitivity Analysis supports the robustness of the model.	Global Sensitivity Analysis supports the robustness of the model.

PUBLICATIONS ON TESTING AND ASSESSMENT

No. 331 Guidance Document on the Characterisation, Validation and Reporting of PBK Models for Regulatory Purposes (Glossy - Mono - Annex IV)

Thirteen case studies (listed in Annex 4)

Case Study I: Generic PBK model for farm animal species: Cattle (*Bos taurus*), Swine (*Sus scrofa*), Sheep (*Ovis aries*) and Chicken (*Gallus gallus domesticus*)

Lautz et al. (2019 a,b; 2020 a,b)

Case Study II: Generic PBK models for four fish species

Grech et al. (2017, 2018 a,b; 2019)

Case Study XIII: Generic Human one compartment and QIVIVE PB-K models

Wiecek et al. (2019 a,b)

Case Study VIII

PBK model application in species and route to route extrapolation

Bessems et al., 2017

Case study IX

Caffeine PBBK model to predict MoIE for risk assessment

IATA caffeine CS

Case study X

IVIVE-PBPK model for phenyl-1,4-dihydropyridine calcium channel antagonists

Gardner et al.

Case XI

Using high-throughput pharmacokinetic simulation and in silico property predictions to predict herbicide absorption and bioavailability

Clark Robert D

Case Study XII

Application of physiologically based kinetic (PBK) modelling in the next generation risk assessment of dermally applied consumer products

Moxon et al. 2020

https://www.oecd.org/chemicalsafety/testing/series-testing-assessment-publications-number.htm

Case Study III

In vitro-to In vivo extrapolation (IVIVE) by PBTK modelling

Fabian et al. 2019

Case Study IV

PBK model predictions using data from analogues

Paini et al., 2021

Case Study V

Physiologically based pharmacokinetic (PBK) model for acrylonitrile in humans

Takano et al 2010

Case Study VI

PBK model predictions for monoisononyl phthalate

Miura et al.,2019

Case Study VII

Quantitative Proteomics-based Bottom-up PBK Modeling to Predict Chemical Exposure in Humans

Chan et al. 2019

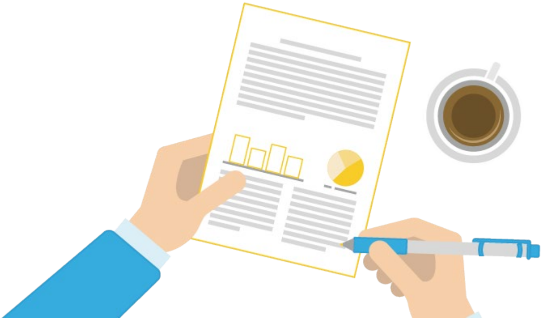

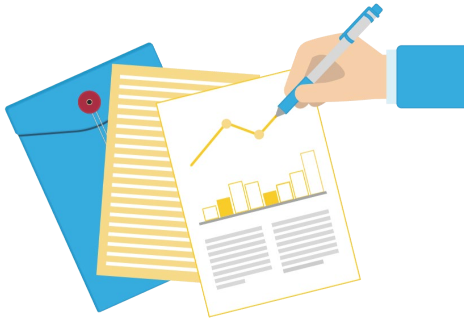

Sources

OECD PBK model GD webinar

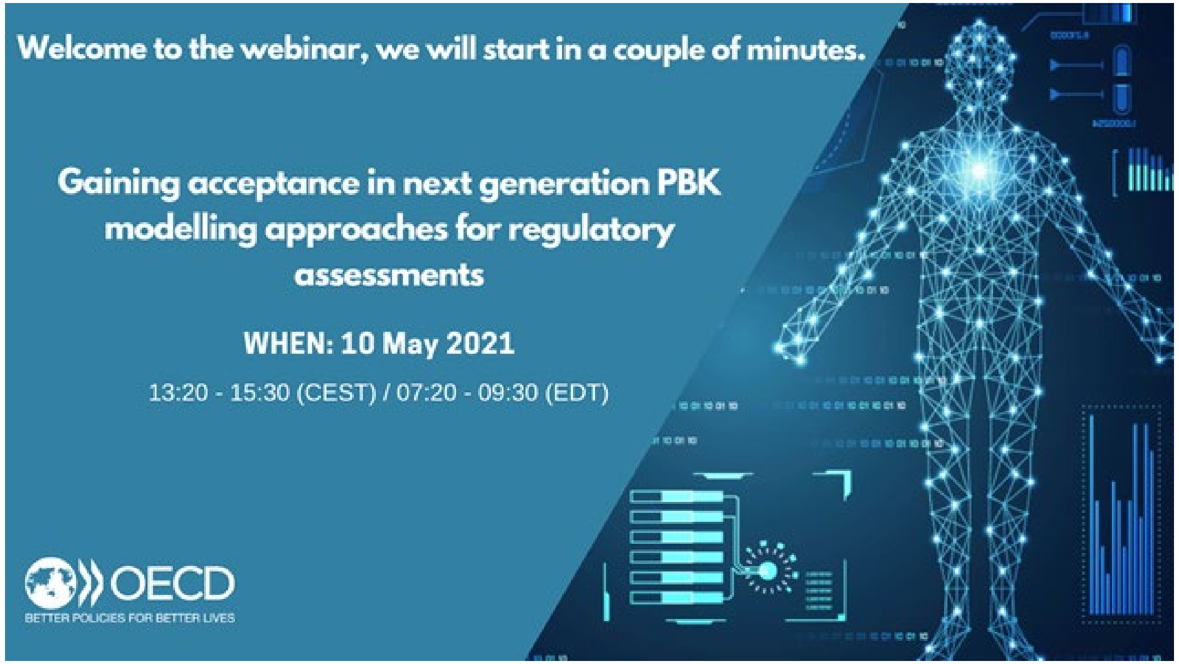

https://www.oecd.org/chemicalsafety/testing/webinars-on-testing-and-assessment-methodologies.htm

OECD PBK model GD (n 331)

Case Studies to illustrate (ANNEX IV)

https://www.oecd.org/chemicalsafety/testing/series-testing-assessment-publications-number.htm

Take Home

- Characterising in vitro and in vivo biokinetics is going to be critical for determining the relevance and context of your results → IVIVE!
- Connected Streams → Integration!
- As the risk assessment community increase its dependence on in vitro systems and NAMs, more PBK models are being developed without the use of in vivo data → Confidence!

Affiliation

Alicia Paini, PhD, ERT

- Scientific project officer at EC Joint Research Centre (April 2012 - September 2021)
- As from 1° of October Senior Scientist, esqLABS GmbH, alicia.paini@esqlabs.com

Inhalation Exposure Modeling for Assessing Health Risks of Toxic Aerosols and Vapors

Richard A. (Rick) Corley, Ph.D.
Greek Creek Toxicokinetics Consulting, LLC
Emeritus Laboratory Fellow, Pacific Northwest National Laboratory
Boise, Idaho
rcorley.gctc@gmail.com

CORESTA 2021
NAM Symposium
19 October 2021

Modeling Examples for Inhaled Aerosols & Vapors

State-of-the-art inhalation modeling approaches for cross-species and *in vitro* to *in vivo* comparisons to assess human health risks

- 3D Imaging-based computational fluid dynamics (CFD) models of the respiratory system
- Incorporate species-specific 3D anatomy, physiology and clearance processes and realistic breathing and exposure scenarios for site-specific dosimetry

- **Ex 1: Ranking relative hazards of tobacco smoke constituents under a harm reduction strategy using existing animal toxicity and measured human exposure data**
  - CFD/PBPK modeling for cell- or tissue-specific internal dose
  - Corley et al., *Toxicol. Sci.* 146(2015)65-88

- **Ex 2: Reducing/replacing animal toxicity studies for pesticide re-registration with *in vitro* toxicity studies with human cells for occupational and residential exposures**
  - CFD/Aerosol/Mucociliary clearance modeling for region-specific retained dose
  - Corley et al., *Toxicol. Sci.* 182(2021)243-259

What is Computational Fluid Dynamics or CFD?

In a nutshell…

- **Numerical method for describing fluid flows**
  - Navier-Stokes Equations that describe the flow of a viscous fluid
  - Solved using a 3D computational mesh with appropriate boundary conditions (e.g. shape, mechanical properties, fluid characteristics, pressure, etc.)
  - The solution is a flow velocity field over space and time
  - Complexities added as needed (equations/mesh refinements) depending upon applications (e.g. physics of heat transfer, turbulence, material transport within fluids, material interactions, etc.)
- **Methods widely used in aerospace, automotive, energy, building HVAC, etc. industries to improve design, trouble-shooting, and decrease costs in product development**

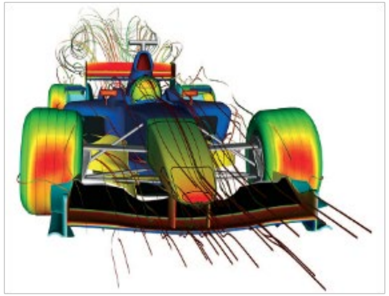
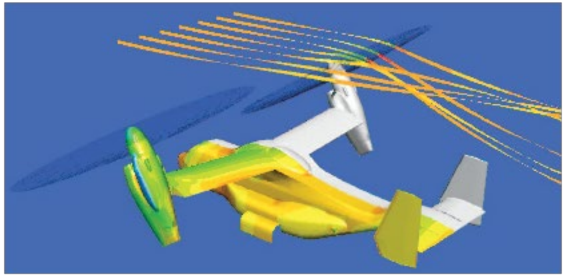
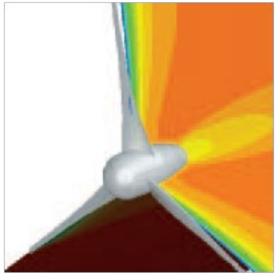

Source: Fluent News, 2005

What is Computational Fluid Dynamics or CFD?

- **Biological applications are a rapidly growing area with the advent of new imaging, image analysis, and computational capabilities**

- **3D/4D MRI and CT**
  - Mod-High resolution
  - Dynamic
  - Structure & Function
- **What once took months, can now be done in days**
- **Personalized models are possible**

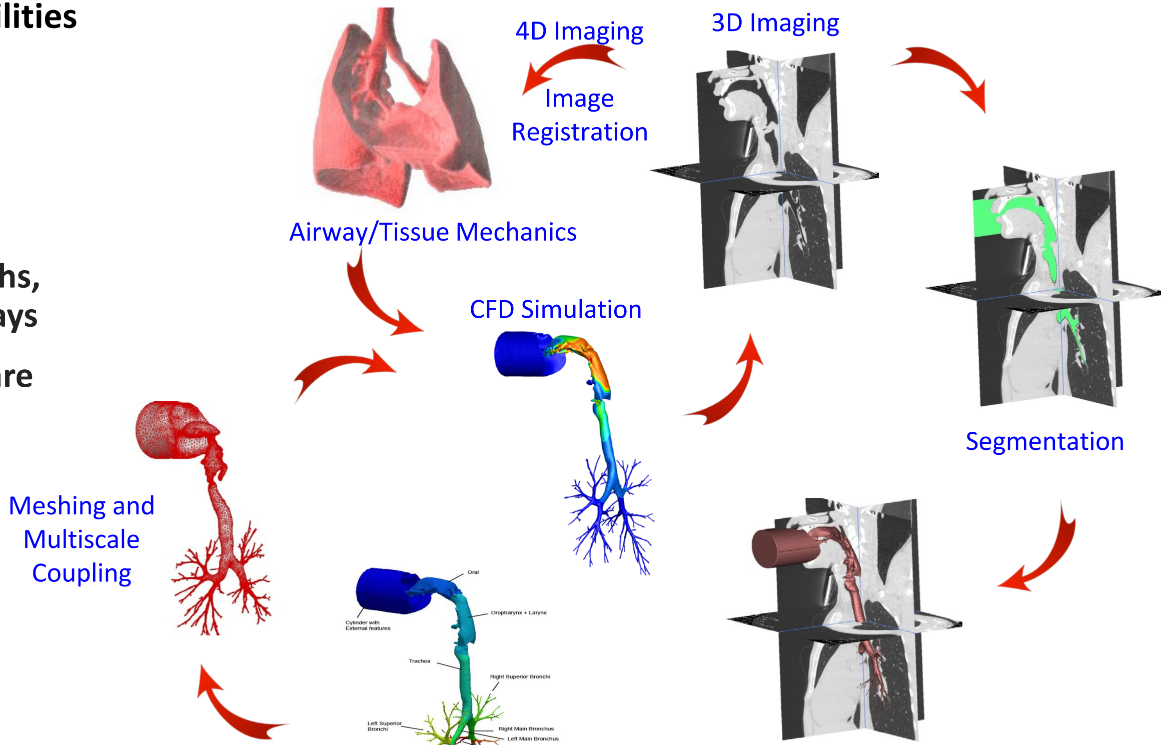

Corley et al. Toxicol. Sci. 128(2012)500-516
Corley et al. Toxicol. Sci. 146(2015)65-88
Jacob et al. Exp. Lung Res. 41(2014)135-145

Ex 1: Multi-Scale CFD/PBPK for Reactive Aldehydes

Formaldehyde 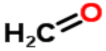

Acetaldehyde 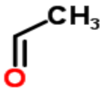

Acrolein 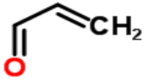

- **Highly reactive, water-soluble vapors**
- **Important industrial chemical intermediates as well as by-products of combustion including smoking of tobacco products**
  - Difficult to directly measure in tissues, endogenously produced and have dietary sources of exposure
- **Cytotoxicity and tumors in specific sites within nasal and upper respiratory tissues of rodents drive many human health risk assessments**
- **Site-specificity of lesions and species differences in anatomy, physiology and tissue clearance rates warranted a combined CFD/PBPK approach**
  - Previous constituent risk comparisons often lacked species-, site-, or exposure-specific dosimetry considerations
- **Took advantage of existing CFD and PBPK models and realistic exposures to create a combined approach**

Ex 1: CFD/PBPK for Reactive Aldehydes

Model Structure

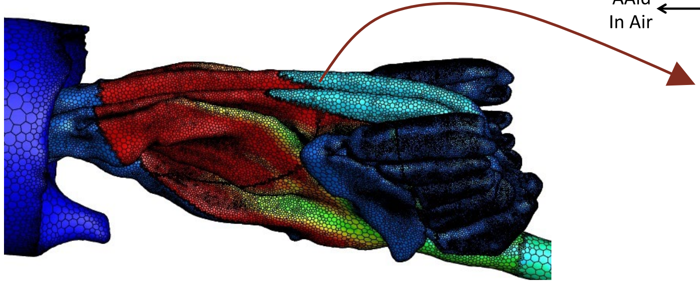

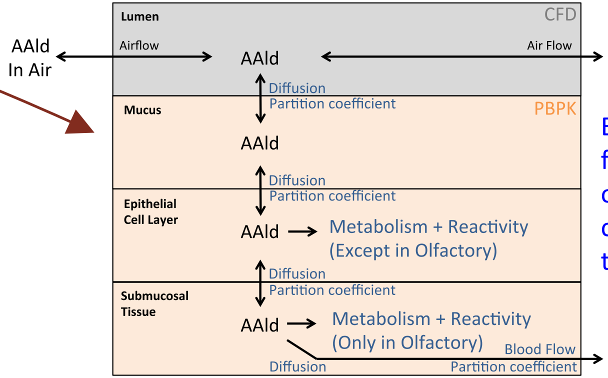

Each surface facet has it's own 2-way coupled PBPK tissue model

Airway surface is annotated by cell type or region to assign appropriate 1D tissue models

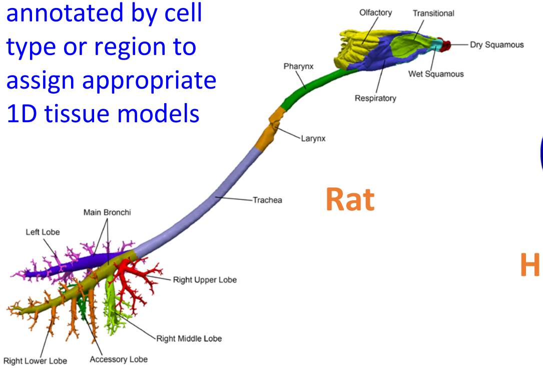

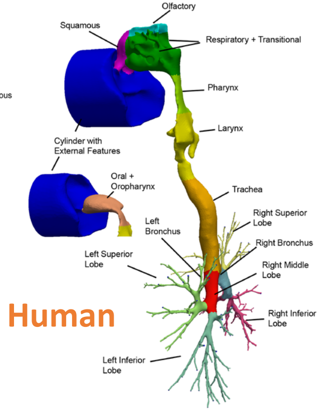

Corley et al. Toxicol. Sci. 128(2012)500-516
Corley et al. Toxicol. Sci. 146(2015)65-88

Ex 1: CFD/PBPK for Reactive Aldehydes

AUC Tissue Concentration “Hot Spots” vs. Lesions

Acetaldehyde

(Rat NOAEL = 50 ppm)

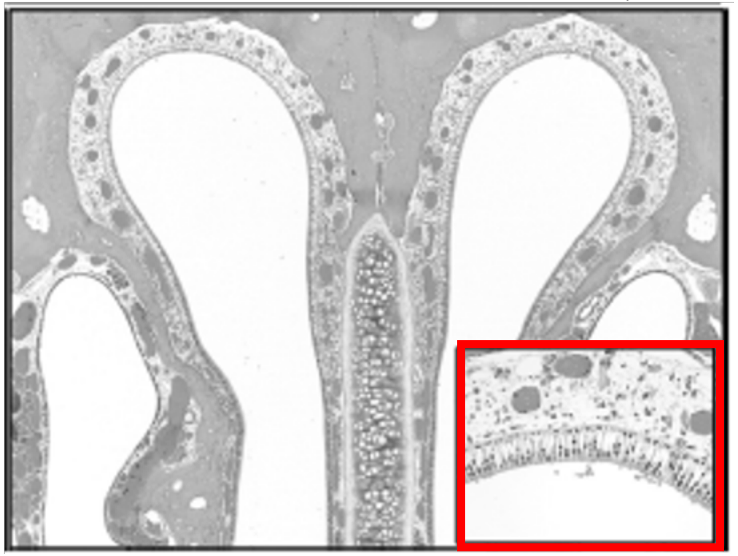

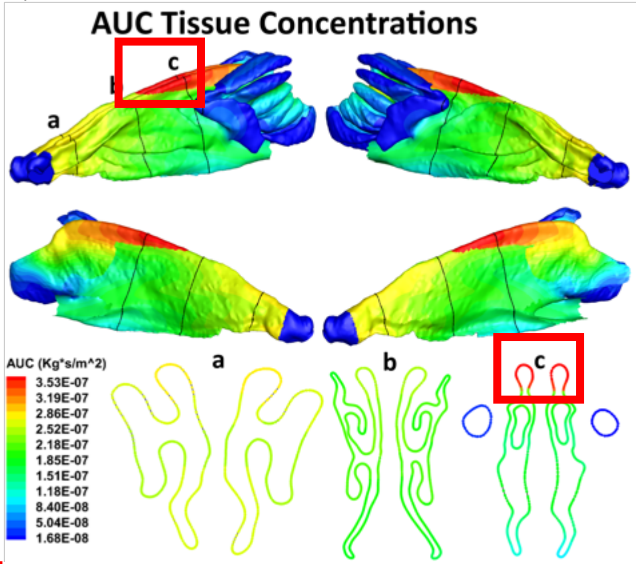

Enzyme Location is Key Determinant

➡AUC dosimetry maps to histopathology

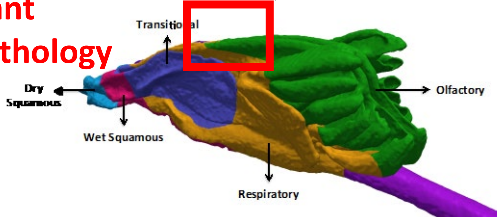

Corley et al. Toxicol. Sci. 146(2015)65-88

Dorman et al. Inhal. Toxicol. 20(2008)245-256

Ex 1: CFD/PBPK for Reactive Aldehydes
Human Exposure via Cigarette Smoking

Human Smoking Profile

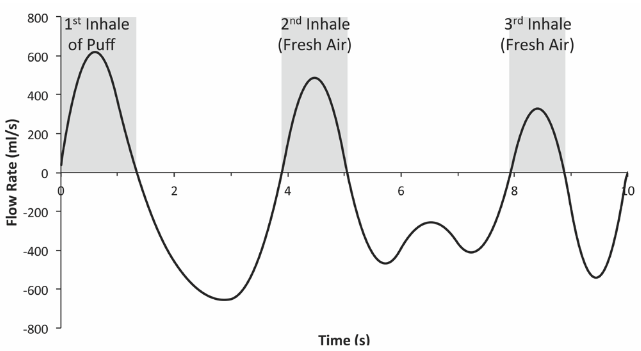

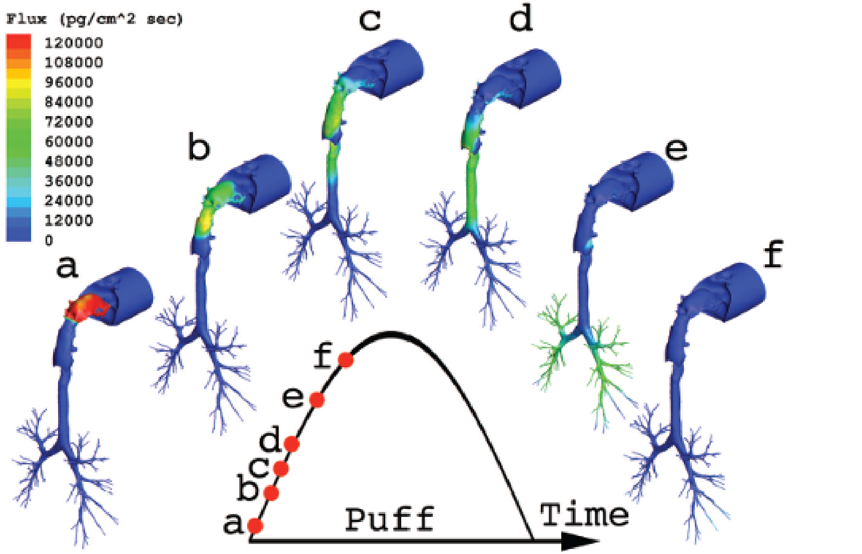

- Measured human puff profile
  - St. Charles et al. Inhal. Toxicol. 21(2009)712-718)
- Measured smoke compositions for representative puff concentrations
  - (Counts et al. Reg. Toxicol. Pharmacol. 41(2005)185-227)
  - Acetaldehyde – 1028 ppm (857 µg/cig)
  - Acrolein – 94 ppm (100 µg/cig)
  - Formaldehyde – 108 ppm (61 µg/cig)

Corley et al. Toxicol. Sci. 146(2015)65-88

Ex 1: CFD/PBPK for Reactive Aldehydes
Comparative Dose Cigarette Smoke Constituents

Acrolein
(Rat NOAEL = 0.2 ppm; Puff = 94 ppm)

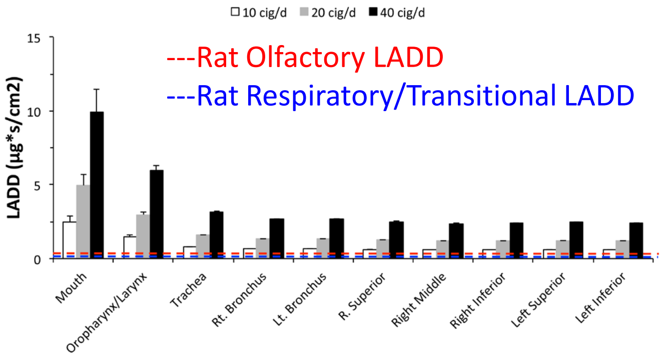

Rat - Human comparisons based upon 'Hot Spot' AUCs and Exposure-Duration/#cigs per day Adjustments

LADD Rat: NOAEL $AUC_{2.5\%}$/breath * bpm * 360 min/d * 5 d/7 d

LADD Human: $AUC_{2.5\%}$/puff * 11 puff/cig * no. cigs/d

Rank order:

Acrolein > Formaldehyde > Acetaldehyde

No significant differences when simulated as a mixture with competitive metabolism

Formaldehyde
(Rat NOAEL = 1 ppm; Puff = 108 ppm)

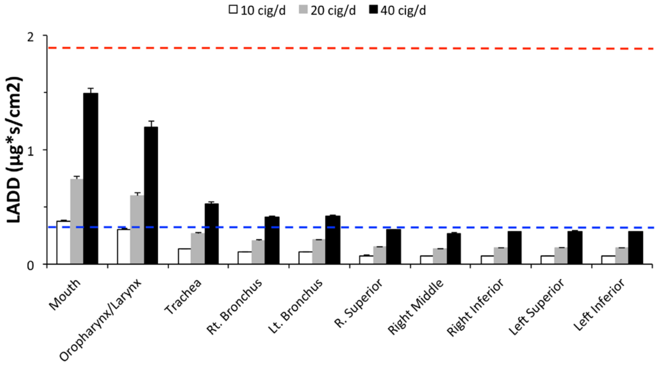

Acetaldehyde
(Rat NOAEL = 50 ppm; Puff = 1024 ppm)

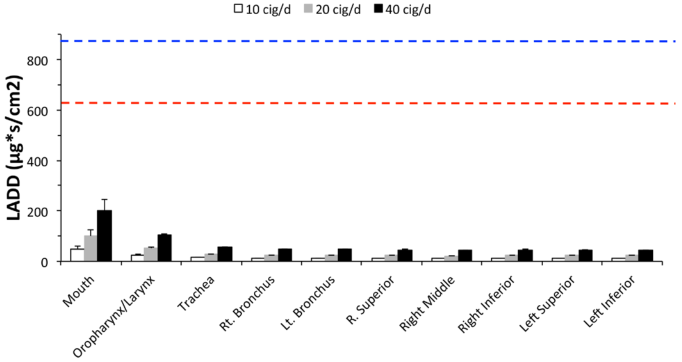

Ex 2: Syngenta's Pesticide Re-Registration
Chlorothalonil

- **A widely-used fungicide since 1966**
  - Labeled for >65 crops
  - Also used as a wood protectant, anti-mold and anti-mildew agent, bacteriocide, microbiocide, algaecide and insecticide
- **Contact irritant by all routes of exposure**
- **Extremely low volatility and water solubility**
  - Formulated as a solid or liquid suspension
  - Applications typically water-diluted spray
- **Aerosol inhalation studies in rats with formulation (acute through 2-week)**
  - Epithelial degeneration/necrosis primarily in nose and larynx; minimal effects in trachea and lung
  - Squamous cell metaplasia in nose and larynx
  - Lesions resolved or reduced following 2-wk recovery

Ex 2: Syngenta's Pesticide Re-Registration
Inhalation Risk Assessment

- **Replace requirement for 90-day rat inhalation toxicity study with *in vitro* studies in human cells coupled to enhanced characterization of exposure and target dose relevant to risk characterization**

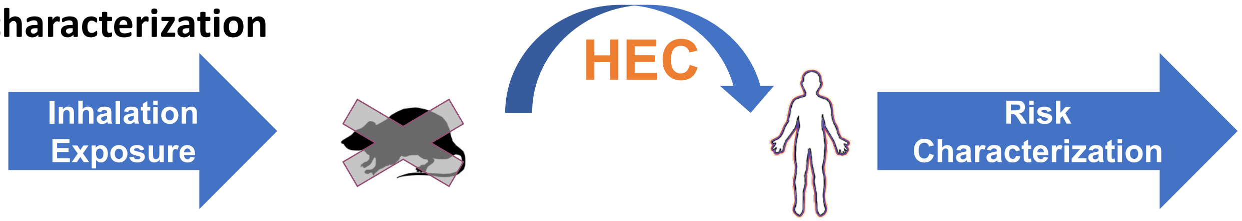

***In vitro* Testing Based Point of Departure using MucilAir™ from Epithelix**

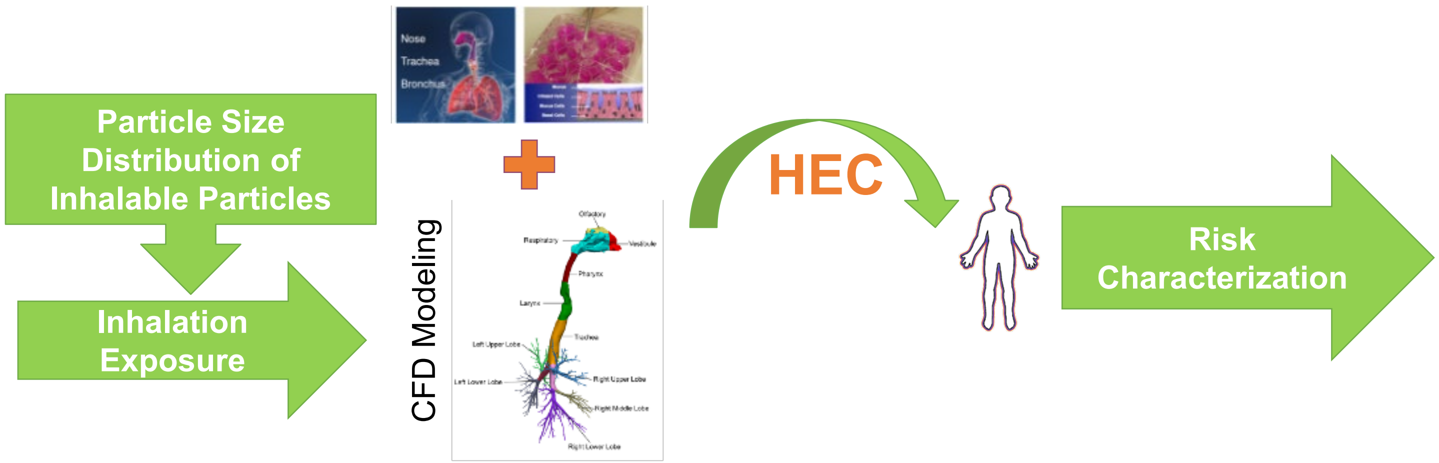

Ex 2: CFD/Particle Dosimetry for Cross-Species and IVIVE

Oral Breathing

58.6% Total Dep
(Nose-Trachea)

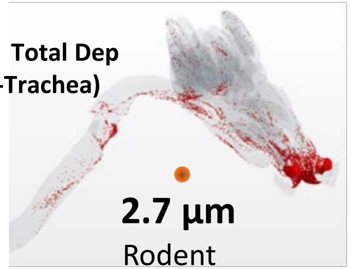

2.7 µm
Rodent
Study

13 µm
Mixer/Loader
Exposure

35 µm
Applicator
Exposure

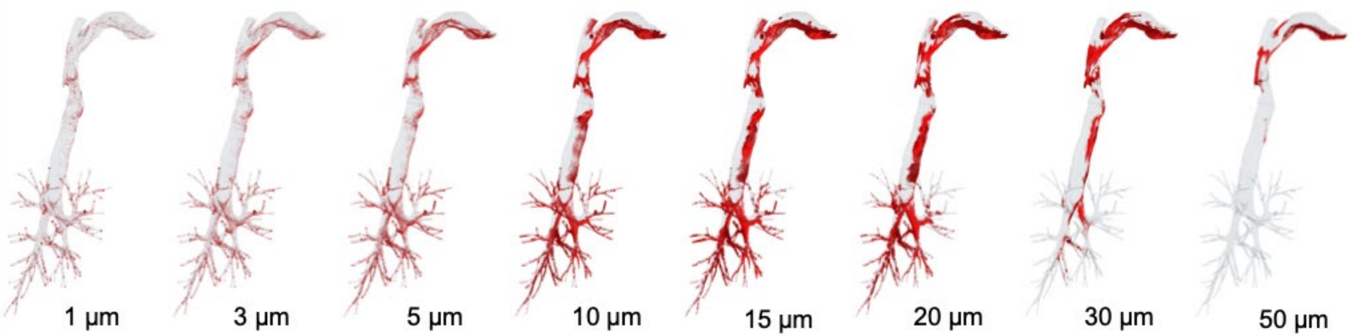

% Total Deposited (Mouth – Bronchi)

1.3	2.0	4.7	31.0	69.1	87.8	96.8	99.5

Ex 2: Clearance Model

Abbreviated ICRP (2015)

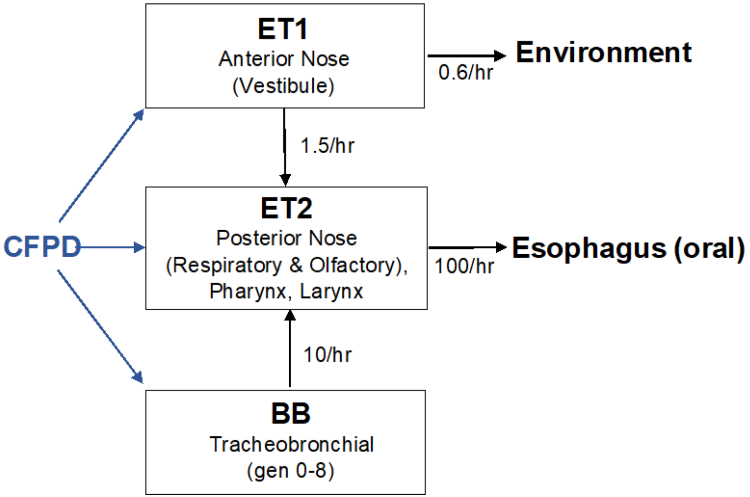

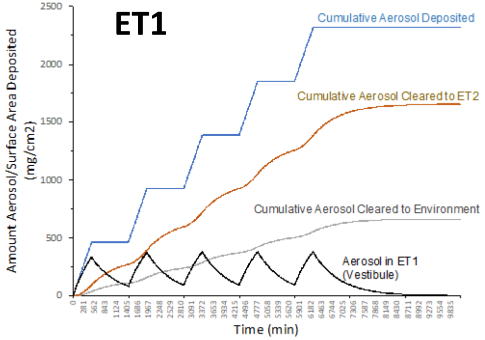

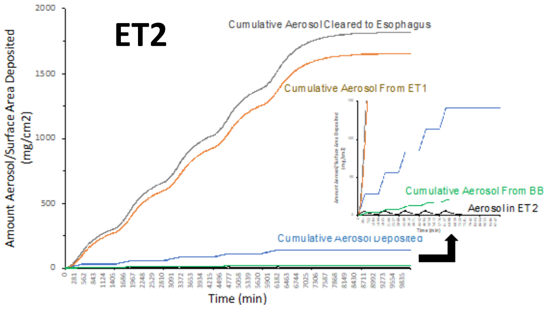

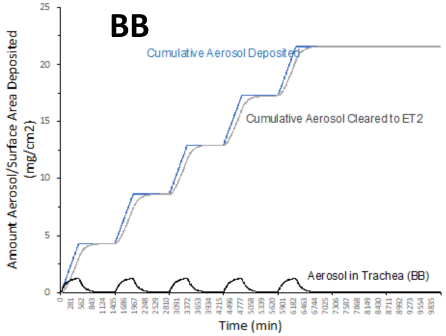

Ex: 10 μm Aerosols
8 hr/d, 5 d/wk

Ex 2: CFPD/CL Human Simulations at Rat LOAEL

AUC Retained Dose vs. In vitro BMDL

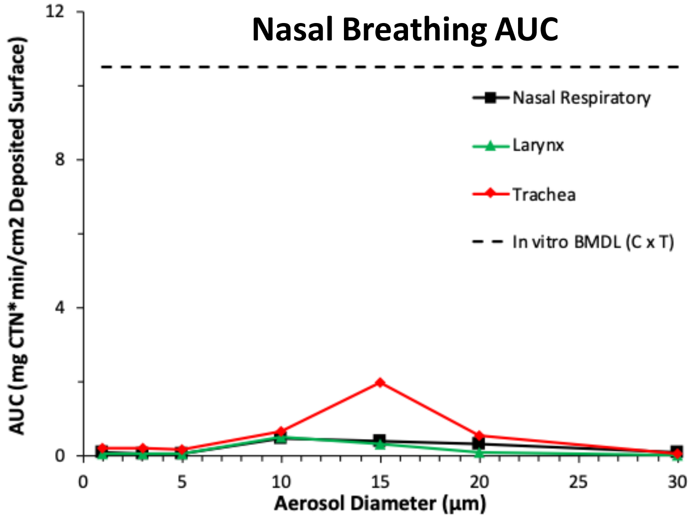

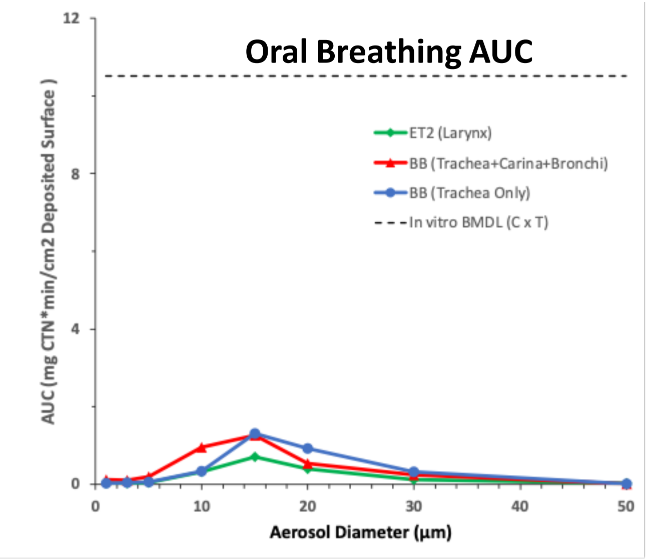

Exposures for 8-hr/day, 5 consecutive days followed by 2 days no exposure

- Day-to-day steady-state retention profile achieved in 2-3 days
- AUC retained doses determined for final exposure day
- AUC compared to BMDL*24 hr (CxT)
- HEC = (BMDL/AUC) * Aerosol Conc * Active Ingredient Conc

Corley et al. Toxicol Sci. 18(2021)243-259

Ex 2: Revised Human Risk Assessment for Inhalation Exposures

- **EPA determined the NAM using human *in vitro* data and CFPD dosimetry was appropriate for evaluating potential risk for inhalation exposure to direct contact irritants**
  - Waved requirement for additional 90-d rat inhalation studies (EPA, 2021).
  - Human equivalent concentrations (HEC) and human equivalent doses (HED) calculated for 2, 8 and 24-hr exposures based upon human *in vitro* BMDL's for multiple polydisperse aerosol scenarios
  - Interspecies UF reduced to 1X (both dosimetry and toxicity determined in human)
  - Intraspecies UF reduced to 3X (ADME not likely an impact for direct contact irritant/cytotoxicant)
  - Revised draft assessment and supporting documents open for comment until Sept. 20, 2021, at: https://www.regulations.gov/docket/EPA-HQ-OPP-2011-0840
    - Manuscripts for the human *in vitro* toxicity study (accepted) and human health risk assessment (in review) have also been submitted

Bottom Lines

- **CFD-based models are well-suited for calculating HEC's from *in vitro* and *in vivo* target tissue doses when site-specificity is important for inhalation toxicity (typically upper conducting airways)**
  - A valuable part of an overall toolkit for modeling inhalation exposures
- **These approaches have been used to refine human risk assessments as well as reduce or even replace animal studies by regulatory agencies**
- **Topics not covered but still important include:**
  - Model evaluation and verification/validation were key components to both examples
    - See references included at the end of this presentation including those used in the case studies
  - Models can be templated or adjusted to fit new materials or exposure scenarios (no need to start from scratch)
    - Airway geometries available for multiple humans and animal models (see Selected References)
    - Existing CFPD simulations are being used to predict site-specific doses for other aerosols that have similar properties
  - CFD models are ideal for site-specificity in upper conducing airways (nose/mouth to generation 5-10) but do not describe the deep lung due to limitations in imaging and the computational challenges
    - However, the Multiple Path Particle Dosimetry (MPPD) model is ideal for predicting regional dosimetry in the deep lung and is now being adopted by the U.S.EPA to replace its RDDR model
      - MPPD is available (free) at: https://www.ara.com/mppd/
    - CFPD models have also been linked with the MPPD model to provide full respiratory system coverage (Kuprat et al., *J. Aerosol Sci.* 151(2021)105647) and take advantage of, and compensate for, the strengths and weaknesses of each model
      - Ongoing work: disease influences on tissue mechanics are now being incorporated into the CFPD/MPPD model and validated against experimental data in humans and rats

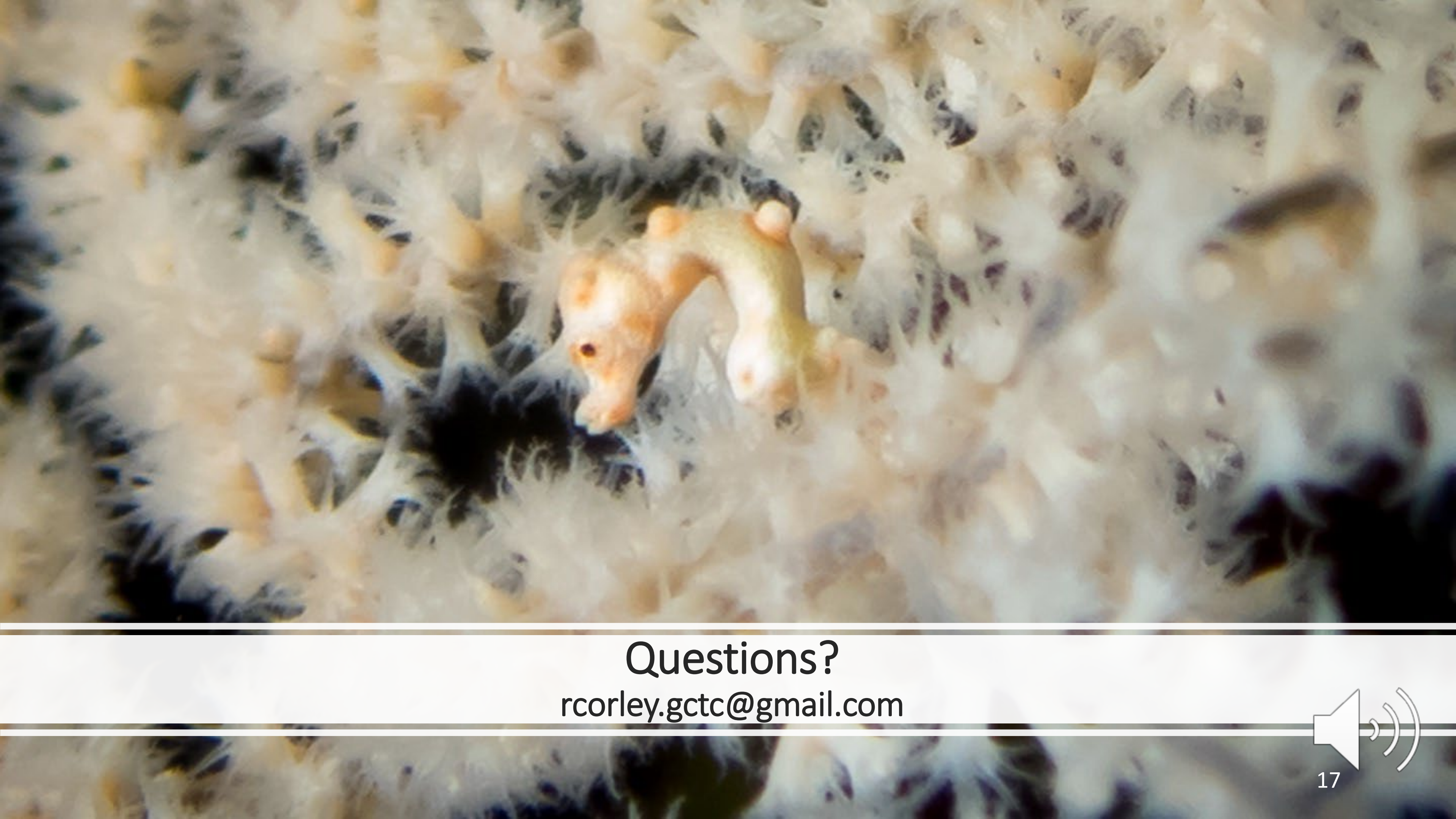
Questions?
rcorley.gctc@gmail.com

Acknowledgments

- **Pacific Northwest National Laboratory (PNNL)**
  - Andrew Kuprat, Sarah Suffield, Senthil Kabilan (*Takeda), Kevin Minard (*Spintronics), Rick Jacob, Sean Colby, Dan Einstein (*St. Martin Univ.), James Carson (*Univ. Texas)
- **University of Washington**
  - Robb Glenny
- **UCSD**
  - Chantal Darquenne
- **Syngenta**
  - Tharacad S. (Rama) Ramanarayanan, Paul Hinderliter (*Axcella), Doug Wolf
- **Battelle Memorial Institute**
  - Kevin Yugulis
- **ARA**
  - Bahman Asgharian, Owen Price, Jeff Schroeter

- **Presented work funded by**
  - NHLBI, NIEHS, Battelle, RJR, DOE, Syngenta

Glossary

- **AUC = Area under the curve, typically of a concentration vs. time curve**
- **CFD = Computational fluid dynamics**
- **CFPD = Computational fluid-particle dynamics**
- **Cmax = Maximum concentration, typically of a concentration vs. time curve**
- **CT = X-ray computed tomography**
- **EPA = U.S. Environmental Protection Agency**
- **FIFRA SAP = Federal Insecticide, Fungicide, and Rodenticide Act Scientific Advisory Panel to EPA**
- **HEC = Human equivalent concentration (typically mg/L or mg/m^3)**
- **HED = Human equivalent dose (typically mg/kg/d)**
- **HVAC = Heating, ventilation, air conditioning**
- **ICRP = International Commission on Radiological Protection**
- **MMAD = Mass median aerodynamic diameter**
- **MPPD = Multiple path particle dosimetry model**
- **MRI = Magnetic resonance imaging**
- **NCRP = National Council on Radiation Protection**
- **PBPK = Physiologically based pharmacokinetic model**

Selected References

- Applied Computational Fluid Dynamics. (2005). Fluent News. Marshall, L., Ed. Vol. 14, Issue 2.
- Ardini-Poleske, M.E., Clark, R.F., Ansong, C., Carson, J.P., Corley, R.A., Deutsch, G.H., Hagood, J.S., Kaminski, N., Mariani, T.J., Potter, S.S., Pryhuber, G.S., Warburton, D., Whitsett, J.A., Palmer, S.M., Ambalavanan, N., LungMAP Consortium. (2017). LungMAP: The molecular atlas of lung development program. Amer. J. Phys. Lung Cell. Mol. Physiol. 313, 733-740.
- Behrsing, H., Hill, E., Raabe, H., Tice, R., Fitzpatrick, S., Devlin, R., Pinkerton, K., Oberdorster, G., Wright, C., Wieczorek, R., Aufderheide, M., Steiner, S., Krebs, T., Asgharian, B., Corley, R., Oldham, M., Adamson, J., Li, X., Rahman, I., Grego, S., Chu, P.H., McCullough, S., and Curren, R. (2017). In Vitro Exposure Systems and Dosimetry Assessment Tools for Inhaled Tobacco Products: Workshop Proceedings, Conclusions and Paths Forward for In Vitro Model Use. ATLA 45, 117-158.
- Colby, S.M., Kabilan, S., Jacob, R.E., Kuprat, A.P., Einstein, D.R., Minard, K.R., and Corley, R.A. (2016). Comparison of realistic and idealized breathing patterns in computational models of airflow and vapor dosimetry in the rodent upper respiratory tract. Inhal. Toxicol. 28(4), 192-202.
- Corley, R.A., Kabilan, S., Kuprat, A.P., Carson, J.P., Jacob, R.E., Minard, K.R., Teeguarden, J.G., Timchalk, C., Pipavath, S., Glenny, R., and Einstein, D.R. (2015). Comparative risks of aldehyde constituents in cigarette smoke using transient computational fluid dynamics/physiologically based pharmacokinetic models of the rat and human respiratory tracts. Toxicol. Sci., 146(1), 65-88.
- Corley, R.A., Kabilan, S., Kuprat, A.P., Carson, J.P., Minard, K.R., Jacob, R.E., Timchalk, C., Glenny, R., Pipavath, S., Cox, T., Wallis, C., Larson, R.F., Fanucchi, M.V., Postlethwait, E., and Einstein, D.R. (2012). Comparative computational modeling of airflows and vapor dosimetry in the respiratory tracts of rat, monkey, and human. Toxicol. Sci., 128(2), 500-516.
- Corley, R.A., Kuprat, A.P., Suffield, S., Kabilan, S., and Hinderliter, P., Yugulis, K. and Ramanarayanan, T.S. (2021). New approach methodology for assessing inhalation risks of a contanct respiratory cytotoxicant: computational fluid-particle dynamic-based aerosol dosimetry modeling for cross-species and in vitro comparisons. Toxicol. Sci. 182(2), 243-259.
- Counts, M. E., Morton, M. J., Laffoon, S. W., Cox, R. H., Lipowicz, P.J. (2005). Smoke composition and predicting relationships for international commercial cigarettes smoked with three machine-smoking conditions. Regul. Toxicol. Pharmacol., 41,185–227.
- Dorman, D. C., Struve, M. F., Wong, B. A., Gross, E. A., Parkinson, C., Willson, G. A., Tan, Y. M., Campbell, J. L., Teeguarden, J. G., Clewell, H. J., 3rd, et al. (2008a). Derivation of an inhalation reference concentration based upon olfactory neuronal loss in male rats following subchronic acetaldehyde inhalation. Inhal. Toxicol., 20, 245–256.
- EPA (U.S. Environmental Protection Agency), 2016. Process for evaluating & implementing alternative approaches to traditional in vivo acute toxicity studies for FIFRA regulatory use. Office of Pesticide Programs, Washington, DC. February 4, 2016.
- EPA (U.S. Environmental Protection Agency), 2018. Strategic plan to promote the development and implementation of alternative test methods within the TSCA program. EPA-740-R1-8004. June 22, 2018.

Selected References (continued)

- ICRP (International Commission on Radiological Protection), 2015. Occupational intakes of radionuclides: Part 1. ICRP Publication 130. Ann. ICRP 44(2). Sage.
- Jacob, R.E., Lamm, W.J., Einstein, D.R., Krueger, M.A., Glenny, R.W. and Corley, R.A. (2014). Comparison of CT-derived ventilation maps with deposition patterns of inhaled microspheres in rats. Exp. Lung Res. 41, 135-145.
- Kabilan, S., Suffield, S.R., Recknagle, K.P., Jacob, R.E., Einstein, D.R., Kuprat, A.P., Carson, J.P., Colby, S.M., Saunders, J.H., Hines, S.A., Teeguarden, J.G., Straub, T.M., Moe, M., Taft, S.C. and Corley, R.A. (2016). Computational fluid dynamics modeling of Bacillus anthracis spore deposition in rabbit and human respiratory airways. J. Aerosol Sci., 99, 64-77.
- Kimbell, J.S., Godo, M.N., Gross, E.A., Joyner, D.R., Richardson, R.B., and Morgan, K.T. (1997). Computer simulation of inspiratory airflow in all regions of the F344 rat nasal passages. Toxicol. Appl. Pharmacol. 145, 388-398.
- Kimbell, J.S., Subramaniam, R.P., Gross, E.A., Schlosser, P.M. and Morgan, K.T. (2001). Dosimetry modeling of inhaled formaldehyde: comparisons of local flux predictions in the rat, monkey, and human nasal passages. Toxicol. Sci. 64, 100-110.
- Kolanjiyil, A. and Kleinstreuer, C., 2016. Computationally efficient analysis of particle transport and deposition in a human whole-lung airway model. Part 1: theory and model validation. Computers Biol. Med. 79, 193-204.
- Kolanjiyil, A. and Kleinstreuer, C., 2017. Computational analysis of aerosol-dynamics in a human whole-lung airway model. J. Aerosol Sci. 114, 301-316.
- Kuprat, A.P., Jalali, M., Jan, T., Corley, R.A., Asgharian, B., Price, O., Singh, R.K., Colby, S., and Darquenne, C. (2021). Efficient bi-directional coupling of 3D Computational Fluid-Particle Dynamics and 1D Multiple Path Particle Dosimetry lung models for multiscale modeling of aerosol dosimetry. J. Aerosol Sci. 151, 105647
- Longest, P.W., Bass, K., Dutta, R., Rani, V., Thomas, M.L., El-Achwah, A. and Hindle, M. (2019) Use of computational fluid dynamics deposition modeling in respiratory drug delivery, Expert Opinion on Drug Delivery, 16:1, 7-26.
- Longest, P. W., and Holbrook, L. T. (2011). In silico models of aerosol delivery to the respiratory tract - Development and applications. Adv. Drug Deliv. Rev. 64, 296–311.
- Longest, P.W., Tian, G., Walenga, R.L. & Hindle, M. (2012). Comparing MDI and DPI aerosol deposition using in vitro experiments and a new stochastic individual path (SIP) model of the conducting airways. Pharm. Res. 29, 1670–1688.
- Lucci, F., Castro, N., Rostami, A., Oldham, M., Hoeng, J., Pithawalla, Y., Kuczaj, A. (2018). Characterization and modeling of aerosol deposition in Vitrocell® exposure systems - exposure well chamber deposition efficiency. J. Aerosol Sci. 123, 141-160.
- Martonen, T. B., and Schroeter, J. D. (2003). Risk assessment dosimetry model for inhaled particulate matter: I. Human subjects. Toxicol. Lett. 138, 119–132.
- Martonen, T. B., and Schroeter, J. D. (2003). Risk assessment dosimetry model for inhaled particulate matter: II. Laboratory surrogates (rat). Toxicol. Lett. 138, 133–142.

Selected References (continued)

- Minard, K. R., Einstein, D. R., Jacob, R. E., Kabilan, S., Kuprat, A. P., Timchalk, C. A., Trease, L. L., and Corley, R. A. (2006). Application of magnetic resonance (MR) imaging for the development and validation of computational fluid dynamic (CFD) models of the rat respiratory system. Inhal. Toxicol. 18, 787–794.
- National Academy of Sciences (NAS). (2007). Toxicity testing in the 21st century: a vision and a strategy. National Academy Press. Washington, D.C.
- National Academy of Sciences (NAS). (2012). Exposure science in the 21st century: a vision and a strategy. National Academy Press. Washington, D.C.
- NIH/NHLBI LungMAP Consortium: Molecular and Cellular Atlas of Lung Development. www.lungmap.net.
- Schroeter, J.D., Kimbell, J.S., Gross, E.A., Willson, G.A., Dorman, D.C., Tan, Y.M., and Clewell, H.J. (2008). Application of physiological computational fluid dynamics models to predict interspecies nasal dosimetry of inhaled acrolein. Inhal. Toxicol. 20, 227-243.
- St Charles, F. K., Krautter, G. R., and Mariner, D. C. (2009). Postpuff respiration measures on smokers of different tar yield cigarettes. Inhal. Toxicol., 21, 712–718
- Walenga, R.L., Babiskin, A.H., and Zhao, L. 2019. In silico methods for development of generic drug-device combination orally inhaled drug products. CPT Pharmacometrics Syst. Pharmacol. 8, 359-370.
- Xi, J., Kim, J.W., Si, X.A., Corley, R.A., and Zhou, Y. (2016). Modeling of inertial depositions in scaled models of rat and human nasal airways: Towards in vitro regional dosimetry in small animals. J. Aerosol. Sci. 99, 78-93.
- Yin, Y., Choi, J., Hoffman, E.A., Tawhai, M.H., and Lin C.L., 2010. Simulation of pulmonary air flow with a subject-specific boundary condition. J. Biomech. 43, 2159-2163.
- Yin, Y., Choi, J., Hoffman, E.A., Tawhai, M.H., and Lin C.L., 2013. A multiscale MDCT image-based breathing lung model with time-varying regional ventilation. J. Comp. Phys. 244, 168-192.

- **NAM Links:**
  - EPA: https://www.epa.gov/research/epa-new-approach-methods-efforts-reduce-use-animals-chemical-testing
  - FDA: https://www.fda.gov/news-events/fda-brief/fda-brief-fda-publishes-report-advancing-alternative-methods

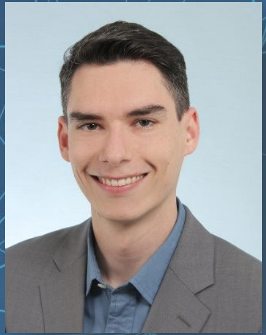

Assessing Respiratory Toxicity of Chemicals in Two Human *in vitro* Systems

Andreas Stucki, PhD
PETA Science Consortium International e.V.

CORESTA SSPT – NAM Symposium
19 October 2021

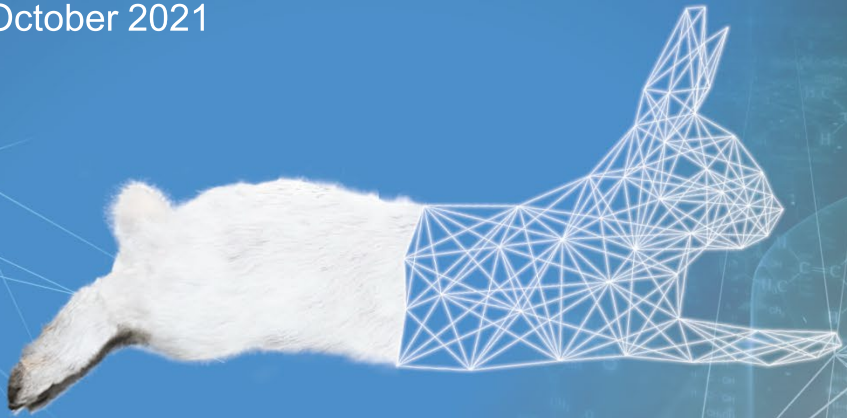

AndreasS@thepsci.eu
www.thePSCI.eu

PETA SCIENCE CONSORTIUM INTERNATIONAL e.V.

FUNDING

TRAINING

WORKSHOPS AND WEBINARS

PUBLICATIONS AND PRESENTATIONS

RETROSPECTIVE REVIEWS

The human lungs

- 100-150 m^2 surface area
- <1 µm air-blood barrier thickness
- 7-10,000 km of blood vessels
- 17,000 breaths per day
- 7,000 L of air per day
- 40+ different cell types

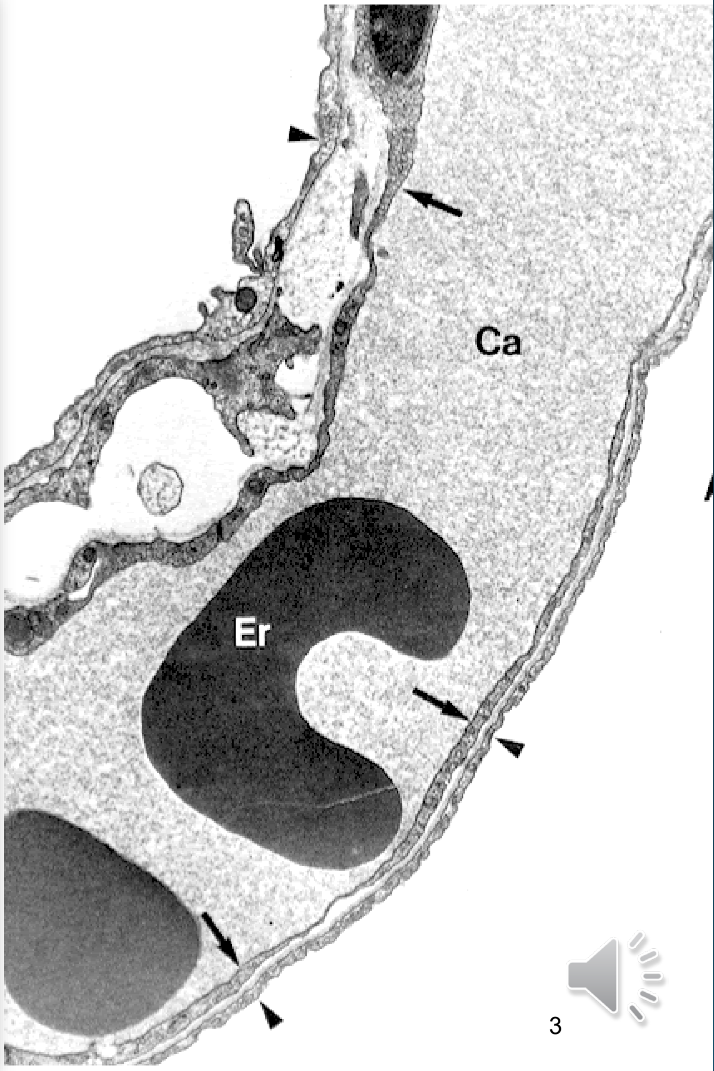

Anatomical and physiological differences

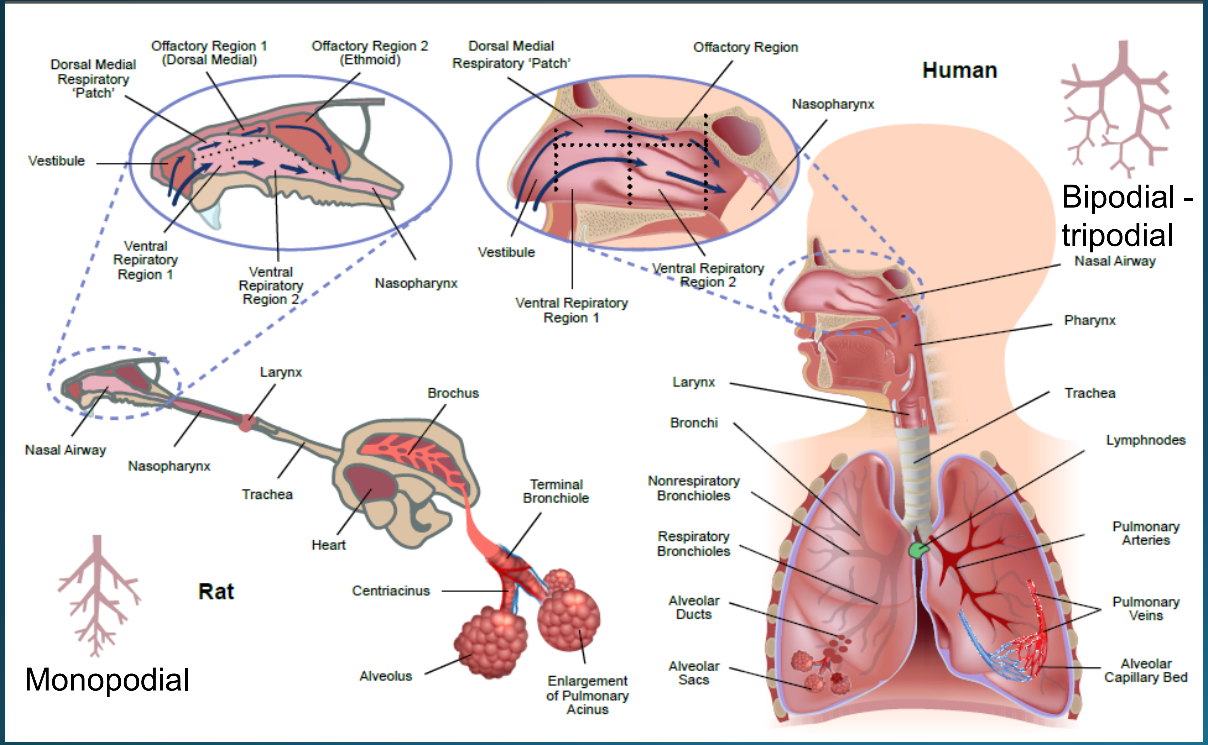

Illustration modified from Dr. Jack R. Harkema,
Professor of Comparative Pathology, Michigan State University

- Ventilation rates and breathing mode
- Airway architecture and branching pattern
- Cell type distribution and mucous composition
- Metabolic activity

The INSPiRE Initiative:
IN vitro System to Predict REspiratory toxicity

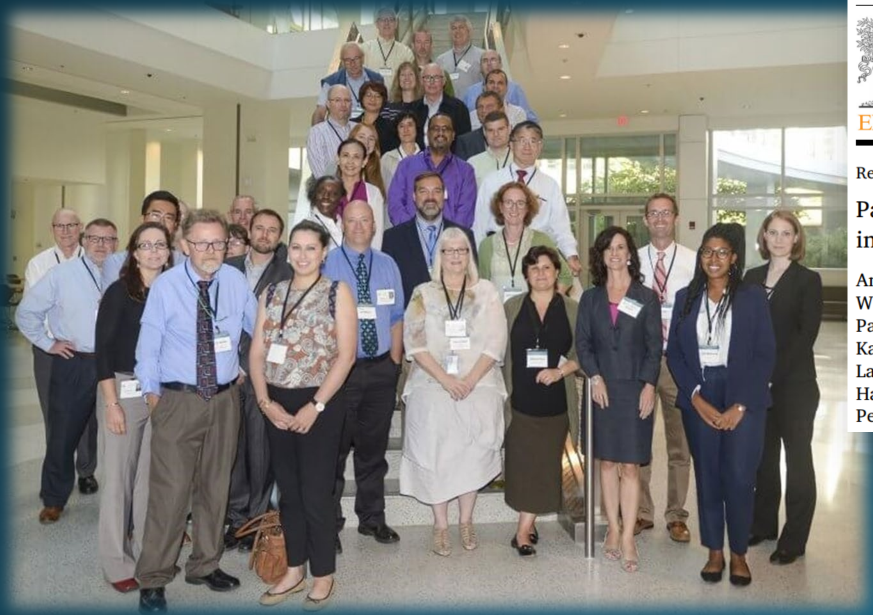

Toxicology in Vitro 52 (2018) 131–145

Contents lists available at ScienceDirect

Toxicology in Vitro

journal homepage: www.elsevier.com/locate/toxinvit

ELSEVIER

Review

Pathway-based predictive approaches for non-animal assessment of acute inhalation toxicity

Amy J. Clippinger[a,*], David Allen[b], Holger Behrsing[c], Kelly A. BéruBé[d], Michael B. Bolger[e], Warren Casey[f], Michael DeLorme[g], Marianna Gaça[h], Sean C. Gehen[i], Kyle Glover[j], Patrick Hayden[k], Paul Hinderliter[l], Jon A. Hotchkiss[m], Anita Iskandar[n], Brian Keyser[o], Karsta Luettich[n] [illegible]
Lawrence Milch[illegible]
Hans Raabe[c], En[illegible]
Peter S. Thorne[x] [illegible]

Contents lists available at ScienceDirect

Toxicology in Vitro

journal homepage: www.elsevier.com/locate/toxinvit

ELSEVIER

Alternative approaches for acute inhalation toxicity testing to address global regulatory and non-regulatory data requirements: An international workshop report

Amy J. Clippinger[a,*], David Allen[b], Annie M. Jarabek[c], Marco Corvaro[d], Marianna Gaça[e], Sean Gehen[f], Jon A. Hotchkiss[g], Grace Patlewicz[h], Jodie Melbourne[a], Paul Hinderliter[i], Miyoung Yoon[j], Dongeun Huh[k], Anna Lowit[l], Barbara Buckley[c], Michael Bartels[m], Kelly BéruBé[n], Daniel M. Wilson[g], Ian Indans[o], Mathieu Vinken[p]

vito

PETA SCIENCE CONSORTIUM INTERNATIONAL e.V.

Goals of the INSPiRE initiative

- Present a case study on how *in vitro* approaches may be used for acute toxicity testing
- Compare a 2D cell line with 3D human reconstructed lung tissues
- Derive *in vitro* point of departure (POD)
- Strengthen scientific confidence in *in vitro* models *en lieu* of animal testing

Initial considerations for human *in vitro* respiratory toxicity testing

CHEMICAL OR SUBSTANCE TO TEST?

WHAT EXPOSURE SYSTEM?

WHICH IN VITRO/EX VIVO SYSTEM?

WHAT KIND OF CELLS?

WHAT ENDPOINTS/ READOUTS?

Picking a chemical or substance to test?

- Know your substance:
  - Physicochemical properties of the substance?
  - Locally metabolized?
  - *in vitro* or *in vivo* data available?
  - Known Adverse Outcome Pathways?

Silanes

– Highly reactive
– Triethoxysilane more stable

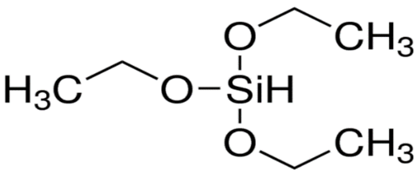

Triethoxysilane
(GHS 2, CAS # 998-30-1)

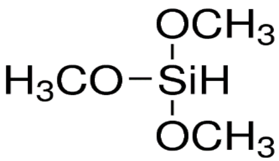

Trimethoxysilane
(GHS 1, CAS# 2487-90-3)

What exposure system to use?

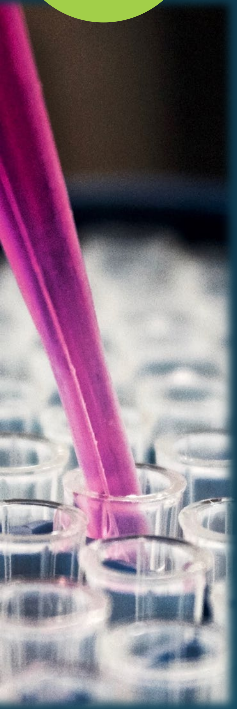

**Pipetting**		**ALI exposure**
Easier to calculate exposure dose	+	Physiologically relevant
No special equipment needed		Final formulation can be used
May disturb surface lining fluid	–	Special equipment needed
Limited to (particles in) liquids		Monitoring exposure dose more challenging

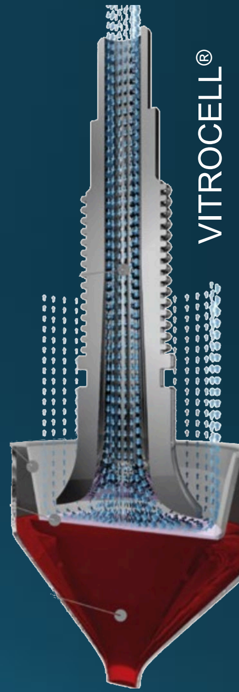

Petersen EJ, Sharma M, Clippinger AJ, et al. Use of Cause-and-Effect Analysis to Optimize the Reliability of *In Vitro* Inhalation Toxicity Measurements Using an Air-Liquid Interface. doi:10.1021/acs.chemrestox.1c00080

Which *in vivo* / *ex vivo* system to use?

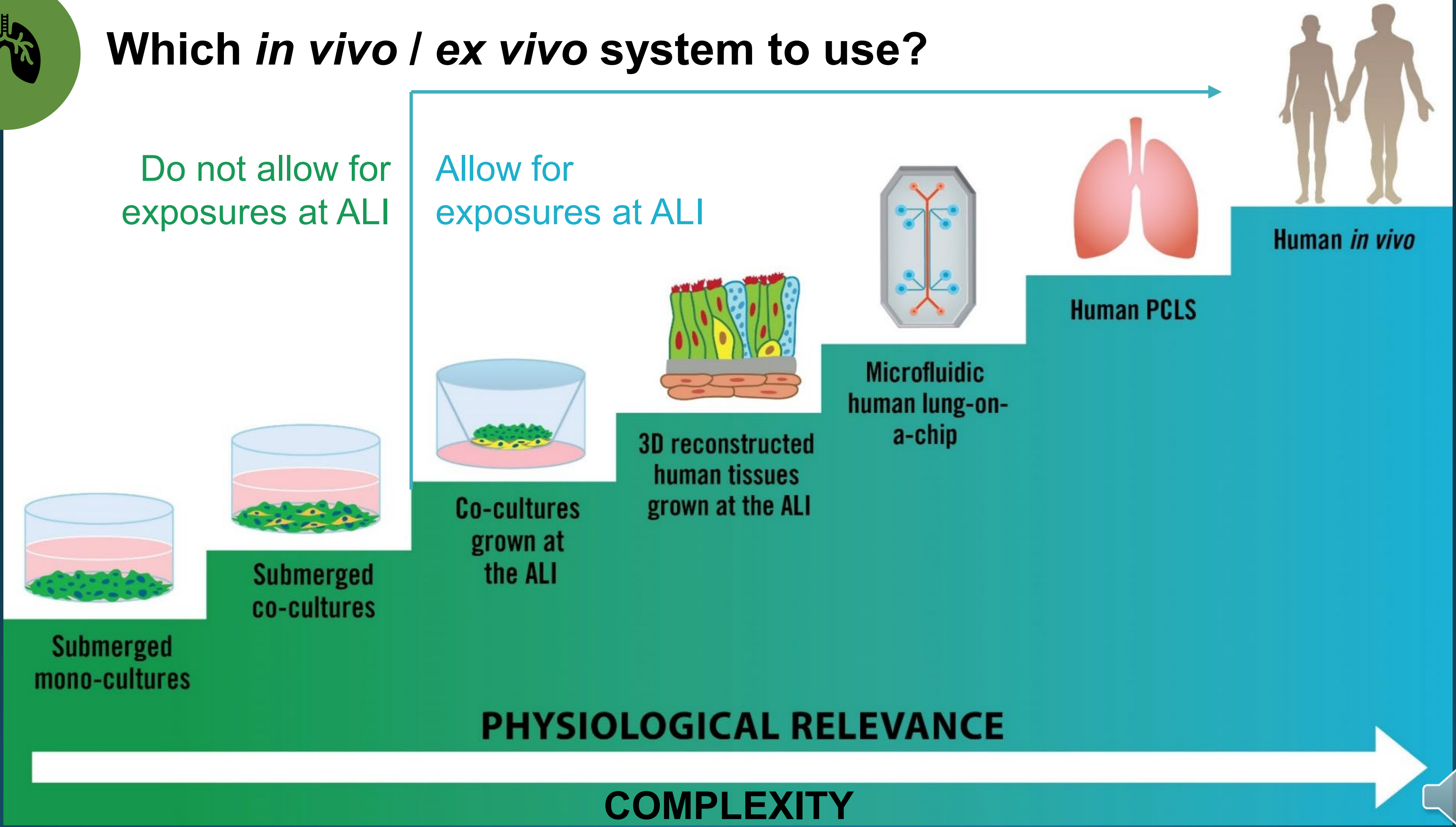

Clippinger et al 2018

Human precision-cut lung slices

- PCLS from healthy and diseased donors
- All relevant cells and structures present
- Culture for 28+ days & cryopreservation possible

- Cross-section
- Multiple cell types may make readout more challenging
- Obtainment of (suitable) donor tissues

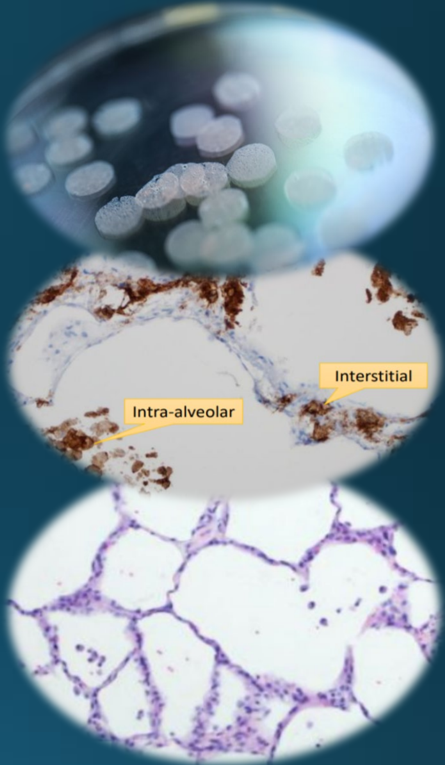

Microphysiological Systems / Organs-on-chip

+

- Influencing microenvironment of tissues/cells
- Mechanical stretch or „blood“ flow possible
- Allow combination of different tissues (e.g., Lung-Liver)

–

- Various materials used → may influence dose (e.g. absorption)
- Choice of cell culture medium for multiple “organs”
- Standardisation and comparability difficult

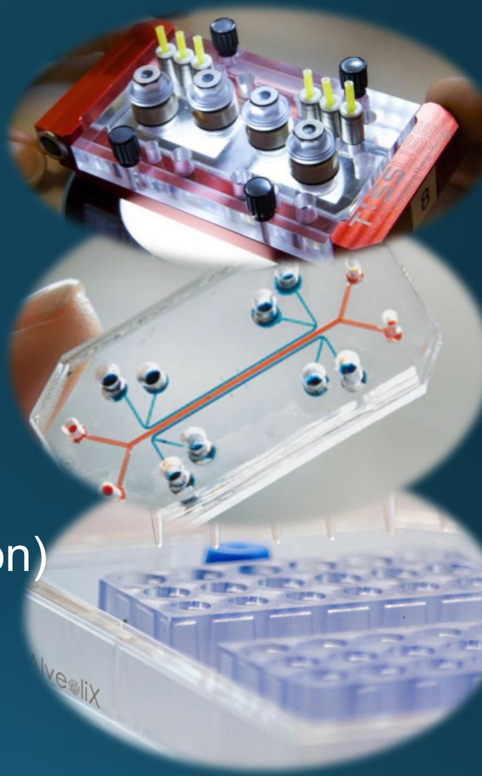

www.thepsci.eu/chips

2D monocultures vs reconstructed tissues

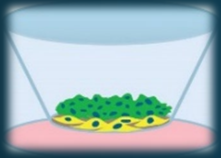

**2D monocultures**		**3D reconstructed tissues**
Simpler & less expensive	+	Primary cells differentiated to *in situ*-like epithelium
Higher throughput		ALI cultures for months
Cell lines and their limitations	–	Generally more expensive
Often short-term ALI cultures		

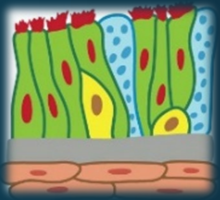

Petersen EJ, Sharma M, Clippinger AJ, et al. Use of Cause-and-Effect Analysis to Optimize the Reliability of *In Vitro* Inhalation Toxicity Measurements Using an Air-Liquid Interface. doi:10.1021/acs.chemrestox.1c00080

What kind of cells to use?

Respiratory tract

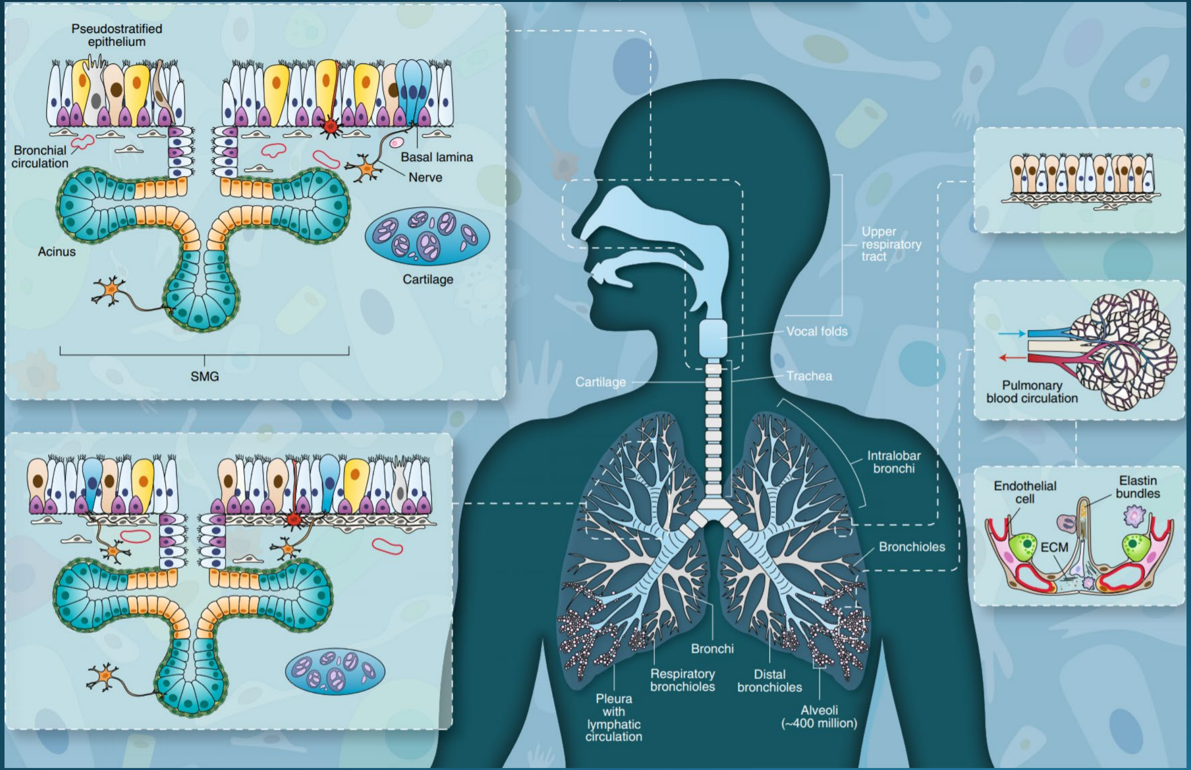

https://www.nature.com/articles/s41556-019-0357-7.pdf

Cell types

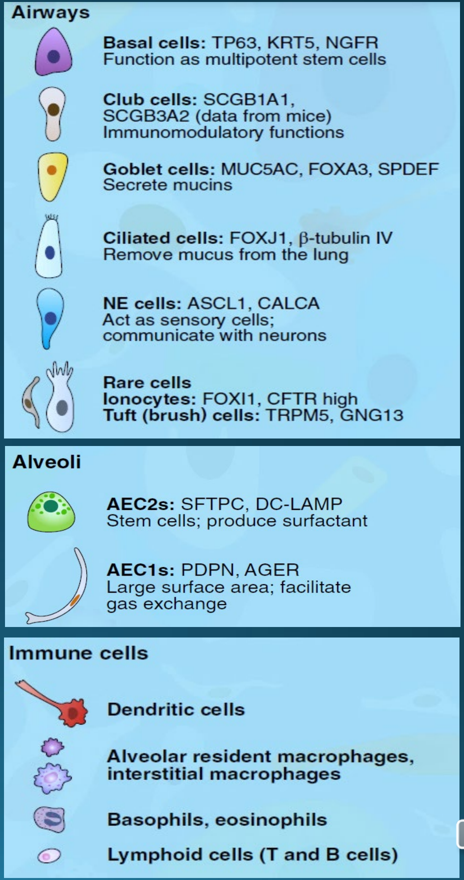

What endpoints/readouts to measure?

Molecular Initiating Event

- Cellular ab-/adsorption and hydrolysis of silane

Key Events

- Cell damage
- Loss of epithelial barrier

Adverse Outcome

- Pulmonary oedema and hemorrhage
- Lethality

- Si content in cells and basal medium

- Evident toxicity (cytotoxicity, cell viability, histology)
- Sub-toxic effects (inflammatory cytokines, cilia beating frequency)
- Trans-epithelial electrical resistance (TEER)

Find more information about AOPs under aopwiki.org

Phase I: Acute Tox of silane in a 2D model

Cell response to triethoxysilane exposure (60 min)

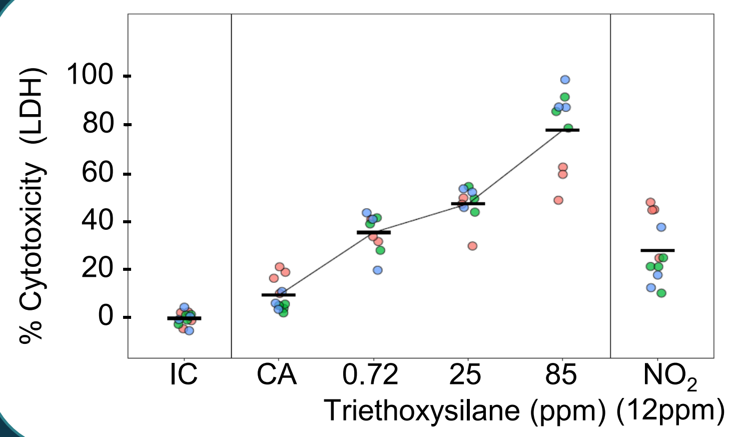

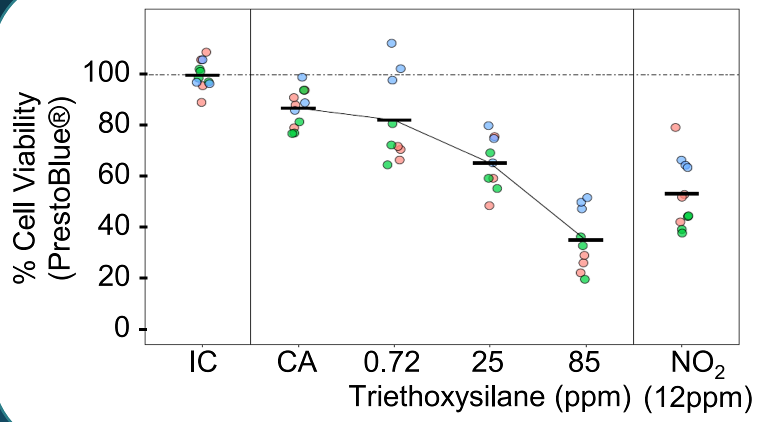

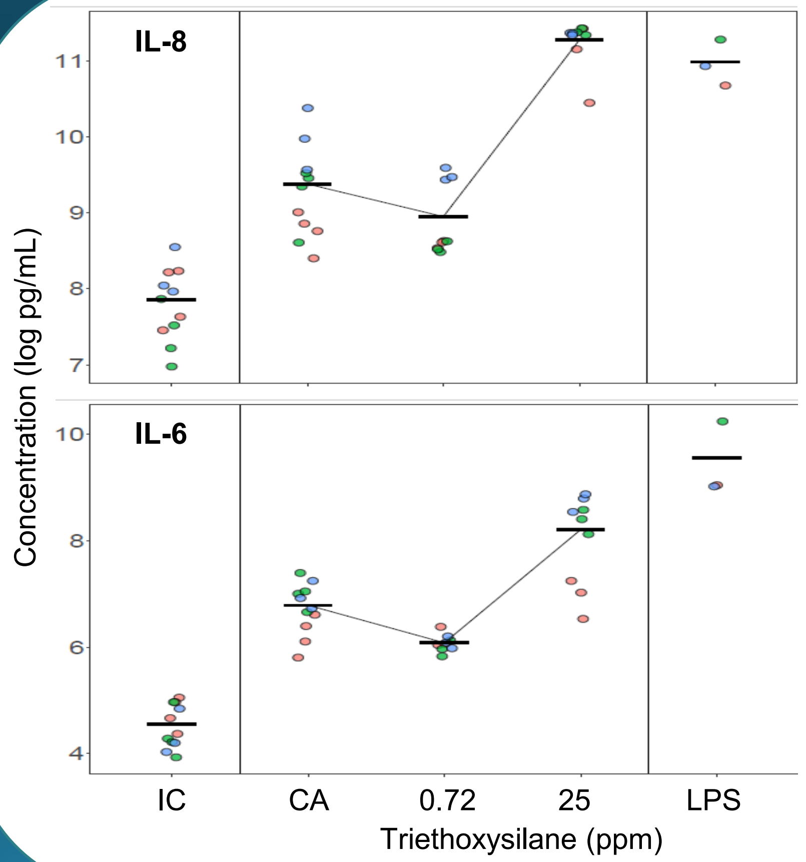

Phase I+II: Acute Tox of silane in a 2D model

2D cell model response to silane exposure (30 min)

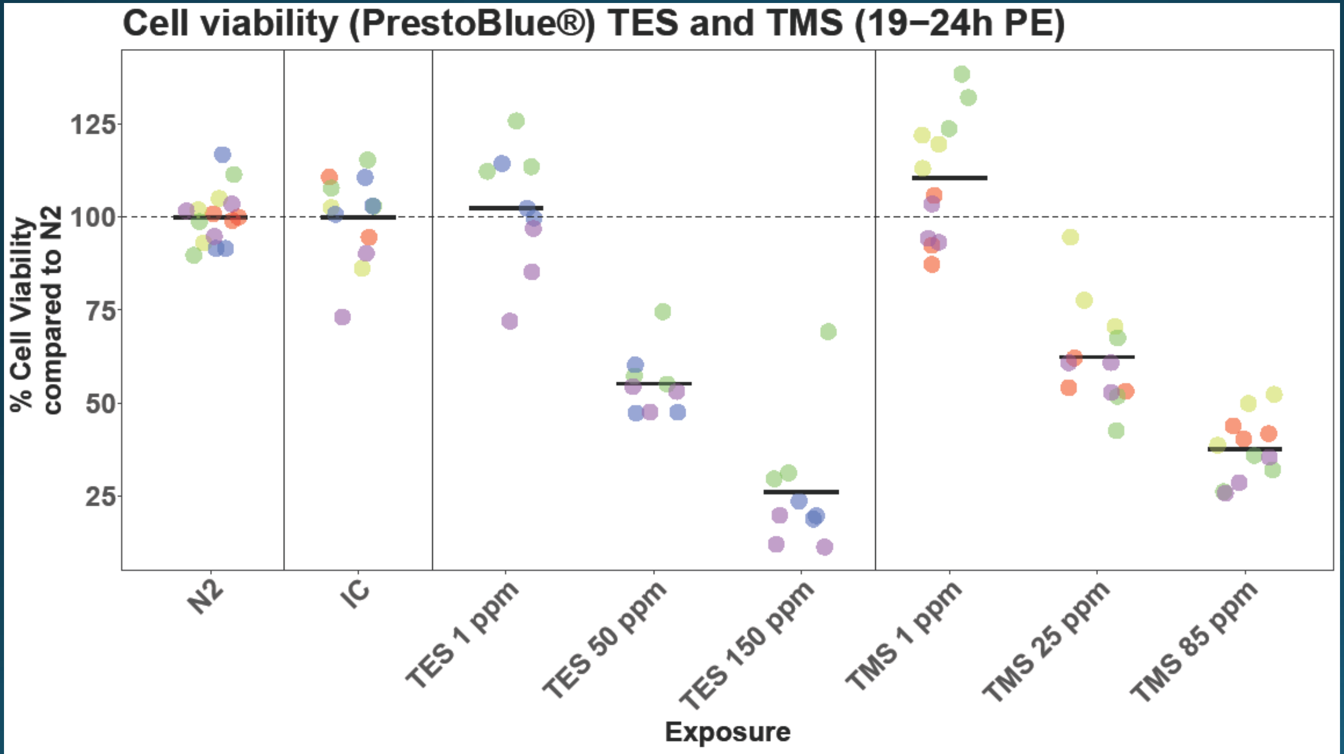

Phase III: Acute Tox of silanes in a 3D model

Silane toxicity to the epithelial barrier in 3D model

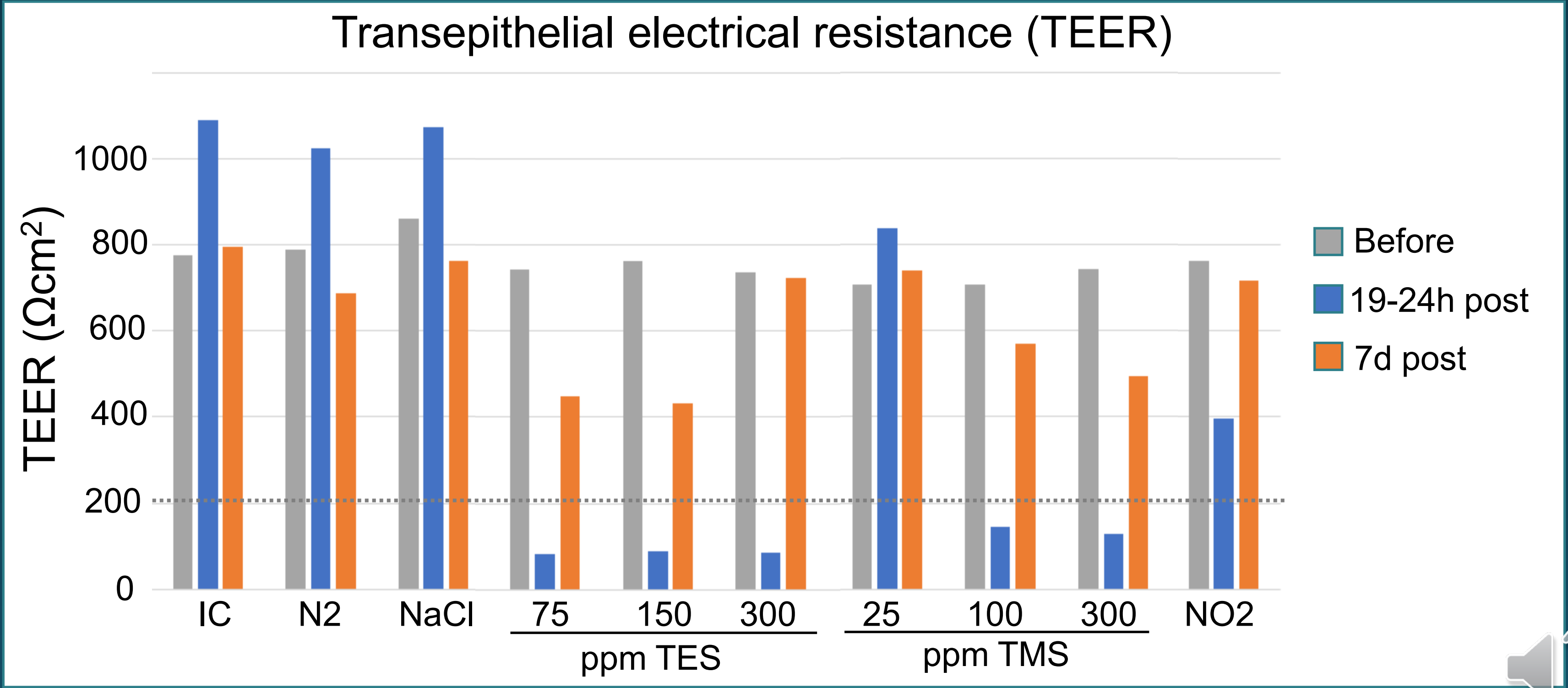

INSPiRE Initiative – Next steps

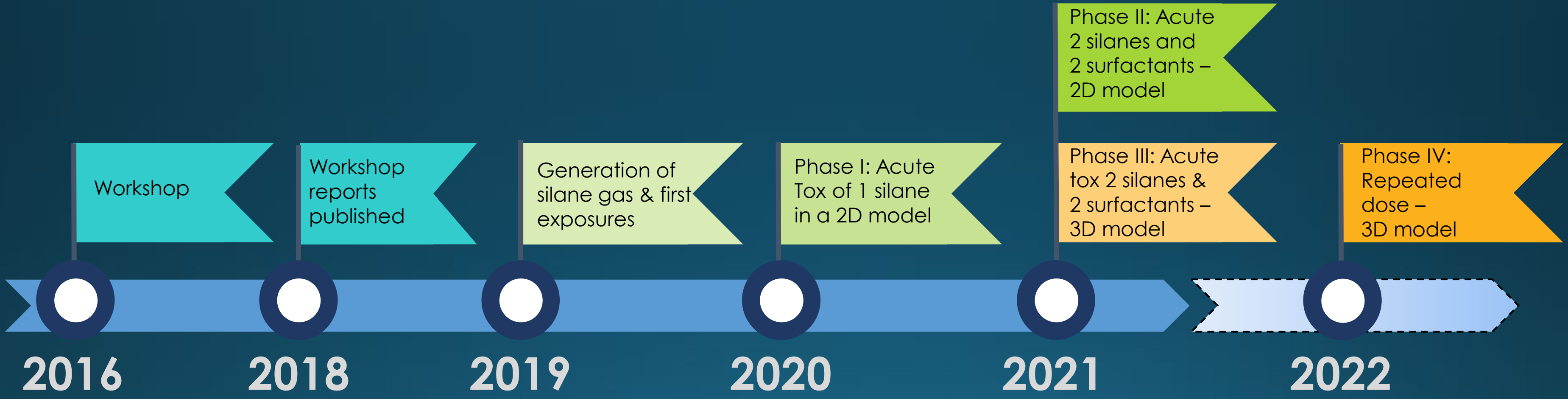

Take home

- Developments in recent years allow for human-relevant exposures of lung cells or tissues
- No size fits all: depending on the substance to test – *in vitro* methods may need adaptation (e.g., addition of endpoints)
- Rather than only having one *in vitro* assay, a battery of assays may be needed to answer a specific question (e.g., OECD TG 497 – Defined approaches on skin sensitisation)
- Especially in combination with *in silico* models, *in vitro* models have the potential to replace inhalation testing in animals
  - Chlorothalonil human health draft risk assessment - 90-day subchronic rat inhalation study waived by EPA OPP based on *in silico* and *in vitro* methods using MucilAir® tissue model *https://www.regulations.gov/document/EPA-HQ-OPP-2011-0840-0080*
- Talk to regulators **early** in the development process to discuss your NAM strategy and to find out whether animal testing is even necessary

Other inhalation related PSCI projects

Generating an Alternative System to Predict Pulmonary Fibrosis (GASPP)

- MatTek EpiAlveolar commercially available
- Used in EU-Horizon 2020 project PATROLS

Development of adverse outcome pathways (AOP)

- AOP 173: Substance interaction with the lung resident cell membrane components leading to lung fibrosis
- AOP 411: Oxidative stress leading to decreased lung function

FBS and Animal-Component free Testing (FACT)

- A549 cells sucessfully transitioned to animal-free medium

Precision-cut lung slices (PCLS)

- Study on cryopreservation ongoing

Awards

- 3D Tissues with MatTek and Epithelix
- CellTox Sampler with MedTec Bio
- VITROCELL inhalation exposure systems
- Travel Grants

Webinars and Workshops

- Several inhalation related webinars in 2016, 2018, 2020 and 2021
- https://www.thepsci.eu/inhalation-webinars/

Please visit www.thepsci.eu/our-work/inhalation/

Selected resources for respiratory *in vitro* methods

- https://www.thepsci.eu/inhalation-publications/
- Petersen EJ, Sharma M, Clippinger AJ, Gordon J, Katz A, Laux P, Leibrock LB, Luch A, Matheson J, Stucki AO, Tentschert J, Bierkandt FS. Use of Cause-and-Effect Analysis to Optimize the Reliability of In Vitro Inhalation Toxicity Measurements Using an Air-Liquid Interface. *Chem Res Toxicol*. 2021;34:1370–1385
- Welch J, Wallace J, Lansley AB, Roper C. Evaluation of the toxicity of sodium dodecyl sulphate (SDS) in the MucilAir™ human airway model in vitro. *Regul Toxicol Pharmacol*. 2021. Epub ahead of print.
- Hargrove MM, Dobrzansk BP, Li L, Constant S, Wallace J, Hinderliter P, Wolf DC, Charlton A. Use of the MucilAir Airway Assay, a New Approach Methodology, for Evaluating the Safety and Inhalation Risk of Agrochemicals. *Appl In Vitro Toxicol.* 2021;7(2):50-60.
- Marescotti D, Serchi T, Luettich K, Xiang Y, Moschini E, Talikka M, Martin F, Baumer K, Dulize R, Peric D, Bornand D, Guedj E, Sewer A, Cambier S, Contal S, Chary A, Gutleb AC, Frentzel S, Ivanov NV, Peitsch MC, Hoeng J. How complex should an in vitro model be? Evaluation of complex 3D alveolar model with transcriptomic data and computational biological network models. *ALTEX*. 2019; 36(3):388-402.
- Behrsing H, Hill E, Raabe H, Tice R, Fitzpatrick S, Devlin R, Pinkerton K, Oberdörster G, Wright C, Wieczorek R, Aufderheide M, Steiner S, Krebs T, Asgharian B, Corley R, Oldham M, Adamson J, Li X, Rahman I, Grego S, Chu PH, McCullough S, Curren R. *In vitro* exposure systems and dosimetry assessment tools for inhaled tobacco products: Workshop proceedings, conclusions and paths forward for in vitro model use. *Altern Lab Anim*. 2017;45(3):117-158.
- Behrsing H, Raabe H, Manuppello J, Bombick B, Curren R, Sullivan K, Sethi S, Phipps R, Tesfaigzi Y, Yan S, D’Ruiz C, Tarran R, Constant S, Phillips G, Gaça M, Hayden P, Cao X, Mathis C, Hoeng J, Braun A, Hill E. Assessment of *in vitro* COPD models for tobacco regulatory science: Workshop proceedings, conclusions and paths forward for *in vitro* model use. *Altern Lab Anim.* 2016;44(2):129-166.
- Clippinger AJ, Allen D, Behrsing H, BéruBé KA, Bolger MB, Casey W, DeLorme M, Gaça M, Gehen SC, Glover K, Hayden P, Hinderliter P, Hotchkiss JA, Iskandar A, Keyser B, Luettich K, Ma-Hock L, Maione A, Makena P, Melbourne J, Milchak L, Ng S, Paini A, Page K, Patlewicz G, Prieto P, Raabe H, Reinke E, Roper C, Rose J, Sharma M, Spoo W, Thorne PA, Wilson DM, Jarabek AM. Pathway-based predictive approaches for non-animal assessment of acute inhalation toxicity. *Toxicol In Vitro*. 2018;52:131-145.

Selected resources for *in vitro* methods in general

- PETA Science International Consortium https://www.thepsci.eu/
- NICEATM https://ntp.niehs.nih.gov/whatwestudy/niceatm/
- EPA‘s List of NAMs: https://www.epa.gov/assessing-and-managing-chemicals-under-tsca/alternative-test-methods-and-strategies-reduce
- EURL ECVAM https://ec.europa.eu/jrc/en/eurl/ecvam
- Tracking System for Alternative methods towards Regulatory acceptance (TSAR) https://tsar.jrc.ec.europa.eu/
- Frontiers in *in vitro* Toxicology Research Topic on Chemical Testing Using NAMs https://www.frontiersin.org/research-topics/19075/
- Non-Animal Technologies (NAT) Database: https://nat-database.org/
- AOP wiki: https://aopwiki.org

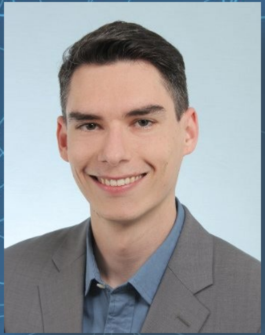

Thank you!

Andreas Stucki, Ph.D.
AndreasS@thepsci.eu

PETA Science Consortium International e.V.
www.thePSCI.eu
@thePSCI

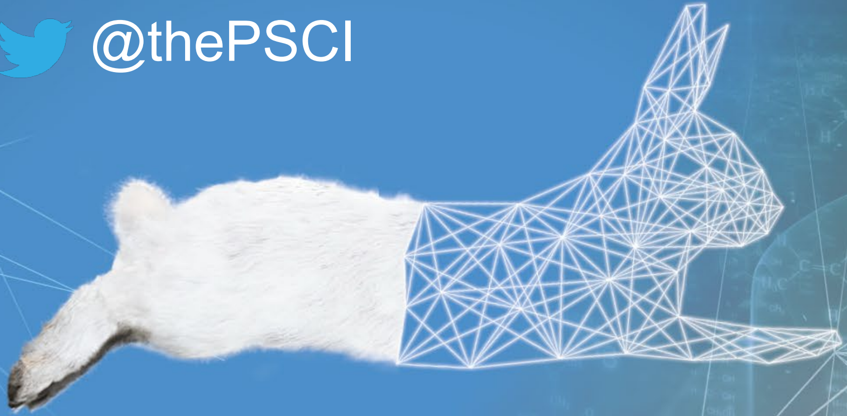

IN SILICO TOXICOLOGY AS A NEW APPROACH METHODOLOGY IN TOBACCO REGULATORY SCIENCE

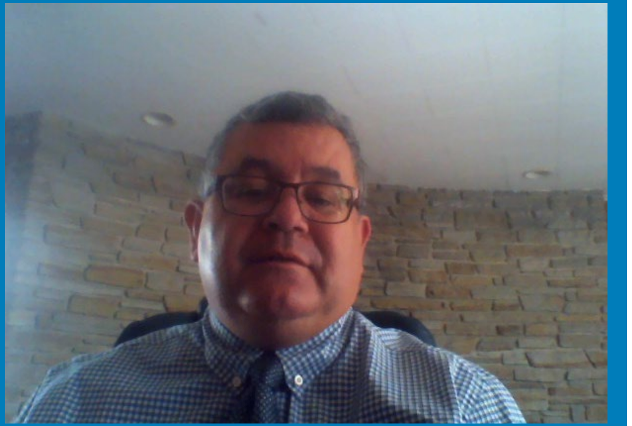

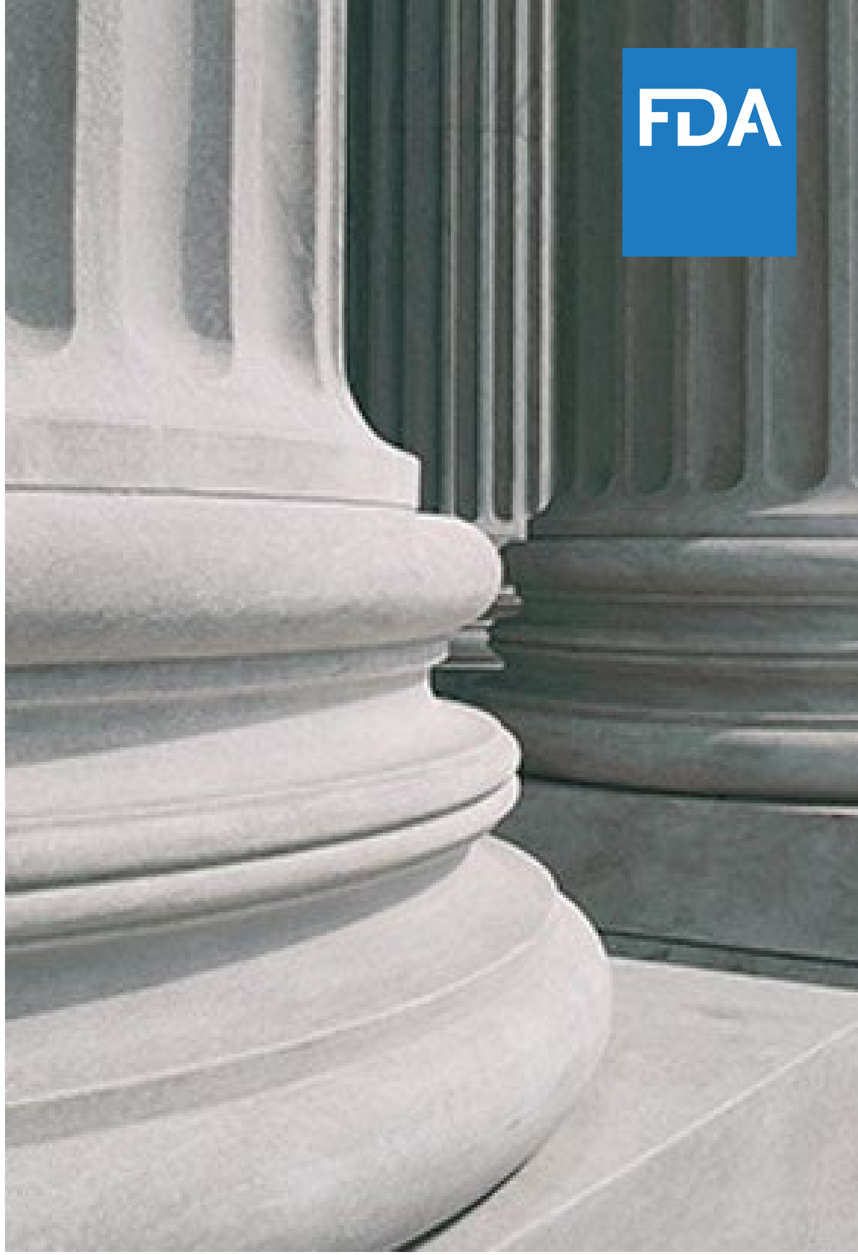

Luis G. Valerio, Jr., Ph.D., ATS
Associate Director
Division of Nonclinical Science
Office of Science | Center for Tobacco Products

Disclaimer: This is not a formal dissemination of information by FDA and does not represent Agency position or policy.

FDA

October 19, 2021

AGENDA

- In silico toxicology
  - Capabilities
  - Challenges

- Proof of concept applied regulatory research
  - Hazard identification
    - Screening for toxicity
    - Structural alerts
    - Validation testing of models
    - Chemical similarity analysis

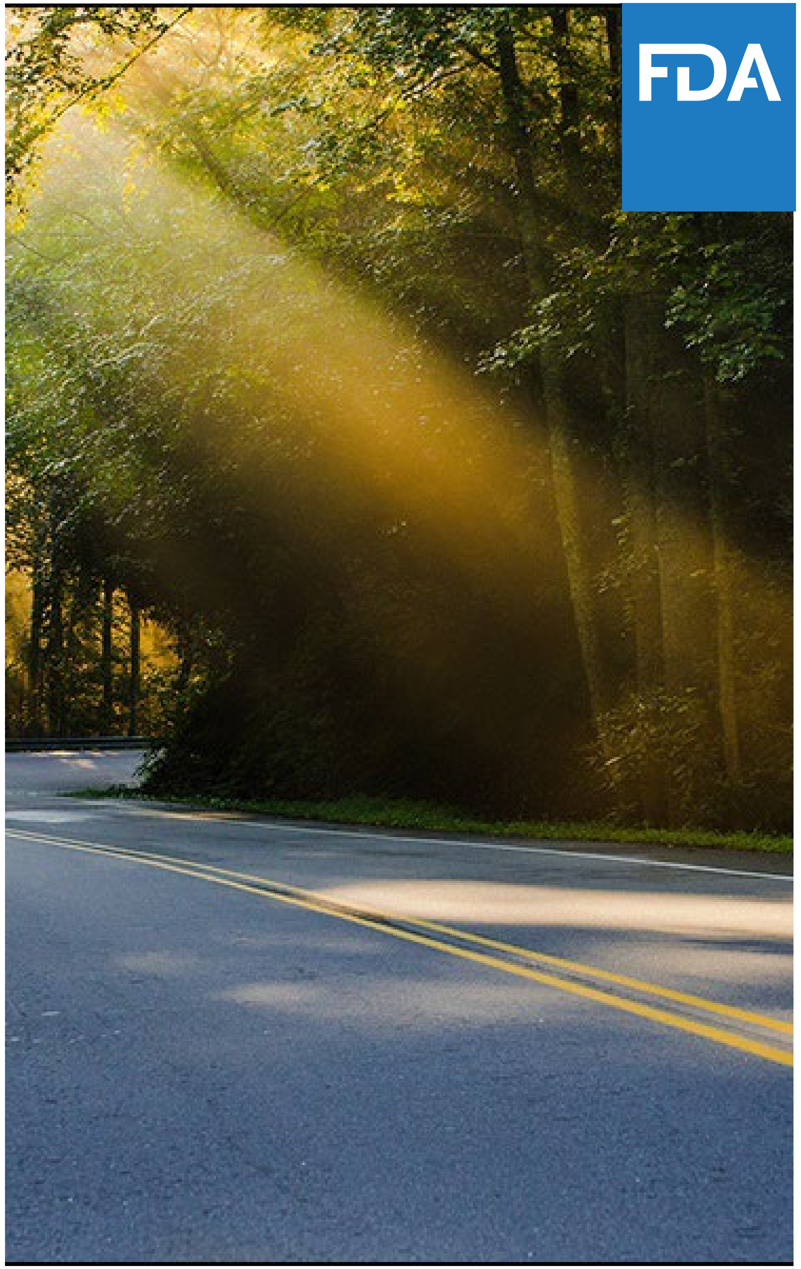

COMPUTATIONAL SCIENCE

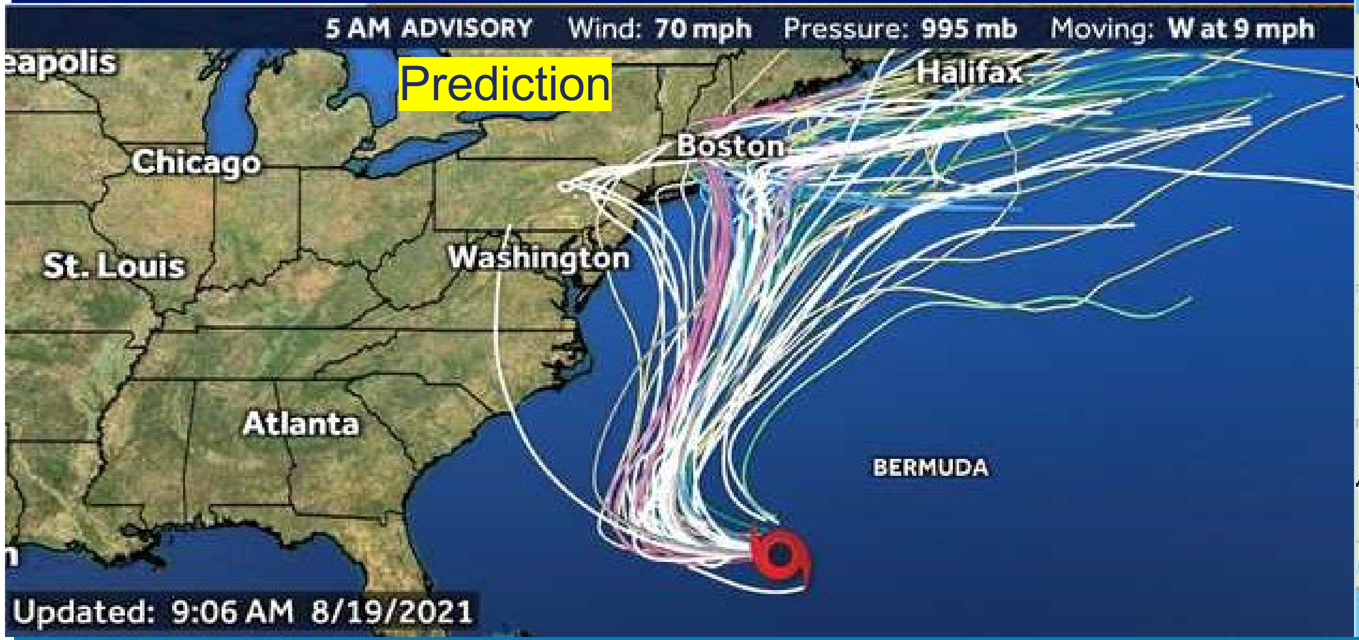

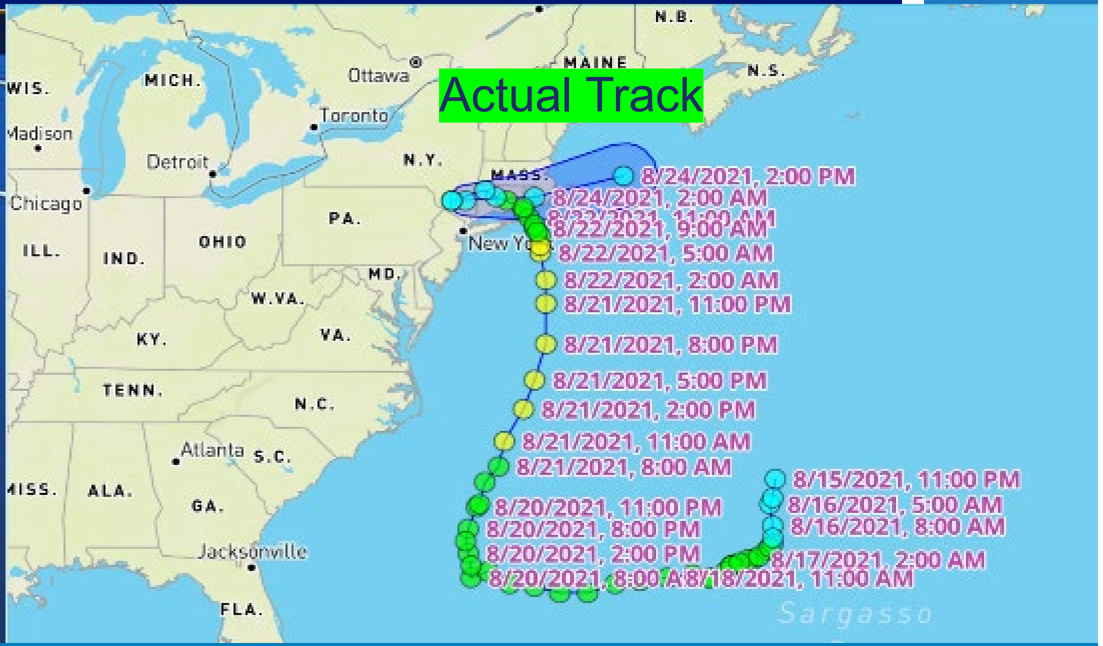

- Predictions for health and safety
- Ensemble of models - consensus
- Data mining for continuous updating

ADVANTAGES OF IN SILICO TOXICOLOGY

FDA

- Rapid and cost-effective
- Maximize resources
- 3Rs principals –reduction, refinement and replacement
  - Ethical and humane
- May strengthen or serve as complementary evidence
- Analyze for non-traditional toxicity alerts
- Enabler for consensus approaches
- Strategy to support prioritization for follow-up
- Pattern recognition
- Does not require synthesis of compound
- Predict hazard and provide mechanistic insight
- Data visualization tool

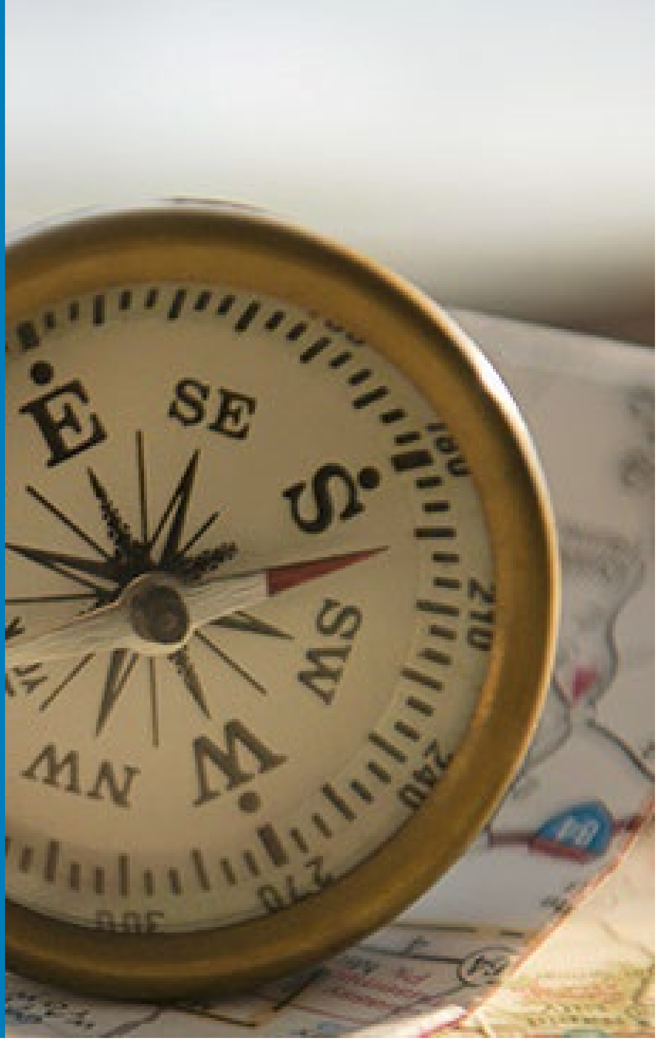

CHALLENGES OF IN SILICO TOXICOLOGY

- Interpretation by expert assessment is important
- Explain predictions
- Selection of technique appropriate for intended use
- Appropriate use of predictive modeling data – in/out of context
- Model selection, updating, and domain space
- Data quality
- Validation and performance
  - method sensitivity
  - prospective validation rare
  - retrospective validation common but what is considered ‘good’ performance

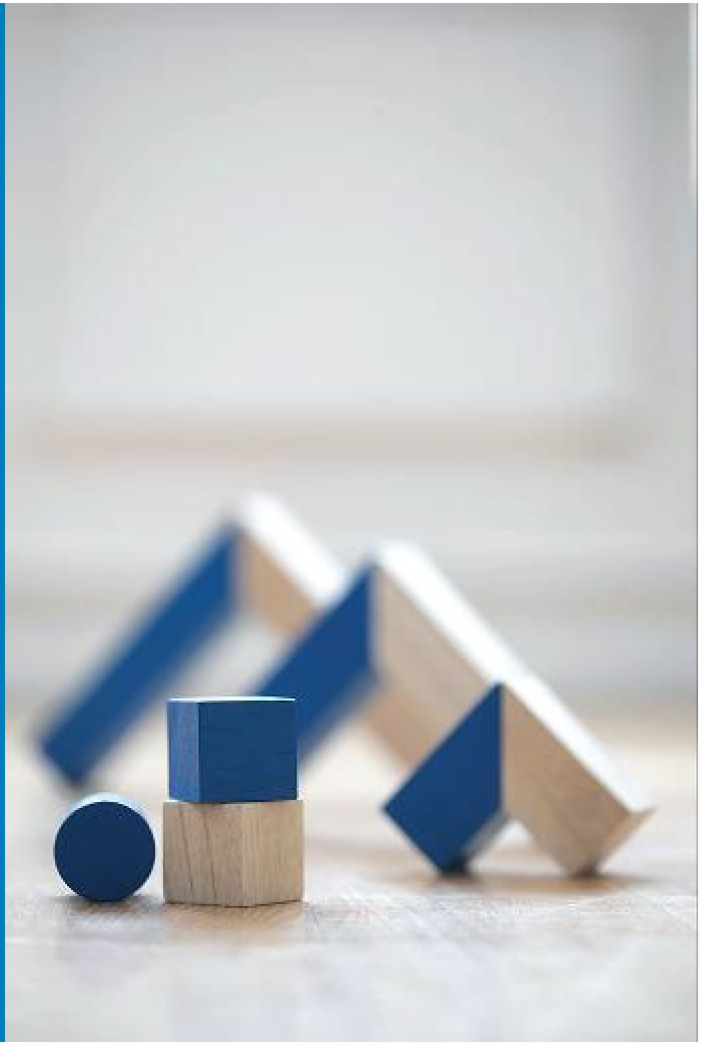

CONCEPT OF COMPUTATIONAL TOXICOLOGY IN TOBACCO REGULATORY SCIENCE

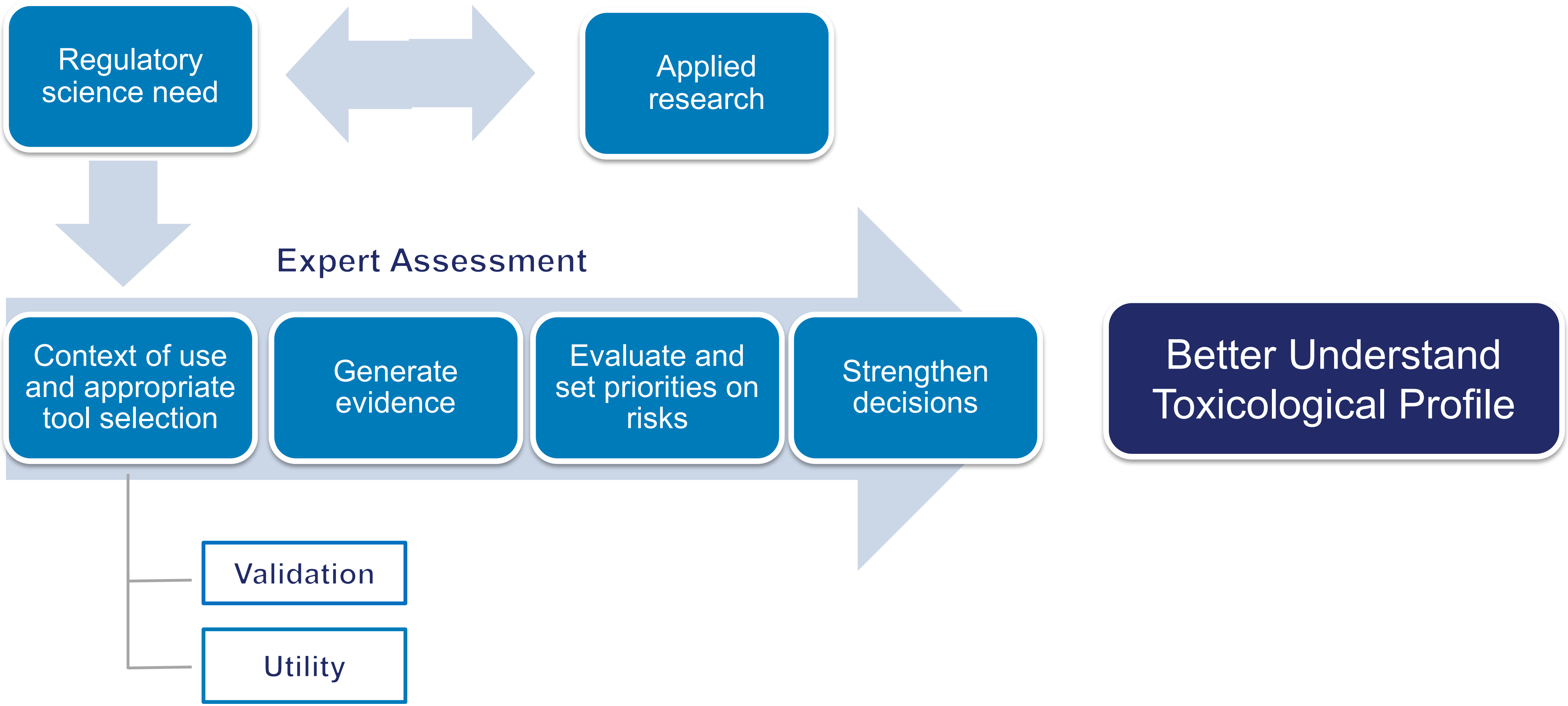

IN SILICO TOXICOLOGY METHODS ARE VERSATILE

- Screen for hazard identification
- Similarity analysis
- Support read-across/bridging
- Rapid detection of promiscuous compounds
  -molecular filtering
- Predict toxicities based on computational models
  -organ toxicity, DNA damage, endpoints unethical to test in humans
- Toxicokinetics
- Physical chemical properties
- Uncover structural alerts
- Mechanistic information
- Mine for toxicity data

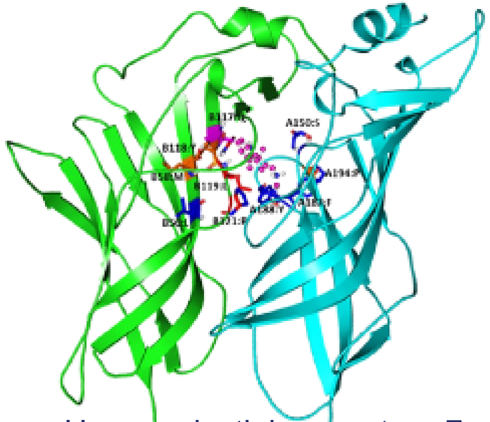
Human nicotinic receptor $\alpha 7$

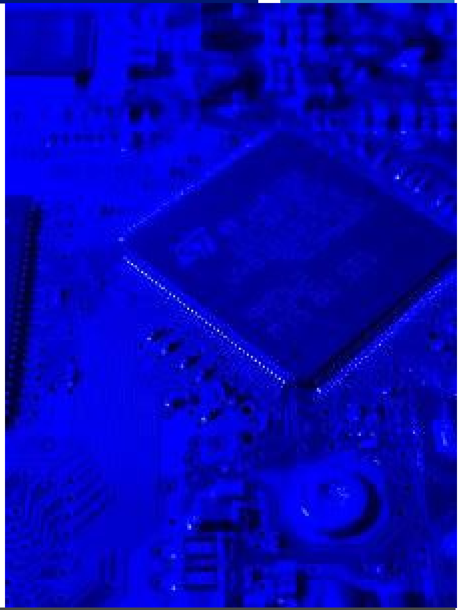

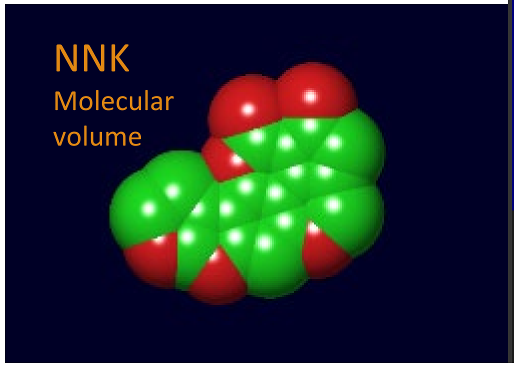

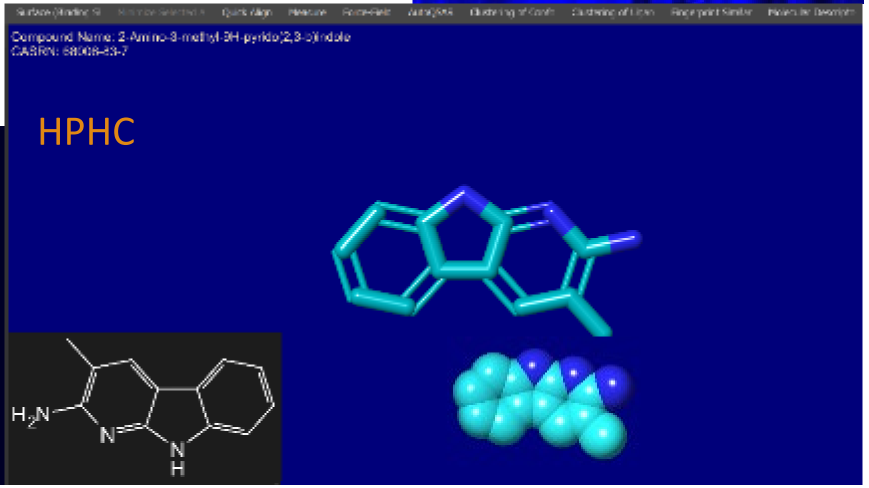

IN SILICO TOXICOLOGY METHODS ARE RAPID

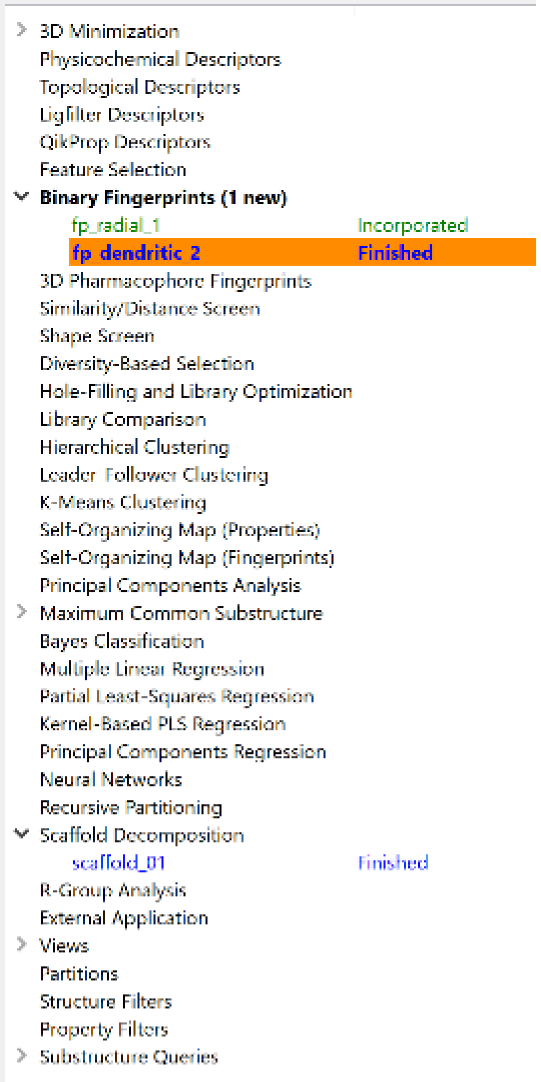

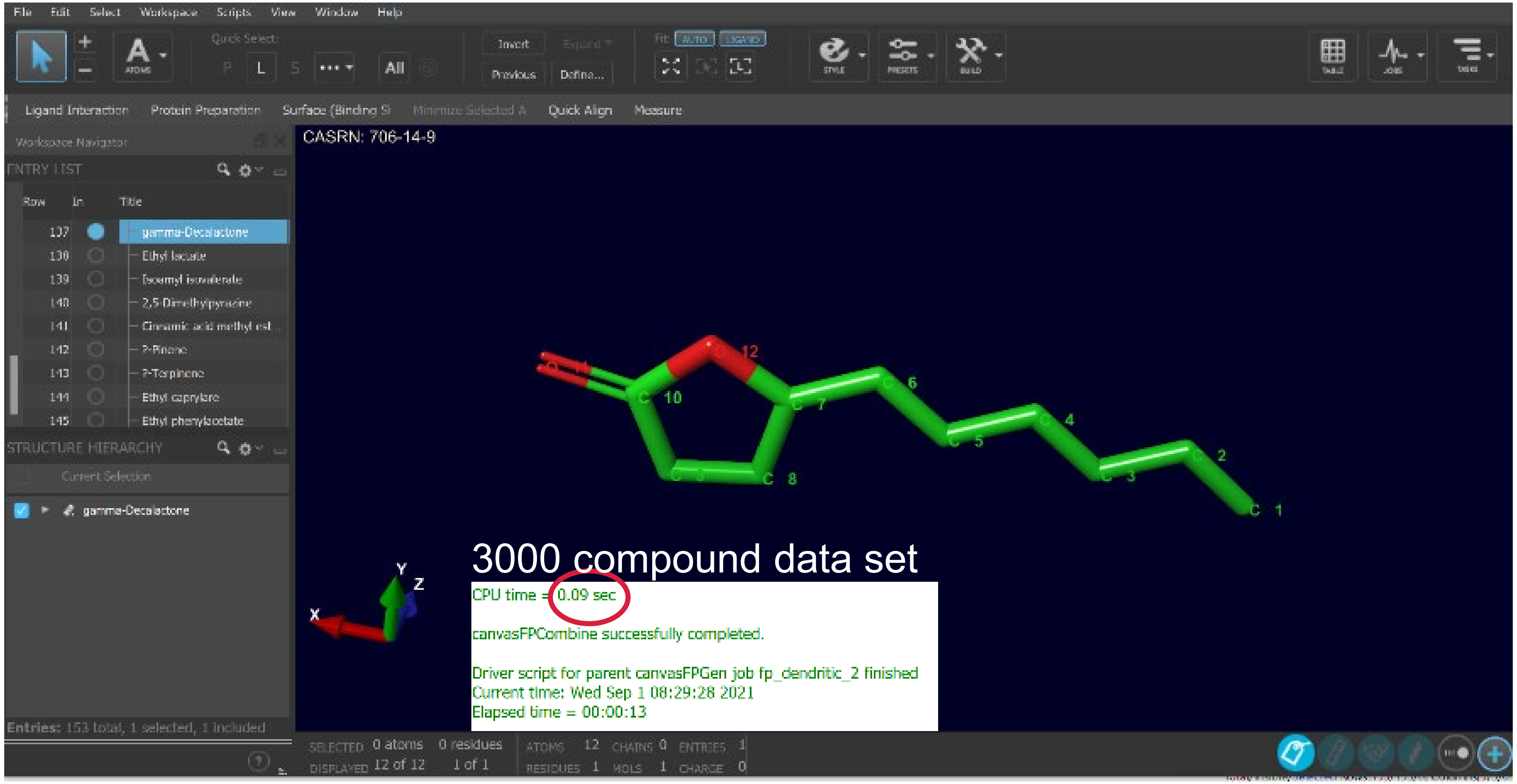

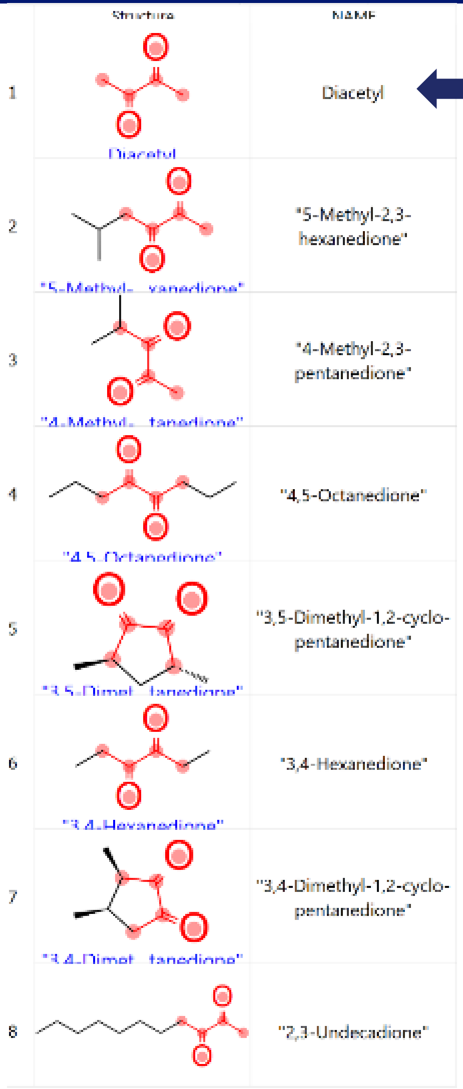

Molecular filtering

screen toxic substructures

Scaffold generation

decipher alerts

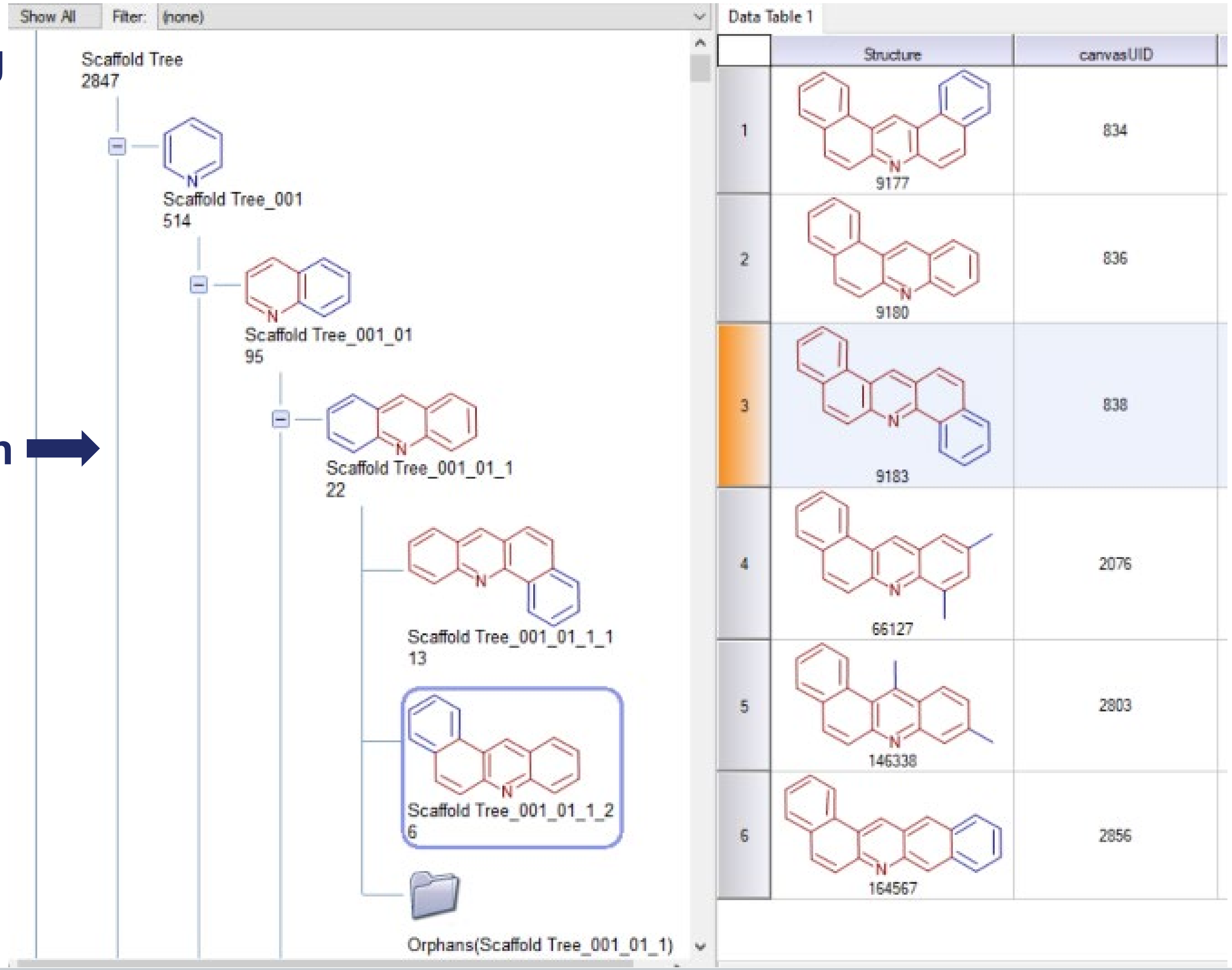

IN SILICO TOXICOLOGY STRUCTURE READ-ACROSS

FDA

Similarity

	[illegible]	[illegible]	[illegible]	[illegible]	[illegible]	[illegible]	[illegible]	[illegible]	[illegible]	[illegible]
1-...l]	1	0.875537	0.818778	0.755556	0.809524	0.510101	0.643312	0.775533	0.765427	0.789431
1,...ol	0.875537	1	0.912711	0.749155	0.860696	0.534392	0.682432	[illegible]	[illegible]	[illegible]
(3...ol	0.818778	0.912711	1	0.804455	0.853716	0.604079	0.647436	[illegible]	[illegible]	[illegible]
(R...al	0.755556	0.749155	0.804455	1	0.810502	0.661465	0.68775	[illegible]	[illegible]	[illegible]
1,...ol	0.809524	0.860696	0.853716	0.810502	1	0.485577	0.60479	[illegible]	[illegible]	[illegible]
2...e	0.510101	0.534392	0.604079	0.661465	0.485577	1	0.693574	0.783772	0.622182	0.727496
2,...ne	0.643312	0.682432	0.647436	0.68775	0.60479	0.693574	1	0.736628	0.590643	0.749155
2,...ne	0.775533	0.765235	0.778962	0.75297	0.742839	0.783772	0.736628	1	0.764045	0.881154
2,...al	0.765427	0.796569	0.893217	0.801567	0.829047	0.622182	0.590643	0.764045	1	0.874072
2...e	0.789431	0.8226	0.822581	0.784341	0.84715	0.727496	0.749155	0.881154	0.874072	1

Distance

	[illegible]	[illegible]	[illegible]	[illegible]	[illegible]	[illegible]	[illegible]	[illegible]	[illegible]	[illegible]
1-...l]	0	2.23607	2.64575	3.31662	3	3.87298	2.82843	3	3.31662	2.82843
1,...ol	2.23607	0	2.44949	3.16228	2.82843	3.74166	2.64575	2.82843	3.16228	2.64575
(3...ol	2.64575	2.44949	0	3.4641	3.16228	4	3	3.16228	3.16228	3
(R...al	[illegible]	[illegible]	[illegible]	[illegible]	3.4641	4.47214	3.60555	3.74166	4	3.60555
1,...ol	3	[illegible]	[illegible]	[illegible]	0	4.24264	3.31662	3.4641	3.4641	3
2...e	[illegible]	[illegible]	[illegible]	[illegible]	4.24264	0	4.12311	4.24264	4.47214	4.12311
2,...ne	[illegible]	[illegible]	[illegible]	[illegible]	3.31662	4.12311	0	3.31662	3.60555	3.16228
2,...ne	3	2.82843	3.16228	3.74166	3.4641	4.24264	3.31662	0	3.74166	3.31662
2,...al	3.31662	3.16228	3.16228	4	3.4641	4.47214	3.60555	3.74166	0	3
2...e	2.82843	2.64575	3	3.60555	3	4.12311	3.16228	3.31662	3	0

Dendritic fingerprint

To encode both linear and branched fragments, linear paths are augmented with intersections of linear paths, with a maximum 5 bonds per path.

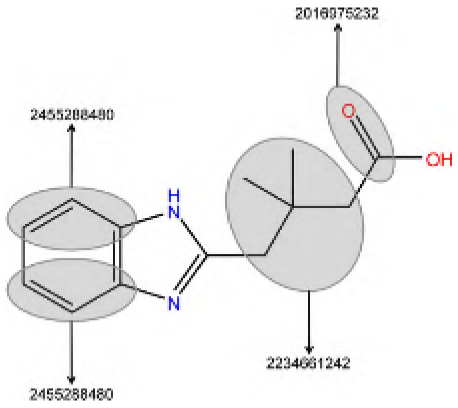

Duan et al. 2010. J Mol Graphics Modeling. 29:157-170

IN SILICO ASSESSMENT OF CHEMICAL SIMILARITY

FDA

Advantages:
- ✓ Science-based
- ✓ Transparent
- ✓ Mech. hypothesis
- ✓ Calculate PCPs
- ✓ Abundant molecular descriptors, metrics, and atom typing

Read-Across

**Similarity coefficient**<br>Buser metric, 2D Linear Chemical fingerprints	
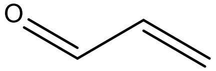 Acrolein	1.0
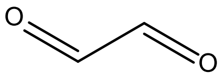 Glyoxal	0.9786
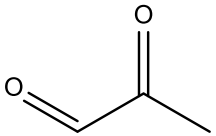 Methylglyoxal	0.9120
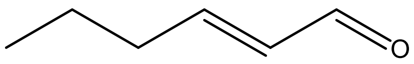 *trans*-2-Hexanal	0.9050
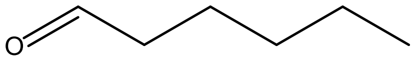 Hexanal	0.8757

Data rich

Confidence

Higher

Lower

Buser metric:
(sqrt(cd)+c)/sqrt(cd)+a+b-c)

Formula key:
a = On bits in structure 1
b = On bits in structure 2
c = On bits in both 1 and 2
d = Off bits in both 1 and 2

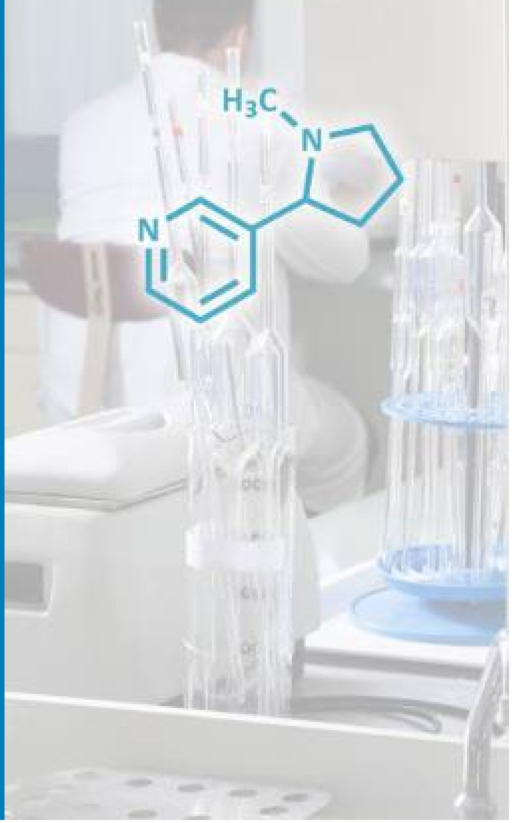

Kang and Valerio, 2020 Toxicol Appl Pharmacol. 398:115026

GAIN MECHANISTIC INFORMATION FROM A DATA SET

Michael Acceptors

"Soft" electrophiles that can alkylate nucleophilic sites on proteins or nucleic acids on DNA forming covalent adducts

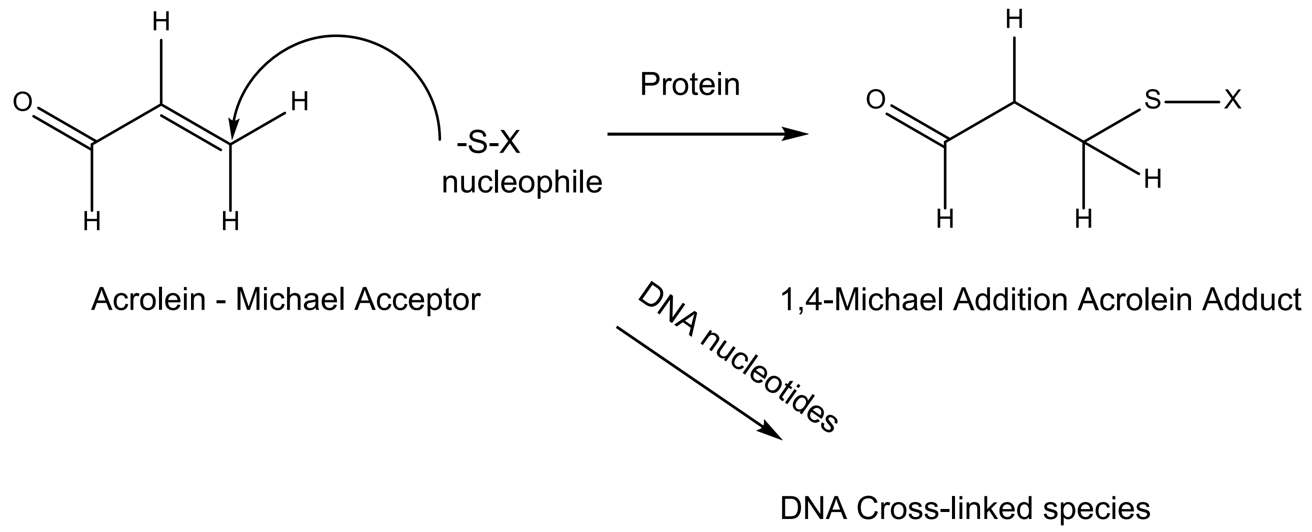

Other Factors

- Good leaving group
- Presence of electron withdrawing groups

Ou et al. 2020. J. Agric. Food Chem. 68(18):5039-5048.
Cai et al. 2009. Chem. Res. Toxicol.22(4):708-716.
Kozekov et al. 2003. JACS. 125(1):50-61.

MOLECULAR FILTERS

Michael Acceptors SMARTS: [#6]=[#6][#6,#16]=[O]

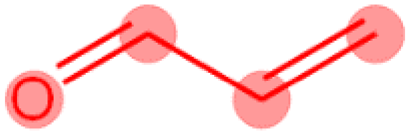

Acrolein (107-02-8)

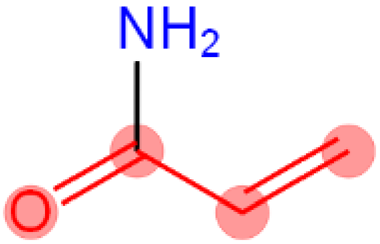

Acrylamide (79-06-1)

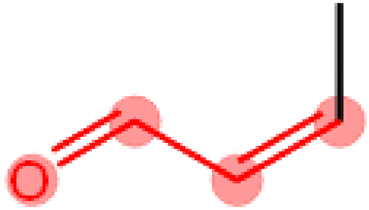

Crotonaldehyde (123-73-9)

Dicarbonyl SMARTS: [C;X3](=O)([C;X3](=O))

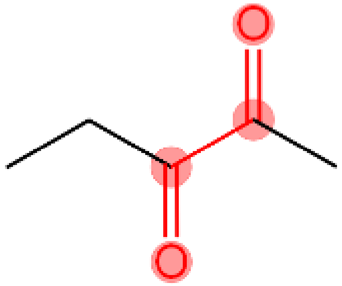

Pentanedione (600-14-6)

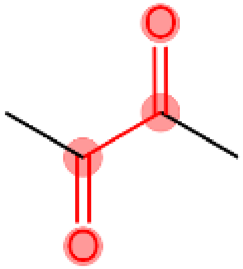

Diacetyl (431-03-8)

SMARTS: SMiles ARbitrary Target Specification

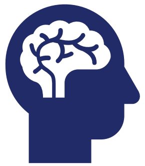

UNSUPERVISED MACHINE LEARNING TECHNIQUES

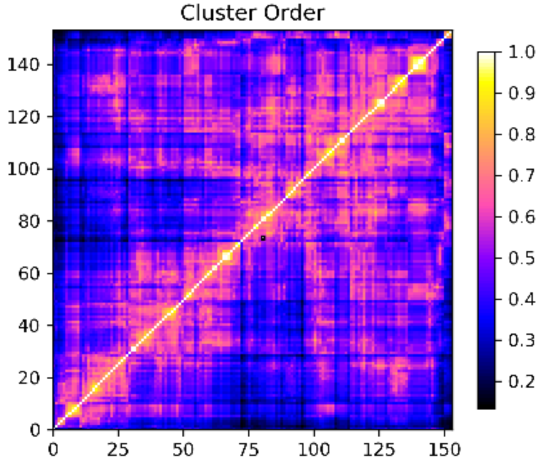

Hierarchical Clustering Dendrogram

By property for in vitro DNA damage

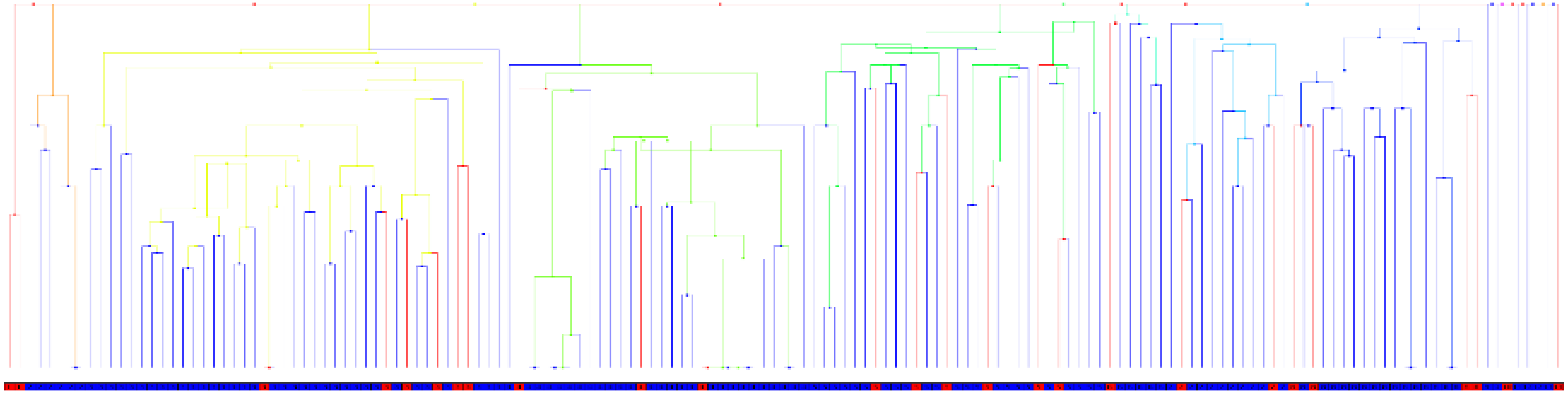

Property	Display	Min	Max
DNA Damage ...	☑	0	1

Self Organizing Map

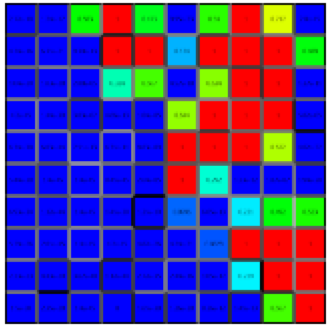

APPLIED RESEARCH IN COMPUTATIONAL TOXICOLOGY

FDA

Toxicology and Applied Pharmacology 398 (2020) 115026

Contents lists available at ScienceDirect

Toxicology and Applied Pharmacology

journal homepage: www.elsevier.com/locate/taap

ELSEVIER

Investigating DNA adduct formation by flavor chemicals and tobacco byproducts in electronic nicotine delivery system (ENDS) using in silico approaches

Jueichuan (Connie) Kang[a,b,*], Luis G. Valerio Jr[a]

[a] United States Food and Drug Administration, Center for Tobacco Products, Office of Science, Division of Nonclinical Science, 11785 Beltsville Drive, Calverton, MD 20705, USA
[b] US Public Health Service Commissioned Corps, Rockville, MD, USA

Received: 19 February 2020 | Revised: 12 May 2020 | Accepted: 16 May 2020
DOI: 10.1002/jat.4020

RESEARCH ARTICLE

Journal of Applied Toxicology WILEY

In vitro and in silico genetic toxicity screening of flavor compounds and other ingredients in tobacco products with emphasis on ENDS

Pei-Hsuan Hung[1] | Matthew Savidge[1] | Mamata De[1] | Jueichuan (Connie) Kang[1,2] | Sheila M. Healy[1] | Luis G. Valerio Jr.[1]

JCIM JOURNAL OF CHEMICAL INFORMATION AND MODELING

pubs.acs.org/jcim Article

Development of a Nicotinic Acetylcholine Receptor nAChR α7 Binding Activity Prediction Model

Sugunadevi Sakkiah, Carmine Leggett,* Bohu Pan, Wenjing Guo, Luis G. Valerio, Jr., and Huixiao Hong*

Cite This: *J. Chem. Inf. Model.* 2020, 60, 2396–2404 Read Online

TOXICOLOGY MECHANISMS AND METHODS
https://doi.org/10.1080/15376516.2020.1805836

Taylor & Francis
Taylor & Francis Group

ORIGINAL ARTICLE

Check for updates

Predicting the mutagenic potential of chemicals in tobacco products using *in silico* toxicology tools

Reema Goel and Luis G. Valerio, Jr.

United States Food and Drug Administration, Division of Nonclinical Science, Office of Science, Center for Tobacco Products, Calverton, MD, USA

PROJECT-BASED ASSESSMENTS

- Designed to assess utility and a proof of concept
  - Machine learning techniques
  - Endpoint
  - Performance
  - Applicability

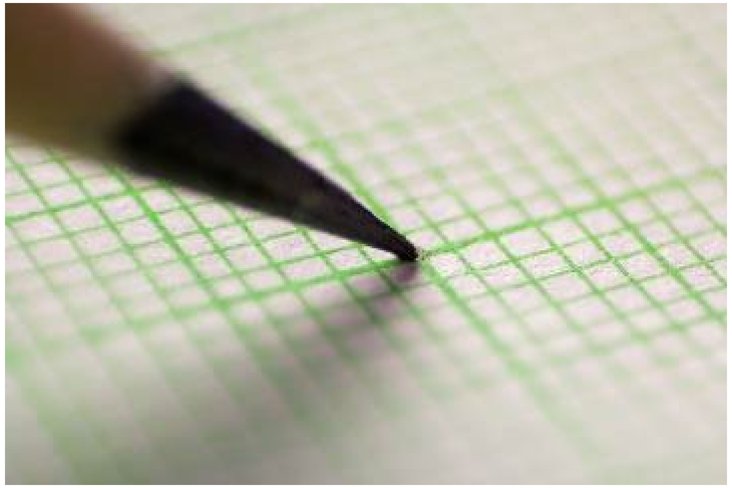

Constituents

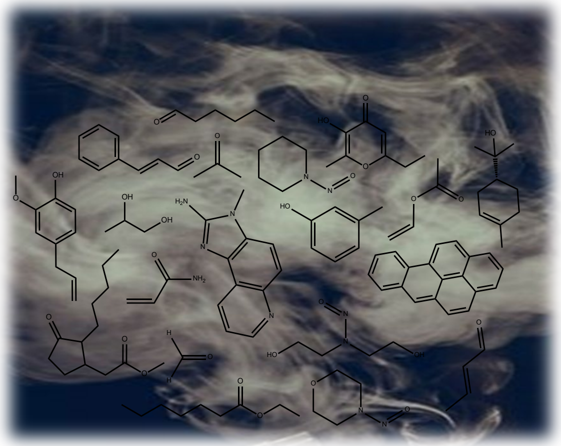

Endpoint

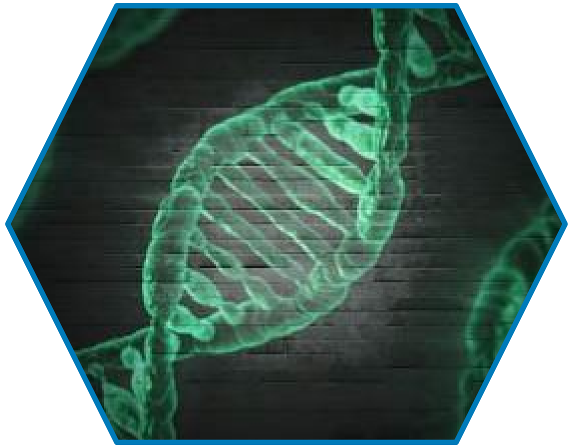

Computational

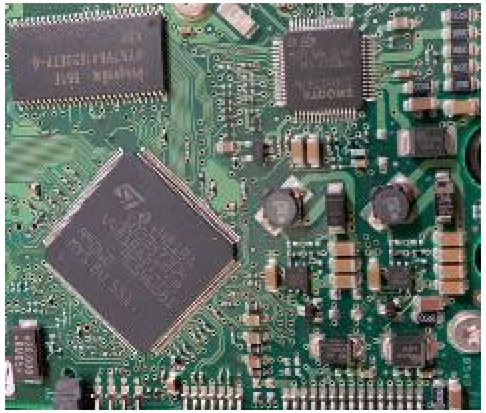

Evaluation

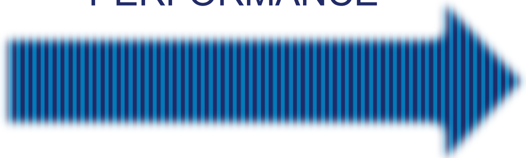

PREDICTING MUTAGENIC POTENTIAL OF CHEMICALS FROM TOBACCO PRODUCTS

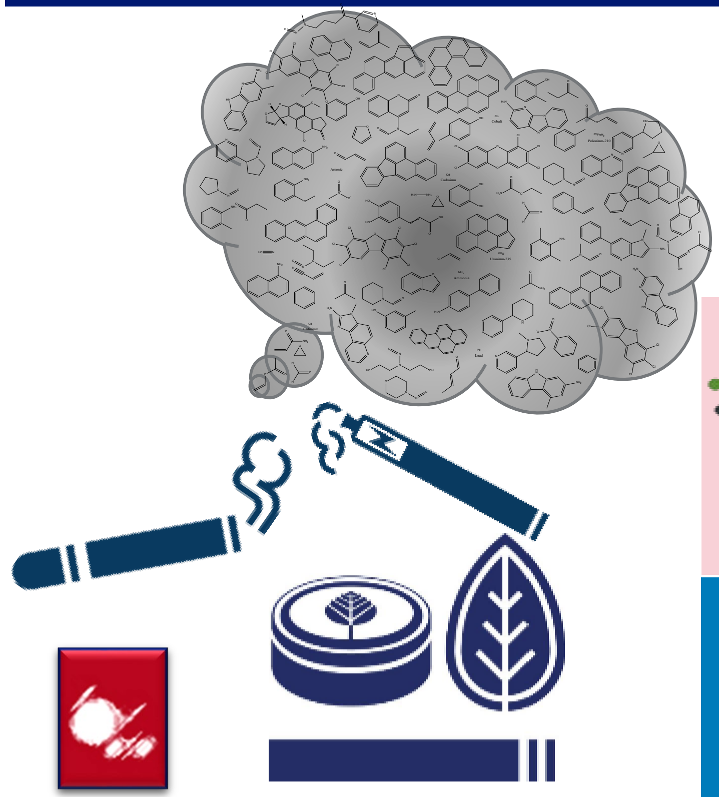

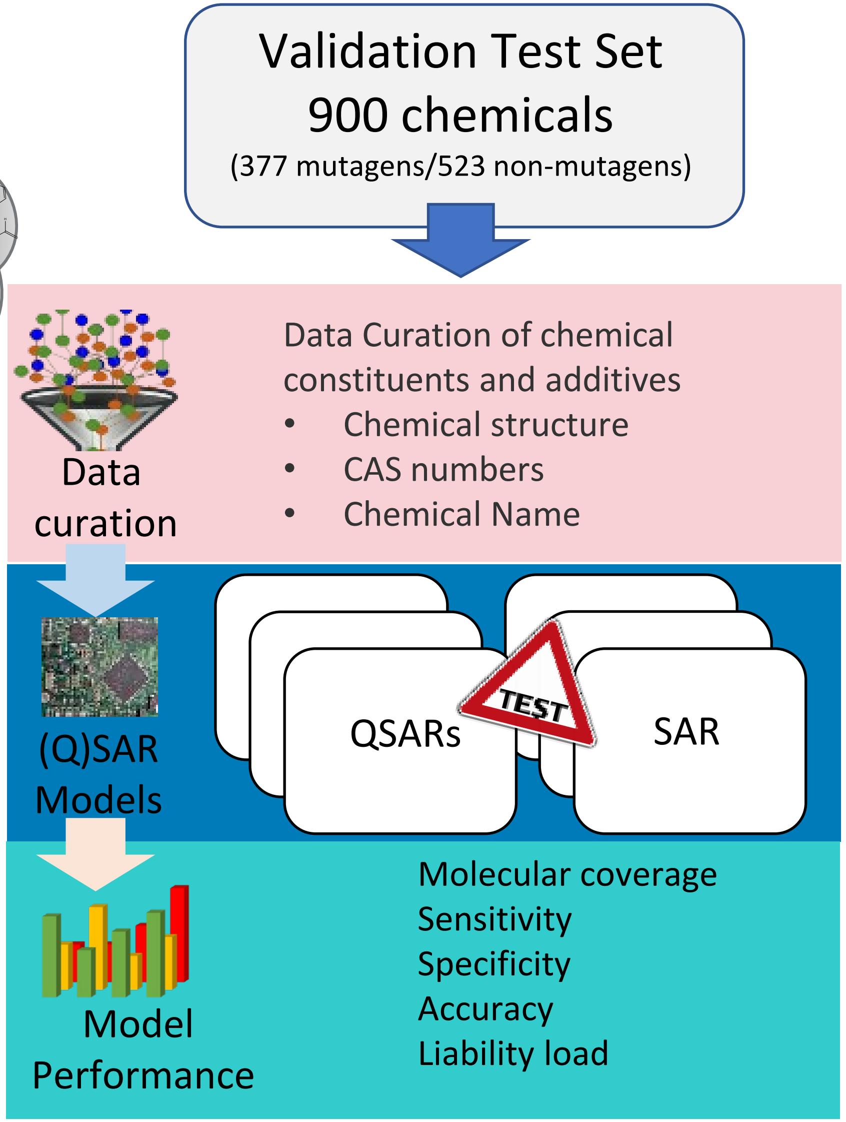

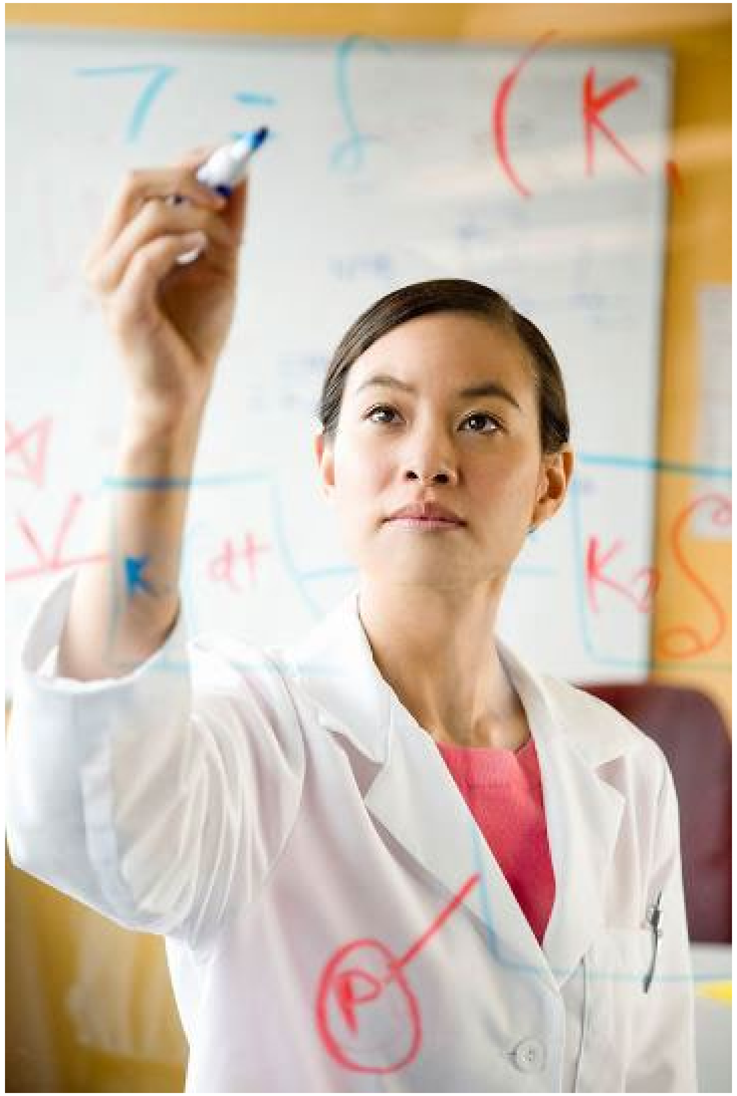

PREDICTIVE PERFORMANCE OF THE (Q)SAR MODELS

Ames test for mutagenicity

- ✓ **Predicted tobacco chemicals**
- ✓ **SAR and QSAR models performed similarly**
- ✓ **Combination of SAR/QSAR did not enhance performance**
- ✓ **Average Accuracy 89%**

Predicted

**Actual**	Non-Toxic	Toxic
Non-Toxic	Specificity	Type I Error (False +)
Toxic	Type II Error (False −)	Sensitivity

In Silico Model	Molecular Coverage	Sensitivity	Specificity	Accuracy	Liability Load
Model 1 (QSAR)	99.89%	77.5%	72.8%	74.7%	48.33%
Model 2 (QSAR)	98.56%	92.0%	96.9%	94.8%	41.67%
Model 3 (QSAR)	94.44%	93.8%	93.2%	93.4%	47.56%
Model 4 (SAR)	99.00%	91.8%	96.9%	94.7%	41.11%
Model 5 (QSAR+SAR)	91.44%	66.2%	92.3%	81.5%	37.67%
Model 6 (QSAR+SAR)	98.22%	92.0%	97.2%	95.0%	41.78%

TP = True Positive; FP = False Positive; TN = True Negative; FN = False Negative

Goel and Valerio, 2020 Toxicol Mech Methods. 30(9)672-678.

NEW APPROACH METHODOLOGIES IN PARALLEL

FDA

Tested 150 Chemical flavors

Biomarkers relevant to DNA Damage Response Pathways
p53, γH2AX, phosopho-histone-H3

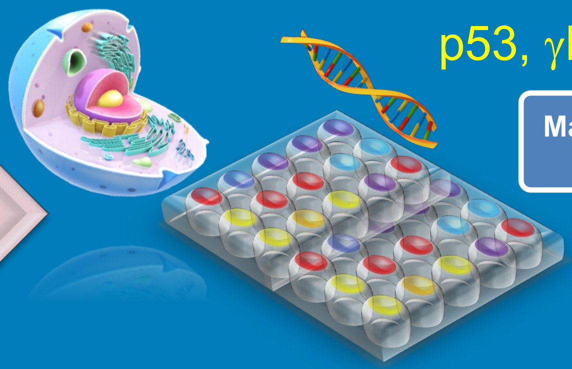

In Vitro
- MultiFlow® DNA Damage Assay

In Silico
- SAR/QSAR Computer Models

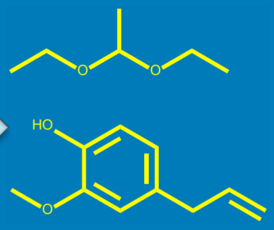

Machine Learning

Random Forest, Artificial Neural Networks, Logistic Regression

PREDICTIONS

Mode of Action

Chemical Hazard ID
Risk Prioritization

Hung et al. 2020. J Appl Toxicol. 40(11):1566-1587
*Contract: HHSF223201510009I

AGREEMENT BETWEEN IN VITRO AND IN SILICO RESULTS

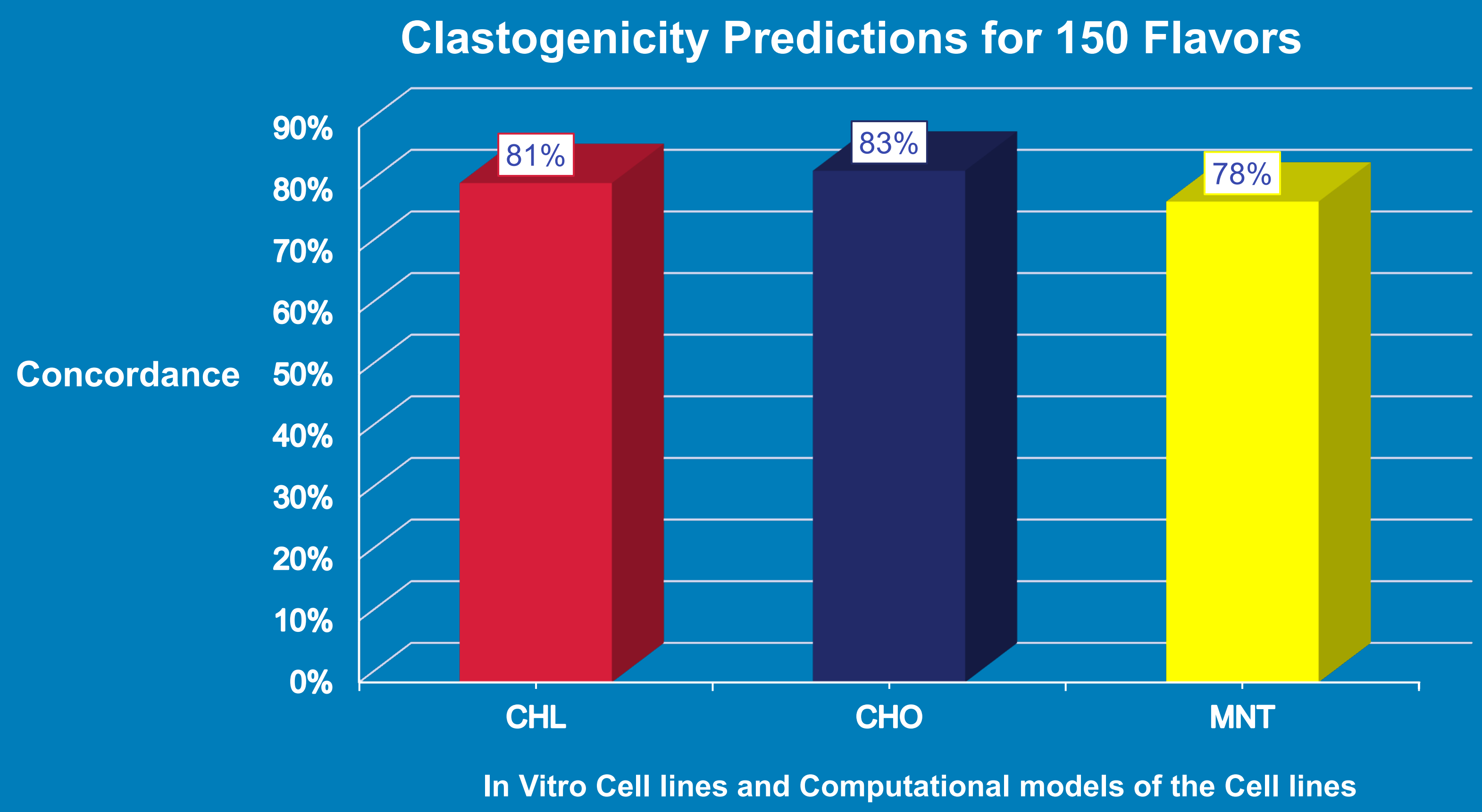

CONTRIBUTIONS OF THIS APPLIED RESEARCH

- First validation study using QSAR/SAR computational models for predicting mutagenic potential of tobacco chemicals
- Evaluated utility of in vitro and in silico screening technologies for assessing DNA damage by chemically defined flavors
- Assessed use of in silico molecular filters/substructure searching as a screening tool for chemically reactive moieties that portend to respiratory toxicity and DNA modification
- Evaluated use of chemical fingerprints for similarity analysis for potential application to support read-across of compound structures

ACKNOWLEDGEMENTS

Reema Goel, PhD

Connie Kang, PhD

Hans Rosenfeldt, PhD

Todd Cecil, PhD

Office of Science

Division of Regulatory Science and Informatics

United States Environmental Protection Agency

Application of Mechanistic Data in Risk Assessment: Exposure Alignment and Evidence Integration

Annie M. Jarabek
Senior Science Advisor

Advancing New Approach Methods for Tobacco Harm Reduction
Virtual Symposium
CORESTA Smoke Science and Product Technology Conference
October 19, 2021

OneORD Quality Together

EPA at 50 Progress for a Stronger Future

Office of Research and Development
Center for Public Health and Environmental Assessment (CPHEA)

Topics

- **Challenge:** Coherent evidence integration across large landscape of risk assessment applications
- **Building confidence:** Create context for translation based on mechanistic modeling to advance novel approach methods (NAMs)
  - AEP and AOP frameworks
  - Exposure alignment
  - Quantitative AOP and IATA
- **Case study:** Evaluation of new chemical substances under TSCA
- **Specific considerations:** Communication and characterization
  - Reporting standards
  - Uncertainty / variability and new translation factors
- **Summary**

Disclaimer: These views are those of the author and do not represent US EPA policy.

Risk Assessment Landscape

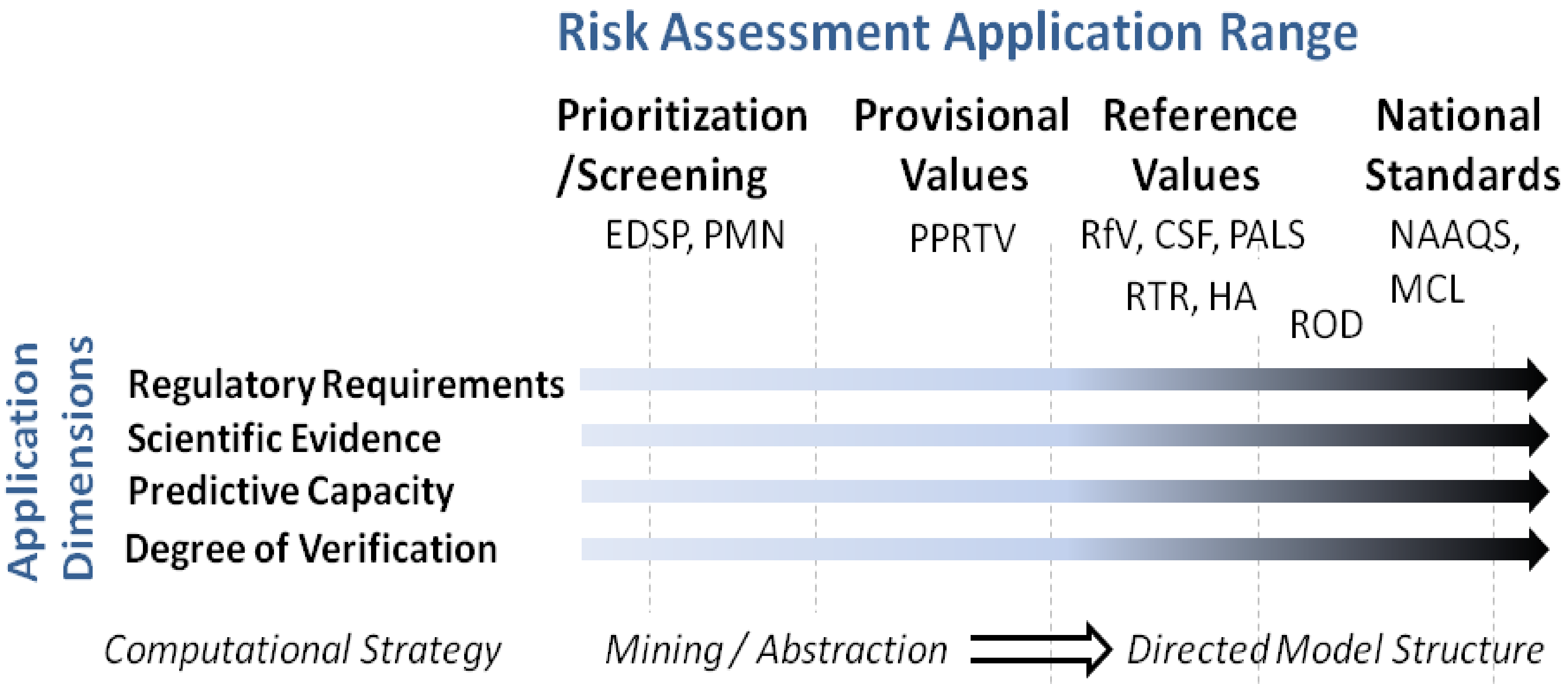

- Problem formulation: Fit for purpose
- Different data sources and strategies across landscape
- ***Mechanistic approach can create coherent context***

United States Environmental Protection Agency

Challenge: Evidence Integration

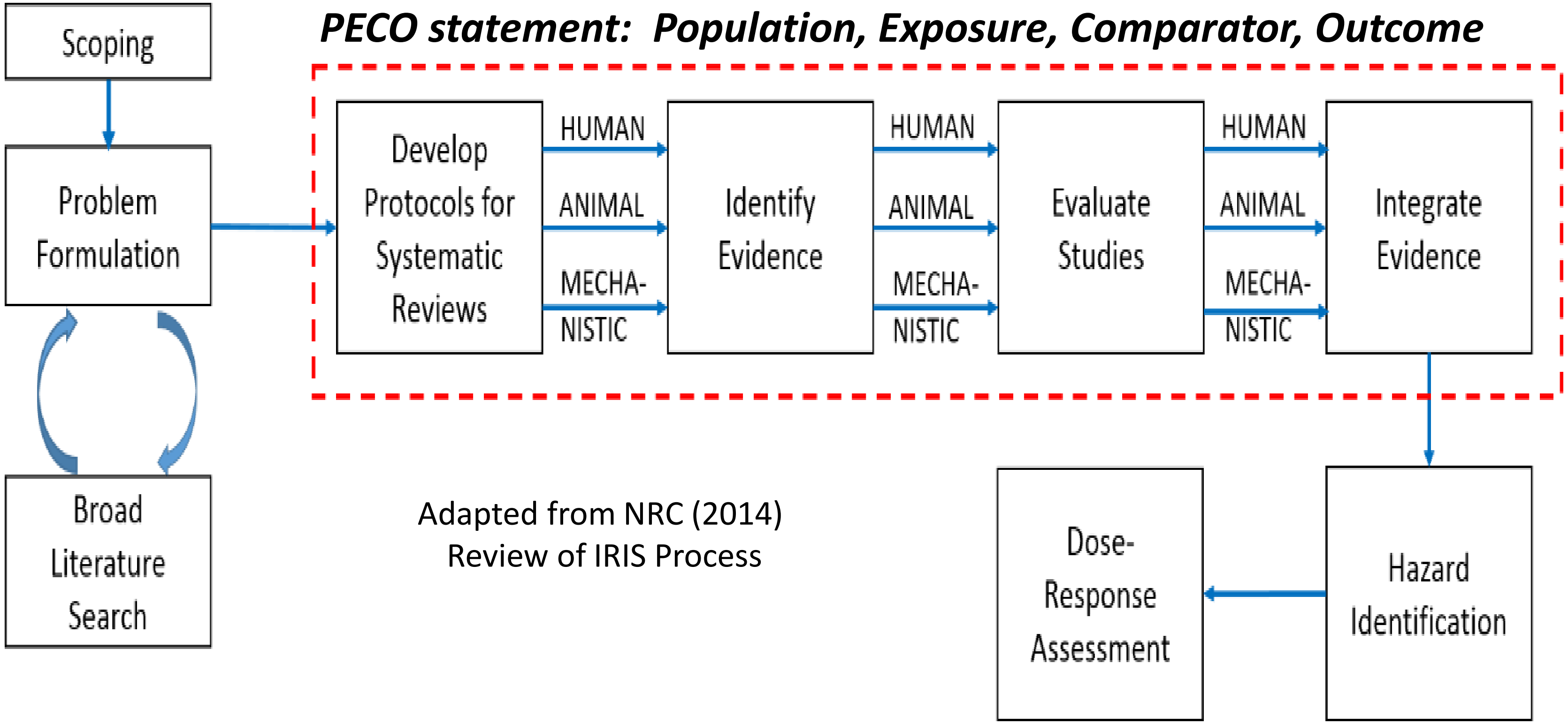

- Diverse exposure systems
- Dose at different levels of biological organization
- Various types of outcomes and modeling approaches
- Mechanistic data not considered in an integrated structure

Transitions: Comprehensive Characterization

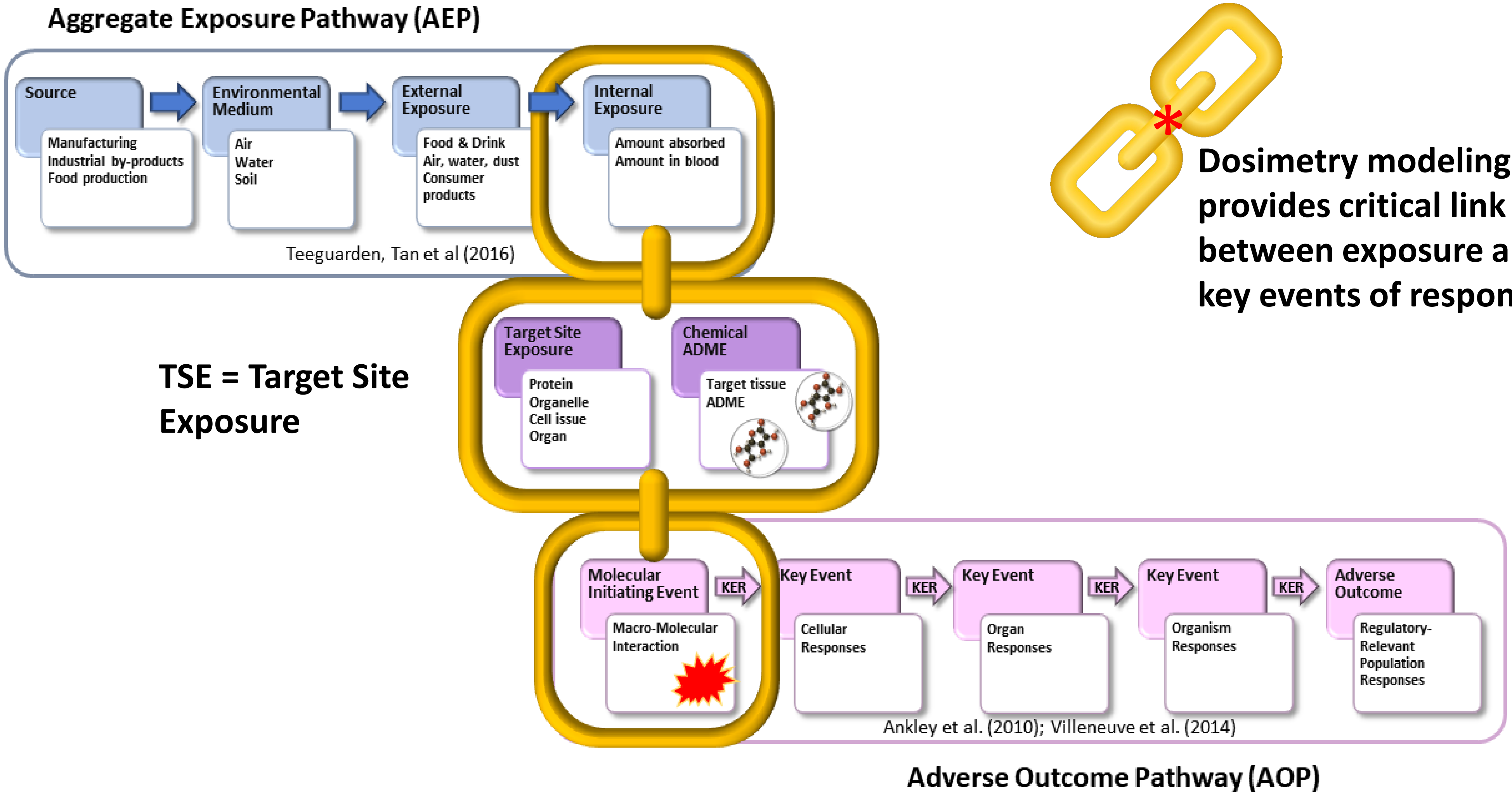

TSE = Target Site Exposure

Dosimetry modeling provides critical link between exposure and key events of response

Transitions: Novel Approach Methods (NAMs)

- EPA Strategic Plan published June 22, 2018 (https://www.epa.gov/assessing-and-managing-chemicals-under-tsca/strategic-plan-reduce-use-vertebrate-animals-chemical)
- EPA views the term New Approach Methodologies (NAMs) as equivalent to alternative test methods and strategies (the language in the statute)

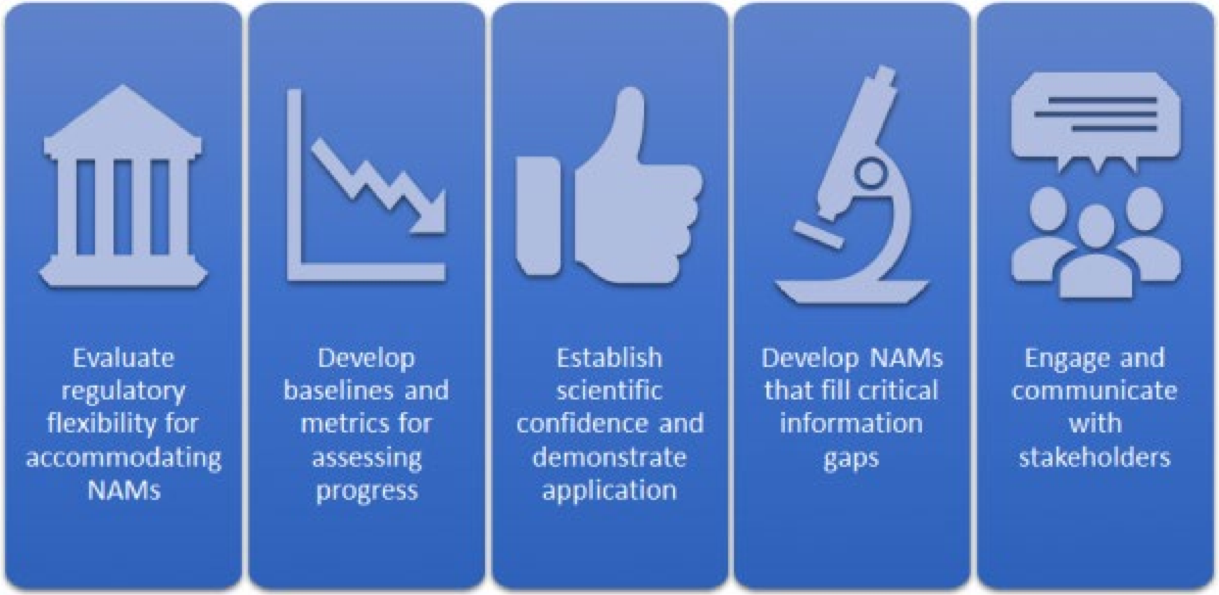

- EPA Work Plan for Reducing Use of Animals in Chemical Testing published June 2021 (https://www.epa.gov/chemical-research/epa-new-approach-methods-work-plan-reducing-use-animals-chemical-testing)

United States Environmental Protection Agency

NAMs: Strategy for Success

- **Strategic plan** components
  - ID, Develop, Integrate
  - Build confidence
  - Implement
- Demonstrated approach for skin sensitization adapted to inhalation
- **Create context** to advance understanding
  - **Target** *in vitro* assays to **evaluate key events** in various AOP
  - **Bridge acute to chronic** pathogenesis

Fig. 1 Core Components of EPA Strategic Plan to Develop and Implement New Approach Methodologies (NAMs) in TSCA

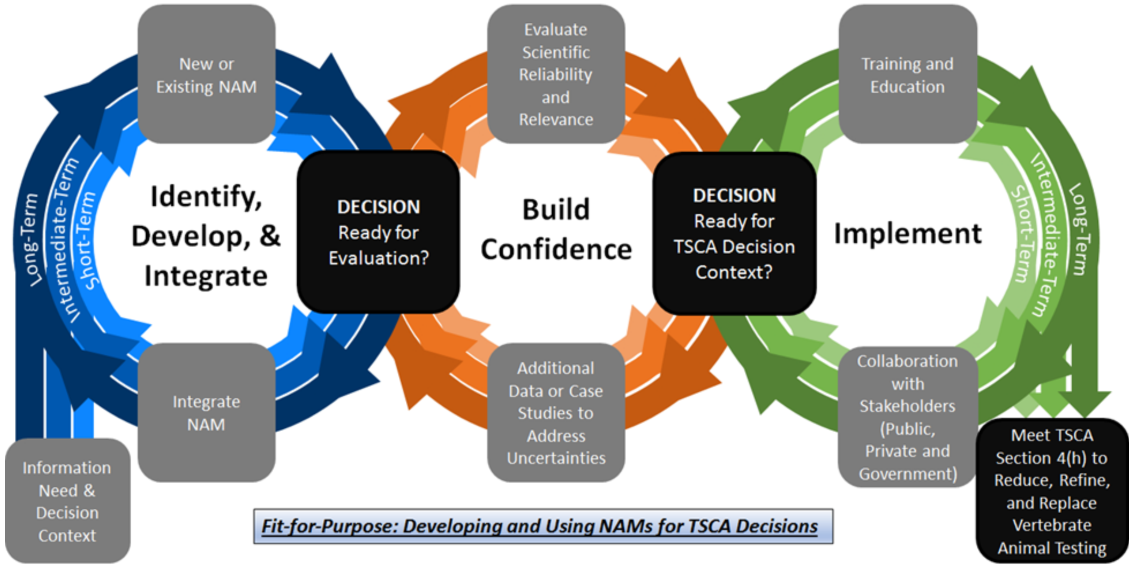

Toxicology in Vitro 52 (2018) 131–145

Contents lists available at ScienceDirect

Toxicology in Vitro

journal homepage: www.elsevier.com/locate/toxinvit

ELSEVIER

Review

Pathway-based predictive approaches for non-animal assessment of acute inhalation toxicity

Amy J. Clippinger[a,*], David Allen[b], Holger Behrsing[c], Kelly A. BéruBé[d], Michael B. Bolger[e], Warren Casey[f], Michael DeLorme[g], Marianna Gaça[h], Sean C. Gehen[i], Kyle Glover[j], Patrick Hayden[k], Paul Hinderliter[l], Jon A. Hotchkiss[m], Anita Iskandar[n], Brian Keyser[o], Karsta Luettich[n], Lan Ma-Hock[p], Anna G. Maione[k], Patrudu Makena[o], Jodie Melbourne[a], Lawrence Milchak[g], Sheung P. Ng[q], Alicia Paini[r], Kathryn Page[s], Grace Patlewicz[t], Pilar Prieto[r], Hans Raabe[c], Emily N. Reinke[u], Clive Roper[v], Jane Rose[w], Monita Sharma[a], Wayne Spoo[o], Peter S. Thorne[x], Daniel M. Wilson[m], Annie M. Jarabek[y]

Translation: AOP as Mechanistic Scaffold

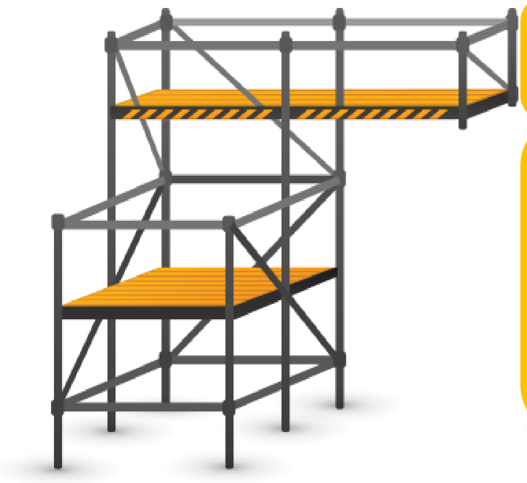

Target Site Exposure
- Solubility
- Vapor pressure
- Particle size, density, distribution
- Mass transfer coefficient
- Chemical reactivity
- ADME
- Breathing mode, rate and volume

*

Molecular Initiating Events
- Oxidation of cellular molecules
- Acetylcholinesterase inhibition
- Cytochrome C oxidase inhibition
- DNA/protein alkylation
- Modulation of ion channels
- Receptor binding e.g.,
  - *Activation of EGFR*
  - *Activation of TRPA1 receptor*
  - *Activation of glucocorticoid receptor*
  - *Activation/inhibition of G protein coupled receptors*
  - *Inhibition of muscarinic acetylcholine receptors*
  - *Inhibition of NMDA receptors*
  - *Binding to hormone receptor*

Cellular Key Events
- ROS formation
- Antioxidant (e.g., glutathione) depletion
- Inhibition of energy (ATP) production
- Cytotoxicity
- Collagen deposition
- Increased mucous production
- Cytoskeleton disruption
- Cytokine/chemokine production
- Surfactant depletion
- Modulation of signal transduction pathways
- Inhibition of nucleotide synthesis
- Protein modification
- Modulation of protein synthesis
- Effects on the blood
- Vitamin interference

Tissue / Organ Key Events
- Cell proliferation
- Inflammatory response
- Cell transformation
- Squamous cell metaplasia
- Loss of epithelial barrier function
- Reduced ciliary beat frequency
- Goblet (mucous) cell hyperplasia, metaplasia, and proliferation
- Respiratory failure
- Tracheitis
- Bronchiolitis
- Alveolitis
- Pulmonary edema
- Bronchoconstriction
- Alveolar distention
- Smooth muscle remodeling
- Change in lung mechanics (resistance, compliance, pressure-volume curves, FEV1)

Organism / Population Responses
- Systemic toxicity
- Acute lethality
- Target organ effects (e.g., hepatotoxicity)
- Airway hyperreactivity
- Chemical narcosis

Clippinger et al (2018)
https://www.ncbi.nlm.nih.gov/pubmed/29908304

- **Mechanistic data** to **describe dose** characterize **key events (KE)**
- ***Transition* assays** from prioritization / hazard ID to **quantitative AOP (qAOP)** for ***in vitro* to *in vivo* extrapolation (IVIVE)**

Translation: Exposure Alignment

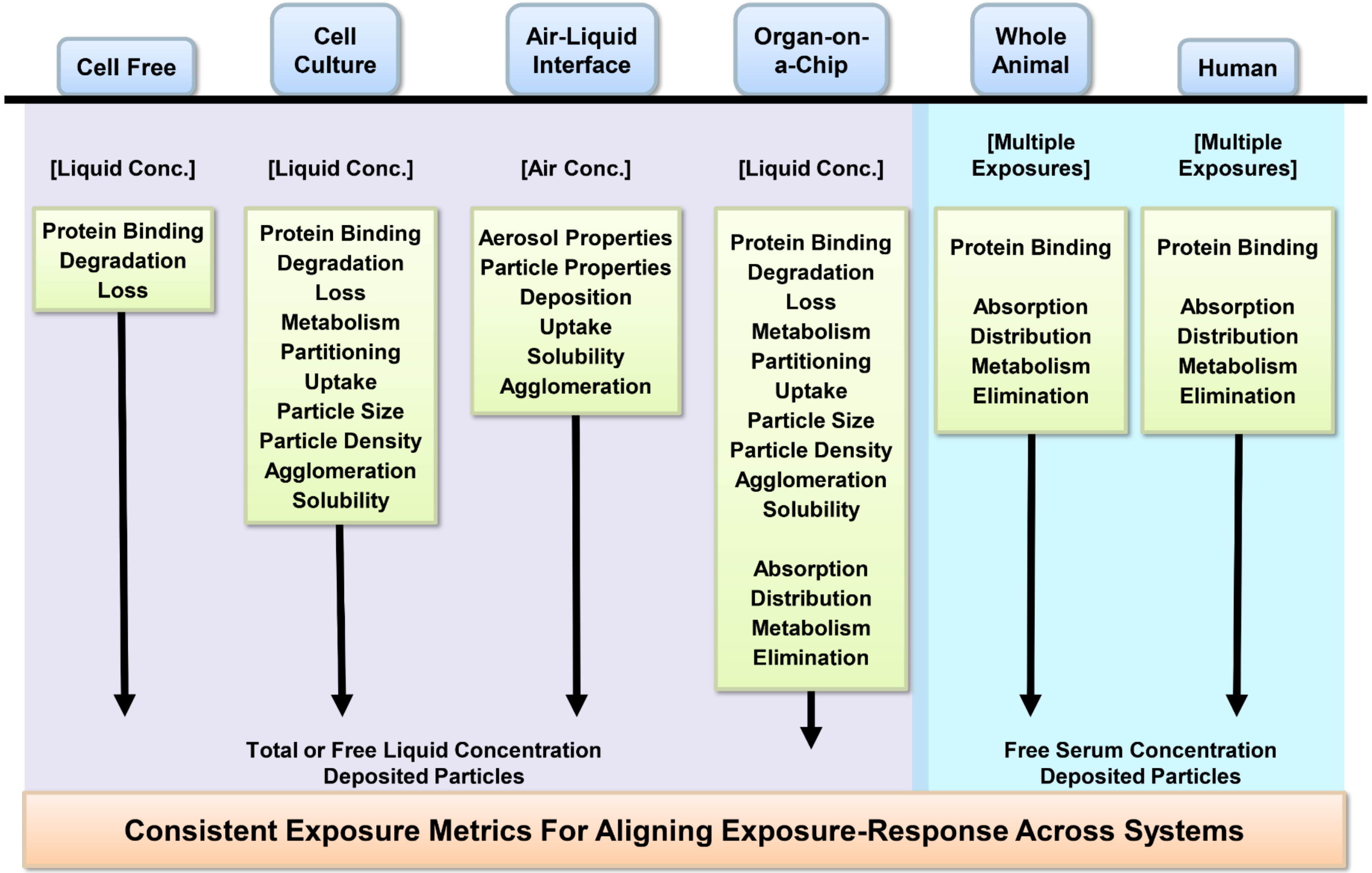

NAS (2017). Using 21st Century Science to Improve Risk-Related Evaluations
http://www.nap.edu/24635

Dosimetry Models in Risk Assessment

- **“Dose”**
  - Exposure versus internal amount at target site of exposure (e.g., deposited or retained; tissue / cell / molecular)
  - *Defined best as causal or at least a metric best associated (correlated) with toxicity or key event / endpoint used to evaluate “dose-response” relationship*
- **“Metric”**
  - Measurement: mass, surface area (SA), number (#); peak concentration, AUC
  - Scale of metric should be same as observation or the key event used as response endpoint (e.g., lung region versus local, specific cell type)
  - Motivate based on understanding of mode of action
- **“Model”**
  - Conceptual or quantitative description of important processes
  - Simulate different exposure scenarios and experimental designs

Translation: Mechanistic Modeling

- **Evolves empirical modeling** (observations of **WHAT**) → to ***HOW*** **and** ***WHY*** they occur
  - **Qualitative agreement** with current **biological understanding** of ADME and pathogenesis processes
  - **Quantitative agreement** with **test measures of key events**
- Incorporates important **physicochemical properties**
- **Translates** dose across various **experimental designs** to improve **data integration**
- **Addresses differences** between test systems, species and humans to **refine inferences**
- **Quantifies** and explores properties **systematically and consistently**

Translation: TSE Alignment and Quantitative AOP

- Account for **key characteristics of exposure**
- Address **physicochemical properties** as determinants of internal dose
- **Characterize** anatomical or physiological parameters and processes determining **dosimetry / ADME**
- **Describe *quantitative* relationships among key events (KE) in an AOP**

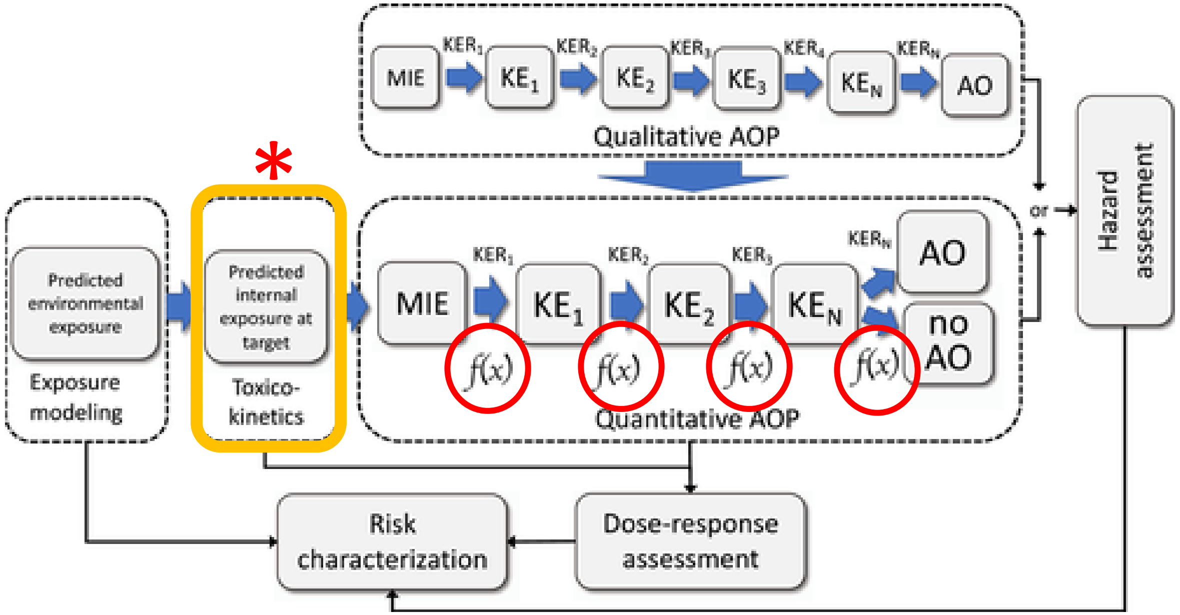

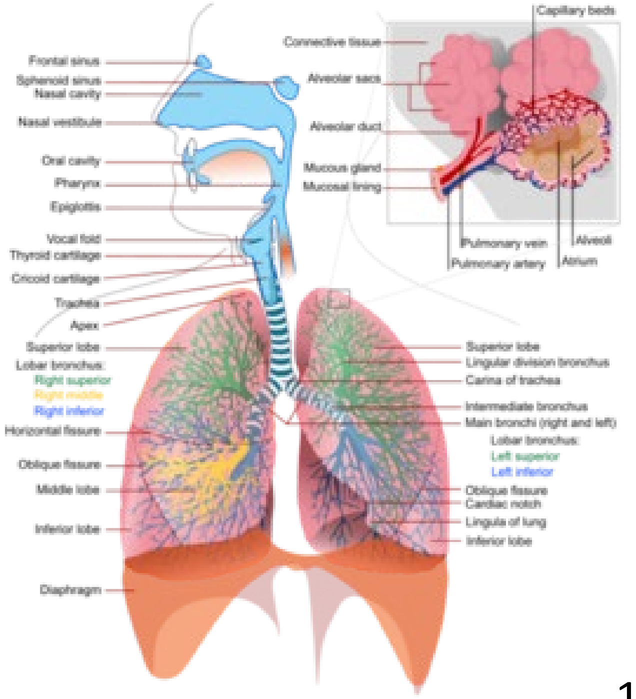

Perkins et al (2019)
https://www.ncbi.nlm.nih.gov/pubmed/31127958

Conceptual Basis of Extrapolation

Not to scale

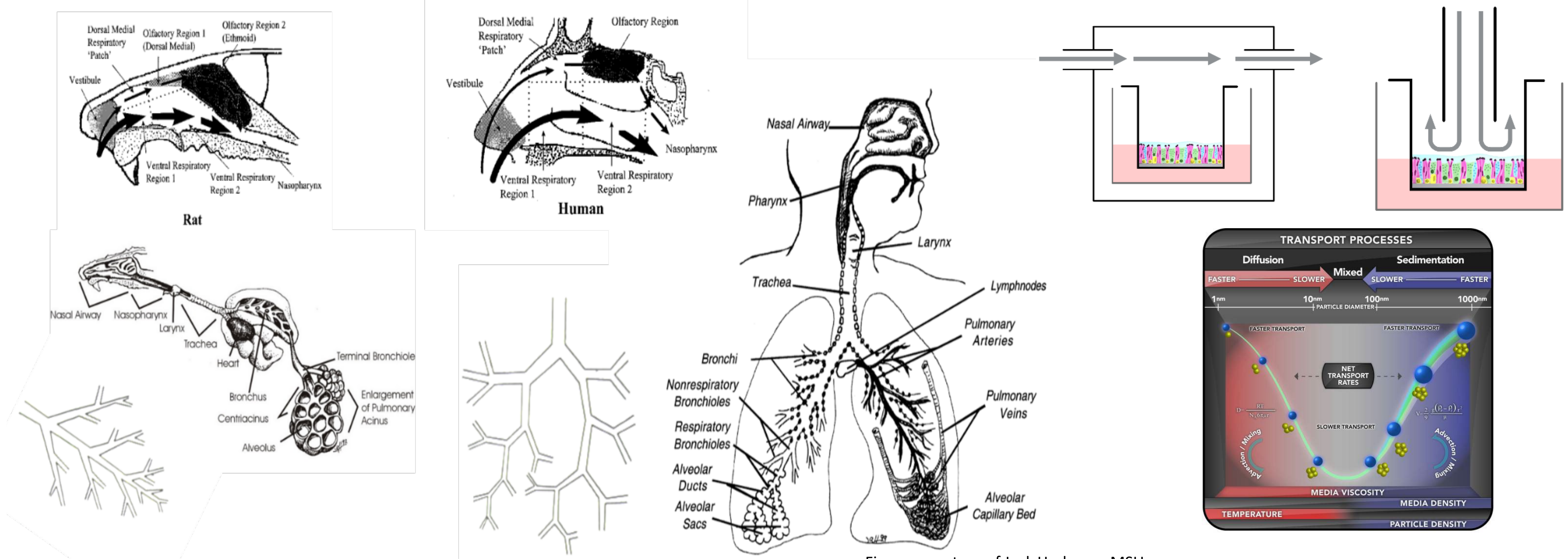

Figures courtesy of Jack Harkema, MSU

- To **integrate** human / laboratory animal and *in vitro* data need to **systematically** account for differences in
  - **Exposure systems and regimen** (e.g., occupational vs laboratory vs *in vitro*)
  - **Anatomy** (e.g., species and age-specific architecture)
  - **Physiology** (e.g., breathing mode and ventilation activity pattern)

Physicochemical Properties

Particle / Fibers / Manufactured Nanomaterials

- Density / Dimensions and Distribution
- Hygroscopicity
- Shape and surface area
- Agglomeration state

Determine aerodynamics and deposition

- Solubility and dissolution rate
- Crystal structure
- Chemical composition (spatially averaged (bulk) and heterogenous)
  - Physiosorption or chemisorption of biomolecules (e.g., proteins)
  - Biochemically-induced changes in surface chemistry
- Surface chemistry
- Surface charge (Zeta potential)
- Porosity

Exposure ≠ internal dose

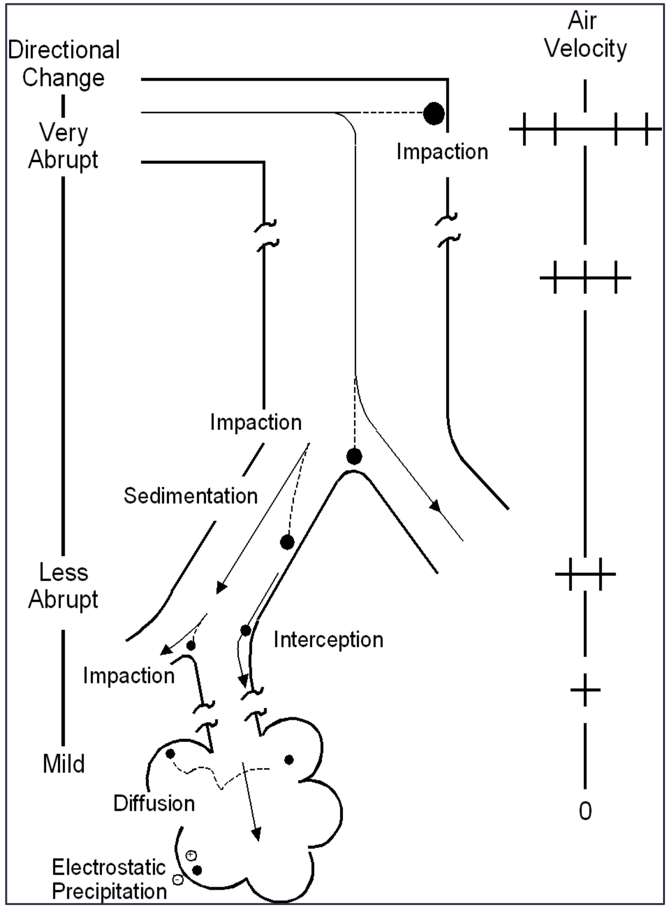

Retained burden = (Inhalability + Deposition) - Clearance

Note: Relative contribution of each mechanism is different in each region of respiratory tract

Physicochemical Properties

Gases

- Molecular diffusivity
- Reactivity
  - Hydrolysis
  - Protein binding
  - Metabolism / tissue reactions
- Solubility
  - Blood:air and blood:tissue partition coefficients

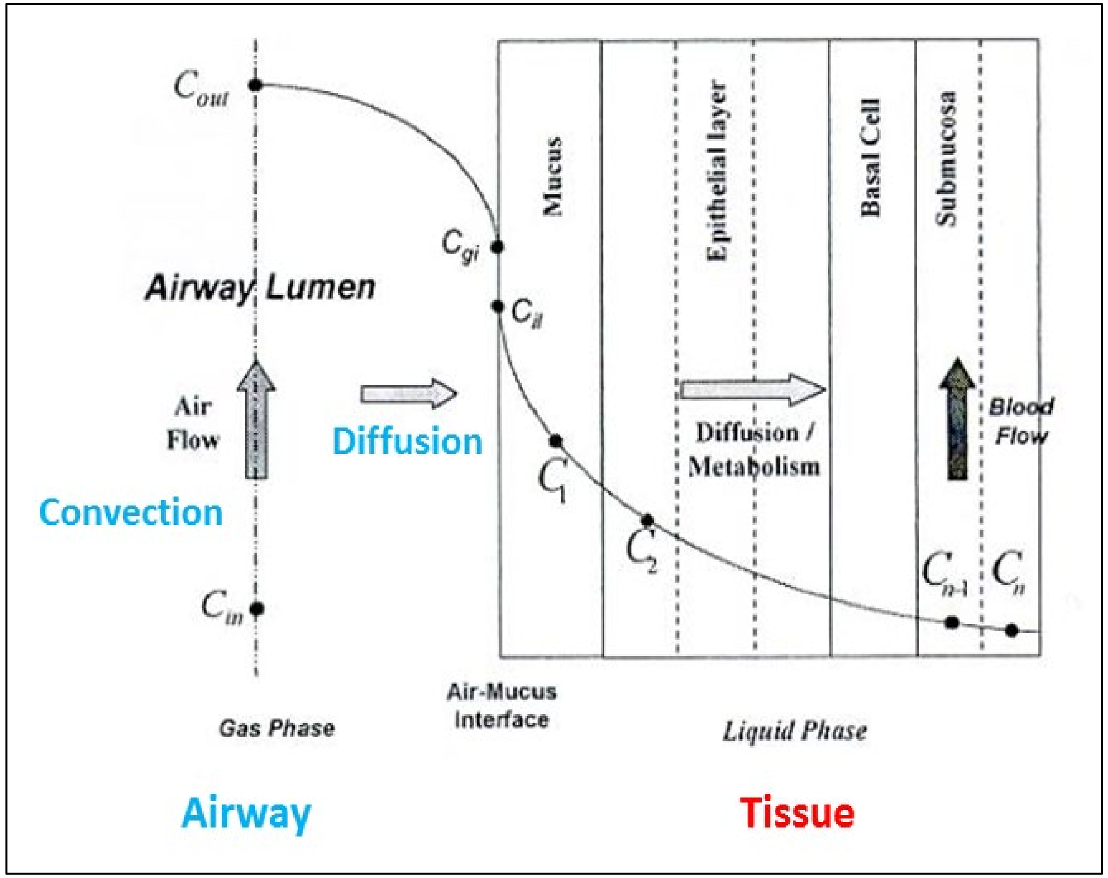

Bogdanffy and Jarabek (1995). *Toxicol Lett* 82-83:919-32.
https://www.ncbi.nlm.nih.gov/pubmed/8597163

Bogdanffy et al. (1999). *Toxicol Sci* Sep;51(1):19-35.
https://www.ncbi.nlm.nih.gov/pubmed/10496674

Dosimetry Deployed to Compute the TSE

- **Range** from default to sophisticated forms
- Differ by **physicochemical property**
  - Particle: MPPD and CFD
  - Gas: CFD, PBPK, hybrid PBPK-CFD
- Account for **key characteristics of exposure:**
  - Concentration, duration, and frequency
  - Regimen: Acute, episodic, ambient (constant), workplace
- Characterize **anatomical and physiological determinants** of ADME
  - Breathing rate, mode (oral, nasal), ADME and metric
- **Determine dose** in exposure test system
  - Submerged vs. air-liquid interface
  - Choice of cell type

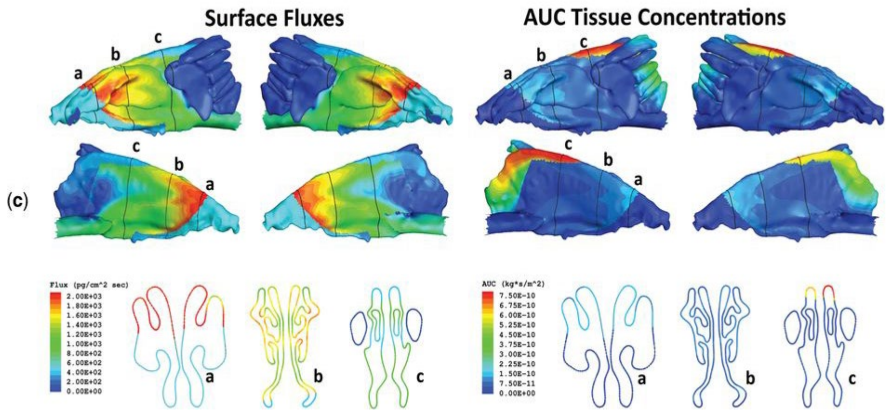

Corley et al. *Toxicol. Sci.* 2015;146:65-88

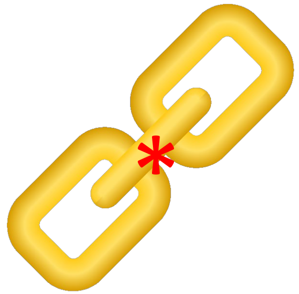

Translate TSE to Human Equivalent Concentration (HEC)

- **Account** for PC and ADME **determinants in test system**
  - Mass per volume of cell media and surface area differs across transwell sizes
  - $[\text{Toxicant}]_{\text{reported}} \neq [\text{Toxicant}]_{\text{applied}} \neq [\text{Toxicant}]_{\text{aqueous}}$ due to analytical issues and losses to media, plate, etc.
- **Adjust** relative to human target and conditions: ***Ratio to appropriately normalize***
- Illustrated for **regional deposited dose (RDD)** of particles in animals (A) ***or in vitro (*)*** and humans (H) but can be calculated for any other **particle dose metric** (SA, #) or **normalizing factor** (# epithelial cells, # alveolar macrophages)
- **Minute volume** can be age-specific and incorporate a ventilatory activity pattern reflecting breathing mode (nasal, mouth, oronasal)

$$(\text{RDDR})_r = \frac{(\text{RDD})_{A*}}{(\text{RDD})_H} = \frac{(C_1)_{A*}}{(C_1)_H} \Big/ \frac{(\text{Normalizing Factor})_{A*}}{(\text{Normalizing Factor})^{‡}_H} \times \frac{(\dot{V}E)_{A*}}{(\dot{V}E)_H} \times \frac{(F_r)_{A*}}{(F_r)_H}$$

$(\dot{V}E)$ = Minute volume (ventilation rate)

F_r = fraction of mass deposited in region predicted with model

r = Region of observed toxicity for extrapolation

‡ = Surface area (SA) for respiratory effects and body weight (BW) for remote effects

Case Study: New Chemical Substances (NCS) under TSCA

- Section 5 of TSCA does not require upfront testing for NCS; only extant data need be submitted
- Various methods used to assess risks with limited data
  - Chemical categories based on comparator chemicals
  - “Read across“ approaches using analogues
- Newly proposed integrated approach to testing and assessment (IATA) based on dosimetry modeling and AOP-inspired NAMs (SOT 2021)
  - General surfactants (Henry et al.; SOT Poster #2583)
  - Poorly soluble low toxicity (PSLT) polymers (Jarabek Stedeford et al.; SOT Poster #2593)
- Manuscripts undergoing re-submission to Chemical Research Toxicol

Integrated Approach to Testing and Assessment (IATA)

- Dosimetry plays critical role in strategy for evidence integration and evaluation to aid assessments
  - Inclusion criterion based on physicochemical (PC) properties
  - Translation of dose across experimental platforms
  - Target specific exposures
- NAMs can provide data to
  - Inform both PC properties and health effects based on AOP
  - Refine model parameters (e.g., solubility rates)

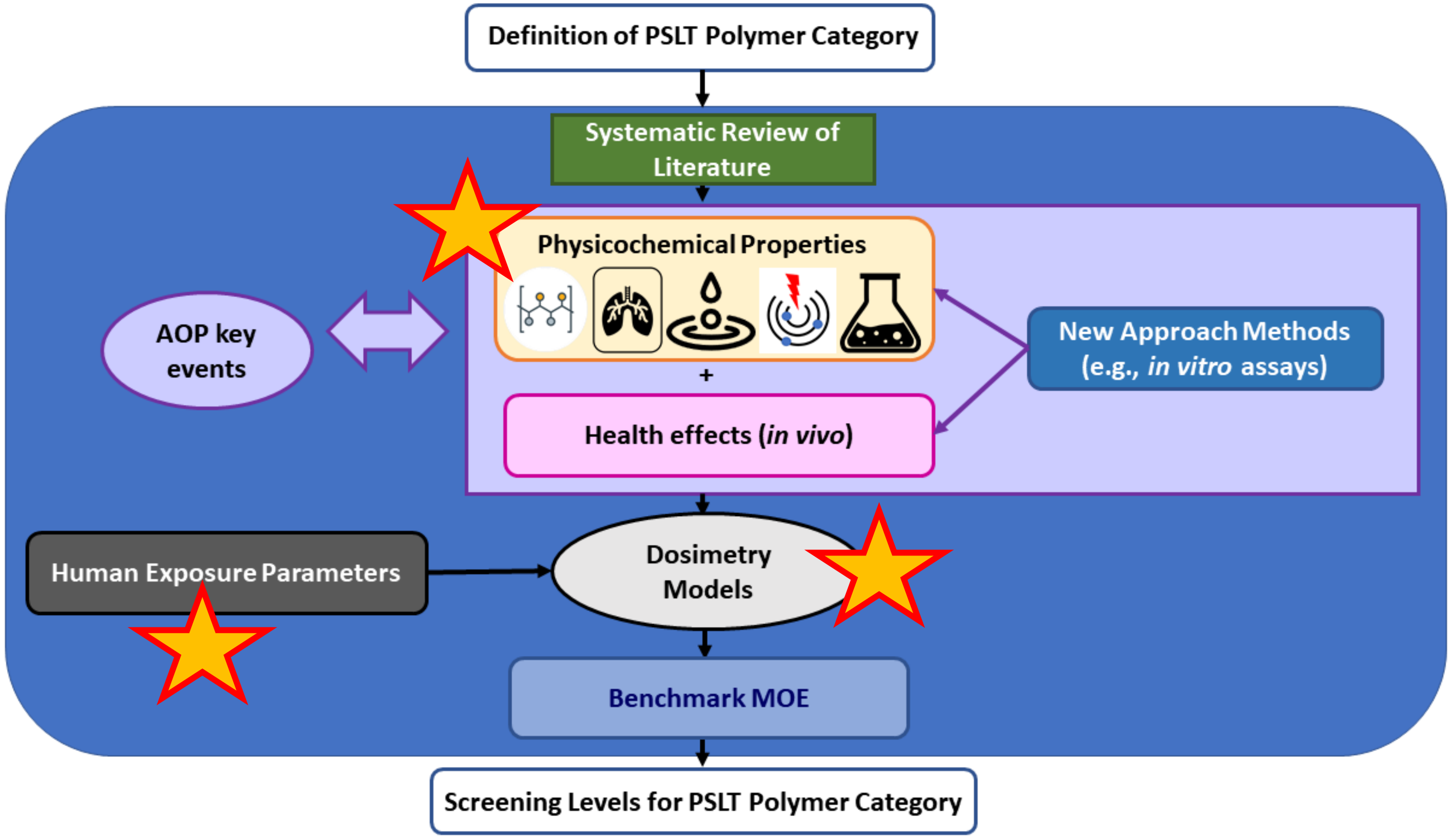

Jarabek Stedeford et al. (*accepted*)

MPPD Model to Calculate HEC: PSLT Polymers

- **Human equivalent concentration (HEC)** based on **extrapolation** of laboratory animal data
- **Multiple-path particle dosimetry (MPPD) model** deployed to **simulate** both the laboratory animal **exposure regimen** (e.g., 6 hr/day and 5 days/week for 28 days) and the human exposure scenario (e.g., occupational 8 hr/day and 5 days/week for 40 years)
- Human exposure scenario can be **default or targeted (*)** with specific data
  - Different particle distribution
  - Various ventilation parameters

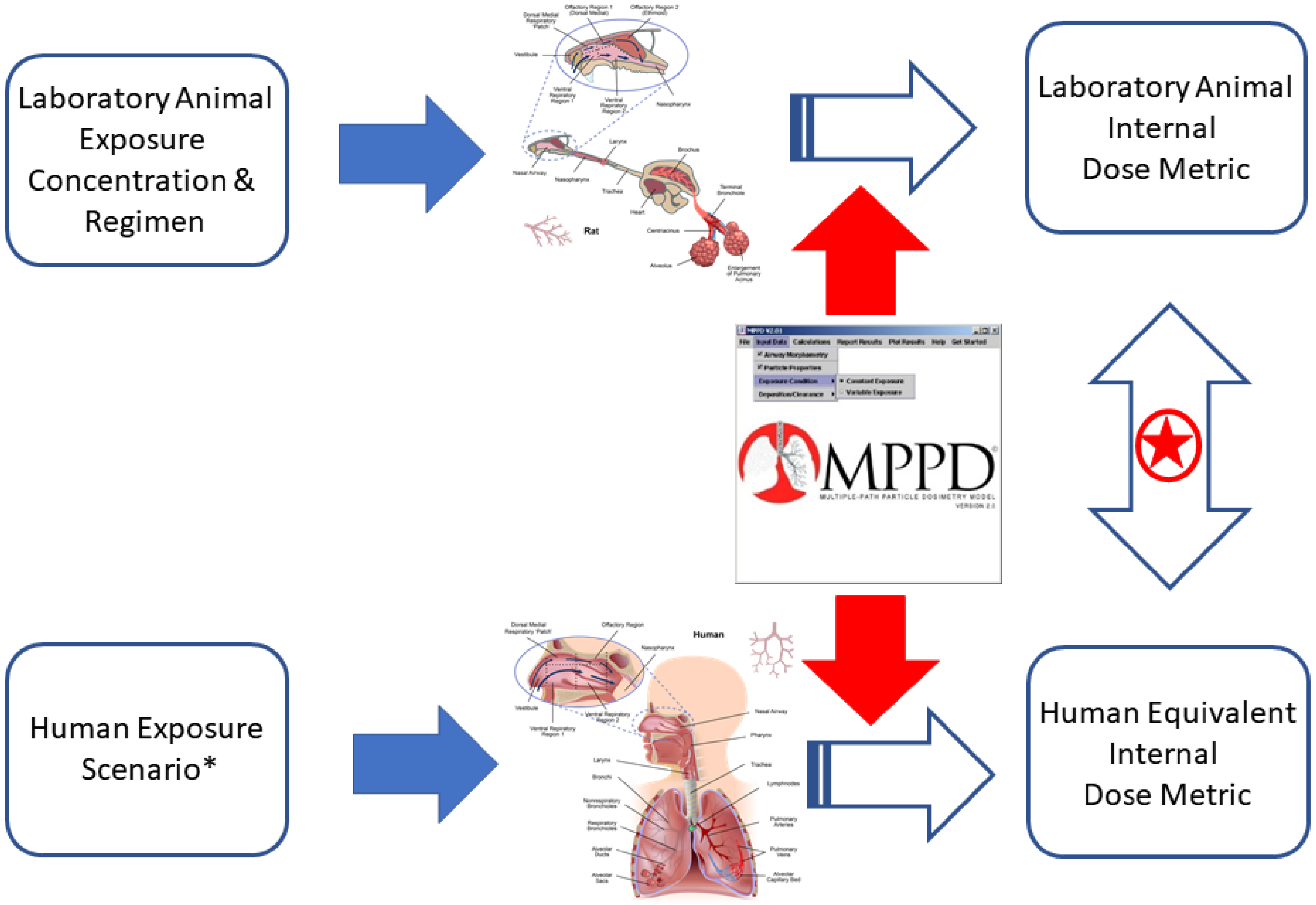

AOP-*Inspired* Integrated Approach to Testing and Assessment

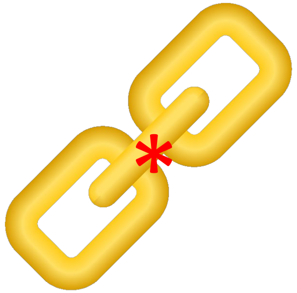

Identify
- Problem formulation: Target exposure
- Inhaled agent

Evaluate
- Existing information
- Physicochemical properties

Predict
- Dosimetry model to predict internal dose and location based on ADME and AOP

Choose
- Exposure system
- Cell types and assays

Test
- Perform battery based on key events in AOP

Align
- Translate (IVIVE) and integrate results in context with available knowledge base

Jarabek et al. (*in preparation*)

United States Environmental Protection Agency

Communication Best Practices: Reporting Standards Roadmap

- **Data sharing: Standards**
  - MIAME: Minimum Information About a Microarray Experiment
  - SEND: Standard for Exchange of Non-clinical Data
- **FAIR Principles: Findable / Accessible / Interoperable / Reusable**
  https://www.ncbi.nlm.nih.gov/pmc/articles/PMC4792175/pdf/sdata201618.pdf
  - **Translate TSE across exposure systems to aid evidence integration**
    - Exposure system operating parameters and conditions
    - Rationale for choice of cells and assays
    - Modular, multi-scale dosimetry to support interoperability
  - **Data pipelines and analytical work flows: Meta data**
    - Experimental annotation: ***WHAT / HOW / WHY***
    - Curation and consistency: Domain expertise and detail
    - Interdisciplinary dialogue
    - ***Repurposing: Applicability***

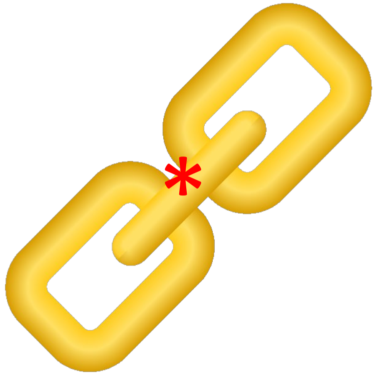

United States Environmental Protection Agency

Reporting Standards: Exposure Systems

- **Generation system and specifications**
  - Dimensions and volume
  - Air flow rate
  - Delivery mechanism(s)
  - Plate size and number, inserts
- **Concentration** (delivered relative to nominal should be consistent)
- **Analytical methods**
- **Temperature**
- **Humidity**
- **Relevance to target scenario**
  - Regimen and duration
  - Physicochemical characteristics
    - Gas: Mass transfer determinants
    - Particle: Deposition mechanisms

Jarabek et al. (*in preparation*)

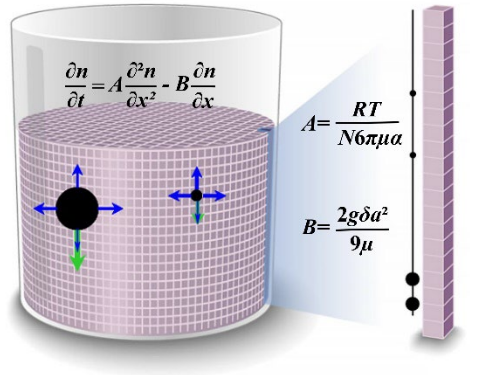

Hinderliter *et al.* 2010. *Part Fibre Toxicol.* 7(1) 36
https://nanodose.pnnl.gov/default.aspx?topic=ISDD

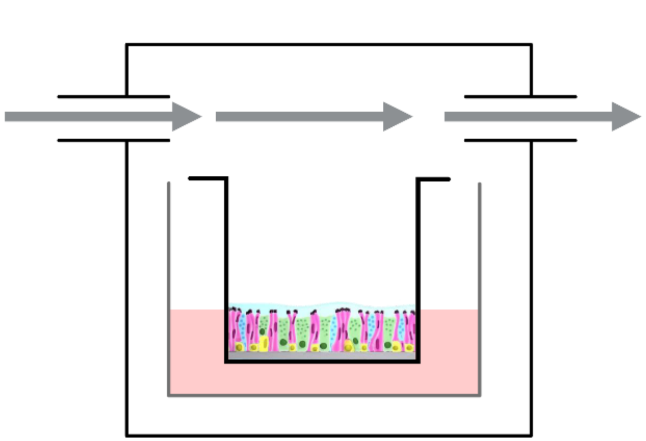

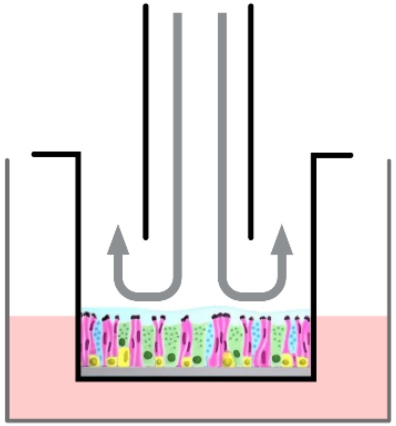

Reporting Standards: Cell Systems

- **Culture system**
  – Demonstrated reliability
- **Cell type(s)**
  – Source(s)
  – Metabolic competency
  – Rationale for choice (e.g., relevance to target scenario)
- **Media**
  – Type (components / lot #)
  – Location (epithelial or endothelial)
  – Volume
- **Viability**
  – Evaluation
  – Duration

Jarabek et al. (*in preparation*)

- **Assays**
  – Relevance to key events and respiratory tract
  – Established performance and variability
  – Response levels and rationale

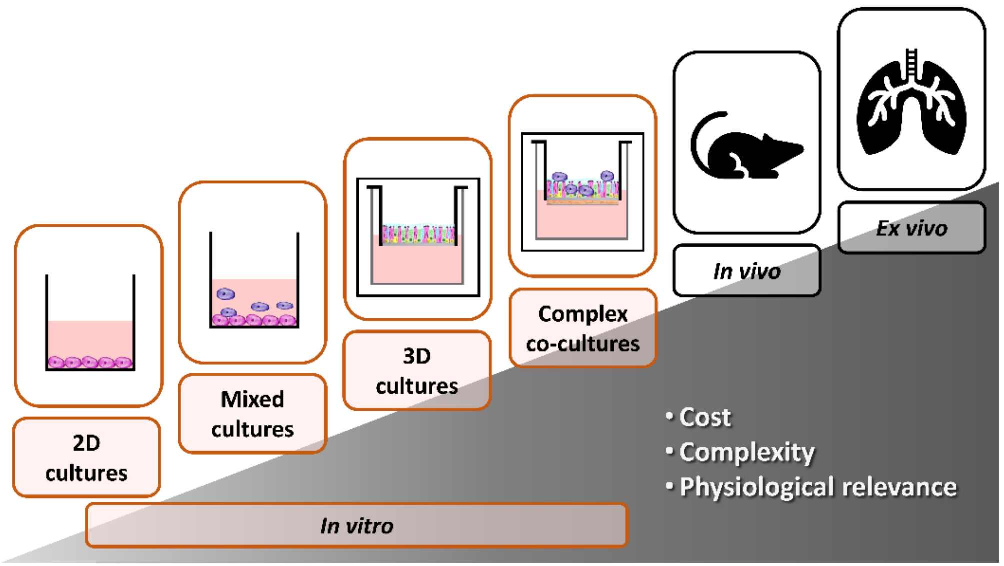

Figure adapted from Lacroix et al. (2018). *Appl in vitro Tox*, 4(2), 91 – 106. https://www.liebertpub.com/doi/full/10.1089/aivt.2017.0034

United States Environmental Protection Agency

Characterization: Translation Factors

- **Traditional factors of uncertainty and variability**
  - **Intrahuman:** Variability within the human population, including susceptible subpopulations, due to differences in life stage, disease states, and other determinants of TK or TD
  - **Interspecies (across experimental systems):** Differences in TK and TD
  - **Duration:** Use of acute data to predict episodic or chronic exposure outcomes
  - **Severity:** Nature of effect and prognostic value
  - **Database:** Coverage to comprehensively address potential effects
- **Novel translations:** Cell system as target tissue / system surrogate
  - Target tissue specificity and viability
  - Spatial representation and variability of sample
  - Metabolic competency and variability

The National Academies of SCIENCES ENGINEERING MEDICINE | ENVIRONMENTAL STUDIES AND TOXICOLOGY

New Study Committee Announcement:
Variability and Relevance of Current Laboratory Mammalian Toxicity Tests and Expectations for New Approach Methods (NAMs) for use in Human Health Risk Assessment

DEADLINE: Sunday, August 29, 2021

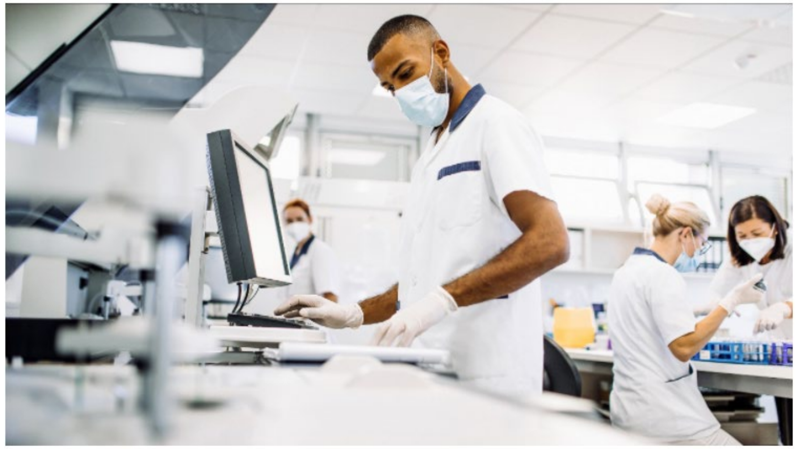

EPA United States Environmental Protection Agency

Impacts: Inferences and Integration

- Clarify terminology
  - “Model”
  - Effects, relationships and outcomes
- Evaluate new data resources
- Incorporate computational outputs
- Rectify units
- Elucidate study quality and utility
- Inform “causality” considerations
- Illuminate assumptions
- Support reusability and interoperability
- ***TRANSFORM translation and improve evidence integration***

United States Environmental Protection Agency

Summary: Advancing NAMs

- **Evolve empirical modeling** (observations of **WHAT**) → to **MECHANISTIC MULTISCALE MODELS** (***HOW*** and ***WHY***)
- Bridge to systems biology with **Integrated Approaches to Testing and Assessment (IATA)**: **key events** of pathogenesis and ***quantitative*** **AOP (qAOP)**
  - **Characterize** dose and effects at different **levels of observation**
  - **Understand** various **dimensions of disease** and relationships (e.g., early or late)
- **Translate targe site exposure** (**TSE) across exposure systems** to aid and transform **evidence integration: develop ANALYTIC WORKFLOWS**
  - **Align** human and animal **exposures**
  - **Refine** in vitro to in vivo extrapolation (**IVIVE**)
- Facilitate **interdisciplinary dialogue**
  - **Transparency** re: assumptions and foundational data
  - Appreciate **assumptions and impacts**
  - Support **modularity for interoperability** with other models

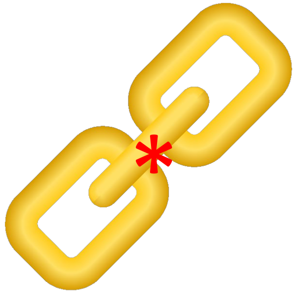

Thanks and Contact Information

Annie M. Jarabek

Jarabek.Annie@epa.gov

919-637-6016

SOT Poster #2583 | Surfactants Category: An Integrated Approach to Testing and Assessment (IATA) Including New Approach Methods (NAMs) for Assessing Inhalation Risks under the Toxic Substances Control Act (TSCA)

***T.R. Henry*[1], K.D. Salazar[1], M.P. Hayes[2], W. Kennedy[3], *A.M. Keene*[3], *A.M. Jarabek*[4], O.T. Price[5], S. Moors[6], *L. Jovanovich*[7], J.L. Rose[8], *A. Tveit*[9], R.T.Tremblay[10], *R.A. Becker*[11], S. Osman-Sypher[11], *P.D. McMullen*[12], *S.D. Slattery*[12], *W. Irwin*[1], M. Odin[13], *J. Melia*[13], *M. Sharma*[14], A.O. Stucki[14], *A.J. Clippinger*[14], and *T. Stedeford*[1].** [1]US EPA, Washington, DC; [2]Procter & Gamble, St. Bernard, OH; [3]Afton Chemical Corporation, Richmond, VA; [4]US EPA, Research Triangle Park, NC; [5]Applied Research Associates, Inc., Arlington, VA; [6]BASF Corporation, Duesseldorf, Germany; [7]Stepan Company, Northfield, IL; [8]Procter & Gamble, Mason, OH; [9]BASF Corporation, Florham Park, NJ; [10]Procter & Gamble, Strombeek-Beaver, Belgium; [11]American Chemistry Council, Washington, DC; [12]ScitoVation, Durham, NC; [13]SRC Inc., North Syracuse, NY; and [14]PETA Science Consortium International e.V., Stuttgart, Germany.

SOT Poster #2593 | Poorly Soluble, Low Toxicity (PSLT) Polymer Category: An Integrated Approach to Testing and Assessment (IATA) Including New Approach Methods (NAMs) under the Toxic Substances Control Act (TSCA)

***A.M. Jarabek*[1], *T. Stedeford*[2], *G.S. Ladics*[3], O.T. Price[4], *A. Tveit*[5], M.P. Hayes[6], R.T. Tremblay[7], *S.A. Snyder*[8], K.D. Salazar[2], S. Osman-Sypher[9], *W.Irwin*[2], M. Odin[10], *J. Melia*[10], *H. Carlson-Lynch*[10], *M. Sharma*[11], A.O. Stucki[11], *A.J. Clippinger*[11], S. Anderson[3], and *T.R. Henry*[2].** [1]US EPA, Research Triangle Park, NC; [2]US EPA, Washington, DC; [3]IFF, Wilmington, DE; [4]Applied Research Associates Inc., Arlington, VA; [5]BASF Corporation, Florham Park, NJ; [6]Procter & Gamble, Mason, OH; [7]Procter & Gamble, Strombeek-Beaver, Belgium; [8]Covestro LLC, Pittsburgh, PA; [9]American Chemistry Council, Washington, DC;[10] SRC Inc., North Syracuse, NY; and [11]PETA Science Consortium International e.V., Stuttgart, Germany.
